# Expression Microarray Meta-Analysis Identifies Genes Associated with Ras/MAPK and Related Pathways in Progression of Muscle-Invasive Bladder Transition Cell Carcinoma

**DOI:** 10.1371/journal.pone.0055414

**Published:** 2013-02-01

**Authors:** Jonathan A. Ewald, Tracy M. Downs, Jeremy P. Cetnar, William A. Ricke

**Affiliations:** 1 Department of Urology, University of Wisconsin School of Medicine and Public Health, Madison, Wisconsin, United States of America; 2 Department of Medicine, Hematology/Oncology Unit, University of Wisconsin School of Medicine and Public Health, Madison, Wisconsin, United States of America; 3 University of Wisconsin Carbone Cancer Center, Madison, Wisconsin, United States of America; University of Kentucky College of Medicine, United States of America

## Abstract

The effective detection and management of muscle-invasive bladder Transition Cell Carcinoma (TCC) continues to be an urgent clinical challenge. While some differences of gene expression and function in papillary (Ta), superficial (T1) and muscle-invasive (≥T2) bladder cancers have been investigated, the understanding of mechanisms involved in the progression of bladder tumors remains incomplete. Statistical methods of pathway-enrichment, cluster analysis and text-mining can extract and help interpret functional information about gene expression patterns in large sets of genomic data. The public availability of patient-derived expression microarray data allows open access and analysis of large amounts of clinical data. Using these resources, we investigated gene expression differences associated with tumor progression and muscle-invasive TCC. Gene expression was calculated relative to Ta tumors to assess progression-associated differences, revealing a network of genes related to Ras/MAPK and PI3K signaling pathways with increased expression. Further, we identified genes within this network that are similarly expressed in superficial Ta and T1 stages but altered in muscle-invasive T2 tumors, finding 7 genes (COL3A1, COL5A1, COL11A1, FN1, ErbB3, MAPK10 and CDC25C) whose expression patterns in muscle-invasive tumors are consistent in 5 to 7 independent outside microarray studies. Further, we found increased expression of the fibrillar collagen proteins COL3A1 and COL5A1 in muscle-invasive tumor samples and metastatic T24 cells. Our results suggest that increased expression of genes involved in mitogenic signaling may support the progression of muscle-invasive bladder tumors that generally lack activating mutations in these pathways, while expression changes of fibrillar collagens, fibronectin and specific signaling proteins are associated with muscle-invasive disease. These results identify potential biomarkers and targets for TCC treatments, and provide an integrated systems-level perspective of TCC pathobiology to inform future studies.

## Introduction

Bladder cancer is a disease receiving growing attention within the cancer biology community. Transition Cell Carcinoma (TCC) occurs as papillary tumors (Ta stage), superficial tumors (T1 stage), and muscle-invasive tumors of increasing severity (T2, T3 and T4 stage). Approximately 20% of primary bladder cancers are muscle-invasive at presentation and are associated with a poor prognosis, with 5 year survival estimates for muscle-invasive TCC approaching the low survival rates for advanced metastatic pancreatic cancers, small cell lung cancers, liver and bile-duct cancers, stomach and non-small cell lung carcinomas [1], [2]. Although papillary and superficial tumors recur in 70% of patients after surgical removal, non-invasive tumors have a more favorable outcome than muscle-invasive tumors as only 10–20% of these recurrences progress to muscle-invasive disease [2]. The regulatory mechanisms that are altered and disrupted in muscle-invasive bladder cancer may represent a barrier to progression in superficial tumors, and are candidate targets for therapeutic intervention.

Accumulating evidence suggests that superficial and muscle-invasive tumors are pathobiologically distinct [3], [4]. Superficial tumors frequently overexpress or express constitutively active mutants of HRAS and FGFR3 leading to hyperactivated Ras/MAPK signaling activity [3], [4]. Muscle-invasive tumors demonstrate disrupted activity of p53 and Rb and other tumor suppressors, overexpress EGFR and ErbB2, MMP2 and MMP9, and other pro-angiogenic factors, while having deleted cyclin-dependent kinase inhibitor genes CDKN2A (p16^Ink4a^) and CDKN2B (p15^Ink4b^) [4]. However, there is evidence that suggests that Ta tumors in some patients may progress and become muscle-invasive. In addition to observations that 10–20% of patients who initially have Ta tumors later develop muscle-invasive disease, tumors ≥T1 share common chromosomal deletions, gains and amplifications that are distinct from those found in Ta stage tumors, suggesting that accumulated chromosomal aberrations may be involved in the progression from papillary to muscle-invasive tumors [1], [2]. Moreover, while activating mutations in *FGFR3*, *ras* isoforms, and *PI3K* are more common in papillary tumors, most high grade superficial tumors lack these mutations and are similar to invasive tumors, suggesting that these may be predisposed to progress to muscle-invasive disease [5]. At the same time, some tumor recurrences lose the activating mutations that are present in earlier tumors, which may also potentially drive progression [5]. In all, the relationship of superficial and muscle-invasive bladder cancer remains controversial and largely unresolved.

There are few effective systemic treatments for muscle-invasive bladder cancer, and an improved understanding of the molecular pathogenesis and progression of TCC is urgently needed. Analysis mRNA expression in different stages of bladder cancer could illustrate differences that exist which may promote progression from Ta and T1 tumors to higher stage recurrences, while similarities in expression patterns could clarify the relationship of tumor stages in progression of the disease. Insights into these molecular mechanisms of bladder tumor progression can provide targets for preventative and therapeutic interventions while providing biomarkers that reliably predict progression into muscle-invasive disease.

Microarray technology provides a powerful tool to measure mRNA expression across the entire genome of biological samples, allowing detailed analysis of large numbers of experimental and clinical samples in relatively little time. Typically in studies of various cancers, clinical samples are analyzed to identify genes expressed at relatively high or low amounts in common patterns that correlate with tumor stage and patient survival [6]. While this approach may be appropriate for identifying potentially useful diagnostic and prognostic biomarkers, it is often difficult to determine whether signature genes in advanced cancers are functionally relevant to tumor progression. Strategies to derive functional information from gene-expression datasets include expression clustering to identify genes with similar patterns of expression [7]and pathway enrichment programs including WebGestalt [8] that query Gene Ontology, Kyoto Encyclopedia of Genes and Genomes (KEGG), and other databases to identify processes in which gene expression changes are focused. Another strategy is to analyze the published literature using automated text-mining programs such as PubGene and Chilibot to identify functional associations between genes, phenotypes and diseases within publications indexed in PubMed [9]–[12]. Such resources have made computational informatic tools available to scientists who need to analyze and interpret microarray data, yet few studies have taken advantage of this information.

Studies of whole-genome expression using microarray technology require resources that may not be widely available to all researchers. Reliable microarray studies require large numbers of samples to provide for an adequate analysis and can be prohibitively expensive and time consuming. Access to human tissues required for relevant analyses is often not available to researchers outside of the medical field. The establishment of public microarray data archives, including the Gene Expression Omnibus (http://www.ncbi.nlm.nih.gov/geo/), has allowed free-access to a wealth of clinical and experimental data that can be analyzed and re-purposed by any investigator with the interest and the means to do so. The development of open-access analytical programs, including Oncomine (https://www.oncomine.org) [13] and Mayday [14], provide a means to investigate gene expression in data across multiple microarray studies. These advances allow researchers to pursue genome-scale investigations in clinically-derived human tissues without the time, expense, and administrative efforts associated with generating primary genomic data.

Here, we use public data to identify genes that are associated with TCC tumor progression and muscle-invasive disease in male patients without metastases. We find that the differential expression of a network of genes related to the mitogenic Ras/MAPK and related signaling pathways are associated with progression beyond Ta stage. Additionally, we identified a subset of 7 genes within this network whose expression is associated with muscle-invasive TCC.

## Materials and Methods

### Microarray Dataset

The Gene Expression Omnibus (GEO) website (http://www.ncbi.nlm.nih.gov/geo/) was used to search publicly available datasets for recent studies of bladder cancers with at least 3 samples representing all tumor grades and performed using up-to-date whole-genome microarray chips. We chose a dataset (GEO# GSE 31684; accessed 2/29/2012) which was originally used to develop predictive models of bladder cancer outcome [15], as it fulfilled these requirements and provided detailed patient data. Data was further selected from male TCC patients without associated metastases, carcinoma *in situ* or sarcoma to reduce potential variation in data, resulting in a total of 36 patient datasets (Table 1).

**Table 1:** Tumor stage, sample size and age range of data selected from GSE 31684 dataset.

Tumor Stage	n =	Age Range
**Ta**	3	57–78
**T1**	7	57–74
**T2**	10	51–80
**T3**	9	66–84
**T4**	7	50–83

### Data selection, calculations and statistics

The meta-analysis of public data was performed according to PRISMA guidelines (Table S1, Table S2). Microarray expression data was organized, managed, and calculated using Microsoft Excel (Redmond, WA). The microarray expression data of all 36 sample datasets were expressed in log_2_ scale, and the average expression values were calculated for samples of each pathological tumor grade. To identify general expression changes associated with progression of TCC, the gene probe expression values of tumor stage T1, T2, T3 and T4 samples were individually normalized to Ta by subtracting their log_2_ averages. Gene probes were selected based on both an increase or decrease in expression by a factor of 2 (log_2_1/−1) and statistical significance (p<0.05) in at least one tumor stage based on Student's t-test as a more stringent method of selection than either criterion alone. To identify expression changes associated with muscle-invasive disease, genes were selected that did not significantly change from Ta to T1 but are significantly different by a factor of 2 in T2 tumors in addition to significance based on Student's t-test. Further, the average expression values of selected gene probes in each tumor grade were compiled, and 3×3 matrix self-organizing map-based clustering analysis of relative gene expression across tumor stages was performed with 10,000 iterations using Mayday [14]. Clusters of gene probes with minimal expression changes, representing apparent false-positives, were arbitrarily omitted from subsequent analysis.

### Statistical pathway enrichment analysis of differentially expressed genes

To perform pathway enrichment analysis on selected genes, gene probe IDs were annotated with Entrez gene symbols (Affymetrix Human Genome U133 Plus 2.0 [HG-U133_Plus_2]; GEO# GPL570) and analyzed using the WebGestalt website (http://bioinfo.vanderbilt.edu/webgestalt/) to identify defined KEGG pathways that are significantly over-represented in the dataset [8], using default settings and the Fisher's Exact test, selecting pathways where p<0.01. These results produced lists of the genes included in the enriched pathways, and provided a connectivity map of each pathway. A model network based on these pathways was generated using the Visual Understanding Environment (http://vue.tufts.edu/). Gene expression heatmaps were generated using Mayday (http://www-ps.informatik.uni-tuebingen.de/mayday/wp/) [14].

### Statistical validation of muscle-invasive tumor gene signature using outside datasets

To determine whether muscle-invasive-specific expression changes identified in our initial dataset are typical across an expanded number of samples and studies, we obtained expression data for each gene compiled within the Oncomine database [13]. This included 8 datasets from 7 studies in which gene expression is measured in superficial and muscle-invasive bladder tumors [16]–[22]. The median expression of each gene in superficial and muscle-invasive tumors from each dataset was recorded and the averaged difference was calculated. A Mann-Whitney Rank-Sum test was performed to determine the significance of expression changes across datasets.

### Bibliomic text-mining for functional gene association

To obtain published results that link functional interactions of genes and phenotypes, selected genes were analyzed using PubGene [10], while genes and keywords “bladder cancer”, “metastasis”, “angiogenesis”, “invasive” were analyzed using Chilibot [11]. An association network based on co-occurrances and implied functional relationships determined by each program was constructed based on these results.

### Immunohistochemistry of Bladder Tumors

Samples (n = 5 each) of normal bladder and T1, T2 and T3 bladder tumors were purchased from Biomax.us (Rockville, MD). Samples were processed and stained by the TRIP Laboratory facility (Depatment of Pathology, University of Wisconsin School of Medince and Public Health) using standard immunostaining procedures. Antibodies to COL3A1 and COL5A1 were purchased from Santa Cruz Biotechnology (Santa Cruz, CA) and visualized using DAB and Warp Red stains. Nuclei were visualized using hematoxylin staining. Microscopy and image processing was performed as previously described [23].

### Western Immunoblotting

T24 bladder cancer cells and E6-immortalized human urothelial cells (HUCs) were the kind gift of Dr. Dale Bjorling, University of Wisconsin. T24 cells were cultured in DMEM media +10% FBS, while HUCs were cultured in Ham's F12 media +10% FBS. Cells were cultured to 70% confluence, scraped from plates, collected, solubilized and processed, analyzed by western immunoblotting, and quantified using Image J, as previously described [23]. Antibodies to COL3A1 and COL5A1 are described above. Antibodies recognizing β-actin were used as a loading control.

## Results

### TCC progression is associated with differential expression of Ras/MAPK and related pathway genes

**Figure 1:**
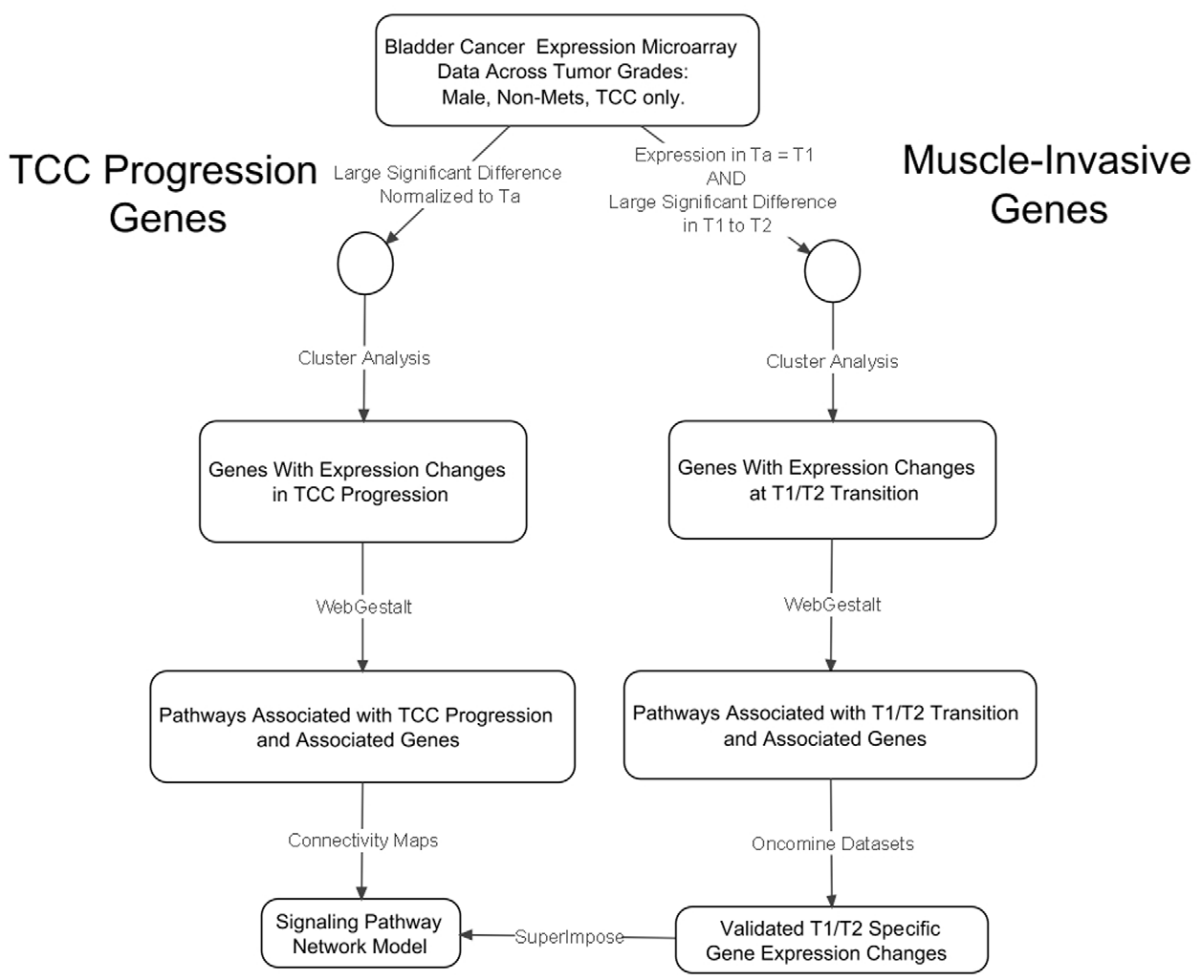
Flow diagram detailing the methods used to identify genes associated with TCC progression and muscle-invasive behavior from expression microarray data.

Our hypothesis was that gene expression differences exist between papillary/superficial and muscle-invasive bladder TCC tumors that define the regulation of tumor invasion, progression and metastasis. We developed an intuitive method, summarized in Figure 1, to select, analyze and interpret public microarray data using open access computational resources. We found a suitable dataset from the GEO database (GSE31684) containing expression data from 93 patient bladder tumors that were removed prior to any chemical treatment and used to identify gene expression signatures that predict outcome of high-risk bladder cancer [15]. To limit potential variation in sample expression data, sample data were selected from male patients with TCC of the bladder, without metastatic tumors, CIS or sarcoma, producing 36 total datasets that represented at least three patients per stage for all stages of TCC tumors (Table 1).

First, we calculated expression differences between Ta and T1–T4 bladder tumors to identify changes broadly associated with bladder cancer progression beyond papillary tumor stages. Data were processed, averaged, normalized to the expression of Ta tumors, and selected based on a greater than 2-fold threshold difference in expression at any stage beyond Ta, and p<0.05 using Student's t-test. This produced a list of 8110 gene-associated probes. Expression data was retrieved for those selected probes and analyzed using the self-organizing map cluster analysis within Mayday [14] to arbitrarily remove probes that reflect little change in expression, selecting 5509 total probes.

As receptor tyrosine kinases, mitogenic signaling pathways and tumor suppressors are each involved in superficial and muscle-invasive bladder cancer, our aim was to identify changes in gene expression that could compliment the activities of these pathways. The list of selected probes was analyzed with WebGestalt [8] to identify defined pathways that are significantly represented in the microarray data (p<0.05). Whether the selected genes were analyzed as a single group or separated based on increased or decreased expression, many of the pathways reliably associated with these genes are related to cancer-related signal transduction and similar pathways (Table 2, Table S3). The ten best supported pathways associated with the entire set of genes include “Cell Cycle”, “Pathways in Cancer”, and “Focal Adhesion”. Beyond these ten, “MAPK signaling”, “ErbB signaling” and “Bladder Cancer” pathways were also well supported. Likewise, when this list was separated into separate lists of 4816 increasing and 693 decreasing genes, many similar cancer-related signal transduction pathways were found to be represented in these genes. While other pathways represented in the data could also be associated with cancer progression and metastasis, such as “Metabolism” and “DNA Repair”, the relationship of these pathways to current models of bladder progression is less direct. These results suggest that bladder cancer progression from Ta to T1+ stage tumors involves the increased expression of genes in mitogenic, cancer-associated pathways related to FGFR3 and ErbB family signaling.

**Table 2:** KEGG pathway enrichment of genes differentially expressed in T1–T4 staged bladder tumors versus Ta-stage tumors.

KEGG Pathway	Number of Pathway Genes in Dataset	Enrichment Factor “R”	Fisher's Exact, Adjusted P-value
**All Changes, Top 10**			
Metabolic pathways	332	3.46	5.26E-93
Cell cycle*	68	6.11	2.46E-36
Spliceosome	68	6.11	2.46E-36
DNA replication	29	9.27	3.29E-23
Pyrimidine metabolism	47	5.52	1.05E-22
Ubiquitin mediated proteolysis	54	4.50	6.33E-21
Pathways in cancer*	86	3.00	2.50E-19
Focal adhesion*	63	3.61	1.30E-18
Huntington's disease	60	3.73	1.32E-18
Purine metabolism	52	3.96	1.75E-17
**Selected Pathways**			
MAPK signaling pathway*	60	2.57	6.88E-11
ErbB signaling pathway*	26	3.44	5.92E-08
Bladder cancer*	16	4.38	7.25E-07
**Genes Up, Top 10**			
Metabolic pathways	299	3.57	1.64E-85
Spliceosome	66	6.79	1.34E-37
Cell cycle*	62	6.38	2.13E-33
DNA replication	29	10.61	7.10E-25
Pyrimidine metabolism	46	6.18	3.64E-24
Ubiquitin mediated proteolysis	50	4.77	3.54E-20
Huntington's disease	58	4.13	7.22E-20
Purine metabolism	51	4.45	3.88E-19
Oocyte meiosis	42	4.85	1.95E-17
Alzheimer's disease	50	3.91	4.12E-16
Parkinson's disease	43	4.26	1.71E-15
**Genes Down, Top 10**			
Complement and coagulation cascades	11	14.12	1.22E-08
Pathways in cancer*	21	5.64	1.22E-08
Focal adhesion*	17	7.49	1.22E-08
MAPK signaling pathway*	17	5.60	4.00E-07
Small cell lung cancer	10	10.54	8.56E-07
Metabolic pathways	35	2.81	1.07E-06
p53 signaling pathway	9	11.55	1.31E-06
Tight junction	11	7.27	5.35E-06
NOD-like receptor signaling pathway	8	11.43	5.84E-06
Metabolism of xenobiotics by cytochrome P450	8	10.12	1.36E-05

(*) indicates KEGG pathways that involve mechanisms of signal transduction. P value represents the results of Fisher's Exact tests reported by WebGestalt.

**Figure 2:**
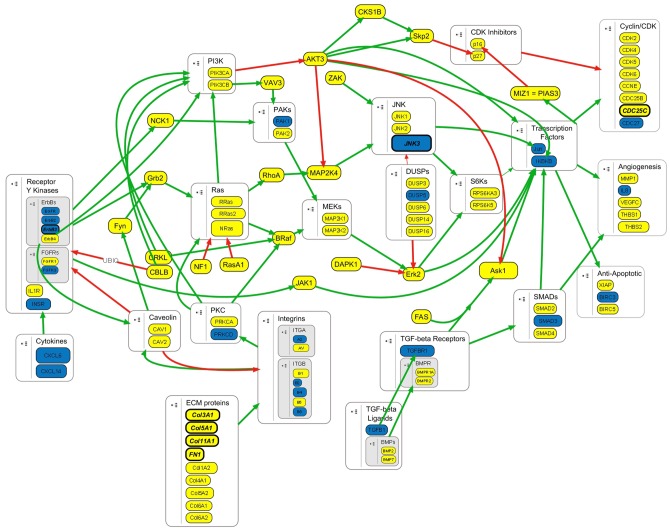
Altered expression in a network of Ras/MAPK associated signaling pathway genes in TCC progression. The stimulatory and inhibitory interactions of gene products differentially expressed in T1–T4 versus Ta tumors were mapped based on KEGG pathway maps. Node color denotes relative expression: Yellow = Increased expression in muscle-invasive tumors; Blue = Decreased expression in muscle-invasive tumors. Green and red arrows represent stimulatory and inhibitory interactions, respectively. The names of genes with supported associations with muscle-invasive tumors are highlighted in bold italics.

KEGG pathways and other related ontologies and annotations are highly redundant, with individual genes associated with many multiple limited and interconnecting pathways. For this and other reasons, pathway enrichment analysis alone often does not adequately represent the global scope and relationships within the data. To better understand the relationships of these genes and how they may function in bladder cancer progression, we built an integrated pathway network model based on the KEGG maps of selected signaling pathways represented in the data (Table 2; Table S3). These results reveal that gene expression differences between Ta and T1 stage tumors largely occur within a signaling network related to Ras/MAPK signaling pathways, with increased expression of a majority of these genes (Figure 2).

### Muscle-invasive TCC is associated with specific expression changes in ECM and signaling proteins

**Figure 3:**
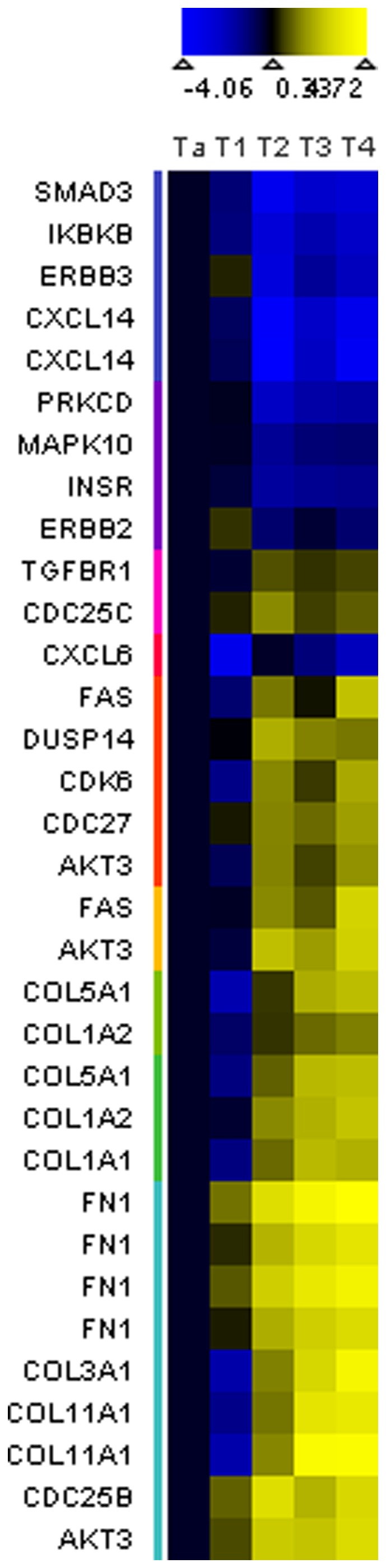
Expression heatmap of candidate muscle-invasive genes. Yellow and blue indicate increased and decreased expression in candidate muscle-invasive genes, respectively, relative to the average expression in Ta tumors.

We further hypothesized that the expression of a subset of the genes within this signaling network model is specifically altered in muscle-invasive versus superficial tumors. Returning to the complete dataset, we selected genes that are expressed at similar levels within a 2 fold difference between Ta and T1 tumors, and are changed greater than 2 fold in T2 stage tumors with Student's t-test p<0.05 (Figure 1). After performing expression clustering analysis and arbitrarily removing clusters with minimal expression changes, as above, 496 total gene probes were selected for pathway enrichment analysis using WebGestalt. As before, these results show that the selected genes are associated with many signal transduction and cancer-related pathways that involve 23 of the selected genes (Table 3, Table S4, Figure 3). These genes were not focused in any single pathway, but instead were distributed across the network of pathways (Figure 2). Visualizing gene expression data relative to Ta tumors in a heatmap, the expression changes of selected genes are consistent in T2, T3 and T4 tumors (Figure 3). These results suggest that the differences of selected gene expression are distinct fundamental molecular characteristics of muscle-invasive tumors.

**Table 3:** KEGG pathway enrichment of genes differentially expressed in T2 versus Ta and T1 stage bladder tumors.

KEGG Pathway	Number of Pathway Genes in Dataset	Enrichment Factor “R”	Fisher's Exact, Adjusted P-value
**All Changes, Top 10**			
Focal adhesion*	14	8.4	1.44E-07
Tight junction	11	9.92	7.39E-07
Pancreatic cancer	8	13.43	4.23E-06
Androgen and estrogen metabolism	6	16.12	3.96E-05
Adherens junction	7	10.99	6.08E-05
ECM-receptor interaction	7	10.08	9.07E-05
Retinol metabolism	6	11.33	2.00E-04
MAPK signaling pathway*	11	4.94	2.00E-04
Ascorbate and aldarate metabolism	4	19.34	5.00E-04
Drug metabolism – other enzymes	5	11.85	5.00E-04
**Selected Pathways**			
Pathways in cancer*	10	3.6	1.90E-03
Cell cycle*	6	5.67	2.30E-03
**Genes Up, Top 10**			
Focal adhesion*	11	12.79	5.49E-08
ECM-receptor interaction	7	19.48	1.66E-06
Cell cycle*	5	9.13	2.10E-03
MAPK signaling pathway*	7	6.08	2.10E-03
Tight junction	5	8.72	2.50E-03
Regulation of actin cytoskeleton	6	6.49	2.70E-03
Progesterone-mediated oocyte maturation	4	10.87	2.90E-03
Valine, leucine and isoleucine degradation	3	15.93	4.60E-03
Sulfur metabolism	2	35.95	6.40E-03
Vascular smooth muscle contraction	4	8.13	6.60E-03
**Genes Down, Top 10**			
Retinol metabolism	6	12024	1.09E-05
Androgen and estrogen metabolism	5	27.68	1.63E-05
Adherens junction	6	19.41	1.63E-05
Drug metabolism – other enzymes	5	24.42	2.29E-05
Ascorbate and aldarate metabolism	4	39.86	2.73E-05
Pentose and glucuronate interconversions	4	36.91	3.14E-05
Pancreatic cancer	5	17.3	5.67E-05
Drug metabolism – cytochrome P450	5	17.3	5.67E-05
Metabolism of xenobiotics by cytochrome P450	5	17.79	5.67E-05

(*) indicates KEGG pathways that involve mechanisms of signal transduction. P value represents the results of Fisher's Exact tests reported by WebGestalt.

**Figure 4:**
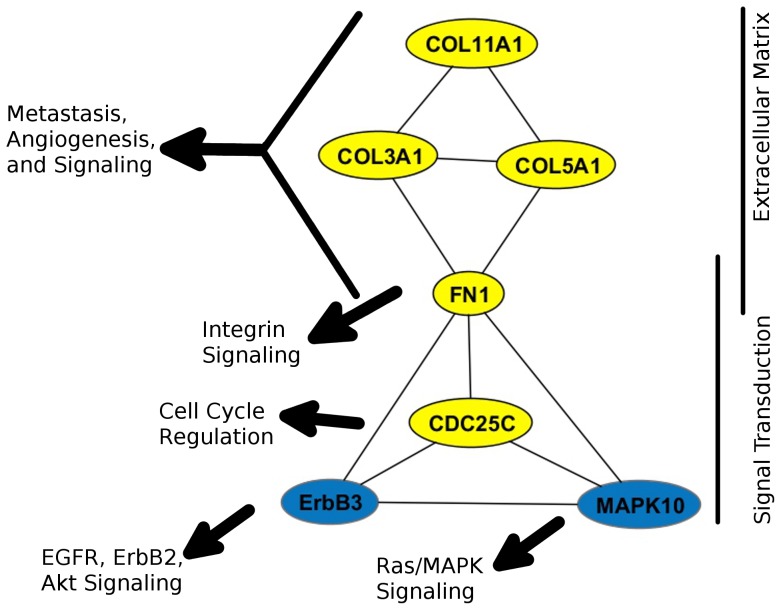
Bibliomic associations network in selected gene expression of muscle-invasive versus superficial tumors. Node color represents change in expression. Edges represent functional associations identified in the literature by text-mining programs PubGene and Chilibot. Selected genes separate into two functional groups of extracellular matrix and signal transduction genes. Black arrows represent generalized groups of outside genes with functional relationships to selected genes in the literature.

We then determined whether the relative expression of the selected genes could be observed in outside microarray datasets of superficial and muscle-invasive bladder tumors. Using Oncomine [24], we identified archived datasets containing expression data for each of the 23 selected candidate genes. The median gene expression of non-invasive and invasive tumors in each dataset was recorded, and the average change in expression of each gene was calculated across all datasets (Table 4). The Mann-Whitney Rank-Sum test was performed to determine the significance of the changes in median gene expression across datasets. These results show that expression of extracellular matrix genes COL3A1, COL5A1, COL11A1 and FN1 are significantly increased in muscle-invasive bladder tumors relative to Ta/T1 (p<0.05), while changes in CDC25C, MAPK10 and ErbB3 expression approached significance (p≤0.08) (Table 4). These final selected genes represent a sub-network of consistently-observed differences in gene expression of muscle-invasive and non-invasive bladder tumors that are associated with the activity of Ras/MAPK, PI3K and other signaling pathways (Figure 4).

**Table 4:** Average normalized median expression of selected genes differentially expressed in non-invasive and muscle-invasive TCC in at least 3 datasets within the Oncomine Collection.

	Gene	Average Change in Median (Fold Increase vs Superficial)	St.Dev.	Mann-Whitney Rank-Sum p =	References
**Validated**	COL3A1	3.99	2.29	0.028	[16]–[21]
	COL5A1	5.07	1.74	0.047	[15]–[17], [19], [20]
	COL11A1	3.37	2.30	0.025	[15]–[17], [19]–[21]
	FN1	5.45	2.25	0.028	[16], [17], [19]–[21]
	CDC25C	1.48	1.43	0.08	[15]–[17], [19]–[21]
	MAPK10	0.55	1.70	0.08	[15]–[17], [19]–[21]
	ERBB3	0.45	1.77	0.072	[16], [17], [19]–[21]
**Rejected**	AKT3	1.64	2.02	0.305	[15]–[17], [19]–[21]
	DUSP14	1.53	1.26	0.105	[16], [17], [19]–[21]
	FAS	1.43	1.49	0.236	[15]–[17], [19], [21]
	COL1A1	3.72	2.52	0.101	[16]–[20]
	COL1A2	2.26	2.73	0.288	[16]–[20]
	CDC25B	1.53	1.54	0.189	[15]–[21]
	CDK6	1.53	1.36	0.189	[15]–[17], [19]–[21]
	TGFBR1	1.24	1.41	0.443	[16], [17], [19], [20]
	CDC27	1.17	1.42	0.222	[15]–[17], [19]–[21]
	IKBKB	0.76	1.25	0.148	[15], [16], [19]–[21]
	ERBB2	0.71	1.65	0.417	[15]–[17], [19]–[21]
	SMAD3	0.69	1.76	0.202	[15]–[19], [21]
	PRKCD	0.58	1.65	0.202	[15]–[17], [19], [20]
	CXCL6	2.96	1.99	0.156	[15], [16], [19]–[21]
	CXCL14	1.02	1.35	0.5	[16], [19], [20]
	INSR	1.05	1.36	0.115	[15]–[17], [19], [21]

Genes selected based on significance (p<0.05) or near-significance (p<0.10) as determined by one-sided Mann-Whitney Rank-Sum tests. “References” contain the citations of the studies from which data analyzed by Oncomine were derived.

While the consistent expression changes of the 7 selected genes are well supported by expression data from human clinical samples, the functional relationships of these genes were not immediate. With the expectation that functional relationships of selected genes would be reflected in the literature, we used the text-mining programs Chilibot and PubGeneto identify and visualize these relationships [10], [11]. The results show that the genes can be placed in two functional groups of extracellular matrix proteins and signal transduction proteins (Figure 4). Outside genes that are functionally related to the selected genes showed little overlap between associated groups, suggesting that seven selected genes are critical nodes of regulation that coordinate the activities of outside gene pathway networks (Figure 4, and Data Not Shown). Further, the relationships of selected genes characteristics associated with advanced metastatic cancer were assessed using Chilibot. The CDC25C, ErbB3 and FN1 are previously associated with bladder cancer: The activity of CDC25C promotes proliferation and is a target for developing cancer therapies, including bladder cancer [25]; Decreased ErbB3 expression in bladder cancer is associated with poor prognosis [26]; and FN1 is a potential urine biomarker for bladder cancer [27]. All of the selected genes are associated with cancer and metastasis (Table 5). Interestingly, MAPK10 and FN1 were related to angiogenesis, COL5A1 and COL11A1 were associated only with invasion, while COL3A1, CDC25C and ErbB3 are associated with both. In all, the selected genes are associated with terms in published reports describe characteristics of muscle-invasive and metastatic tumors, and are relevant to mechanisms regulating the progression of advanced stage bladder cancer.

**Table 5:** Literature-based associations of selected genes and characteristics associated with advanced or metastatic cancers, identified using Chilibot [11].

Gene	Bladder Cancer	Cancer	Metastasis	Angiogenesis	Invasion
**COL11A1**		*	*		*
**COL5A1**		*	*		*
**COL3A1**		*	*	*	*
**CDC25C**	*	*	*	*	*
**ErbB3**	*	*	*	*	*
**MAPK10**		*	*	*	
**FN1**	*	*	*	*	

* denotes identification of relationships between the terms and gene.

### Increased COL3A1 and COL5A1 protein expression in muscle-invasive tumors and metastatic T24 cells

**Figure 5:**
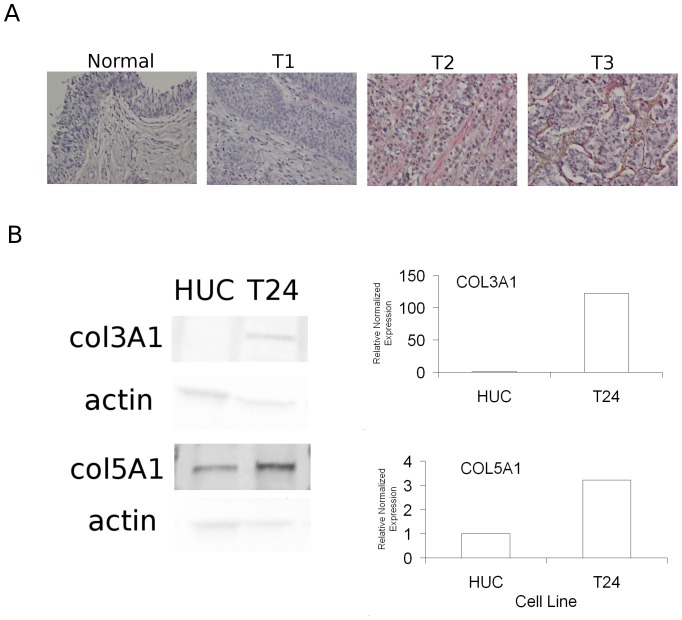
Elevated expression of COL3A1 and COL5A1 in muscle-invasive bladder tumors and metatstatic T24 cells. A. Immunohistochemistry of bladder tumors. DAB (brown) staining represents COL3A1 protein expression, while Warp Red staining represents COL5A1. Results are representative of five independent sample tissues per grade. B. Immunoblot analysis in immortalized non-cancer HUC cells and T24 bladder cancer cells. Expression of COL3A1 and COL5A1 was standardized to that of β-actin, and normalized to expression in HUC cells. These results are representative of 2 independent experiments.

Because the expression changes of COL3A1 and COL5A1 are well supported statistically, the novelty of these genes' association with muscle-invasive bladder cancer, and the availability of validated antibodies recognizing these proteins, we investigated whether their protein expression is increased in samples of muscle-invasive bladder tumors removed from patients. While the intensity and extent of staining results were variable, we found that COL3A1 and COL5A1 was detected in epithelial and stromal cells of T2 and T3 tumors, while little to no staining was detected in normal bladder tissue and T1 tumors (Figure 5A). Additionally, we found that T24 cells, which are metastatic in an *in vivo* xenograft model, show increased expression of these proteins compared to E6-immortalized HUCs derived from normal tissue (Figure 5B, Data Not Shown). These results validate the novel association of COL3A1 and COL5A1 expression with muscle-invasive bladder tumors.

## Discussion

The outcome of bladder cancer treatment is largely dependent upon the pathobiology of individual bladder tumors [2]. Previous microarray-based gene expression studies of various stage bladder cancers have recognized the distinct behaviors and genetic nature of superficial and muscle-invasive tumors, suggesting the possibility that the two are discrete pathobiological entities [4]. However, no biomarkers have proven able to predict progression to muscle-invasive disease, and the physiological mechanisms that drive bladder tumor progression and invasive behavior are not well understood.

We have analyzed a large collection of expression microarray-derived data to develop a functional picture of bladder cancer progression to muscle-invasive disease. We achieved this by using only publicly available microarray data and open-access computational resources. This avoids the need to generate custom tools to perform specific analyses to analyze large amounts of multi-dimensional data. Moreover, this strategy avoided the need to invest the time and resources to acquire appropriate clinical samples in large enough numbers to allow a meaningful analysis, as well as the time and resources to perform a large number of microarrays analyses. This is especially important given that a large amount of data already exists and is available to the biomedical research community. By re-purposing public data to address our specific interests, we have not only avoided significant expenses but we also help to increase the value drawn from existing data. This has resulted in a novel systems-level model of TCC progression and muscle-invasive disease that integrates gene expression data with functional information and relationships from previous experimental observations.

In the present study, we identified specific expression differences that occur with bladder cancer progression and muscle-invasive tumors. Our results show that progression from papillary Ta to more advanced stage tumors is associated with a general increase in expression of a network of genes involved in Ras/MAPK and associated signaling pathways (Figure 2). These pathways are closely associated with proliferation and are frequently disregulated in cancers, including bladder cancer. Interestingly, these pathways are downstream of the EGFR and ErbB2 family proteins whose expression and activity are related to bladder cancer progression, as well as ErbB3, FGFR3 and HRAS that are active in superficial tumors [4]. The increased expression of genes in Ras/MAPK and PI3K pathways may be related to the disregulation of p53, Rb and other tumor suppressors in muscle-invasive bladder tumors [3], [4]. The increased expression of components of this large pathway network may act to promote and enhance the effects of EGFR and ErbB2 activity, but the dependence of muscle-invasive tumors on the activities of these pathways in concert has not been investigated. Conversely, the relatively low expression of Ras/MAPK and PI3K pathways in superficial tumors may compensate for the elevated activity of ErbB3, FGFR3 and RAS isoforms, possibly a factor that limits the progression to muscle-invasive disease. These and other issues will be further addressed through the course of developing a systems-level understanding of bladder cancer progression.

We also used a similar approach to identify gene expression changes specifically associated with muscle-invasive tumors (Figure 2, Figure 3, Figure 4). We found that these initially selected genes occur sporadically across the initial network rather than focused on a particular pathway within the network, but the expression of these genes is consistent and distinct in superficial and muscle-invasive tumor samples (Figure 3). We then tested whether the expression differences of selected genes in our initial dataset were consistent with data from outside studies by using Oncomine to measure the median expression of each genes in 3 to 8 independent studies that include superficial and muscle-invasive bladder tumors, selecting a final set of 7 genes (Figure 4) [16]–[22]. Taking into account the number of samples within each study, these results ultimately represent the gene expression data of hundreds of individual tumors, supporting the statistical validity of our observations.

The selected 7 genes can be separated into extracellular matrix proteins (COL3A1, COL5A1, COL11A1, FN1) and kinase signaling proteins (CDC25C, MAPK10, ErbB3). While the variation in expression data does not support the use of any of these genes as biomarkers individually (Data Not Shown), their expression may support a more specific means to identify muscle-invasive bladder tumors in the future. FN1 itself is a potential urine biomarker for bladder cancer detection [27]. While well supported, their functional relationships and relevance to muscle-invasive bladder cancer were not immediately apparent. To aid in our interpretation, the data were analyzed using text-mining programs that identify instances where gene names occur in potentially interactive contexts within abstracts and full texts of published reports in PubMed [10], [11]. We found that these genes have been associated with cancer, bladder cancer, metastasis, angiogenesis, and invasion, suggesting that their involvement in cancer progression and/or muscle-invasive behaviors have been previously observed in various systems (Table 5).

**Figure 6:**
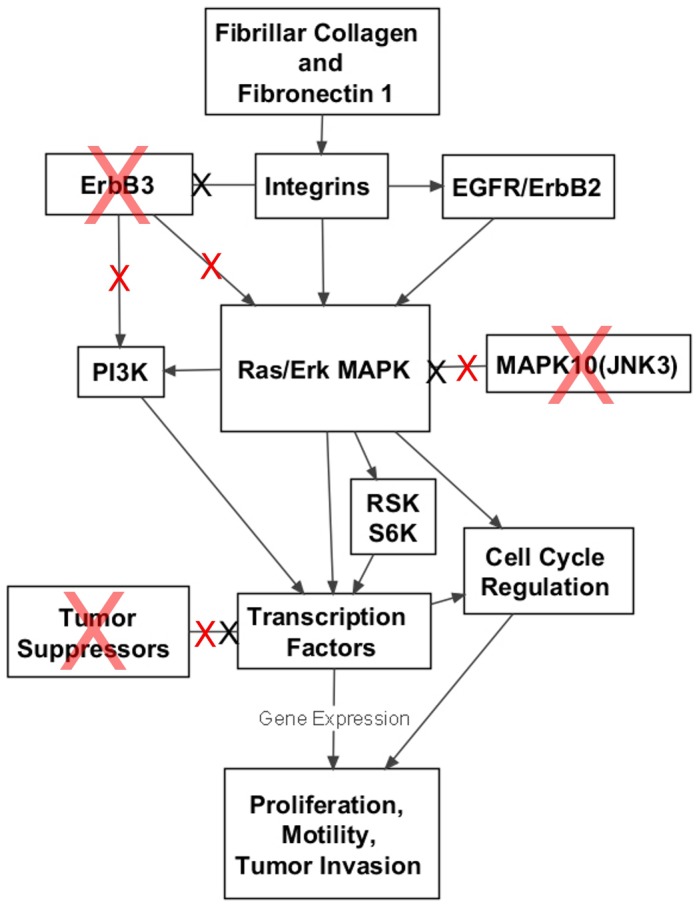
Model of muscle-invasive bladder cancer based on expression data and published observations. Arrows represent positive interactions. Black “X”s on arrows represent antagonistic interactions. Red “X”s represent disrupted or decreased activity.

The reported functions and interactions of the selected genes suggest a novel model of muscle-invasive disease in bladder cancer (Figure 6). COL3A1, COL5A1 and COL11A1 are fibrillar collagens, which act as “tracks” for metastatic invasion of breast tumors into secondary organs [28]. Formation of these fibrils is initiated by interactions between COL5A1 and COL11A1 proteins [29]. Tumor-secreted proteases diffuse along collagen fibrils and modify existing matrix to allow tumor cells a path of least-resistance along fibrils [30]. Collagens and FN1 are among many ligands for integrins, a class of surface receptor proteins that are functional heterodimers of 8 α and 18 β subunits that each bind specific ligands [31]. COL3A1 and COL5A1 are functionally related in connective tissue disorders [32], and expression of different collagens are associated with various cancers. FN1 “decorates” collagen fibrils and affects integrin binding specificity [31], [33]. Integrin signaling stimulates RAS/MAPK signaling and modifies the activity of receptor tyrosine kinases, including EGFR/ErbB proteins, which are also upstream of Ras/MAPK [31], [34]. Interestingly, FN1 has been reported to preferentially induce the activity of EGFR, ErbB2 and ErbB4 but not ErbB3 activity [34]. Our identification of decreased ErbB3 expression in muscle-invasive tumors is well supported by previous studies [26]. Formation of ErbB3/ErbB2 is kinetically preferred among ErbB family receptors, and ErbB3 is the only ErbB receptor that directly associates with and activates the PI3K signaling pathway [35]. In all, this evidence suggests a “class-switch” from ErbB3 to EGFR/ErbB2 mediated ErbB signaling, mediated in part by collagen and fibronectin-stimulated integrin signaling, is a key mechanism promoting muscle-invasion of bladder tumors. This may affect trafficking of active receptor proteins and impact the intensity and duration of downstream signaling [36], as well as alter the pathways that stimulate PI3K signaling.

Importantly, the effects of these gene expression differences in muscle-invasive tumors are likely augmented by increased expression of genes in mitogenic pathways that we find associated with general bladder cancer progression in the absence of activation mutations in RAS and PI3K pathways that are prevalent in superficial tumors. The ErbB and collagen-activated integrin proteins are upstream activators of Ras/MAPK signaling, which is associated with proliferative and tumorigenic activity and which our above expression data shows is generally increased in muscle-invasive bladder cancer (Figure 2). Changes in how Ras/MAPK and PI3K signaling are regulated by upstream receptors may fundamentally alter the nature of the responses to those signaling pathways. The MAPK family protein MAPK10/JNK3 is generally involved in stress signaling and acts counter to Erk MAPK and PI3K signaling [37]. The decreased expression of antagonistic signaling proteins such as MAPK10 would further promote Ras/Erk MAPK signaling activity. Ultimately these activities modulate the expression and activities of proteins that affect proliferative, pro-metastatic and invasive behavior, including the CDC25C phosphatase which stimulates cyclin/CDK activity and mitosis. Metastasis and progression are further promoted by the disruption of tumor suppressors in muscle-invasive bladder cancer, including p53, Rb, myc and others that would act to limit proliferative and metastatic behavior [2]–[4]. As well as providing a basis for future systems-level studies of TCC, this model will inform efforts to identify and develop the therapeutic targets and predictive biomarkers that will guide the clinical treatment of muscle-invasive bladder cancer.

In summary, we have analyzed public expression microarray data of bladder tumors across stages, identifying the increased expression of the proliferative and pro-survival Ras/MAPK and PI3K pathways as a potential regulatory hallmark of tumor progression, while related genes, including fibrillar collagens are associated specifically with muscle-invasive bladder cancer. Translation of this information into clinical treatment may potentially improve the identification, treatment and outcome of bladder cancer in patients as well as in the treatment of other cancers.

## Supporting Information

Table S1 **Prefered Reporting Items for Systematic Reviews and Meta-Analyses (PRISMA) Guidelines Checklist.**
(PDF)

Table S2 **Prefered Reporting Items for Systematic Reviews and Meta-Analyses (PRISMA) Guidelines Checklist (Continued).**
(PDF)

Table S3 **Genes differentially expressed, both increased and decreased, in T1–T4 staged bladder tumors versus Ta-stage tumors and associated with KEGG pathways, including Top 10 pathways listed in** **Table 2** **.** Pathways selected based on Fisher's Exact Scores ≤0.01.
(DOC)

Table S4 **Genes differentially expressed, both increased and decreased, in Ta/T1-staged bladder tumors vs T2 tumors, and associated with KEGG pathways, including Top 10 pathways listen in** **Table 3** **.** Pathways selected based on Fisher's Exact Scores ≤0.01.
(DOC)
