# Supplementary material for: Expression Microarray Meta-Analysis Identifies Genes Associated with Ras/MAPK and Related Pathways in Progression of Muscle-Invasive Bladder Transition Cell Carcinoma
Source: PLoS One. 2013 Feb 1;8(2):e55414. doi: 10.1371/journal.pone.0055414 (PMC3562183; doi:10.1371/journal.pone.0055414)
Supplement: Table S3 — Genes differentially expressed, both increased and decreased, in T1–T4 staged bladder tumors versus Ta-stage tumors and associated with KEGG pathways, including Top 10 pathways listed in Table 2 . Pathways selected based on Fisher's Exact Scores ≤0.01. (DOC) [file pone.0055414.s003.doc]

| **Pathway** | **Gene Symbol** | **Gene Name** |
| --- | --- | --- |
| **KEGG pathway----Metabolic pathways----01100** | GAA | glucosidase, alpha; acid |
|  | ACACB | acetyl-Coenzyme A carboxylase beta |
|  | AMY1A | amylase, alpha 1A (salivary) |
|  | NDUFB6 | NADH dehydrogenase (ubiquinone) 1 beta subcomplex, 6, 17kDa |
|  | PRDX6 | peroxiredoxin 6 |
|  | PRDX6 | peroxiredoxin 6 |
|  | HEXB | hexosaminidase B (beta polypeptide) |
|  | CSAD | cysteine sulfinic acid decarboxylase |
|  | GLUD1 | glutamate dehydrogenase 1 |
|  | OAT | ornithine aminotransferase (gyrate atrophy) |
|  | MTMR2 | myotubularin related protein 2 |
|  | MTMR2 | myotubularin related protein 2 |
|  | MTMR2 | myotubularin related protein 2 |
|  | POLR3K | polymerase (RNA) III (DNA directed) polypeptide K, 12.3 kDa |
|  | TYMP | thymidine phosphorylase |
|  | PNLIPRP2 | pancreatic lipase-related protein 2 |
|  | ALDH1A3 | aldehyde dehydrogenase 1 family, member A3 |
|  | MAN2A1 | mannosidase, alpha, class 2A, member 1 |
|  | MAN2A1 | mannosidase, alpha, class 2A, member 1 |
|  | NDUFB8 | NADH dehydrogenase (ubiquinone) 1 beta subcomplex, 8, 19kDa |
|  | UPRT | uracil phosphoribosyltransferase (FUR1) homolog (S. cerevisiae) |
|  | ME1 | malic enzyme 1, NADP(+)-dependent, cytosolic |
|  | COMT | catechol-O-methyltransferase |
|  | OXSM | 3-oxoacyl-ACP synthase, mitochondrial |
|  | DPYD | dihydropyrimidine dehydrogenase |
|  | DLD | dihydrolipoamide dehydrogenase |
|  | ZNRD1 | zinc ribbon domain containing 1 |
|  | ZNRD1 | zinc ribbon domain containing 1 |
|  | DNMT1 | DNA (cytosine-5-)-methyltransferase 1 |
|  | ACACA | acetyl-Coenzyme A carboxylase alpha |
|  | PDHA1 | pyruvate dehydrogenase (lipoamide) alpha 1 |
|  | LIPF | lipase, gastric |
|  | DTYMK | deoxythymidylate kinase (thymidylate kinase) |
|  | PIGK | phosphatidylinositol glycan anchor biosynthesis, class K |
|  | PIGK | phosphatidylinositol glycan anchor biosynthesis, class K |
|  | GALNT5 | UDP-N-acetyl-alpha-D-galactosamine:polypeptide N-acetylgalactosaminyltransferase 5 (GalNAc-T5) |
|  | FH | fumarate hydratase |
|  | HSD17B2 | hydroxysteroid (17-beta) dehydrogenase 2 |
|  | PRIM1 | primase, DNA, polypeptide 1 (49kDa) |
|  | PYCR1 | pyrroline-5-carboxylate reductase 1 |
|  | KDSR | 3-ketodihydrosphingosine reductase |
|  | KDSR | 3-ketodihydrosphingosine reductase |
|  | POLR2C | polymerase (RNA) II (DNA directed) polypeptide C, 33kDa |
|  | STT3A | STT3, subunit of the oligosaccharyltransferase complex, homolog A (S. cerevisiae) |
|  | FBP1 | fructose-1,6-bisphosphatase 1 |
|  | CYP3A5 | cytochrome P450, family 3, subfamily A, polypeptide 5 |
|  | OGT | O-linked N-acetylglucosamine (GlcNAc) transferase (UDP-N-acetylglucosamine:polypeptide-N-acetylglucosaminyl transferase) |
|  | OGT | O-linked N-acetylglucosamine (GlcNAc) transferase (UDP-N-acetylglucosamine:polypeptide-N-acetylglucosaminyl transferase) |
|  | ALAS1 | aminolevulinate, delta-, synthase 1 |
|  | BDH2 | 3-hydroxybutyrate dehydrogenase, type 2 |
|  | TYMS | thymidylate synthetase |
|  | TYMS | thymidylate synthetase |
|  | UQCRH | ubiquinol-cytochrome c reductase hinge protein |
|  | GNPDA2 | glucosamine-6-phosphate deaminase 2 |
|  | CHPF2 | chondroitin polymerizing factor 2 |
|  | PFKP | phosphofructokinase, platelet |
|  | B4GALT2 | UDP-Gal:betaGlcNAc beta 1,4- galactosyltransferase, polypeptide 2 |
|  | DLAT | dihydrolipoamide S-acetyltransferase |
|  | EHHADH | enoyl-Coenzyme A, hydratase/3-hydroxyacyl Coenzyme A dehydrogenase |
|  | MUT | methylmalonyl Coenzyme A mutase |
|  | ALDH6A1 | aldehyde dehydrogenase 6 family, member A1 |
|  | POLR2K | polymerase (RNA) II (DNA directed) polypeptide K, 7.0kDa |
|  | POLR2K | polymerase (RNA) II (DNA directed) polypeptide K, 7.0kDa |
|  | DGKQ | diacylglycerol kinase, theta 110kDa |
|  | DHCR24 | 24-dehydrocholesterol reductase |
|  | RRM1 | ribonucleotide reductase M1 |
|  | RRM1 | ribonucleotide reductase M1 |
|  | NDUFA4 | NADH dehydrogenase (ubiquinone) 1 alpha subcomplex, 4, 9kDa |
|  | PLA2G12A | phospholipase A2, group XIIA |
|  | DPM3 | dolichyl-phosphate mannosyltransferase polypeptide 3 |
|  | ISYNA1 | inositol-3-phosphate synthase 1 |
|  | NDUFS7 | NADH dehydrogenase (ubiquinone) Fe-S protein 7, 20kDa (NADH-coenzyme Q reductase) |
|  | NDUFS4 | NADH dehydrogenase (ubiquinone) Fe-S protein 4, 18kDa (NADH-coenzyme Q reductase) |
|  | GNS | glucosamine (N-acetyl)-6-sulfatase |
|  | RPN1 | ribophorin I |
|  | SC4MOL | sterol-C4-methyl oxidase-like |
|  | INPP5A | inositol polyphosphate-5-phosphatase, 40kDa |
|  | DAD1 | defender against cell death 1 |
|  | ATP5H | ATP synthase, H+ transporting, mitochondrial F0 complex, subunit d |
|  | CTPS | CTP synthase |
|  | CKB | creatine kinase, brain |
|  | CYC1 | cytochrome c-1 |
|  | COQ3 | coenzyme Q3 homolog, methyltransferase (S. cerevisiae) |
|  | AGL | amylo-1, 6-glucosidase, 4-alpha-glucanotransferase |
|  | PRPS1 | phosphoribosyl pyrophosphate synthetase 1 |
|  | IDUA | iduronidase, alpha-L- |
|  | SMS | spermine synthase |
|  | HMGCR | 3-hydroxy-3-methylglutaryl-Coenzyme A reductase |
|  | HMGCR | 3-hydroxy-3-methylglutaryl-Coenzyme A reductase |
|  | FDFT1 | farnesyl-diphosphate farnesyltransferase 1 |
|  | FDFT1 | farnesyl-diphosphate farnesyltransferase 1 |
|  | GFPT1 | glutamine-fructose-6-phosphate transaminase 1 |
|  | GFPT1 | glutamine-fructose-6-phosphate transaminase 1 |
|  | ADH7 | alcohol dehydrogenase 7 (class IV), mu or sigma polypeptide |
|  | B3GNT7 | UDP-GlcNAc:betaGal beta-1,3-N-acetylglucosaminyltransferase 7 |
|  | B3GNT7 | UDP-GlcNAc:betaGal beta-1,3-N-acetylglucosaminyltransferase 7 |
|  | NP | nucleoside phosphorylase |
|  | PRIM2 | primase, DNA, polypeptide 2 (58kDa) |
|  | PRIM2 | primase, DNA, polypeptide 2 (58kDa) |
|  | PRIM2 | primase, DNA, polypeptide 2 (58kDa) |
|  | PIGT | phosphatidylinositol glycan anchor biosynthesis, class T |
|  | PIGT | phosphatidylinositol glycan anchor biosynthesis, class T |
|  | ALG6 | asparagine-linked glycosylation 6, alpha-1,3-glucosyltransferase homolog (S. cerevisiae) |
|  | POLA1 | polymerase (DNA directed), alpha 1, catalytic subunit |
|  | MAN1B1 | mannosidase, alpha, class 1B, member 1 |
|  | MAN1B1 | mannosidase, alpha, class 1B, member 1 |
|  | RDH10 | retinol dehydrogenase 10 (all-trans) |
|  | FASN | fatty acid synthase |
|  | DCTD | dCMP deaminase |
|  | SPTLC2 | serine palmitoyltransferase, long chain base subunit 2 |
|  | PFKM | phosphofructokinase, muscle |
|  | PLA2G2A | phospholipase A2, group IIA (platelets, synovial fluid) |
|  | CAT | catalase |
|  | EPRS | glutamyl-prolyl-tRNA synthetase |
|  | EPRS | glutamyl-prolyl-tRNA synthetase |
|  | TUSC3 | tumor suppressor candidate 3 |
|  | TUSC3 | tumor suppressor candidate 3 |
|  | NT5C2 | 5'-nucleotidase, cytosolic II |
|  | MTHFD2 | methylenetetrahydrofolate dehydrogenase (NADP+ dependent) 2, methenyltetrahydrofolate cyclohydrolase |
|  | PGP | phosphoglycolate phosphatase |
|  | NME7 | non-metastatic cells 7, protein expressed in (nucleoside-diphosphate kinase) |
|  | ATP5C1 | ATP synthase, H+ transporting, mitochondrial F1 complex, gamma polypeptide 1 |
|  | ACLY | ATP citrate lyase |
|  | SQLE | squalene epoxidase |
|  | SQLE | squalene epoxidase |
|  | SQLE | squalene epoxidase |
|  | SGMS2 | sphingomyelin synthase 2 |
|  | UMPS | uridine monophosphate synthetase |
|  | UMPS | uridine monophosphate synthetase |
|  | MDH1 | malate dehydrogenase 1, NAD (soluble) |
|  | ALG2 | asparagine-linked glycosylation 2, alpha-1,3-mannosyltransferase homolog (S. cerevisiae) |
|  | ACADVL | acyl-Coenzyme A dehydrogenase, very long chain |
|  | DHCR7 | 7-dehydrocholesterol reductase |
|  | DHCR7 | 7-dehydrocholesterol reductase |
|  | GALE | UDP-galactose-4-epimerase |
|  | PAPSS2 | 3'-phosphoadenosine 5'-phosphosulfate synthase 2 |
|  | PAPSS2 | 3'-phosphoadenosine 5'-phosphosulfate synthase 2 |
|  | ATP6V1A | ATPase, H+ transporting, lysosomal 70kDa, V1 subunit A |
|  | ATP6V1A | ATPase, H+ transporting, lysosomal 70kDa, V1 subunit A |
|  | PAFAH1B1 | platelet-activating factor acetylhydrolase, isoform Ib, subunit 1 (45kDa) |
|  | GALNT12 | UDP-N-acetyl-alpha-D-galactosamine:polypeptide N-acetylgalactosaminyltransferase 12 (GalNAc-T12) |
|  | GALNT12 | UDP-N-acetyl-alpha-D-galactosamine:polypeptide N-acetylgalactosaminyltransferase 12 (GalNAc-T12) |
|  | NDUFB5 | NADH dehydrogenase (ubiquinone) 1 beta subcomplex, 5, 16kDa |
|  | ETNK1 | ethanolamine kinase 1 |
|  | ETNK1 | ethanolamine kinase 1 |
|  | ETNK1 | ethanolamine kinase 1 |
|  | ETNK1 | ethanolamine kinase 1 |
|  | NDUFAB1 | NADH dehydrogenase (ubiquinone) 1, alpha/beta subcomplex, 1, 8kDa |
|  | BCAT1 | branched chain aminotransferase 1, cytosolic |
|  | BCAT1 | branched chain aminotransferase 1, cytosolic |
|  | hCG_1776980 | hCG1776980 |
|  | LCLAT1 | lysocardiolipin acyltransferase 1 |
|  | NDUFB3 | NADH dehydrogenase (ubiquinone) 1 beta subcomplex, 3, 12kDa |
|  | FLAD1 | FAD1 flavin adenine dinucleotide synthetase homolog (S. cerevisiae) |
|  | ALOX5 | arachidonate 5-lipoxygenase |
|  | ALOX5 | arachidonate 5-lipoxygenase |
|  | ALOX5 | arachidonate 5-lipoxygenase |
|  | PPAP2A | phosphatidic acid phosphatase type 2A |
|  | PPAP2A | phosphatidic acid phosphatase type 2A |
|  | ADI1 | acireductone dioxygenase 1 |
|  | DUT | deoxyuridine triphosphatase |
|  | ALDOC | aldolase C, fructose-bisphosphate |
|  | FUT3 | fucosyltransferase 3 (galactoside 3(4)-L-fucosyltransferase, Lewis blood group) |
|  | ACSL4 | acyl-CoA synthetase long-chain family member 4 |
|  | CMPK1 | cytidine monophosphate (UMP-CMP) kinase 1, cytosolic |
|  | CMPK1 | cytidine monophosphate (UMP-CMP) kinase 1, cytosolic |
|  | MGLL | monoglyceride lipase |
|  | MGLL | monoglyceride lipase |
|  | GATM | glycine amidinotransferase (L-arginine:glycine amidinotransferase) |
|  | ATP6V1G1 | ATPase, H+ transporting, lysosomal 13kDa, V1 subunit G1 |
|  | PIGY | phosphatidylinositol glycan anchor biosynthesis, class Y |
|  | COX7C | cytochrome c oxidase subunit VIIc |
|  | AGPS | alkylglycerone phosphate synthase |
|  | AGPS | alkylglycerone phosphate synthase |
|  | AGPS | alkylglycerone phosphate synthase |
|  | IDH3A | isocitrate dehydrogenase 3 (NAD+) alpha |
|  | PANK1 | pantothenate kinase 1 |
|  | SAT1 | spermidine/spermine N1-acetyltransferase 1 |
|  | POLR2D | polymerase (RNA) II (DNA directed) polypeptide D |
|  | COX11 | COX11 homolog, cytochrome c oxidase assembly protein (yeast) |
|  | CEL | carboxyl ester lipase (bile salt-stimulated lipase) |
|  | CEL | carboxyl ester lipase (bile salt-stimulated lipase) |
|  | GALK2 | galactokinase 2 |
|  | MAT2A | methionine adenosyltransferase II, alpha |
|  | SDHD | succinate dehydrogenase complex, subunit D, integral membrane protein |
|  | ALDH3B1 | aldehyde dehydrogenase 3 family, member B1 |
|  | ALDH3B1 | aldehyde dehydrogenase 3 family, member B1 |
|  | GRHPR | glyoxylate reductase/hydroxypyruvate reductase |
|  | GRHPR | glyoxylate reductase/hydroxypyruvate reductase |
|  | GRHPR | glyoxylate reductase/hydroxypyruvate reductase |
|  | ACAT2 | acetyl-Coenzyme A acetyltransferase 2 |
|  | ACAT2 | acetyl-Coenzyme A acetyltransferase 2 |
|  | ACAT2 | acetyl-Coenzyme A acetyltransferase 2 |
|  | DDOST | dolichyl-diphosphooligosaccharide-protein glycosyltransferase |
|  | DDOST | dolichyl-diphosphooligosaccharide-protein glycosyltransferase |
|  | NME1 | non-metastatic cells 1, protein (NM23A) expressed in |
|  | DCXR | dicarbonyl/L-xylulose reductase |
|  | PEMT | phosphatidylethanolamine N-methyltransferase |
|  | INPP4B | inositol polyphosphate-4-phosphatase, type II, 105kDa |
|  | PDXK | pyridoxal (pyridoxine, vitamin B6) kinase |
|  | PDXK | pyridoxal (pyridoxine, vitamin B6) kinase |
|  | B4GALT3 | UDP-Gal:betaGlcNAc beta 1,4- galactosyltransferase, polypeptide 3 |
|  | ALDOB | aldolase B, fructose-bisphosphate |
|  | NNMT | nicotinamide N-methyltransferase |
|  | NNMT | nicotinamide N-methyltransferase |
|  | PIGW | phosphatidylinositol glycan anchor biosynthesis, class W |
|  | BCKDHA | branched chain keto acid dehydrogenase E1, alpha polypeptide |
|  | SDHC | succinate dehydrogenase complex, subunit C, integral membrane protein, 15kDa |
|  | SDHC | succinate dehydrogenase complex, subunit C, integral membrane protein, 15kDa |
|  | ME3 | malic enzyme 3, NADP(+)-dependent, mitochondrial |
|  | MAN1A2 | mannosidase, alpha, class 1A, member 2 |
|  | SGMS1 | sphingomyelin synthase 1 |
|  | TKT | transketolase |
|  | AGPAT3 | 1-acylglycerol-3-phosphate O-acyltransferase 3 |
|  | INPP1 | inositol polyphosphate-1-phosphatase |
|  | PMVK | phosphomevalonate kinase |
|  | ADA | adenosine deaminase |
|  | PIGF | phosphatidylinositol glycan anchor biosynthesis, class F |
|  | PIGF | phosphatidylinositol glycan anchor biosynthesis, class F |
|  | ALDH3B2 | aldehyde dehydrogenase 3 family, member B2 |
|  | EBP | emopamil binding protein (sterol isomerase) |
|  | SGPL1 | sphingosine-1-phosphate lyase 1 |
|  | SGPL1 | sphingosine-1-phosphate lyase 1 |
|  | MGAT1 | mannosyl (alpha-1,3-)-glycoprotein beta-1,2-N-acetylglucosaminyltransferase |
|  | NDUFB4 | NADH dehydrogenase (ubiquinone) 1 beta subcomplex, 4, 15kDa |
|  | ALG13 | asparagine-linked glycosylation 13 homolog (S. cerevisiae) |
|  | ALG13 | asparagine-linked glycosylation 13 homolog (S. cerevisiae) |
|  | PAICS | phosphoribosylaminoimidazole carboxylase, phosphoribosylaminoimidazole succinocarboxamide synthetase |
|  | PAICS | phosphoribosylaminoimidazole carboxylase, phosphoribosylaminoimidazole succinocarboxamide synthetase |
|  | MGAT4A | mannosyl (alpha-1,3-)-glycoprotein beta-1,4-N-acetylglucosaminyltransferase, isozyme A |
|  | PIGP | phosphatidylinositol glycan anchor biosynthesis, class P |
|  | PTGS1 | prostaglandin-endoperoxide synthase 1 (prostaglandin G/H synthase and cyclooxygenase) |
|  | COX7B | cytochrome c oxidase subunit VIIb |
|  | IMPA2 | inositol(myo)-1(or 4)-monophosphatase 2 |
|  | LDHB | lactate dehydrogenase B |
|  | PCCB | propionyl Coenzyme A carboxylase, beta polypeptide |
|  | TK1 | thymidine kinase 1, soluble |
|  | TK1 | thymidine kinase 1, soluble |
|  | ALDH2 | aldehyde dehydrogenase 2 family (mitochondrial) |
|  | B3GNT2 | UDP-GlcNAc:betaGal beta-1,3-N-acetylglucosaminyltransferase 2 |
|  | PSAT1 | phosphoserine aminotransferase 1 |
|  | C1GALT1C1 | C1GALT1-specific chaperone 1 |
|  | ADSL | adenylosuccinate lyase |
|  | ADSL | adenylosuccinate lyase |
|  | AK3L1 | adenylate kinase 3-like 1 |
|  | AK3L1 | adenylate kinase 3-like 1 |
|  | POLE4 | polymerase (DNA-directed), epsilon 4 (p12 subunit) |
|  | POLE4 | polymerase (DNA-directed), epsilon 4 (p12 subunit) |
|  | CYP3A4 | cytochrome P450, family 3, subfamily A, polypeptide 4 |
|  | SGSH | N-sulfoglucosamine sulfohydrolase |
|  | HADH | hydroxyacyl-Coenzyme A dehydrogenase |
|  | POLE2 | polymerase (DNA directed), epsilon 2 (p59 subunit) |
|  | ATP5G3 | ATP synthase, H+ transporting, mitochondrial F0 complex, subunit C3 (subunit 9) |
|  | PGK1 | phosphoglycerate kinase 1 |
|  | PGK1 | phosphoglycerate kinase 1 |
|  | PGK1 | phosphoglycerate kinase 1 |
|  | PGK1 | phosphoglycerate kinase 1 |
|  | PTGDS | prostaglandin D2 synthase 21kDa (brain) |
|  | PTGDS | prostaglandin D2 synthase 21kDa (brain) |
|  | ACOX1 | acyl-Coenzyme A oxidase 1, palmitoyl |
|  | ACOX1 | acyl-Coenzyme A oxidase 1, palmitoyl |
|  | ACOX1 | acyl-Coenzyme A oxidase 1, palmitoyl |
|  | ALG14 | asparagine-linked glycosylation 14 homolog (S. cerevisiae) |
|  | DEGS1 | degenerative spermatocyte homolog 1, lipid desaturase (Drosophila) |
|  | DEGS1 | degenerative spermatocyte homolog 1, lipid desaturase (Drosophila) |
|  | GPAM | glycerol-3-phosphate acyltransferase, mitochondrial |
|  | GPAM | glycerol-3-phosphate acyltransferase, mitochondrial |
|  | CBR3 | carbonyl reductase 3 |
|  | ADK | adenosine kinase |
|  | ADK | adenosine kinase |
|  | PIGM | phosphatidylinositol glycan anchor biosynthesis, class M |
|  | COX15 | COX15 homolog, cytochrome c oxidase assembly protein (yeast) |
|  | ACSL1 | acyl-CoA synthetase long-chain family member 1 |
|  | ACSL1 | acyl-CoA synthetase long-chain family member 1 |
|  | POLR2F | polymerase (RNA) II (DNA directed) polypeptide F |
|  | UQCRB | ubiquinol-cytochrome c reductase binding protein |
|  | UCRC | ubiquinol-cytochrome c reductase complex (7.2 kD) |
|  | UGDH | UDP-glucose dehydrogenase |
|  | SI | sucrase-isomaltase (alpha-glucosidase) |
|  | SLC33A1 | solute carrier family 33 (acetyl-CoA transporter), member 1 |
|  | SLC33A1 | solute carrier family 33 (acetyl-CoA transporter), member 1 |
|  | ASS1 | argininosuccinate synthetase 1 |
|  | ABAT | 4-aminobutyrate aminotransferase |
|  | EXT1 | exostoses (multiple) 1 |
|  | HYAL2 | hyaluronoglucosaminidase 2 |
|  | BCAT2 | branched chain aminotransferase 2, mitochondrial |
|  | ALG10 | asparagine-linked glycosylation 10, alpha-1,2-glucosyltransferase homolog (S. pombe) |
|  | CYP51A1 | cytochrome P450, family 51, subfamily A, polypeptide 1 |
|  | AASDHPPT | aminoadipate-semialdehyde dehydrogenase-phosphopantetheinyl transferase |
|  | AASDHPPT | aminoadipate-semialdehyde dehydrogenase-phosphopantetheinyl transferase |
|  | ACY1 | aminoacylase 1 |
|  | ACAA2 | acetyl-Coenzyme A acyltransferase 2 |
|  | UQCRC2 | ubiquinol-cytochrome c reductase core protein II |
|  | DNMT3B | DNA (cytosine-5-)-methyltransferase 3 beta |
|  | AKR1B1 | aldo-keto reductase family 1, member B1 (aldose reductase) |
|  | POLE3 | polymerase (DNA directed), epsilon 3 (p17 subunit) |
|  | NDUFA6 | NADH dehydrogenase (ubiquinone) 1 alpha subcomplex, 6, 14kDa |
|  | ENO1 | enolase 1, (alpha) |
|  | ATP6V1E1 | ATPase, H+ transporting, lysosomal 31kDa, V1 subunit E1 |
|  | NDUFS2 | NADH dehydrogenase (ubiquinone) Fe-S protein 2, 49kDa (NADH-coenzyme Q reductase) |
|  | PIGX | phosphatidylinositol glycan anchor biosynthesis, class X |
|  | PIGX | phosphatidylinositol glycan anchor biosynthesis, class X |
|  | DPM1 | dolichyl-phosphate mannosyltransferase polypeptide 1, catalytic subunit |
|  | LAP3 | leucine aminopeptidase 3 |
|  | HADHA | hydroxyacyl-Coenzyme A dehydrogenase/3-ketoacyl-Coenzyme A thiolase/enoyl-Coenzyme A hydratase (trifunctional protein), alpha subunit |
|  | PPAP2C | phosphatidic acid phosphatase type 2C |
|  | RRM2 | ribonucleotide reductase M2 |
|  | ENPP7 | ectonucleotide pyrophosphatase/phosphodiesterase 7 |
|  | IDH1 | isocitrate dehydrogenase 1 (NADP+), soluble |
|  | IDH1 | isocitrate dehydrogenase 1 (NADP+), soluble |
|  | ODC1 | ornithine decarboxylase 1 |
|  | AMT | aminomethyltransferase |
|  | PDHB | pyruvate dehydrogenase (lipoamide) beta |
|  | ST3GAL5 | ST3 beta-galactoside alpha-2,3-sialyltransferase 5 |
|  | AKR1A1 | aldo-keto reductase family 1, member A1 (aldehyde reductase) |
|  | ATP5F1 | ATP synthase, H+ transporting, mitochondrial F0 complex, subunit B1 |
|  | GLS | glutaminase |
|  | GLS | glutaminase |
|  | GLS | glutaminase |
|  | ALG3 | asparagine-linked glycosylation 3, alpha-1,3- mannosyltransferase homolog (S. cerevisiae) |
|  | HPRT1 | hypoxanthine phosphoribosyltransferase 1 |
|  | LSS | lanosterol synthase (2,3-oxidosqualene-lanosterol cyclase) |
|  | AGPAT2 | 1-acylglycerol-3-phosphate O-acyltransferase 2 (lysophosphatidic acid acyltransferase, beta) |
|  | UGT1A9 | UDP glucuronosyltransferase 1 family, polypeptide A9 |
|  | KYNU | kynureninase (L-kynurenine hydrolase) |
|  | KYNU | kynureninase (L-kynurenine hydrolase) |
|  | KYNU | kynureninase (L-kynurenine hydrolase) |
|  | P4HA2 | prolyl 4-hydroxylase, alpha polypeptide II |
|  | NNT | nicotinamide nucleotide transhydrogenase |
|  | NNT | nicotinamide nucleotide transhydrogenase |
|  | PIK3C2B | phosphoinositide-3-kinase, class 2, beta polypeptide |
|  | GALNT10 | UDP-N-acetyl-alpha-D-galactosamine:polypeptide N-acetylgalactosaminyltransferase 10 (GalNAc-T10) |
|  | GALNT10 | UDP-N-acetyl-alpha-D-galactosamine:polypeptide N-acetylgalactosaminyltransferase 10 (GalNAc-T10) |
|  | NDUFA8 | NADH dehydrogenase (ubiquinone) 1 alpha subcomplex, 8, 19kDa |
|  | CHPT1 | choline phosphotransferase 1 |
|  | ATP6V0E1 | ATPase, H+ transporting, lysosomal 9kDa, V0 subunit e1 |
|  | ATP6V0E1 | ATPase, H+ transporting, lysosomal 9kDa, V0 subunit e1 |
|  | PANK2 | pantothenate kinase 2 |
|  | PANK2 | pantothenate kinase 2 |
|  | SEPHS2 | selenophosphate synthetase 2 |
|  | AOC3 | amine oxidase, copper containing 3 (vascular adhesion protein 1) |
|  | AK2 | adenylate kinase 2 |
|  | AK2 | adenylate kinase 2 |
|  | AK2 | adenylate kinase 2 |
|  | AK2 | adenylate kinase 2 |
|  | NDUFS6 | NADH dehydrogenase (ubiquinone) Fe-S protein 6, 13kDa (NADH-coenzyme Q reductase) |
|  | HSD17B10 | hydroxysteroid (17-beta) dehydrogenase 10 |
|  | ASAH1 | N-acylsphingosine amidohydrolase (acid ceramidase) 1 |
|  | COX4I1 | cytochrome c oxidase subunit IV isoform 1 |
|  | B4GALT4 | UDP-Gal:betaGlcNAc beta 1,4- galactosyltransferase, polypeptide 4 |
|  | PHGDH | phosphoglycerate dehydrogenase |
|  | DHRS3 | dehydrogenase/reductase (SDR family) member 3 |
|  | PGAP1 | post-GPI attachment to proteins 1 |
|  | PGAP1 | post-GPI attachment to proteins 1 |
|  | UGT1A3 | UDP glucuronosyltransferase 1 family, polypeptide A3 |
|  | TK2 | thymidine kinase 2, mitochondrial |
|  | C1GALT1 | core 1 synthase, glycoprotein-N-acetylgalactosamine 3-beta-galactosyltransferase, 1 |
|  | B3GALNT1 | beta-1,3-N-acetylgalactosaminyltransferase 1 (globoside blood group) |
|  | B3GALNT1 | beta-1,3-N-acetylgalactosaminyltransferase 1 (globoside blood group) |
|  | NDUFB2 | NADH dehydrogenase (ubiquinone) 1 beta subcomplex, 2, 8kDa |
|  | NDUFB2 | NADH dehydrogenase (ubiquinone) 1 beta subcomplex, 2, 8kDa |
|  | CTPS2 | CTP synthase II |
|  | GMPS | guanine monphosphate synthetase |
|  | PTS | 6-pyruvoyltetrahydropterin synthase |
|  | AHCY | adenosylhomocysteinase |
|  | ST6GALNAC1 | ST6 (alpha-N-acetyl-neuraminyl-2,3-beta-galactosyl-1,3)-N-acetylgalactosaminide alpha-2,6-sialyltransferase 1 |
|  | GMDS | GDP-mannose 4,6-dehydratase |
|  | ALDH18A1 | aldehyde dehydrogenase 18 family, member A1 |
|  | ALDH18A1 | aldehyde dehydrogenase 18 family, member A1 |
|  | ALDH3A2 | aldehyde dehydrogenase 3 family, member A2 |
|  | NT5E | 5'-nucleotidase, ecto (CD73) |
|  | POLR3F | polymerase (RNA) III (DNA directed) polypeptide F, 39 kDa |
|  | MTHFD1 | methylenetetrahydrofolate dehydrogenase (NADP+ dependent) 1, methenyltetrahydrofolate cyclohydrolase, formyltetrahydrofolate synthetase |
|  | CHSY3 | chondroitin sulfate synthase 3 |
|  | SUCLA2 | succinate-CoA ligase, ADP-forming, beta subunit |
|  | ATP5G1 | ATP synthase, H+ transporting, mitochondrial F0 complex, subunit C1 (subunit 9) |
|  | POLR2I | polymerase (RNA) II (DNA directed) polypeptide I, 14.5kDa |
|  | NME6 | non-metastatic cells 6, protein expressed in (nucleoside-diphosphate kinase) |
|  | PPAT | phosphoribosyl pyrophosphate amidotransferase |
|  | PPAT | phosphoribosyl pyrophosphate amidotransferase |
|  | NSDHL | NAD(P) dependent steroid dehydrogenase-like |
|  | AMD1 | adenosylmethionine decarboxylase 1 |
|  | AMD1 | adenosylmethionine decarboxylase 1 |
|  | CYP3A7 | cytochrome P450, family 3, subfamily A, polypeptide 7 |
|  | GBA | glucosidase, beta; acid (includes glucosylceramidase) |
|  | SCP2 | sterol carrier protein 2 |
|  | TM7SF2 | transmembrane 7 superfamily member 2 |
|  | MAN1C1 | mannosidase, alpha, class 1C, member 1 |
|  | MAN1C1 | mannosidase, alpha, class 1C, member 1 |
|  | PIK3C2A | phosphoinositide-3-kinase, class 2, alpha polypeptide |
|  | PIK3C2A | phosphoinositide-3-kinase, class 2, alpha polypeptide |
|  | GLCE | glucuronic acid epimerase |
|  | PGD | phosphogluconate dehydrogenase |
|  | FECH | ferrochelatase (protoporphyria) |
|  | IDS | iduronate 2-sulfatase |
|  | SDHB | succinate dehydrogenase complex, subunit B, iron sulfur (Ip) |
|  | COX17 | COX17 cytochrome c oxidase assembly homolog (S. cerevisiae) |
|  | MGAT2 | mannosyl (alpha-1,6-)-glycoprotein beta-1,2-N-acetylglucosaminyltransferase |
|  | MGAT2 | mannosyl (alpha-1,6-)-glycoprotein beta-1,2-N-acetylglucosaminyltransferase |
|  | REV3L | REV3-like, catalytic subunit of DNA polymerase zeta (yeast) |
|  | REV3L | REV3-like, catalytic subunit of DNA polymerase zeta (yeast) |
|  | CBR1 | carbonyl reductase 1 |
|  | GALNT2 | UDP-N-acetyl-alpha-D-galactosamine:polypeptide N-acetylgalactosaminyltransferase 2 (GalNAc-T2) |
|  | GALNT2 | UDP-N-acetyl-alpha-D-galactosamine:polypeptide N-acetylgalactosaminyltransferase 2 (GalNAc-T2) |
|  | UAP1 | UDP-N-acteylglucosamine pyrophosphorylase 1 |
|  | POLR1D | polymerase (RNA) I polypeptide D, 16kDa |
|  | PPAP2B | phosphatidic acid phosphatase type 2B |
|  | PPAP2B | phosphatidic acid phosphatase type 2B |
|  | PRPS2 | phosphoribosyl pyrophosphate synthetase 2 |
|  | PRPS2 | phosphoribosyl pyrophosphate synthetase 2 |
|  | FDPS | farnesyl diphosphate synthase (farnesyl pyrophosphate synthetase, dimethylallyltranstransferase, geranyltranstransferase) |
|  | POLR2E | polymerase (RNA) II (DNA directed) polypeptide E, 25kDa |
|  | NANP | N-acetylneuraminic acid phosphatase |
|  | POLD2 | polymerase (DNA directed), delta 2, regulatory subunit 50kDa |
|  | HIBADH | 3-hydroxyisobutyrate dehydrogenase |
|  | CHST6 | carbohydrate (N-acetylglucosamine 6-O) sulfotransferase 6 |
|  | NDUFC2 | NADH dehydrogenase (ubiquinone) 1, subcomplex unknown, 2, 14.5kDa |
|  | UGP2 | UDP-glucose pyrophosphorylase 2 |
|  | UGP2 | UDP-glucose pyrophosphorylase 2 |
|  | CYP2C9 | cytochrome P450, family 2, subfamily C, polypeptide 9 |
|  | SUCLG1 | succinate-CoA ligase, alpha subunit |
|  | MDH2 | malate dehydrogenase 2, NAD (mitochondrial) |
|  | XYLT1 | xylosyltransferase I |
|  | DGKA | diacylglycerol kinase, alpha 80kDa |
|  | UCK2 | uridine-cytidine kinase 2 |
|  | PNLIPRP1 | pancreatic lipase-related protein 1 |
|  | CHKB | choline kinase beta |
|  | ATP6V1B2 | ATPase, H+ transporting, lysosomal 56/58kDa, V1 subunit B2 |
|  | POLD1 | polymerase (DNA directed), delta 1, catalytic subunit 125kDa |
|  | EXT2 | exostoses (multiple) 2 |
|  | EXT2 | exostoses (multiple) 2 |
|  | UQCRFS1 | ubiquinol-cytochrome c reductase, Rieske iron-sulfur polypeptide 1 |
|  | CAD | carbamoyl-phosphate synthetase 2, aspartate transcarbamylase, and dihydroorotase |
|  | UXS1 | UDP-glucuronate decarboxylase 1 |
|  | UXS1 | UDP-glucuronate decarboxylase 1 |
|  | ATP6V0B | ATPase, H+ transporting, lysosomal 21kDa, V0 subunit b |
|  | IDI1 | isopentenyl-diphosphate delta isomerase 1 |
|  | IDI1 | isopentenyl-diphosphate delta isomerase 1 |
|  | PANK4 | pantothenate kinase 4 |
|  | RPN2 | ribophorin II |
|  | HIBCH | 3-hydroxyisobutyryl-Coenzyme A hydrolase |
|  | APIP | APAF1 interacting protein |
|  | SPTLC1 | serine palmitoyltransferase, long chain base subunit 1 |
|  | AMACR | alpha-methylacyl-CoA racemase |
|  | SEPHS1 | selenophosphate synthetase 1 |
|  | ALG10B | asparagine-linked glycosylation 10, alpha-1,2-glucosyltransferase homolog B (yeast) |
|  | ALG10B | asparagine-linked glycosylation 10, alpha-1,2-glucosyltransferase homolog B (yeast) |
|  | ACSL5 | acyl-CoA synthetase long-chain family member 5 |
|  | ACSL5 | acyl-CoA synthetase long-chain family member 5 |
|  | MCEE | methylmalonyl CoA epimerase |
|  | COQ5 | coenzyme Q5 homolog, methyltransferase (S. cerevisiae) |
|  | UROS | uroporphyrinogen III synthase |
|  | POLR2G | polymerase (RNA) II (DNA directed) polypeptide G |
|  | RFK | riboflavin kinase |
|  | B3GALT4 | UDP-Gal:betaGlcNAc beta 1,3-galactosyltransferase, polypeptide 4 |
|  | GLUD2 | glutamate dehydrogenase 2 |
|  | PPT1 | palmitoyl-protein thioesterase 1 |
|  | CPOX | coproporphyrinogen oxidase |
|  | MTMR6 | myotubularin related protein 6 |
|  | PON2 | paraoxonase 2 |
|  | PAFAH1B3 | platelet-activating factor acetylhydrolase, isoform Ib, subunit 3 (29kDa) |
|  | TCIRG1 | T-cell, immune regulator 1, ATPase, H+ transporting, lysosomal V0 subunit A3 |
|  | NDUFA11 | NADH dehydrogenase (ubiquinone) 1 alpha subcomplex, 11, 14.7kDa |
|  | MAOA | monoamine oxidase A |
|  | MAOA | monoamine oxidase A |
|  | MAOA | monoamine oxidase A |
|  | NDUFB7 | NADH dehydrogenase (ubiquinone) 1 beta subcomplex, 7, 18kDa |
|  | ST3GAL1 | ST3 beta-galactoside alpha-2,3-sialyltransferase 1 |
|  | PSPH | phosphoserine phosphatase |
|  | RPIA | ribose 5-phosphate isomerase A |
|  | UGT1A1 | UDP glucuronosyltransferase 1 family, polypeptide A1 |
|  | NADSYN1 | NAD synthetase 1 |
|  | CMAS | cytidine monophosphate N-acetylneuraminic acid synthetase |
|  | GCLM | glutamate-cysteine ligase, modifier subunit |
|  | GCLM | glutamate-cysteine ligase, modifier subunit |
|  | CDS2 | CDP-diacylglycerol synthase (phosphatidate cytidylyltransferase) 2 |
|  | PGM2 | phosphoglucomutase 2 |
|  | PGM2 | phosphoglucomutase 2 |
|  | PGM2 | phosphoglucomutase 2 |
|  | ENO2 | enolase 2 (gamma, neuronal) |
|  | JMJD7-PLA2G4B | JMJD7-PLA2G4B readthrough |
|  | SORD | sorbitol dehydrogenase |
|  | GNE | glucosamine (UDP-N-acetyl)-2-epimerase/N-acetylmannosamine kinase |
|  | CYP3A43 | cytochrome P450, family 3, subfamily A, polypeptide 43 |
|  | APRT | adenine phosphoribosyltransferase |
|  | MAN1A1 | mannosidase, alpha, class 1A, member 1 |
|  | ADH5 | alcohol dehydrogenase 5 (class III), chi polypeptide |
|  | ADH5 | alcohol dehydrogenase 5 (class III), chi polypeptide |
|  | GART | phosphoribosylglycinamide formyltransferase, phosphoribosylglycinamide synthetase, phosphoribosylaminoimidazole synthetase |
|  | GART | phosphoribosylglycinamide formyltransferase, phosphoribosylglycinamide synthetase, phosphoribosylaminoimidazole synthetase |
|  | GART | phosphoribosylglycinamide formyltransferase, phosphoribosylglycinamide synthetase, phosphoribosylaminoimidazole synthetase |
|  | ABO | ABO blood group (transferase A, alpha 1-3-N-acetylgalactosaminyltransferase; transferase B, alpha 1-3-galactosyltransferase) |
|  | CKMT1B | creatine kinase, mitochondrial 1B |
|  | FUT9 | fucosyltransferase 9 (alpha (1,3) fucosyltransferase) |
|  | PNLIP | pancreatic lipase |
|  | QDPR | quinoid dihydropteridine reductase |
|  | UGT1A6 | UDP glucuronosyltransferase 1 family, polypeptide A6 |
|  | SHMT2 | serine hydroxymethyltransferase 2 (mitochondrial) |
|  | SHMT2 | serine hydroxymethyltransferase 2 (mitochondrial) |
|  | ALDH7A1 | aldehyde dehydrogenase 7 family, member A1 |
|  | ALDH7A1 | aldehyde dehydrogenase 7 family, member A1 |
|  | NDUFS8 | NADH dehydrogenase (ubiquinone) Fe-S protein 8, 23kDa (NADH-coenzyme Q reductase) |
|  | POLR1B | polymerase (RNA) I polypeptide B, 128kDa |
|  | POLR3C | polymerase (RNA) III (DNA directed) polypeptide C (62kD) |
|  | DBT | dihydrolipoamide branched chain transacylase E2 |
|  | PLA2G6 | phospholipase A2, group VI (cytosolic, calcium-independent) |
|  | GCLC | glutamate-cysteine ligase, catalytic subunit |
|  | GCLC | glutamate-cysteine ligase, catalytic subunit |
|  | TRIT1 | tRNA isopentenyltransferase 1 |
|  | POLD3 | polymerase (DNA-directed), delta 3, accessory subunit |
|  | GALNT14 | UDP-N-acetyl-alpha-D-galactosamine:polypeptide N-acetylgalactosaminyltransferase 14 (GalNAc-T14) |
|  | NAT1 | N-acetyltransferase 1 (arylamine N-acetyltransferase) |
|  | PLA2G1B | phospholipase A2, group IB (pancreas) |
|  | DHFR | dihydrofolate reductase |
|  | DHFR | dihydrofolate reductase |
|  | DHFR | dihydrofolate reductase |
|  | ACAT1 | acetyl-Coenzyme A acetyltransferase 1 |
|  | GLB1 | galactosidase, beta 1 |
|  | FPGT | fucose-1-phosphate guanylyltransferase |
|  | ACADM | acyl-Coenzyme A dehydrogenase, C-4 to C-12 straight chain |
|  | ATP6V1C1 | ATPase, H+ transporting, lysosomal 42kDa, V1 subunit C1 |
|  | ATP6V1C1 | ATPase, H+ transporting, lysosomal 42kDa, V1 subunit C1 |
|  | POLR1C | polymerase (RNA) I polypeptide C, 30kDa |
|  | POLR1C | polymerase (RNA) I polypeptide C, 30kDa |
|  | PTGES | prostaglandin E synthase |
|  | ATP5O | ATP synthase, H+ transporting, mitochondrial F1 complex, O subunit |
| **KEGG pathway----Spliceosome----03040** | **Gene Symbol** | **Gene Name** |
|  | WBP11 | WW domain binding protein 11 |
|  | WBP11 | WW domain binding protein 11 |
|  | SNRNP40 | small nuclear ribonucleoprotein 40kDa (U5) |
|  | CHERP | calcium homeostasis endoplasmic reticulum protein |
|  | SF3B1 | splicing factor 3b, subunit 1, 155kDa |
|  | PLRG1 | pleiotropic regulator 1 (PRL1 homolog, Arabidopsis) |
|  | RBM8A | RNA binding motif protein 8A |
|  | RBM8A | RNA binding motif protein 8A |
|  | SNRPF | small nuclear ribonucleoprotein polypeptide F |
|  | USP39 | ubiquitin specific peptidase 39 |
|  | PPIL1 | peptidylprolyl isomerase (cyclophilin)-like 1 |
|  | SNRPE | small nuclear ribonucleoprotein polypeptide E |
|  | SNRPE | small nuclear ribonucleoprotein polypeptide E |
|  | SNRPD3 | small nuclear ribonucleoprotein D3 polypeptide 18kDa |
|  | PCBP1 | poly(rC) binding protein 1 |
|  | ZMAT2 | zinc finger, matrin type 2 |
|  | HSPA1A | heat shock 70kDa protein 1A |
|  | HNRNPA3 | heterogeneous nuclear ribonucleoprotein A3 |
|  | HNRNPA3 | heterogeneous nuclear ribonucleoprotein A3 |
|  | SF3B5 | splicing factor 3b, subunit 5, 10kDa |
|  | MAGOHB | mago-nashi homolog B (Drosophila) |
|  | SNRPC | small nuclear ribonucleoprotein polypeptide C |
|  | SNRPD1 | small nuclear ribonucleoprotein D1 polypeptide 16kDa |
|  | SNRPD1 | small nuclear ribonucleoprotein D1 polypeptide 16kDa |
|  | SFRS2 | splicing factor, arginine/serine-rich 2 |
|  | BCAS2 | breast carcinoma amplified sequence 2 |
|  | EIF4A3 | eukaryotic translation initiation factor 4A, isoform 3 |
|  | SFRS4 | splicing factor, arginine/serine-rich 4 |
|  | TRA2B | transformer 2 beta homolog (Drosophila) |
|  | TRA2B | transformer 2 beta homolog (Drosophila) |
|  | SF3A1 | splicing factor 3a, subunit 1, 120kDa |
|  | SFRS13A | splicing factor, arginine/serine-rich 13A |
|  | SFRS13A | splicing factor, arginine/serine-rich 13A |
|  | SFRS13A | splicing factor, arginine/serine-rich 13A |
|  | CDC5L | CDC5 cell division cycle 5-like (S. pombe) |
|  | CDC5L | CDC5 cell division cycle 5-like (S. pombe) |
|  | SNRPB | small nuclear ribonucleoprotein polypeptides B and B1 |
|  | HSPA8 | heat shock 70kDa protein 8 |
|  | HSPA8 | heat shock 70kDa protein 8 |
|  | HSPA8 | heat shock 70kDa protein 8 |
|  | PPIE | peptidylprolyl isomerase E (cyclophilin E) |
|  | PPIE | peptidylprolyl isomerase E (cyclophilin E) |
|  | LSM7 | LSM7 homolog, U6 small nuclear RNA associated (S. cerevisiae) |
|  | HNRNPC | heterogeneous nuclear ribonucleoprotein C (C1/C2) |
|  | CRNKL1 | crooked neck pre-mRNA splicing factor-like 1 (Drosophila) |
|  | MAGOH | mago-nashi homolog, proliferation-associated (Drosophila) |
|  | MAGOH | mago-nashi homolog, proliferation-associated (Drosophila) |
|  | LSM8 | LSM8 homolog, U6 small nuclear RNA associated (S. cerevisiae) |
|  | TXNL4A | thioredoxin-like 4A |
|  | SNRNP27 | small nuclear ribonucleoprotein 27kDa (U4/U6.U5) |
|  | SNRNP27 | small nuclear ribonucleoprotein 27kDa (U4/U6.U5) |
|  | HSPA6 | heat shock 70kDa protein 6 (HSP70B') |
|  | SFRS1 | splicing factor, arginine/serine-rich 1 |
|  | DDX46 | DEAD (Asp-Glu-Ala-Asp) box polypeptide 46 |
|  | LSM6 | LSM6 homolog, U6 small nuclear RNA associated (S. cerevisiae) |
|  | SR140 | U2-associated SR140 protein |
|  | PRPF4 | PRP4 pre-mRNA processing factor 4 homolog (yeast) |
|  | PRPF4 | PRP4 pre-mRNA processing factor 4 homolog (yeast) |
|  | SNRPG | small nuclear ribonucleoprotein polypeptide G |
|  | DDX42 | DEAD (Asp-Glu-Ala-Asp) box polypeptide 42 |
|  | SNRPA1 | small nuclear ribonucleoprotein polypeptide A' |
|  | SNRPA1 | small nuclear ribonucleoprotein polypeptide A' |
|  | PRPF38B | PRP38 pre-mRNA processing factor 38 (yeast) domain containing B |
|  | CDC40 | cell division cycle 40 homolog (S. cerevisiae) |
|  | PRPF31 | PRP31 pre-mRNA processing factor 31 homolog (S. cerevisiae) |
|  | PRPF31 | PRP31 pre-mRNA processing factor 31 homolog (S. cerevisiae) |
|  | ISY1 | ISY1 splicing factor homolog (S. cerevisiae) |
|  | ISY1 | ISY1 splicing factor homolog (S. cerevisiae) |
|  | ISY1 | ISY1 splicing factor homolog (S. cerevisiae) |
|  | SFRS6 | splicing factor, arginine/serine-rich 6 |
|  | SF3A3 | splicing factor 3a, subunit 3, 60kDa |
|  | LSM4 | LSM4 homolog, U6 small nuclear RNA associated (S. cerevisiae) |
|  | ACIN1 | apoptotic chromatin condensation inducer 1 |
|  | PRPF18 | PRP18 pre-mRNA processing factor 18 homolog (S. cerevisiae) |
|  | SNW1 | SNW domain containing 1 |
|  | LSM5 | LSM5 homolog, U6 small nuclear RNA associated (S. cerevisiae) |
|  | LSM5 | LSM5 homolog, U6 small nuclear RNA associated (S. cerevisiae) |
|  | THOC4 | THO complex 4 |
|  | THOC4 | THO complex 4 |
|  | HNRNPM | heterogeneous nuclear ribonucleoprotein M |
|  | HNRNPM | heterogeneous nuclear ribonucleoprotein M |
|  | PRPF40A | PRP40 pre-mRNA processing factor 40 homolog A (S. cerevisiae) |
|  | TRA2A | transformer 2 alpha homolog (Drosophila) |
|  | SF3B14 | splicing factor 3B, 14 kDa subunit |
|  | SF3A2 | splicing factor 3a, subunit 2, 66kDa |
|  | HSPA1B | heat shock 70kDa protein 1B |
|  | DDX23 | DEAD (Asp-Glu-Ala-Asp) box polypeptide 23 |
|  | DDX23 | DEAD (Asp-Glu-Ala-Asp) box polypeptide 23 |
|  | NCBP1 | nuclear cap binding protein subunit 1, 80kDa |
|  | RBM25 | RNA binding motif protein 25 |
|  | RBM25 | RNA binding motif protein 25 |
|  | RBM25 | RNA binding motif protein 25 |
|  | RBM25 | RNA binding motif protein 25 |
|  | RBMX | RNA binding motif protein, X-linked |
|  | THOC2 | THO complex 2 |
|  | LSM3 | LSM3 homolog, U6 small nuclear RNA associated (S. cerevisiae) |
|  | LSM2 | LSM2 homolog, U6 small nuclear RNA associated (S. cerevisiae) |
|  | HNRNPK | heterogeneous nuclear ribonucleoprotein K |
|  | NCBP2 | nuclear cap binding protein subunit 2, 20kDa |
|  | SFRS3 | splicing factor, arginine/serine-rich 3 |
|  | PPIH | peptidylprolyl isomerase H (cyclophilin H) |
|  | SNRPD2 | small nuclear ribonucleoprotein D2 polypeptide 16.5kDa |
|  | SFRS9 | splicing factor, arginine/serine-rich 9 |
|  | SFRS9 | splicing factor, arginine/serine-rich 9 |
| **KEGG pathway----Cell cycle----04110** | **Gene Symbol** | **Gene Name** |
|  | PTTG1 | pituitary tumor-transforming 1 |
|  | CUL1 | cullin 1 |
|  | YWHAE | tyrosine 3-monooxygenase/tryptophan 5-monooxygenase activation protein, epsilon polypeptide |
|  | CCNB1 | cyclin B1 |
|  | CCNB1 | cyclin B1 |
|  | CCNE1 | cyclin E1 |
|  | CCND1 | cyclin D1 |
|  | CCND1 | cyclin D1 |
|  | CDKN2C | cyclin-dependent kinase inhibitor 2C (p18, inhibits CDK4) |
|  | MCM4 | minichromosome maintenance complex component 4 |
|  | MCM4 | minichromosome maintenance complex component 4 |
|  | MCM4 | minichromosome maintenance complex component 4 |
|  | MDM2 | Mdm2 p53 binding protein homolog (mouse) |
|  | PCNA | proliferating cell nuclear antigen |
|  | CCNA2 | cyclin A2 |
|  | CCNA2 | cyclin A2 |
|  | ANAPC11 | anaphase promoting complex subunit 11 |
|  | ANAPC10 | anaphase promoting complex subunit 10 |
|  | CDKN1B | cyclin-dependent kinase inhibitor 1B (p27, Kip1) |
|  | CDC25B | cell division cycle 25 homolog B (S. pombe) |
|  | CDK6 | cyclin-dependent kinase 6 |
|  | CDK6 | cyclin-dependent kinase 6 |
|  | RBX1 | ring-box 1 |
|  | CDK4 | cyclin-dependent kinase 4 |
|  | CDC20 | cell division cycle 20 homolog (S. cerevisiae) |
|  | STAG2 | stromal antigen 2 |
|  | STAG2 | stromal antigen 2 |
|  | STAG2 | stromal antigen 2 |
|  | E2F3 | E2F transcription factor 3 |
|  | YWHAQ | tyrosine 3-monooxygenase/tryptophan 5-monooxygenase activation protein, theta polypeptide |
|  | YWHAQ | tyrosine 3-monooxygenase/tryptophan 5-monooxygenase activation protein, theta polypeptide |
|  | CDC6 | cell division cycle 6 homolog (S. cerevisiae) |
|  | CDC6 | cell division cycle 6 homolog (S. cerevisiae) |
|  | BUB1B | budding uninhibited by benzimidazoles 1 homolog beta (yeast) |
|  | CDK7 | cyclin-dependent kinase 7 |
|  | CDC23 | cell division cycle 23 homolog (S. cerevisiae) |
|  | MCM2 | minichromosome maintenance complex component 2 |
|  | CDC25C | cell division cycle 25 homolog C (S. pombe) |
|  | CDC2 | cell division cycle 2, G1 to S and G2 to M |
|  | CDC2 | cell division cycle 2, G1 to S and G2 to M |
|  | CDC2 | cell division cycle 2, G1 to S and G2 to M |
|  | SMAD3 | SMAD family member 3 |
|  | SMAD3 | SMAD family member 3 |
|  | SMAD3 | SMAD family member 3 |
|  | CDC45L | CDC45 cell division cycle 45-like (S. cerevisiae) |
|  | SMAD2 | SMAD family member 2 |
|  | SMAD2 | SMAD family member 2 |
|  | SMAD2 | SMAD family member 2 |
|  | GADD45B | growth arrest and DNA-damage-inducible, beta |
|  | GADD45B | growth arrest and DNA-damage-inducible, beta |
|  | MAD2L1 | MAD2 mitotic arrest deficient-like 1 (yeast) |
|  | MAD2L1 | MAD2 mitotic arrest deficient-like 1 (yeast) |
|  | SMC1A | structural maintenance of chromosomes 1A |
|  | SKP1 | S-phase kinase-associated protein 1 |
|  | SKP1 | S-phase kinase-associated protein 1 |
|  | TFDP2 | transcription factor Dp-2 (E2F dimerization partner 2) |
|  | SMAD4 | SMAD family member 4 |
|  | SMAD4 | SMAD family member 4 |
|  | MCM3 | minichromosome maintenance complex component 3 |
|  | MCM6 | minichromosome maintenance complex component 6 |
|  | DBF4 | DBF4 homolog (S. cerevisiae) |
|  | CHEK1 | CHK1 checkpoint homolog (S. pombe) |
|  | CHEK1 | CHK1 checkpoint homolog (S. pombe) |
|  | CHEK1 | CHK1 checkpoint homolog (S. pombe) |
|  | MCM7 | minichromosome maintenance complex component 7 |
|  | RBL2 | retinoblastoma-like 2 (p130) |
|  | MAD2L2 | MAD2 mitotic arrest deficient-like 2 (yeast) |
|  | SMC3 | structural maintenance of chromosomes 3 |
|  | SMC3 | structural maintenance of chromosomes 3 |
|  | SMC3 | structural maintenance of chromosomes 3 |
|  | CDC7 | cell division cycle 7 homolog (S. cerevisiae) |
|  | MYC | v-myc myelocytomatosis viral oncogene homolog (avian) |
|  | MCM5 | minichromosome maintenance complex component 5 |
|  | MCM5 | minichromosome maintenance complex component 5 |
|  | TTK | TTK protein kinase |
|  | ATR | ataxia telangiectasia and Rad3 related |
|  | BUB1 | budding uninhibited by benzimidazoles 1 homolog (yeast) |
|  | CCND2 | cyclin D2 |
|  | TGFB1 | transforming growth factor, beta 1 |
|  | CDC25A | cell division cycle 25 homolog A (S. pombe) |
|  | BUB3 | budding uninhibited by benzimidazoles 3 homolog (yeast) |
|  | BUB3 | budding uninhibited by benzimidazoles 3 homolog (yeast) |
|  | BUB3 | budding uninhibited by benzimidazoles 3 homolog (yeast) |
|  | STAG1 | stromal antigen 1 |
|  | PRKDC | protein kinase, DNA-activated, catalytic polypeptide |
|  | PRKDC | protein kinase, DNA-activated, catalytic polypeptide |
|  | SKP2 | S-phase kinase-associated protein 2 (p45) |
|  | SKP2 | S-phase kinase-associated protein 2 (p45) |
|  | CDC26 | cell division cycle 26 homolog (S. cerevisiae) |
|  | GADD45A | growth arrest and DNA-damage-inducible, alpha |
|  | CDC27 | cell division cycle 27 homolog (S. cerevisiae) |
|  | CDC27 | cell division cycle 27 homolog (S. cerevisiae) |
|  | TFDP1 | transcription factor Dp-1 |
|  | ORC5L | origin recognition complex, subunit 5-like (yeast) |
|  | ORC5L | origin recognition complex, subunit 5-like (yeast) |
|  | YWHAZ | tyrosine 3-monooxygenase/tryptophan 5-monooxygenase activation protein, zeta polypeptide |
|  | YWHAZ | tyrosine 3-monooxygenase/tryptophan 5-monooxygenase activation protein, zeta polypeptide |
|  | CCNB2 | cyclin B2 |
|  | YWHAH | tyrosine 3-monooxygenase/tryptophan 5-monooxygenase activation protein, eta polypeptide |
|  | CDK2 | cyclin-dependent kinase 2 |
|  | ESPL1 | extra spindle pole bodies homolog 1 (S. cerevisiae) |
|  | YWHAB | tyrosine 3-monooxygenase/tryptophan 5-monooxygenase activation protein, beta polypeptide |
|  | CDKN2A | cyclin-dependent kinase inhibitor 2A (melanoma, p16, inhibits CDK4) |
|  | ORC6L | origin recognition complex, subunit 6 like (yeast) |
|  | SFN | stratifin |
|  | SFN | stratifin |
|  | RAD21 | RAD21 homolog (S. pombe) |
| **KEGG pathway----Pathways in cancer----05200** | **Gene Symbol** | **Gene Name** |
|  | MAPK8 | mitogen-activated protein kinase 8 |
|  | EPAS1 | endothelial PAS domain protein 1 |
|  | RALBP1 | ralA binding protein 1 |
|  | RALBP1 | ralA binding protein 1 |
|  | RXRA | retinoid X receptor, alpha |
|  | MAP2K1 | mitogen-activated protein kinase kinase 1 |
|  | XIAP | X-linked inhibitor of apoptosis |
|  | XIAP | X-linked inhibitor of apoptosis |
|  | APC | adenomatous polyposis coli |
|  | CTBP2 | C-terminal binding protein 2 |
|  | CTBP2 | C-terminal binding protein 2 |
|  | CTBP2 | C-terminal binding protein 2 |
|  | CEBPA | CCAAT/enhancer binding protein (C/EBP), alpha |
|  | BCL2L1 | BCL2-like 1 |
|  | FZD2 | frizzled homolog 2 (Drosophila) |
|  | PRKCA | protein kinase C, alpha |
|  | PDGFRA | platelet-derived growth factor receptor, alpha polypeptide |
|  | BIRC2 | baculoviral IAP repeat-containing 2 |
|  | CCNE1 | cyclin E1 |
|  | WNT5A | wingless-type MMTV integration site family, member 5A |
|  | WNT5A | wingless-type MMTV integration site family, member 5A |
|  | CRKL | v-crk sarcoma virus CT10 oncogene homolog (avian)-like |
|  | CCND1 | cyclin D1 |
|  | CCND1 | cyclin D1 |
|  | MDM2 | Mdm2 p53 binding protein homolog (mouse) |
|  | LAMA3 | laminin, alpha 3 |
|  | CDH1 | cadherin 1, type 1, E-cadherin (epithelial) |
|  | RALB | v-ral simian leukemia viral oncogene homolog B (ras related; GTP binding protein) |
|  | RALB | v-ral simian leukemia viral oncogene homolog B (ras related; GTP binding protein) |
|  | IKBKB | inhibitor of kappa light polypeptide gene enhancer in B-cells, kinase beta |
|  | IKBKB | inhibitor of kappa light polypeptide gene enhancer in B-cells, kinase beta |
|  | MITF | microphthalmia-associated transcription factor |
|  | MITF | microphthalmia-associated transcription factor |
|  | PIK3CA | phosphoinositide-3-kinase, catalytic, alpha polypeptide |
|  | CDKN1B | cyclin-dependent kinase inhibitor 1B (p27, Kip1) |
|  | COL4A1 | collagen, type IV, alpha 1 |
|  | TFG | TRK-fused gene |
|  | EGLN3 | egl nine homolog 3 (C. elegans) |
|  | EGLN3 | egl nine homolog 3 (C. elegans) |
|  | CDK6 | cyclin-dependent kinase 6 |
|  | CDK6 | cyclin-dependent kinase 6 |
|  | PDGFRB | platelet-derived growth factor receptor, beta polypeptide |
|  | FZD1 | frizzled homolog 1 (Drosophila) |
|  | LAMA4 | laminin, alpha 4 |
|  | LAMA4 | laminin, alpha 4 |
|  | NRAS | neuroblastoma RAS viral (v-ras) oncogene homolog |
|  | TPM3 | tropomyosin 3 |
|  | BIRC3 | baculoviral IAP repeat-containing 3 |
|  | RBX1 | ring-box 1 |
|  | PPARG | peroxisome proliferator-activated receptor gamma |
|  | ACVR1B | activin A receptor, type IB |
|  | HSP90AB1 | heat shock protein 90kDa alpha (cytosolic), class B member 1 |
|  | HSP90AB1 | heat shock protein 90kDa alpha (cytosolic), class B member 1 |
|  | CDK4 | cyclin-dependent kinase 4 |
|  | FH | fumarate hydratase |
|  | CBLB | Cas-Br-M (murine) ecotropic retroviral transforming sequence b |
|  | E2F3 | E2F transcription factor 3 |
|  | MMP1 | matrix metallopeptidase 1 (interstitial collagenase) |
|  | VEGFA | vascular endothelial growth factor A |
|  | VEGFA | vascular endothelial growth factor A |
|  | VEGFA | vascular endothelial growth factor A |
|  | FGFR1 | fibroblast growth factor receptor 1 |
|  | FZD7 | frizzled homolog 7 (Drosophila) |
|  | FZD7 | frizzled homolog 7 (Drosophila) |
|  | SMAD3 | SMAD family member 3 |
|  | SMAD3 | SMAD family member 3 |
|  | SMAD3 | SMAD family member 3 |
|  | PIAS3 | protein inhibitor of activated STAT, 3 |
|  | PTGS2 | prostaglandin-endoperoxide synthase 2 (prostaglandin G/H synthase and cyclooxygenase) |
|  | PTGS2 | prostaglandin-endoperoxide synthase 2 (prostaglandin G/H synthase and cyclooxygenase) |
|  | BRCA2 | breast cancer 2, early onset |
|  | SMAD2 | SMAD family member 2 |
|  | SMAD2 | SMAD family member 2 |
|  | SMAD2 | SMAD family member 2 |
|  | JAK1 | Janus kinase 1 |
|  | IL8 | interleukin 8 |
|  | ITGA2 | integrin, alpha 2 (CD49B, alpha 2 subunit of VLA-2 receptor) |
|  | LAMB1 | laminin, beta 1 |
|  | LAMB1 | laminin, beta 1 |
|  | ITGB1 | integrin, beta 1 (fibronectin receptor, beta polypeptide, antigen CD29 includes MDF2, MSK12) |
|  | ITGB1 | integrin, beta 1 (fibronectin receptor, beta polypeptide, antigen CD29 includes MDF2, MSK12) |
|  | RUNX1 | runt-related transcription factor 1 |
|  | MAPK10 | mitogen-activated protein kinase 10 |
|  | TRAF5 | TNF receptor-associated factor 5 |
|  | MAPK1 | mitogen-activated protein kinase 1 |
|  | MAPK1 | mitogen-activated protein kinase 1 |
|  | LAMA5 | laminin, alpha 5 |
|  | LAMC2 | laminin, gamma 2 |
|  | SMAD4 | SMAD family member 4 |
|  | SMAD4 | SMAD family member 4 |
|  | MMP9 | matrix metallopeptidase 9 (gelatinase B, 92kDa gelatinase, 92kDa type IV collagenase) |
|  | HSP90B1 | heat shock protein 90kDa beta (Grp94), member 1 |
|  | HSP90B1 | heat shock protein 90kDa beta (Grp94), member 1 |
|  | HSP90B1 | heat shock protein 90kDa beta (Grp94), member 1 |
|  | BRAF | v-raf murine sarcoma viral oncogene homolog B1 |
|  | FN1 | fibronectin 1 |
|  | FN1 | fibronectin 1 |
|  | FN1 | fibronectin 1 |
|  | FN1 | fibronectin 1 |
|  | FOXO1 | forkhead box O1 |
|  | JUP | junction plakoglobin |
|  | CTNNA1 | catenin (cadherin-associated protein), alpha 1, 102kDa |
|  | WNT2 | wingless-type MMTV integration site family member 2 |
|  | RALGDS | ral guanine nucleotide dissociation stimulator |
|  | RALGDS | ral guanine nucleotide dissociation stimulator |
|  | STK36 | serine/threonine kinase 36, fused homolog (Drosophila) |
|  | STK36 | serine/threonine kinase 36, fused homolog (Drosophila) |
|  | TGFBR1 | transforming growth factor, beta receptor 1 |
|  | TGFBR1 | transforming growth factor, beta receptor 1 |
|  | LAMB3 | laminin, beta 3 |
|  | BMP2 | bone morphogenetic protein 2 |
|  | JUN | jun oncogene |
|  | JUN | jun oncogene |
|  | JUN | jun oncogene |
|  | RAC2 | ras-related C3 botulinum toxin substrate 2 (rho family, small GTP binding protein Rac2) |
|  | FGFR3 | fibroblast growth factor receptor 3 |
|  | FGFR3 | fibroblast growth factor receptor 3 |
|  | ITGA3 | integrin, alpha 3 (antigen CD49C, alpha 3 subunit of VLA-3 receptor) |
|  | WNT10A | wingless-type MMTV integration site family, member 10A |
|  | MYC | v-myc myelocytomatosis viral oncogene homolog (avian) |
|  | MAPK9 | mitogen-activated protein kinase 9 |
|  | MMP2 | matrix metallopeptidase 2 (gelatinase A, 72kDa gelatinase, 72kDa type IV collagenase) |
|  | BAX | BCL2-associated X protein |
|  | BAX | BCL2-associated X protein |
|  | MSH2 | mutS homolog 2, colon cancer, nonpolyposis type 1 (E. coli) |
|  | DAPK1 | death-associated protein kinase 1 |
|  | CKS1B | CDC28 protein kinase regulatory subunit 1B |
|  | MSH6 | mutS homolog 6 (E. coli) |
|  | MSH6 | mutS homolog 6 (E. coli) |
|  | BCR | breakpoint cluster region |
|  | VEGFC | vascular endothelial growth factor C |
|  | TGFB1 | transforming growth factor, beta 1 |
|  | RHOA | ras homolog gene family, member A |
|  | RHOA | ras homolog gene family, member A |
|  | LAMC1 | laminin, gamma 1 (formerly LAMB2) |
|  | SOS1 | son of sevenless homolog 1 (Drosophila) |
|  | FZD3 | frizzled homolog 3 (Drosophila) |
|  | SKP2 | S-phase kinase-associated protein 2 (p45) |
|  | SKP2 | S-phase kinase-associated protein 2 (p45) |
|  | TCEB1 | transcription elongation factor B (SIII), polypeptide 1 (15kDa, elongin C) |
|  | TCEB1 | transcription elongation factor B (SIII), polypeptide 1 (15kDa, elongin C) |
|  | PTK2 | PTK2 protein tyrosine kinase 2 |
|  | PTK2 | PTK2 protein tyrosine kinase 2 |
|  | FOS | FBJ murine osteosarcoma viral oncogene homolog |
|  | PIK3CB | phosphoinositide-3-kinase, catalytic, beta polypeptide |
|  | APPL1 | adaptor protein, phosphotyrosine interaction, PH domain and leucine zipper containing 1 |
|  | APPL1 | adaptor protein, phosphotyrosine interaction, PH domain and leucine zipper containing 1 |
|  | MAP2K2 | mitogen-activated protein kinase kinase 2 |
|  | EGFR | epidermal growth factor receptor (erythroblastic leukemia viral (v-erb-b) oncogene homolog, avian) |
|  | EGFR | epidermal growth factor receptor (erythroblastic leukemia viral (v-erb-b) oncogene homolog, avian) |
|  | EGFR | epidermal growth factor receptor (erythroblastic leukemia viral (v-erb-b) oncogene homolog, avian) |
|  | TCF7L2 | transcription factor 7-like 2 (T-cell specific, HMG-box) |
|  | TCF7L2 | transcription factor 7-like 2 (T-cell specific, HMG-box) |
|  | TCF7L2 | transcription factor 7-like 2 (T-cell specific, HMG-box) |
|  | TCF7L2 | transcription factor 7-like 2 (T-cell specific, HMG-box) |
|  | TCF7L2 | transcription factor 7-like 2 (T-cell specific, HMG-box) |
|  | TCF7L2 | transcription factor 7-like 2 (T-cell specific, HMG-box) |
|  | FAS | Fas (TNF receptor superfamily, member 6) |
|  | FAS | Fas (TNF receptor superfamily, member 6) |
|  | FAS | Fas (TNF receptor superfamily, member 6) |
|  | FAS | Fas (TNF receptor superfamily, member 6) |
|  | GRB2 | growth factor receptor-bound protein 2 |
|  | TPR | translocated promoter region (to activated MET oncogene) |
|  | TPR | translocated promoter region (to activated MET oncogene) |
|  | BIRC5 | baculoviral IAP repeat-containing 5 |
|  | RAD51 | RAD51 homolog (RecA homolog, E. coli) (S. cerevisiae) |
|  | FGFR2 | fibroblast growth factor receptor 2 |
|  | AKT3 | v-akt murine thymoma viral oncogene homolog 3 (protein kinase B, gamma) |
|  | AKT3 | v-akt murine thymoma viral oncogene homolog 3 (protein kinase B, gamma) |
|  | AKT3 | v-akt murine thymoma viral oncogene homolog 3 (protein kinase B, gamma) |
|  | AKT3 | v-akt murine thymoma viral oncogene homolog 3 (protein kinase B, gamma) |
|  | CUL2 | cullin 2 |
|  | CUL2 | cullin 2 |
|  | NFKBIA | nuclear factor of kappa light polypeptide gene enhancer in B-cells inhibitor, alpha |
|  | SHH | sonic hedgehog homolog (Drosophila) |
|  | PML | promyelocytic leukemia |
|  | PML | promyelocytic leukemia |
|  | PML | promyelocytic leukemia |
|  | ITGAV | integrin, alpha V (vitronectin receptor, alpha polypeptide, antigen CD51) |
|  | CDK2 | cyclin-dependent kinase 2 |
|  | CTBP1 | C-terminal binding protein 1 |
|  | CDKN2A | cyclin-dependent kinase inhibitor 2A (melanoma, p16, inhibits CDK4) |
|  | STK4 | serine/threonine kinase 4 |
|  | EGLN1 | egl nine homolog 1 (C. elegans) |
| **KEGG pathway----Ubiquitin mediated proteolysis----04120** | **Gene Symbol** | **Gene Name** |
|  | CUL4A | cullin 4A |
|  | UBE2W | ubiquitin-conjugating enzyme E2W (putative) |
|  | UBE2W | ubiquitin-conjugating enzyme E2W (putative) |
|  | UBE2W | ubiquitin-conjugating enzyme E2W (putative) |
|  | CUL4B | cullin 4B |
|  | CUL4B | cullin 4B |
|  | CUL4B | cullin 4B |
|  | CUL4B | cullin 4B |
|  | XIAP | X-linked inhibitor of apoptosis |
|  | XIAP | X-linked inhibitor of apoptosis |
|  | RCHY1 | ring finger and CHY zinc finger domain containing 1 |
|  | RCHY1 | ring finger and CHY zinc finger domain containing 1 |
|  | CUL1 | cullin 1 |
|  | SAE1 | SUMO1 activating enzyme subunit 1 |
|  | UBE2I | ubiquitin-conjugating enzyme E2I (UBC9 homolog, yeast) |
|  | BIRC2 | baculoviral IAP repeat-containing 2 |
|  | MDM2 | Mdm2 p53 binding protein homolog (mouse) |
|  | SIAH1 | seven in absentia homolog 1 (Drosophila) |
|  | SIAH1 | seven in absentia homolog 1 (Drosophila) |
|  | SIAH1 | seven in absentia homolog 1 (Drosophila) |
|  | UBA2 | ubiquitin-like modifier activating enzyme 2 |
|  | ANAPC11 | anaphase promoting complex subunit 11 |
|  | ANAPC10 | anaphase promoting complex subunit 10 |
|  | UBE2G1 | ubiquitin-conjugating enzyme E2G 1 (UBC7 homolog, yeast) |
|  | UBE2G1 | ubiquitin-conjugating enzyme E2G 1 (UBC7 homolog, yeast) |
|  | UBE2G1 | ubiquitin-conjugating enzyme E2G 1 (UBC7 homolog, yeast) |
|  | HERC4 | hect domain and RLD 4 |
|  | UBE2D1 | ubiquitin-conjugating enzyme E2D 1 (UBC4/5 homolog, yeast) |
|  | UBE2D1 | ubiquitin-conjugating enzyme E2D 1 (UBC4/5 homolog, yeast) |
|  | UBE2F | ubiquitin-conjugating enzyme E2F (putative) |
|  | KLHL9 | kelch-like 9 (Drosophila) |
|  | BIRC3 | baculoviral IAP repeat-containing 3 |
|  | RBX1 | ring-box 1 |
|  | ITCH | itchy E3 ubiquitin protein ligase homolog (mouse) |
|  | ITCH | itchy E3 ubiquitin protein ligase homolog (mouse) |
|  | ITCH | itchy E3 ubiquitin protein ligase homolog (mouse) |
|  | CDC20 | cell division cycle 20 homolog (S. cerevisiae) |
|  | CBLB | Cas-Br-M (murine) ecotropic retroviral transforming sequence b |
|  | BRCA1 | breast cancer 1, early onset |
|  | CDC23 | cell division cycle 23 homolog (S. cerevisiae) |
|  | SOCS3 | suppressor of cytokine signaling 3 |
|  | PIAS3 | protein inhibitor of activated STAT, 3 |
|  | UBE2B | ubiquitin-conjugating enzyme E2B (RAD6 homolog) |
|  | UBE2B | ubiquitin-conjugating enzyme E2B (RAD6 homolog) |
|  | UBE2B | ubiquitin-conjugating enzyme E2B (RAD6 homolog) |
|  | SKP1 | S-phase kinase-associated protein 1 |
|  | SKP1 | S-phase kinase-associated protein 1 |
|  | TRIM32 | tripartite motif-containing 32 |
|  | UBE2E3 | ubiquitin-conjugating enzyme E2E 3 (UBC4/5 homolog, yeast) |
|  | SMURF1 | SMAD specific E3 ubiquitin protein ligase 1 |
|  | UBE2N | ubiquitin-conjugating enzyme E2N (UBC13 homolog, yeast) |
|  | UBE2N | ubiquitin-conjugating enzyme E2N (UBC13 homolog, yeast) |
|  | MID1 | midline 1 (Opitz/BBB syndrome) |
|  | UBE2D3 | ubiquitin-conjugating enzyme E2D 3 (UBC4/5 homolog, yeast) |
|  | UBE2D3 | ubiquitin-conjugating enzyme E2D 3 (UBC4/5 homolog, yeast) |
|  | FBXO4 | F-box protein 4 |
|  | NEDD4 | neural precursor cell expressed, developmentally down-regulated 4 |
|  | CUL5 | cullin 5 |
|  | UBE2Q2 | ubiquitin-conjugating enzyme E2Q family member 2 |
|  | UBE3A | ubiquitin protein ligase E3A |
|  | UBA7 | ubiquitin-like modifier activating enzyme 7 |
|  | UBA7 | ubiquitin-like modifier activating enzyme 7 |
|  | UBE2J1 | ubiquitin-conjugating enzyme E2, J1 (UBC6 homolog, yeast) |
|  | UBE2J1 | ubiquitin-conjugating enzyme E2, J1 (UBC6 homolog, yeast) |
|  | UBE2J1 | ubiquitin-conjugating enzyme E2, J1 (UBC6 homolog, yeast) |
|  | UBE2J1 | ubiquitin-conjugating enzyme E2, J1 (UBC6 homolog, yeast) |
|  | WWP1 | WW domain containing E3 ubiquitin protein ligase 1 |
|  | UBE3B | ubiquitin protein ligase E3B |
|  | UBE3C | ubiquitin protein ligase E3C |
|  | UBE3C | ubiquitin protein ligase E3C |
|  | UBE3C | ubiquitin protein ligase E3C |
|  | UBE2M | ubiquitin-conjugating enzyme E2M (UBC12 homolog, yeast) |
|  | UBE2G2 | ubiquitin-conjugating enzyme E2G 2 (UBC7 homolog, yeast) |
|  | DDB2 | damage-specific DNA binding protein 2, 48kDa |
|  | UBE2L3 | ubiquitin-conjugating enzyme E2L 3 |
|  | UBE2L3 | ubiquitin-conjugating enzyme E2L 3 |
|  | UBE2L3 | ubiquitin-conjugating enzyme E2L 3 |
|  | RNF7 | ring finger protein 7 |
|  | SKP2 | S-phase kinase-associated protein 2 (p45) |
|  | SKP2 | S-phase kinase-associated protein 2 (p45) |
|  | UBE2A | ubiquitin-conjugating enzyme E2A (RAD6 homolog) |
|  | UBE2A | ubiquitin-conjugating enzyme E2A (RAD6 homolog) |
|  | UBE2H | ubiquitin-conjugating enzyme E2H (UBC8 homolog, yeast) |
|  | TCEB1 | transcription elongation factor B (SIII), polypeptide 1 (15kDa, elongin C) |
|  | TCEB1 | transcription elongation factor B (SIII), polypeptide 1 (15kDa, elongin C) |
|  | UBE4B | ubiquitination factor E4B (UFD2 homolog, yeast) |
|  | SMURF2 | SMAD specific E3 ubiquitin protein ligase 2 |
|  | SMURF2 | SMAD specific E3 ubiquitin protein ligase 2 |
|  | TRIM37 | tripartite motif-containing 37 |
|  | UBE2K | ubiquitin-conjugating enzyme E2K (UBC1 homolog, yeast) |
|  | CDC26 | cell division cycle 26 homolog (S. cerevisiae) |
|  | UBA6 | ubiquitin-like modifier activating enzyme 6 |
|  | UBA6 | ubiquitin-like modifier activating enzyme 6 |
|  | UBE2E1 | ubiquitin-conjugating enzyme E2E 1 (UBC4/5 homolog, yeast) |
|  | RFWD2 | ring finger and WD repeat domain 2 |
|  | CDC27 | cell division cycle 27 homolog (S. cerevisiae) |
|  | CDC27 | cell division cycle 27 homolog (S. cerevisiae) |
|  | CUL2 | cullin 2 |
|  | CUL2 | cullin 2 |
|  | UBE2D2 | ubiquitin-conjugating enzyme E2D 2 (UBC4/5 homolog, yeast) |
|  | PML | promyelocytic leukemia |
|  | PML | promyelocytic leukemia |
|  | PML | promyelocytic leukemia |
|  | UBE2S | ubiquitin-conjugating enzyme E2S |
|  | UBE2C | ubiquitin-conjugating enzyme E2C |
|  | BTRC | beta-transducin repeat containing |
| **KEGG pathway----Focal adhesion----04510** | **Gene Symbol** | **Gene Name** |
|  | MYL9 | myosin, light chain 9, regulatory |
|  | MAPK8 | mitogen-activated protein kinase 8 |
|  | DOCK1 | dedicator of cytokinesis 1 |
|  | DOCK1 | dedicator of cytokinesis 1 |
|  | MAP2K1 | mitogen-activated protein kinase kinase 1 |
|  | XIAP | X-linked inhibitor of apoptosis |
|  | XIAP | X-linked inhibitor of apoptosis |
|  | COL11A1 | collagen, type XI, alpha 1 |
|  | COL11A1 | collagen, type XI, alpha 1 |
|  | PRKCA | protein kinase C, alpha |
|  | PDGFRA | platelet-derived growth factor receptor, alpha polypeptide |
|  | TNC | tenascin C |
|  | BIRC2 | baculoviral IAP repeat-containing 2 |
|  | SHC2 | SHC (Src homology 2 domain containing) transforming protein 2 |
|  | FLNB | filamin B, beta |
|  | CRKL | v-crk sarcoma virus CT10 oncogene homolog (avian)-like |
|  | CCND1 | cyclin D1 |
|  | CCND1 | cyclin D1 |
|  | ZYX | zyxin |
|  | RAP1A | RAP1A, member of RAS oncogene family |
|  | LAMA3 | laminin, alpha 3 |
|  | SHC1 | SHC (Src homology 2 domain containing) transforming protein 1 |
|  | COL5A1 | collagen, type V, alpha 1 |
|  | COL5A1 | collagen, type V, alpha 1 |
|  | COL5A1 | collagen, type V, alpha 1 |
|  | PIK3CA | phosphoinositide-3-kinase, catalytic, alpha polypeptide |
|  | COL4A1 | collagen, type IV, alpha 1 |
|  | ITGB6 | integrin, beta 6 |
|  | PDGFRB | platelet-derived growth factor receptor, beta polypeptide |
|  | LAMA4 | laminin, alpha 4 |
|  | LAMA4 | laminin, alpha 4 |
|  | COL6A2 | collagen, type VI, alpha 2 |
|  | COL6A2 | collagen, type VI, alpha 2 |
|  | COL5A2 | collagen, type V, alpha 2 |
|  | BIRC3 | baculoviral IAP repeat-containing 3 |
|  | COL1A1 | collagen, type I, alpha 1 |
|  | COL1A1 | collagen, type I, alpha 1 |
|  | ARHGAP5 | Rho GTPase activating protein 5 |
|  | ACTN1 | actinin, alpha 1 |
|  | ACTN1 | actinin, alpha 1 |
|  | ACTN1 | actinin, alpha 1 |
|  | CAV1 | caveolin 1, caveolae protein, 22kDa |
|  | CAV1 | caveolin 1, caveolae protein, 22kDa |
|  | FLNA | filamin A, alpha |
|  | FLNA | filamin A, alpha |
|  | PARVA | parvin, alpha |
|  | PARVA | parvin, alpha |
|  | VEGFA | vascular endothelial growth factor A |
|  | VEGFA | vascular endothelial growth factor A |
|  | VEGFA | vascular endothelial growth factor A |
|  | FYN | FYN oncogene related to SRC, FGR, YES |
|  | ITGA2 | integrin, alpha 2 (CD49B, alpha 2 subunit of VLA-2 receptor) |
|  | MYLK | myosin light chain kinase |
|  | LAMB1 | laminin, beta 1 |
|  | LAMB1 | laminin, beta 1 |
|  | ITGB1 | integrin, beta 1 (fibronectin receptor, beta polypeptide, antigen CD29 includes MDF2, MSK12) |
|  | ITGB1 | integrin, beta 1 (fibronectin receptor, beta polypeptide, antigen CD29 includes MDF2, MSK12) |
|  | PPP1CB | protein phosphatase 1, catalytic subunit, beta isoform |
|  | PPP1CB | protein phosphatase 1, catalytic subunit, beta isoform |
|  | ROCK1 | Rho-associated, coiled-coil containing protein kinase 1 |
|  | ROCK1 | Rho-associated, coiled-coil containing protein kinase 1 |
|  | MAPK10 | mitogen-activated protein kinase 10 |
|  | MAPK1 | mitogen-activated protein kinase 1 |
|  | MAPK1 | mitogen-activated protein kinase 1 |
|  | LAMA5 | laminin, alpha 5 |
|  | LAMC2 | laminin, gamma 2 |
|  | BRAF | v-raf murine sarcoma viral oncogene homolog B1 |
|  | FN1 | fibronectin 1 |
|  | FN1 | fibronectin 1 |
|  | FN1 | fibronectin 1 |
|  | FN1 | fibronectin 1 |
|  | PPP1R12A | protein phosphatase 1, regulatory (inhibitor) subunit 12A |
|  | ITGB5 | integrin, beta 5 |
|  | ITGB5 | integrin, beta 5 |
|  | SRC | v-src sarcoma (Schmidt-Ruppin A-2) viral oncogene homolog (avian) |
|  | COL3A1 | collagen, type III, alpha 1 |
|  | COL3A1 | collagen, type III, alpha 1 |
|  | COL3A1 | collagen, type III, alpha 1 |
|  | PPP1CC | protein phosphatase 1, catalytic subunit, gamma isoform |
|  | COL6A3 | collagen, type VI, alpha 3 |
|  | LAMB3 | laminin, beta 3 |
|  | THBS1 | thrombospondin 1 |
|  | THBS1 | thrombospondin 1 |
|  | THBS1 | thrombospondin 1 |
|  | JUN | jun oncogene |
|  | JUN | jun oncogene |
|  | JUN | jun oncogene |
|  | CAPN2 | calpain 2, (m/II) large subunit |
|  | RAC2 | ras-related C3 botulinum toxin substrate 2 (rho family, small GTP binding protein Rac2) |
|  | SPP1 | secreted phosphoprotein 1 |
|  | ITGA3 | integrin, alpha 3 (antigen CD49C, alpha 3 subunit of VLA-3 receptor) |
|  | ITGB8 | integrin, beta 8 |
|  | COL6A1 | collagen, type VI, alpha 1 |
|  | MAPK9 | mitogen-activated protein kinase 9 |
|  | PAK2 | p21 protein (Cdc42/Rac)-activated kinase 2 |
|  | PAK2 | p21 protein (Cdc42/Rac)-activated kinase 2 |
|  | PAK2 | p21 protein (Cdc42/Rac)-activated kinase 2 |
|  | CCND2 | cyclin D2 |
|  | ROCK2 | Rho-associated, coiled-coil containing protein kinase 2 |
|  | CAV2 | caveolin 2 |
|  | CAV2 | caveolin 2 |
|  | VEGFC | vascular endothelial growth factor C |
|  | PDPK1 | 3-phosphoinositide dependent protein kinase-1 |
|  | PAK1 | p21 protein (Cdc42/Rac)-activated kinase 1 |
|  | PAK1 | p21 protein (Cdc42/Rac)-activated kinase 1 |
|  | VAV3 | vav 3 guanine nucleotide exchange factor |
|  | PDGFC | platelet derived growth factor C |
|  | PXN | paxillin |
|  | RHOA | ras homolog gene family, member A |
|  | RHOA | ras homolog gene family, member A |
|  | LAMC1 | laminin, gamma 1 (formerly LAMB2) |
|  | THBS2 | thrombospondin 2 |
|  | SOS1 | son of sevenless homolog 1 (Drosophila) |
|  | ITGA5 | integrin, alpha 5 (fibronectin receptor, alpha polypeptide) |
|  | PTK2 | PTK2 protein tyrosine kinase 2 |
|  | PTK2 | PTK2 protein tyrosine kinase 2 |
|  | PIK3CB | phosphoinositide-3-kinase, catalytic, beta polypeptide |
|  | EGFR | epidermal growth factor receptor (erythroblastic leukemia viral (v-erb-b) oncogene homolog, avian) |
|  | EGFR | epidermal growth factor receptor (erythroblastic leukemia viral (v-erb-b) oncogene homolog, avian) |
|  | EGFR | epidermal growth factor receptor (erythroblastic leukemia viral (v-erb-b) oncogene homolog, avian) |
|  | GRB2 | growth factor receptor-bound protein 2 |
|  | AKT3 | v-akt murine thymoma viral oncogene homolog 3 (protein kinase B, gamma) |
|  | AKT3 | v-akt murine thymoma viral oncogene homolog 3 (protein kinase B, gamma) |
|  | AKT3 | v-akt murine thymoma viral oncogene homolog 3 (protein kinase B, gamma) |
|  | AKT3 | v-akt murine thymoma viral oncogene homolog 3 (protein kinase B, gamma) |
|  | VWF | von Willebrand factor |
|  | ITGAV | integrin, alpha V (vitronectin receptor, alpha polypeptide, antigen CD51) |
|  | COL1A2 | collagen, type I, alpha 2 |
|  | COL1A2 | collagen, type I, alpha 2 |
|  | COL1A2 | collagen, type I, alpha 2 |
|  | ITGB4 | integrin, beta 4 |
| **KEGG pathway----Pyrimidine metabolism----00240** | **Gene Symbol** | **Gene Name** |
|  | POLR2F | polymerase (RNA) II (DNA directed) polypeptide F |
|  | POLR2K | polymerase (RNA) II (DNA directed) polypeptide K, 7.0kDa |
|  | POLR2K | polymerase (RNA) II (DNA directed) polypeptide K, 7.0kDa |
|  | UCK2 | uridine-cytidine kinase 2 |
|  | RRM1 | ribonucleotide reductase M1 |
|  | RRM1 | ribonucleotide reductase M1 |
|  | POLD1 | polymerase (DNA directed), delta 1, catalytic subunit 125kDa |
|  | POLR3K | polymerase (RNA) III (DNA directed) polypeptide K, 12.3 kDa |
|  | TK2 | thymidine kinase 2, mitochondrial |
|  | CAD | carbamoyl-phosphate synthetase 2, aspartate transcarbamylase, and dihydroorotase |
|  | TYMP | thymidine phosphorylase |
|  | ENTPD4 | ectonucleoside triphosphate diphosphohydrolase 4 |
|  | DUT | deoxyuridine triphosphatase |
|  | CTPS2 | CTP synthase II |
|  | CTPS | CTP synthase |
|  | UPRT | uracil phosphoribosyltransferase (FUR1) homolog (S. cerevisiae) |
|  | DPYD | dihydropyrimidine dehydrogenase |
|  | POLE3 | polymerase (DNA directed), epsilon 3 (p17 subunit) |
|  | CMPK1 | cytidine monophosphate (UMP-CMP) kinase 1, cytosolic |
|  | CMPK1 | cytidine monophosphate (UMP-CMP) kinase 1, cytosolic |
|  | POLR1B | polymerase (RNA) I polypeptide B, 128kDa |
|  | POLR3C | polymerase (RNA) III (DNA directed) polypeptide C (62kD) |
|  | ZNRD1 | zinc ribbon domain containing 1 |
|  | ZNRD1 | zinc ribbon domain containing 1 |
|  | TXNRD2 | thioredoxin reductase 2 |
|  | NT5E | 5'-nucleotidase, ecto (CD73) |
|  | POLR3F | polymerase (RNA) III (DNA directed) polypeptide F, 39 kDa |
|  | TXNRD1 | thioredoxin reductase 1 |
|  | DTYMK | deoxythymidylate kinase (thymidylate kinase) |
|  | POLR2I | polymerase (RNA) II (DNA directed) polypeptide I, 14.5kDa |
|  | TK1 | thymidine kinase 1, soluble |
|  | TK1 | thymidine kinase 1, soluble |
|  | POLR2G | polymerase (RNA) II (DNA directed) polypeptide G |
|  | PRIM2 | primase, DNA, polypeptide 2 (58kDa) |
|  | PRIM2 | primase, DNA, polypeptide 2 (58kDa) |
|  | PRIM2 | primase, DNA, polypeptide 2 (58kDa) |
|  | NME6 | non-metastatic cells 6, protein expressed in (nucleoside-diphosphate kinase) |
|  | NP | nucleoside phosphorylase |
|  | POLA1 | polymerase (DNA directed), alpha 1, catalytic subunit |
|  | POLR2D | polymerase (RNA) II (DNA directed) polypeptide D |
|  | RRM2 | ribonucleotide reductase M2 |
|  | DCTD | dCMP deaminase |
|  | NUDT2 | nudix (nucleoside diphosphate linked moiety X)-type motif 2 |
|  | POLD3 | polymerase (DNA-directed), delta 3, accessory subunit |
|  | POLE4 | polymerase (DNA-directed), epsilon 4 (p12 subunit) |
|  | POLE4 | polymerase (DNA-directed), epsilon 4 (p12 subunit) |
|  | PRIM1 | primase, DNA, polypeptide 1 (49kDa) |
|  | POLR2C | polymerase (RNA) II (DNA directed) polypeptide C, 33kDa |
|  | NT5C2 | 5'-nucleotidase, cytosolic II |
|  | NME1 | non-metastatic cells 1, protein (NM23A) expressed in |
|  | NME7 | non-metastatic cells 7, protein expressed in (nucleoside-diphosphate kinase) |
|  | POLE2 | polymerase (DNA directed), epsilon 2 (p59 subunit) |
|  | POLR1D | polymerase (RNA) I polypeptide D, 16kDa |
|  | DCK | deoxycytidine kinase |
|  | POLR2E | polymerase (RNA) II (DNA directed) polypeptide E, 25kDa |
|  | CANT1 | calcium activated nucleotidase 1 |
|  | CANT1 | calcium activated nucleotidase 1 |
|  | ENTPD3 | ectonucleoside triphosphate diphosphohydrolase 3 |
|  | TYMS | thymidylate synthetase |
|  | TYMS | thymidylate synthetase |
|  | UMPS | uridine monophosphate synthetase |
|  | UMPS | uridine monophosphate synthetase |
|  | POLR1C | polymerase (RNA) I polypeptide C, 30kDa |
|  | POLR1C | polymerase (RNA) I polypeptide C, 30kDa |
|  | PNPT1 | polyribonucleotide nucleotidyltransferase 1 |
|  | POLD2 | polymerase (DNA directed), delta 2, regulatory subunit 50kDa |
| **KEGG pathway----Endocytosis----04144** | **Gene Symbol** | **Gene Name** |
|  | PARD6G | par-6 partitioning defective 6 homolog gamma (C. elegans) |
|  | CHMP5 | chromatin modifying protein 5 |
|  | CHMP5 | chromatin modifying protein 5 |
|  | LDLR | low density lipoprotein receptor |
|  | PDGFRA | platelet-derived growth factor receptor, alpha polypeptide |
|  | ERBB4 | v-erb-a erythroblastic leukemia viral oncogene homolog 4 (avian) |
|  | HSPA1A | heat shock 70kDa protein 1A |
|  | EEA1 | early endosome antigen 1 |
|  | EEA1 | early endosome antigen 1 |
|  | SH3KBP1 | SH3-domain kinase binding protein 1 |
|  | SH3KBP1 | SH3-domain kinase binding protein 1 |
|  | RAB11FIP3 | RAB11 family interacting protein 3 (class II) |
|  | MDM2 | Mdm2 p53 binding protein homolog (mouse) |
|  | ACAP2 | ArfGAP with coiled-coil, ankyrin repeat and PH domains 2 |
|  | ACAP2 | ArfGAP with coiled-coil, ankyrin repeat and PH domains 2 |
|  | RAB4A | RAB4A, member RAS oncogene family |
|  | DNM2 | dynamin 2 |
|  | AP2B1 | adaptor-related protein complex 2, beta 1 subunit |
|  | CLTB | clathrin, light chain (Lcb) |
|  | CLTB | clathrin, light chain (Lcb) |
|  | CLTB | clathrin, light chain (Lcb) |
|  | F2R | coagulation factor II (thrombin) receptor |
|  | CHMP1B | chromatin modifying protein 1B |
|  | STAMBP | STAM binding protein |
|  | ACVR1B | activin A receptor, type IB |
|  | ITCH | itchy E3 ubiquitin protein ligase homolog (mouse) |
|  | ITCH | itchy E3 ubiquitin protein ligase homolog (mouse) |
|  | ITCH | itchy E3 ubiquitin protein ligase homolog (mouse) |
|  | HSPA8 | heat shock 70kDa protein 8 |
|  | HSPA8 | heat shock 70kDa protein 8 |
|  | HSPA8 | heat shock 70kDa protein 8 |
|  | CBLB | Cas-Br-M (murine) ecotropic retroviral transforming sequence b |
|  | RAB5A | RAB5A, member RAS oncogene family |
|  | CHMP2B | chromatin modifying protein 2B |
|  | CHMP2B | chromatin modifying protein 2B |
|  | VPS36 | vacuolar protein sorting 36 homolog (S. cerevisiae) |
|  | RAB22A | RAB22A, member RAS oncogene family |
|  | RAB22A | RAB22A, member RAS oncogene family |
|  | HSPA6 | heat shock 70kDa protein 6 (HSP70B') |
|  | EPN2 | epsin 2 |
|  | ZFYVE20 | zinc finger, FYVE domain containing 20 |
|  | CHMP4A | chromatin modifying protein 4A |
|  | DNM1L | dynamin 1-like |
|  | CLTC | clathrin, heavy chain (Hc) |
|  | RAB11FIP1 | RAB11 family interacting protein 1 (class I) |
|  | RAB11FIP1 | RAB11 family interacting protein 1 (class I) |
|  | PRKCZ | protein kinase C, zeta |
|  | SH3GLB2 | SH3-domain GRB2-like endophilin B2 |
|  | SH3GLB2 | SH3-domain GRB2-like endophilin B2 |
|  | CHMP4B | chromatin modifying protein 4B |
|  | ARF6 | ADP-ribosylation factor 6 |
|  | ARF6 | ADP-ribosylation factor 6 |
|  | SMURF1 | SMAD specific E3 ubiquitin protein ligase 1 |
|  | AP2S1 | adaptor-related protein complex 2, sigma 1 subunit |
|  | AP2S1 | adaptor-related protein complex 2, sigma 1 subunit |
|  | CHMP4C | chromatin modifying protein 4C |
|  | GRK5 | G protein-coupled receptor kinase 5 |
|  | ARAP3 | ArfGAP with RhoGAP domain, ankyrin repeat and PH domain 3 |
|  | AP2M1 | adaptor-related protein complex 2, mu 1 subunit |
|  | SMAP1 | small ArfGAP 1 |
|  | VTA1 | Vps20-associated 1 homolog (S. cerevisiae) |
|  | VTA1 | Vps20-associated 1 homolog (S. cerevisiae) |
|  | VTA1 | Vps20-associated 1 homolog (S. cerevisiae) |
|  | PSD3 | pleckstrin and Sec7 domain containing 3 |
|  | SNF8 | SNF8, ESCRT-II complex subunit, homolog (S. cerevisiae) |
|  | TGFBR1 | transforming growth factor, beta receptor 1 |
|  | TGFBR1 | transforming growth factor, beta receptor 1 |
|  | SRC | v-src sarcoma (Schmidt-Ruppin A-2) viral oncogene homolog (avian) |
|  | VPS25 | vacuolar protein sorting 25 homolog (S. cerevisiae) |
|  | RAB11A | RAB11A, member RAS oncogene family |
|  | NEDD4 | neural precursor cell expressed, developmentally down-regulated 4 |
|  | TFRC | transferrin receptor (p90, CD71) |
|  | TFRC | transferrin receptor (p90, CD71) |
|  | FGFR3 | fibroblast growth factor receptor 3 |
|  | FGFR3 | fibroblast growth factor receptor 3 |
|  | DAB2 | disabled homolog 2, mitogen-responsive phosphoprotein (Drosophila) |
|  | DAB2 | disabled homolog 2, mitogen-responsive phosphoprotein (Drosophila) |
|  | DAB2 | disabled homolog 2, mitogen-responsive phosphoprotein (Drosophila) |
|  | DAB2 | disabled homolog 2, mitogen-responsive phosphoprotein (Drosophila) |
|  | ASAP1 | ArfGAP with SH3 domain, ankyrin repeat and PH domain 1 |
|  | ASAP1 | ArfGAP with SH3 domain, ankyrin repeat and PH domain 1 |
|  | ASAP1 | ArfGAP with SH3 domain, ankyrin repeat and PH domain 1 |
|  | EHD2 | EH-domain containing 2 |
|  | WWP1 | WW domain containing E3 ubiquitin protein ligase 1 |
|  | ERBB3 | v-erb-b2 erythroblastic leukemia viral oncogene homolog 3 (avian) |
|  | ERBB3 | v-erb-b2 erythroblastic leukemia viral oncogene homolog 3 (avian) |
|  | ARAP2 | ArfGAP with RhoGAP domain, ankyrin repeat and PH domain 2 |
|  | VPS37A | vacuolar protein sorting 37 homolog A (S. cerevisiae) |
|  | IL2RG | interleukin 2 receptor, gamma (severe combined immunodeficiency) |
|  | SMURF2 | SMAD specific E3 ubiquitin protein ligase 2 |
|  | SMURF2 | SMAD specific E3 ubiquitin protein ligase 2 |
|  | VPS24 | vacuolar protein sorting 24 homolog (S. cerevisiae) |
|  | VPS24 | vacuolar protein sorting 24 homolog (S. cerevisiae) |
|  | EGFR | epidermal growth factor receptor (erythroblastic leukemia viral (v-erb-b) oncogene homolog, avian) |
|  | EGFR | epidermal growth factor receptor (erythroblastic leukemia viral (v-erb-b) oncogene homolog, avian) |
|  | EGFR | epidermal growth factor receptor (erythroblastic leukemia viral (v-erb-b) oncogene homolog, avian) |
|  | SH3GLB1 | SH3-domain GRB2-like endophilin B1 |
|  | SH3GLB1 | SH3-domain GRB2-like endophilin B1 |
|  | HSPA1B | heat shock 70kDa protein 1B |
|  | EPN3 | epsin 3 |
|  | CXCR4 | chemokine (C-X-C motif) receptor 4 |
|  | CXCR4 | chemokine (C-X-C motif) receptor 4 |
|  | FGFR2 | fibroblast growth factor receptor 2 |
|  | STAM2 | signal transducing adaptor molecule (SH3 domain and ITAM motif) 2 |
|  | RAB11FIP2 | RAB11 family interacting protein 2 (class I) |
|  | VPS37B | vacuolar protein sorting 37 homolog B (S. cerevisiae) |
|  | RAB31 | RAB31, member RAS oncogene family |
|  | RAB31 | RAB31, member RAS oncogene family |
|  | RAB31 | RAB31, member RAS oncogene family |
|  | EHD4 | EH-domain containing 4 |
|  | EHD4 | EH-domain containing 4 |
|  | RABEP1 | rabaptin, RAB GTPase binding effector protein 1 |
|  | RAB5C | RAB5C, member RAS oncogene family |
| **KEGG pathway----Lysosome----04142** | **Gene Symbol** | **Gene Name** |
|  | LAMP1 | lysosomal-associated membrane protein 1 |
|  | GAA | glucosidase, alpha; acid |
|  | GGA1 | golgi associated, gamma adaptin ear containing, ARF binding protein 1 |
|  | CTSB | cathepsin B |
|  | CTSB | cathepsin B |
|  | HEXB | hexosaminidase B (beta polypeptide) |
|  | AP3M2 | adaptor-related protein complex 3, mu 2 subunit |
|  | AP3M2 | adaptor-related protein complex 3, mu 2 subunit |
|  | ASAH1 | N-acylsphingosine amidohydrolase (acid ceramidase) 1 |
|  | AP3D1 | adaptor-related protein complex 3, delta 1 subunit |
|  | CTSZ | cathepsin Z |
|  | GGA2 | golgi associated, gamma adaptin ear containing, ARF binding protein 2 |
|  | M6PR | mannose-6-phosphate receptor (cation dependent) |
|  | M6PR | mannose-6-phosphate receptor (cation dependent) |
|  | TPP1 | tripeptidyl peptidase I |
|  | GNS | glucosamine (N-acetyl)-6-sulfatase |
|  | ATP6V0B | ATPase, H+ transporting, lysosomal 21kDa, V0 subunit b |
|  | AP1M2 | adaptor-related protein complex 1, mu 2 subunit |
|  | CD164 | CD164 molecule, sialomucin |
|  | CD164 | CD164 molecule, sialomucin |
|  | ENTPD4 | ectonucleoside triphosphate diphosphohydrolase 4 |
|  | CTSS | cathepsin S |
|  | CTSS | cathepsin S |
|  | CTSC | cathepsin C |
|  | CTSC | cathepsin C |
|  | CTSC | cathepsin C |
|  | LGMN | legumain |
|  | CLTB | clathrin, light chain (Lcb) |
|  | CLTB | clathrin, light chain (Lcb) |
|  | CLTB | clathrin, light chain (Lcb) |
|  | CTSH | cathepsin H |
|  | NAGA | N-acetylgalactosaminidase, alpha- |
|  | NAGA | N-acetylgalactosaminidase, alpha- |
|  | AGA | aspartylglucosaminidase |
|  | AGA | aspartylglucosaminidase |
|  | AGA | aspartylglucosaminidase |
|  | IDUA | iduronidase, alpha-L- |
|  | GM2A | GM2 ganglioside activator |
|  | GM2A | GM2 ganglioside activator |
|  | LAMP3 | lysosomal-associated membrane protein 3 |
|  | LAMP2 | lysosomal-associated membrane protein 2 |
|  | LAMP2 | lysosomal-associated membrane protein 2 |
|  | LAMP2 | lysosomal-associated membrane protein 2 |
|  | LAMP2 | lysosomal-associated membrane protein 2 |
|  | PPT1 | palmitoyl-protein thioesterase 1 |
|  | AP1S2 | adaptor-related protein complex 1, sigma 2 subunit |
|  | CTSE | cathepsin E |
|  | GBA | glucosidase, beta; acid (includes glucosylceramidase) |
|  | AP3B1 | adaptor-related protein complex 3, beta 1 subunit |
|  | SLC11A2 | solute carrier family 11 (proton-coupled divalent metal ion transporters), member 2 |
|  | SLC11A2 | solute carrier family 11 (proton-coupled divalent metal ion transporters), member 2 |
|  | SLC17A5 | solute carrier family 17 (anion/sugar transporter), member 5 |
|  | CTSK | cathepsin K |
|  | CLN5 | ceroid-lipofuscinosis, neuronal 5 |
|  | CLN5 | ceroid-lipofuscinosis, neuronal 5 |
|  | LIPA | lipase A, lysosomal acid, cholesterol esterase |
|  | NPC1 | Niemann-Pick disease, type C1 |
|  | AP3M1 | adaptor-related protein complex 3, mu 1 subunit |
|  | NEU1 | sialidase 1 (lysosomal sialidase) |
|  | FUCA1 | fucosidase, alpha-L- 1, tissue |
|  | IDS | iduronate 2-sulfatase |
|  | CTSL1 | cathepsin L1 |
|  | SCARB2 | scavenger receptor class B, member 2 |
|  | SGSH | N-sulfoglucosamine sulfohydrolase |
|  | TCIRG1 | T-cell, immune regulator 1, ATPase, H+ transporting, lysosomal V0 subunit A3 |
|  | CTSO | cathepsin O |
|  | CLTC | clathrin, heavy chain (Hc) |
|  | CTSL2 | cathepsin L2 |
|  | LAPTM5 | lysosomal protein transmembrane 5 |
|  | LAPTM5 | lysosomal protein transmembrane 5 |
|  | LAPTM4B | lysosomal protein transmembrane 4 beta |
|  | LAPTM4B | lysosomal protein transmembrane 4 beta |
|  | LAPTM4B | lysosomal protein transmembrane 4 beta |
|  | GLB1 | galactosidase, beta 1 |
|  | CTSA | cathepsin A |
|  | PSAP | prosaposin |
|  | GLA | galactosidase, alpha |
|  | ACP5 | acid phosphatase 5, tartrate resistant |
| **KEGG pathway----DNA replication----03030** | **Gene Symbol** | **Gene Name** |
|  | RFC4 | replication factor C (activator 1) 4, 37kDa |
|  | RFC3 | replication factor C (activator 1) 3, 38kDa |
|  | MCM6 | minichromosome maintenance complex component 6 |
|  | PRIM2 | primase, DNA, polypeptide 2 (58kDa) |
|  | PRIM2 | primase, DNA, polypeptide 2 (58kDa) |
|  | PRIM2 | primase, DNA, polypeptide 2 (58kDa) |
|  | POLA1 | polymerase (DNA directed), alpha 1, catalytic subunit |
|  | MCM2 | minichromosome maintenance complex component 2 |
|  | RNASEH2B | ribonuclease H2, subunit B |
|  | POLD3 | polymerase (DNA-directed), delta 3, accessory subunit |
|  | POLD1 | polymerase (DNA directed), delta 1, catalytic subunit 125kDa |
|  | MCM7 | minichromosome maintenance complex component 7 |
|  | RFC2 | replication factor C (activator 1) 2, 40kDa |
|  | RFC2 | replication factor C (activator 1) 2, 40kDa |
|  | RPA3 | replication protein A3, 14kDa |
|  | POLE4 | polymerase (DNA-directed), epsilon 4 (p12 subunit) |
|  | POLE4 | polymerase (DNA-directed), epsilon 4 (p12 subunit) |
|  | RNASEH1 | ribonuclease H1 |
|  | RPA1 | replication protein A1, 70kDa |
|  | PRIM1 | primase, DNA, polypeptide 1 (49kDa) |
|  | RPA2 | replication protein A2, 32kDa |
|  | MCM4 | minichromosome maintenance complex component 4 |
|  | MCM4 | minichromosome maintenance complex component 4 |
|  | MCM4 | minichromosome maintenance complex component 4 |
|  | PCNA | proliferating cell nuclear antigen |
|  | POLE2 | polymerase (DNA directed), epsilon 2 (p59 subunit) |
|  | RFC5 | replication factor C (activator 1) 5, 36.5kDa |
|  | RFC5 | replication factor C (activator 1) 5, 36.5kDa |
|  | MCM5 | minichromosome maintenance complex component 5 |
|  | MCM5 | minichromosome maintenance complex component 5 |
|  | DNA2 | DNA replication helicase 2 homolog (yeast) |
|  | SSBP1 | single-stranded DNA binding protein 1 |
|  | SSBP1 | single-stranded DNA binding protein 1 |
|  | POLE3 | polymerase (DNA directed), epsilon 3 (p17 subunit) |
|  | RFC1 | replication factor C (activator 1) 1, 145kDa |
|  | RFC1 | replication factor C (activator 1) 1, 145kDa |
|  | RNASEH2A | ribonuclease H2, subunit A |
|  | POLD2 | polymerase (DNA directed), delta 2, regulatory subunit 50kDa |
|  | FEN1 | flap structure-specific endonuclease 1 |
|  | FEN1 | flap structure-specific endonuclease 1 |
|  | MCM3 | minichromosome maintenance complex component 3 |
| **KEGG pathway----MAPK signaling pathway----04010** | **Gene Symbol** | **Gene Name** |
|  | MAPK8 | mitogen-activated protein kinase 8 |
|  | DUSP6 | dual specificity phosphatase 6 |
|  | DUSP6 | dual specificity phosphatase 6 |
|  | DUSP6 | dual specificity phosphatase 6 |
|  | NF1 | neurofibromin 1 |
|  | MAP2K1 | mitogen-activated protein kinase kinase 1 |
|  | NLK | nemo-like kinase |
|  | PRKCA | protein kinase C, alpha |
|  | PPM1B | protein phosphatase 1B (formerly 2C), magnesium-dependent, beta isoform |
|  | PDGFRA | platelet-derived growth factor receptor, alpha polypeptide |
|  | STMN1 | stathmin 1 |
|  | DUSP14 | dual specificity phosphatase 14 |
|  | HSPA1A | heat shock 70kDa protein 1A |
|  | FLNB | filamin B, beta |
|  | JUND | jun D proto-oncogene |
|  | JUND | jun D proto-oncogene |
|  | CRKL | v-crk sarcoma virus CT10 oncogene homolog (avian)-like |
|  | RAP1A | RAP1A, member of RAS oncogene family |
|  | IKBKB | inhibitor of kappa light polypeptide gene enhancer in B-cells, kinase beta |
|  | IKBKB | inhibitor of kappa light polypeptide gene enhancer in B-cells, kinase beta |
|  | CDC25B | cell division cycle 25 homolog B (S. pombe) |
|  | PRKACB | protein kinase, cAMP-dependent, catalytic, beta |
|  | RASGRP1 | RAS guanyl releasing protein 1 (calcium and DAG-regulated) |
|  | PDGFRB | platelet-derived growth factor receptor, beta polypeptide |
|  | NRAS | neuroblastoma RAS viral (v-ras) oncogene homolog |
|  | ACVR1B | activin A receptor, type IB |
|  | MAP4K4 | mitogen-activated protein kinase kinase kinase kinase 4 |
|  | MAP4K4 | mitogen-activated protein kinase kinase kinase kinase 4 |
|  | MAP4K4 | mitogen-activated protein kinase kinase kinase kinase 4 |
|  | HSPA8 | heat shock 70kDa protein 8 |
|  | HSPA8 | heat shock 70kDa protein 8 |
|  | HSPA8 | heat shock 70kDa protein 8 |
|  | NTF4 | neurotrophin 4 |
|  | MAPKAPK5 | mitogen-activated protein kinase-activated protein kinase 5 |
|  | CD14 | CD14 molecule |
|  | FLNA | filamin A, alpha |
|  | FLNA | filamin A, alpha |
|  | DUSP4 | dual specificity phosphatase 4 |
|  | DUSP4 | dual specificity phosphatase 4 |
|  | FGFR1 | fibroblast growth factor receptor 1 |
|  | HSPA6 | heat shock 70kDa protein 6 (HSP70B') |
|  | MKNK1 | MAP kinase interacting serine/threonine kinase 1 |
|  | MAP3K7IP2 | mitogen-activated protein kinase kinase kinase 7 interacting protein 2 |
|  | MAP3K7IP2 | mitogen-activated protein kinase kinase kinase 7 interacting protein 2 |
|  | DUSP16 | dual specificity phosphatase 16 |
|  | RASGRF2 | Ras protein-specific guanine nucleotide-releasing factor 2 |
|  | GADD45B | growth arrest and DNA-damage-inducible, beta |
|  | GADD45B | growth arrest and DNA-damage-inducible, beta |
|  | CACNB3 | calcium channel, voltage-dependent, beta 3 subunit |
|  | CACNB3 | calcium channel, voltage-dependent, beta 3 subunit |
|  | MAPK10 | mitogen-activated protein kinase 10 |
|  | MAPK1 | mitogen-activated protein kinase 1 |
|  | MAPK1 | mitogen-activated protein kinase 1 |
|  | GNG12 | guanine nucleotide binding protein (G protein), gamma 12 |
|  | GNG12 | guanine nucleotide binding protein (G protein), gamma 12 |
|  | GNG12 | guanine nucleotide binding protein (G protein), gamma 12 |
|  | BRAF | v-raf murine sarcoma viral oncogene homolog B1 |
|  | JMJD7-PLA2G4B | JMJD7-PLA2G4B readthrough |
|  | MAPK8IP3 | mitogen-activated protein kinase 8 interacting protein 3 |
|  | MAPK8IP3 | mitogen-activated protein kinase 8 interacting protein 3 |
|  | TGFBR1 | transforming growth factor, beta receptor 1 |
|  | TGFBR1 | transforming growth factor, beta receptor 1 |
|  | PLA2G12A | phospholipase A2, group XIIA |
|  | PPP3CB | protein phosphatase 3 (formerly 2B), catalytic subunit, beta isoform |
|  | RPS6KA5 | ribosomal protein S6 kinase, 90kDa, polypeptide 5 |
|  | RPS6KA5 | ribosomal protein S6 kinase, 90kDa, polypeptide 5 |
|  | DUSP5 | dual specificity phosphatase 5 |
|  | JUN | jun oncogene |
|  | JUN | jun oncogene |
|  | JUN | jun oncogene |
|  | RAC2 | ras-related C3 botulinum toxin substrate 2 (rho family, small GTP binding protein Rac2) |
|  | FGFR3 | fibroblast growth factor receptor 3 |
|  | FGFR3 | fibroblast growth factor receptor 3 |
|  | MYC | v-myc myelocytomatosis viral oncogene homolog (avian) |
|  | CACNB2 | calcium channel, voltage-dependent, beta 2 subunit |
|  | MAPK9 | mitogen-activated protein kinase 9 |
|  | PAK2 | p21 protein (Cdc42/Rac)-activated kinase 2 |
|  | PAK2 | p21 protein (Cdc42/Rac)-activated kinase 2 |
|  | PAK2 | p21 protein (Cdc42/Rac)-activated kinase 2 |
|  | IL1R2 | interleukin 1 receptor, type II |
|  | MKNK2 | MAP kinase interacting serine/threonine kinase 2 |
|  | MAP3K7 | mitogen-activated protein kinase kinase kinase 7 |
|  | MAP3K7 | mitogen-activated protein kinase kinase kinase 7 |
|  | RRAS2 | related RAS viral (r-ras) oncogene homolog 2 |
|  | PLA2G6 | phospholipase A2, group VI (cytosolic, calcium-independent) |
|  | DUSP1 | dual specificity phosphatase 1 |
|  | TGFB1 | transforming growth factor, beta 1 |
|  | PAK1 | p21 protein (Cdc42/Rac)-activated kinase 1 |
|  | PAK1 | p21 protein (Cdc42/Rac)-activated kinase 1 |
|  | PPP3CA | protein phosphatase 3 (formerly 2B), catalytic subunit, alpha isoform |
|  | PPP3CA | protein phosphatase 3 (formerly 2B), catalytic subunit, alpha isoform |
|  | RRAS | related RAS viral (r-ras) oncogene homolog |
|  | SOS1 | son of sevenless homolog 1 (Drosophila) |
|  | NR4A1 | nuclear receptor subfamily 4, group A, member 1 |
|  | RAPGEF2 | Rap guanine nucleotide exchange factor (GEF) 2 |
|  | RAPGEF2 | Rap guanine nucleotide exchange factor (GEF) 2 |
|  | MAP4K3 | mitogen-activated protein kinase kinase kinase kinase 3 |
|  | FOS | FBJ murine osteosarcoma viral oncogene homolog |
|  | MAP2K2 | mitogen-activated protein kinase kinase 2 |
|  | EGFR | epidermal growth factor receptor (erythroblastic leukemia viral (v-erb-b) oncogene homolog, avian) |
|  | EGFR | epidermal growth factor receptor (erythroblastic leukemia viral (v-erb-b) oncogene homolog, avian) |
|  | EGFR | epidermal growth factor receptor (erythroblastic leukemia viral (v-erb-b) oncogene homolog, avian) |
|  | RASA1 | RAS p21 protein activator (GTPase activating protein) 1 |
|  | HSPA1B | heat shock 70kDa protein 1B |
|  | PLA2G2A | phospholipase A2, group IIA (platelets, synovial fluid) |
|  | FAS | Fas (TNF receptor superfamily, member 6) |
|  | FAS | Fas (TNF receptor superfamily, member 6) |
|  | FAS | Fas (TNF receptor superfamily, member 6) |
|  | FAS | Fas (TNF receptor superfamily, member 6) |
|  | GRB2 | growth factor receptor-bound protein 2 |
|  | IL1R1 | interleukin 1 receptor, type I |
|  | MAPKAPK2 | mitogen-activated protein kinase-activated protein kinase 2 |
|  | GNA12 | guanine nucleotide binding protein (G protein) alpha 12 |
|  | GADD45A | growth arrest and DNA-damage-inducible, alpha |
|  | FGFR2 | fibroblast growth factor receptor 2 |
|  | DUSP3 | dual specificity phosphatase 3 |
|  | PLA2G1B | phospholipase A2, group IB (pancreas) |
|  | AKT3 | v-akt murine thymoma viral oncogene homolog 3 (protein kinase B, gamma) |
|  | AKT3 | v-akt murine thymoma viral oncogene homolog 3 (protein kinase B, gamma) |
|  | AKT3 | v-akt murine thymoma viral oncogene homolog 3 (protein kinase B, gamma) |
|  | AKT3 | v-akt murine thymoma viral oncogene homolog 3 (protein kinase B, gamma) |
|  | PPP3R1 | protein phosphatase 3 (formerly 2B), regulatory subunit B, alpha isoform |
|  | MAP3K5 | mitogen-activated protein kinase kinase kinase 5 |
|  | MAP3K5 | mitogen-activated protein kinase kinase kinase 5 |
|  | ZAK | sterile alpha motif and leucine zipper containing kinase AZK |
|  | ZAK | sterile alpha motif and leucine zipper containing kinase AZK |
|  | ZAK | sterile alpha motif and leucine zipper containing kinase AZK |
|  | MAP2K4 | mitogen-activated protein kinase kinase 4 |
|  | RPS6KA3 | ribosomal protein S6 kinase, 90kDa, polypeptide 3 |
|  | RPS6KA3 | ribosomal protein S6 kinase, 90kDa, polypeptide 3 |
|  | STK4 | serine/threonine kinase 4 |
| **KEGG pathway----Regulation of actin cytoskeleton----04810** | **Gene Symbol** | **Gene Name** |
|  | MYL9 | myosin, light chain 9, regulatory |
|  | WASF1 | WAS protein family, member 1 |
|  | DOCK1 | dedicator of cytokinesis 1 |
|  | DOCK1 | dedicator of cytokinesis 1 |
|  | MAP2K1 | mitogen-activated protein kinase kinase 1 |
|  | MYH9 | myosin, heavy chain 9, non-muscle |
|  | ENAH | enabled homolog (Drosophila) |
|  | ENAH | enabled homolog (Drosophila) |
|  | ENAH | enabled homolog (Drosophila) |
|  | APC | adenomatous polyposis coli |
|  | DIAPH3 | diaphanous homolog 3 (Drosophila) |
|  | PDGFRA | platelet-derived growth factor receptor, alpha polypeptide |
|  | MYH10 | myosin, heavy chain 10, non-muscle |
|  | CRKL | v-crk sarcoma virus CT10 oncogene homolog (avian)-like |
|  | C3orf10 | chromosome 3 open reading frame 10 |
|  | PIK3CA | phosphoinositide-3-kinase, catalytic, alpha polypeptide |
|  | MYH14 | myosin, heavy chain 14 |
|  | MYH14 | myosin, heavy chain 14 |
|  | F2R | coagulation factor II (thrombin) receptor |
|  | TIAM1 | T-cell lymphoma invasion and metastasis 1 |
|  | ITGB6 | integrin, beta 6 |
|  | PDGFRB | platelet-derived growth factor receptor, beta polypeptide |
|  | CFL1 | cofilin 1 (non-muscle) |
|  | SSH3 | slingshot homolog 3 (Drosophila) |
|  | SSH3 | slingshot homolog 3 (Drosophila) |
|  | NRAS | neuroblastoma RAS viral (v-ras) oncogene homolog |
|  | EZR | ezrin |
|  | ACTN1 | actinin, alpha 1 |
|  | ACTN1 | actinin, alpha 1 |
|  | ACTN1 | actinin, alpha 1 |
|  | CD14 | CD14 molecule |
|  | FGFR1 | fibroblast growth factor receptor 1 |
|  | INS | insulin |
|  | ITGB2 | integrin, beta 2 (complement component 3 receptor 3 and 4 subunit) |
|  | GSN | gelsolin (amyloidosis, Finnish type) |
|  | GSN | gelsolin (amyloidosis, Finnish type) |
|  | ITGAE | integrin, alpha E (antigen CD103, human mucosal lymphocyte antigen 1; alpha polypeptide) |
|  | ARHGEF6 | Rac/Cdc42 guanine nucleotide exchange factor (GEF) 6 |
|  | ITGA2 | integrin, alpha 2 (CD49B, alpha 2 subunit of VLA-2 receptor) |
|  | MYLK | myosin light chain kinase |
|  | ITGB1 | integrin, beta 1 (fibronectin receptor, beta polypeptide, antigen CD29 includes MDF2, MSK12) |
|  | ITGB1 | integrin, beta 1 (fibronectin receptor, beta polypeptide, antigen CD29 includes MDF2, MSK12) |
|  | PPP1CB | protein phosphatase 1, catalytic subunit, beta isoform |
|  | PPP1CB | protein phosphatase 1, catalytic subunit, beta isoform |
|  | ROCK1 | Rho-associated, coiled-coil containing protein kinase 1 |
|  | ROCK1 | Rho-associated, coiled-coil containing protein kinase 1 |
|  | GNA13 | guanine nucleotide binding protein (G protein), alpha 13 |
|  | MAPK1 | mitogen-activated protein kinase 1 |
|  | MAPK1 | mitogen-activated protein kinase 1 |
|  | GNG12 | guanine nucleotide binding protein (G protein), gamma 12 |
|  | GNG12 | guanine nucleotide binding protein (G protein), gamma 12 |
|  | GNG12 | guanine nucleotide binding protein (G protein), gamma 12 |
|  | BRAF | v-raf murine sarcoma viral oncogene homolog B1 |
|  | FN1 | fibronectin 1 |
|  | FN1 | fibronectin 1 |
|  | FN1 | fibronectin 1 |
|  | FN1 | fibronectin 1 |
|  | PPP1R12A | protein phosphatase 1, regulatory (inhibitor) subunit 12A |
|  | ARPC4 | actin related protein 2/3 complex, subunit 4, 20kDa |
|  | PFN2 | profilin 2 |
|  | ITGB5 | integrin, beta 5 |
|  | ITGB5 | integrin, beta 5 |
|  | PPP1CC | protein phosphatase 1, catalytic subunit, gamma isoform |
|  | PIP4K2A | phosphatidylinositol-5-phosphate 4-kinase, type II, alpha |
|  | RAC2 | ras-related C3 botulinum toxin substrate 2 (rho family, small GTP binding protein Rac2) |
|  | FGFR3 | fibroblast growth factor receptor 3 |
|  | FGFR3 | fibroblast growth factor receptor 3 |
|  | ITGB8 | integrin, beta 8 |
|  | ITGA3 | integrin, alpha 3 (antigen CD49C, alpha 3 subunit of VLA-3 receptor) |
|  | ARPC5 | actin related protein 2/3 complex, subunit 5, 16kDa |
|  | ARPC5 | actin related protein 2/3 complex, subunit 5, 16kDa |
|  | PAK2 | p21 protein (Cdc42/Rac)-activated kinase 2 |
|  | PAK2 | p21 protein (Cdc42/Rac)-activated kinase 2 |
|  | PAK2 | p21 protein (Cdc42/Rac)-activated kinase 2 |
|  | SLC9A1 | solute carrier family 9 (sodium/hydrogen exchanger), member 1 |
|  | RDX | radixin |
|  | RDX | radixin |
|  | RRAS2 | related RAS viral (r-ras) oncogene homolog 2 |
|  | ROCK2 | Rho-associated, coiled-coil containing protein kinase 2 |
|  | LIMK2 | LIM domain kinase 2 |
|  | LIMK2 | LIM domain kinase 2 |
|  | PAK1 | p21 protein (Cdc42/Rac)-activated kinase 1 |
|  | PAK1 | p21 protein (Cdc42/Rac)-activated kinase 1 |
|  | VAV3 | vav 3 guanine nucleotide exchange factor |
|  | PDGFC | platelet derived growth factor C |
|  | PXN | paxillin |
|  | RHOA | ras homolog gene family, member A |
|  | RHOA | ras homolog gene family, member A |
|  | RRAS | related RAS viral (r-ras) oncogene homolog |
|  | SOS1 | son of sevenless homolog 1 (Drosophila) |
|  | ITGA5 | integrin, alpha 5 (fibronectin receptor, alpha polypeptide) |
|  | PTK2 | PTK2 protein tyrosine kinase 2 |
|  | PTK2 | PTK2 protein tyrosine kinase 2 |
|  | CYFIP2 | cytoplasmic FMR1 interacting protein 2 |
|  | ARPC1A | actin related protein 2/3 complex, subunit 1A, 41kDa |
|  | MAP2K2 | mitogen-activated protein kinase kinase 2 |
|  | PIK3CB | phosphoinositide-3-kinase, catalytic, beta polypeptide |
|  | EGFR | epidermal growth factor receptor (erythroblastic leukemia viral (v-erb-b) oncogene homolog, avian) |
|  | EGFR | epidermal growth factor receptor (erythroblastic leukemia viral (v-erb-b) oncogene homolog, avian) |
|  | EGFR | epidermal growth factor receptor (erythroblastic leukemia viral (v-erb-b) oncogene homolog, avian) |
|  | BAIAP2 | BAI1-associated protein 2 |
|  | CFL2 | cofilin 2 (muscle) |
|  | CFL2 | cofilin 2 (muscle) |
|  | GNA12 | guanine nucleotide binding protein (G protein) alpha 12 |
|  | FGFR2 | fibroblast growth factor receptor 2 |
|  | ITGAV | integrin, alpha V (vitronectin receptor, alpha polypeptide, antigen CD51) |
|  | ITGB4 | integrin, beta 4 |
|  | MSN | moesin |
| **KEGG pathway----Insulin signaling pathway----04910** | **Gene Symbol** | **Gene Name** |
|  | MAPK8 | mitogen-activated protein kinase 8 |
|  | ACACB | acetyl-Coenzyme A carboxylase beta |
|  | PRKAB1 | protein kinase, AMP-activated, beta 1 non-catalytic subunit |
|  | SORBS1 | sorbin and SH3 domain containing 1 |
|  | SORBS1 | sorbin and SH3 domain containing 1 |
|  | BRAF | v-raf murine sarcoma viral oncogene homolog B1 |
|  | FOXO1 | forkhead box O1 |
|  | TRIP10 | thyroid hormone receptor interactor 10 |
|  | PRKAB2 | protein kinase, AMP-activated, beta 2 non-catalytic subunit |
|  | PRKAB2 | protein kinase, AMP-activated, beta 2 non-catalytic subunit |
|  | INPP5D | inositol polyphosphate-5-phosphatase, 145kDa |
|  | SOCS2 | suppressor of cytokine signaling 2 |
|  | PRKAA1 | protein kinase, AMP-activated, alpha 1 catalytic subunit |
|  | MAP2K1 | mitogen-activated protein kinase kinase 1 |
|  | PPP1R3C | protein phosphatase 1, regulatory (inhibitor) subunit 3C |
|  | PPP1CC | protein phosphatase 1, catalytic subunit, gamma isoform |
|  | SHC2 | SHC (Src homology 2 domain containing) transforming protein 2 |
|  | CRKL | v-crk sarcoma virus CT10 oncogene homolog (avian)-like |
|  | CALM2 | calmodulin 2 (phosphorylase kinase, delta) |
|  | SHC1 | SHC (Src homology 2 domain containing) transforming protein 1 |
|  | IKBKB | inhibitor of kappa light polypeptide gene enhancer in B-cells, kinase beta |
|  | IKBKB | inhibitor of kappa light polypeptide gene enhancer in B-cells, kinase beta |
|  | MAPK9 | mitogen-activated protein kinase 9 |
|  | PIK3CA | phosphoinositide-3-kinase, catalytic, alpha polypeptide |
|  | SREBF1 | sterol regulatory element binding transcription factor 1 |
|  | MKNK2 | MAP kinase interacting serine/threonine kinase 2 |
|  | EIF4E | eukaryotic translation initiation factor 4E |
|  | EIF4E | eukaryotic translation initiation factor 4E |
|  | PRKACB | protein kinase, cAMP-dependent, catalytic, beta |
|  | ACACA | acetyl-Coenzyme A carboxylase alpha |
|  | PDPK1 | 3-phosphoinositide dependent protein kinase-1 |
|  | NRAS | neuroblastoma RAS viral (v-ras) oncogene homolog |
|  | EIF4E2 | eukaryotic translation initiation factor 4E family member 2 |
|  | RHOQ | ras homolog gene family, member Q |
|  | RHOQ | ras homolog gene family, member Q |
|  | RHOQ | ras homolog gene family, member Q |
|  | SOS1 | son of sevenless homolog 1 (Drosophila) |
|  | CALM1 | calmodulin 1 (phosphorylase kinase, delta) |
|  | CBLB | Cas-Br-M (murine) ecotropic retroviral transforming sequence b |
|  | FASN | fatty acid synthase |
|  | PYGL | phosphorylase, glycogen, liver |
|  | PIK3CB | phosphoinositide-3-kinase, catalytic, beta polypeptide |
|  | MAP2K2 | mitogen-activated protein kinase kinase 2 |
|  | RHEB | Ras homolog enriched in brain |
|  | INS | insulin |
|  | PYGB | phosphorylase, glycogen; brain |
|  | SOCS3 | suppressor of cytokine signaling 3 |
|  | GRB2 | growth factor receptor-bound protein 2 |
|  | IRS2 | insulin receptor substrate 2 |
|  | IRS2 | insulin receptor substrate 2 |
|  | PTPRF | protein tyrosine phosphatase, receptor type, F |
|  | PTPRF | protein tyrosine phosphatase, receptor type, F |
|  | IRS1 | insulin receptor substrate 1 |
|  | TSC1 | tuberous sclerosis 1 |
|  | MKNK1 | MAP kinase interacting serine/threonine kinase 1 |
|  | INSR | insulin receptor |
|  | INSR | insulin receptor |
|  | AKT3 | v-akt murine thymoma viral oncogene homolog 3 (protein kinase B, gamma) |
|  | AKT3 | v-akt murine thymoma viral oncogene homolog 3 (protein kinase B, gamma) |
|  | AKT3 | v-akt murine thymoma viral oncogene homolog 3 (protein kinase B, gamma) |
|  | AKT3 | v-akt murine thymoma viral oncogene homolog 3 (protein kinase B, gamma) |
|  | FBP1 | fructose-1,6-bisphosphatase 1 |
|  | EIF4EBP1 | eukaryotic translation initiation factor 4E binding protein 1 |
|  | PRKCZ | protein kinase C, zeta |
|  | PPP1CB | protein phosphatase 1, catalytic subunit, beta isoform |
|  | PPP1CB | protein phosphatase 1, catalytic subunit, beta isoform |
|  | RPS6KB1 | ribosomal protein S6 kinase, 70kDa, polypeptide 1 |
|  | MAPK10 | mitogen-activated protein kinase 10 |
|  | MAPK1 | mitogen-activated protein kinase 1 |
|  | MAPK1 | mitogen-activated protein kinase 1 |
|  | PRKAR2A | protein kinase, cAMP-dependent, regulatory, type II, alpha |
|  | PRKAR1A | protein kinase, cAMP-dependent, regulatory, type I, alpha (tissue specific extinguisher 1) |
|  | PRKAR1A | protein kinase, cAMP-dependent, regulatory, type I, alpha (tissue specific extinguisher 1) |
|  | PRKAR1A | protein kinase, cAMP-dependent, regulatory, type I, alpha (tissue specific extinguisher 1) |
| **KEGG pathway----Oocyte meiosis----04114** | **Gene Symbol** | **Gene Name** |
|  | PTTG1 | pituitary tumor-transforming 1 |
|  | MAP2K1 | mitogen-activated protein kinase kinase 1 |
|  | PPP2CB | protein phosphatase 2 (formerly 2A), catalytic subunit, beta isoform |
|  | CUL1 | cullin 1 |
|  | PPP3CB | protein phosphatase 3 (formerly 2B), catalytic subunit, beta isoform |
|  | YWHAE | tyrosine 3-monooxygenase/tryptophan 5-monooxygenase activation protein, epsilon polypeptide |
|  | CCNB1 | cyclin B1 |
|  | CCNB1 | cyclin B1 |
|  | CCNE1 | cyclin E1 |
|  | PPP1CC | protein phosphatase 1, catalytic subunit, gamma isoform |
|  | MAD2L2 | MAD2 mitotic arrest deficient-like 2 (yeast) |
|  | PPP2R1B | protein phosphatase 2 (formerly 2A), regulatory subunit A, beta isoform |
|  | PPP2R1B | protein phosphatase 2 (formerly 2A), regulatory subunit A, beta isoform |
|  | PPP2R1B | protein phosphatase 2 (formerly 2A), regulatory subunit A, beta isoform |
|  | FBXO5 | F-box protein 5 |
|  | CALM2 | calmodulin 2 (phosphorylase kinase, delta) |
|  | SMC3 | structural maintenance of chromosomes 3 |
|  | SMC3 | structural maintenance of chromosomes 3 |
|  | SMC3 | structural maintenance of chromosomes 3 |
|  | ANAPC11 | anaphase promoting complex subunit 11 |
|  | ANAPC10 | anaphase promoting complex subunit 10 |
|  | ADCY3 | adenylate cyclase 3 |
|  | BUB1 | budding uninhibited by benzimidazoles 1 homolog (yeast) |
|  | PRKACB | protein kinase, cAMP-dependent, catalytic, beta |
|  | CAMK2G | calcium/calmodulin-dependent protein kinase II gamma |
|  | ADCY7 | adenylate cyclase 7 |
|  | PPP3CA | protein phosphatase 3 (formerly 2B), catalytic subunit, alpha isoform |
|  | PPP3CA | protein phosphatase 3 (formerly 2B), catalytic subunit, alpha isoform |
|  | RBX1 | ring-box 1 |
|  | CDC20 | cell division cycle 20 homolog (S. cerevisiae) |
|  | CALM1 | calmodulin 1 (phosphorylase kinase, delta) |
|  | YWHAQ | tyrosine 3-monooxygenase/tryptophan 5-monooxygenase activation protein, theta polypeptide |
|  | YWHAQ | tyrosine 3-monooxygenase/tryptophan 5-monooxygenase activation protein, theta polypeptide |
|  | CDC23 | cell division cycle 23 homolog (S. cerevisiae) |
|  | ITPR3 | inositol 1,4,5-triphosphate receptor, type 3 |
|  | CDC26 | cell division cycle 26 homolog (S. cerevisiae) |
|  | CDC25C | cell division cycle 25 homolog C (S. pombe) |
|  | CDC2 | cell division cycle 2, G1 to S and G2 to M |
|  | CDC2 | cell division cycle 2, G1 to S and G2 to M |
|  | CDC2 | cell division cycle 2, G1 to S and G2 to M |
|  | INS | insulin |
|  | CAMK2D | calcium/calmodulin-dependent protein kinase II delta |
|  | CDC27 | cell division cycle 27 homolog (S. cerevisiae) |
|  | CDC27 | cell division cycle 27 homolog (S. cerevisiae) |
|  | ITPR1 | inositol 1,4,5-triphosphate receptor, type 1 |
|  | MAD2L1 | MAD2 mitotic arrest deficient-like 1 (yeast) |
|  | MAD2L1 | MAD2 mitotic arrest deficient-like 1 (yeast) |
|  | PPP3R1 | protein phosphatase 3 (formerly 2B), regulatory subunit B, alpha isoform |
|  | SMC1A | structural maintenance of chromosomes 1A |
|  | PPP1CB | protein phosphatase 1, catalytic subunit, beta isoform |
|  | PPP1CB | protein phosphatase 1, catalytic subunit, beta isoform |
|  | PPP2R5C | protein phosphatase 2, regulatory subunit B', gamma isoform |
|  | PPP2R5C | protein phosphatase 2, regulatory subunit B', gamma isoform |
|  | PPP2R5C | protein phosphatase 2, regulatory subunit B', gamma isoform |
|  | SKP1 | S-phase kinase-associated protein 1 |
|  | SKP1 | S-phase kinase-associated protein 1 |
|  | AURKA | aurora kinase A |
|  | AURKA | aurora kinase A |
|  | YWHAZ | tyrosine 3-monooxygenase/tryptophan 5-monooxygenase activation protein, zeta polypeptide |
|  | YWHAZ | tyrosine 3-monooxygenase/tryptophan 5-monooxygenase activation protein, zeta polypeptide |
|  | CDK2 | cyclin-dependent kinase 2 |
|  | YWHAH | tyrosine 3-monooxygenase/tryptophan 5-monooxygenase activation protein, eta polypeptide |
|  | CCNB2 | cyclin B2 |
|  | MAPK1 | mitogen-activated protein kinase 1 |
|  | MAPK1 | mitogen-activated protein kinase 1 |
|  | ESPL1 | extra spindle pole bodies homolog 1 (S. cerevisiae) |
|  | YWHAB | tyrosine 3-monooxygenase/tryptophan 5-monooxygenase activation protein, beta polypeptide |
|  | RPS6KA3 | ribosomal protein S6 kinase, 90kDa, polypeptide 3 |
|  | RPS6KA3 | ribosomal protein S6 kinase, 90kDa, polypeptide 3 |
|  | BTRC | beta-transducin repeat containing |
| **KEGG pathway----Huntington's disease----05016** | **Gene Symbol** | **Gene Name** |
|  | NDUFC2 | NADH dehydrogenase (ubiquinone) 1, subcomplex unknown, 2, 14.5kDa |
|  | NDUFB6 | NADH dehydrogenase (ubiquinone) 1 beta subcomplex, 6, 17kDa |
|  | UQCRB | ubiquinol-cytochrome c reductase binding protein |
|  | POLR2F | polymerase (RNA) II (DNA directed) polypeptide F |
|  | UCRC | ubiquinol-cytochrome c reductase complex (7.2 kD) |
|  | NDUFB5 | NADH dehydrogenase (ubiquinone) 1 beta subcomplex, 5, 16kDa |
|  | SOD2 | superoxide dismutase 2, mitochondrial |
|  | SOD2 | superoxide dismutase 2, mitochondrial |
|  | NDUFAB1 | NADH dehydrogenase (ubiquinone) 1, alpha/beta subcomplex, 1, 8kDa |
|  | hCG_1776980 | hCG1776980 |
|  | NDUFB3 | NADH dehydrogenase (ubiquinone) 1 beta subcomplex, 3, 12kDa |
|  | UQCRFS1 | ubiquinol-cytochrome c reductase, Rieske iron-sulfur polypeptide 1 |
|  | PPID | peptidylprolyl isomerase D |
|  | PPID | peptidylprolyl isomerase D |
|  | PPID | peptidylprolyl isomerase D |
|  | UQCRC2 | ubiquinol-cytochrome c reductase core protein II |
|  | NDUFB8 | NADH dehydrogenase (ubiquinone) 1 beta subcomplex, 8, 19kDa |
|  | AP2B1 | adaptor-related protein complex 2, beta 1 subunit |
|  | VDAC3 | voltage-dependent anion channel 3 |
|  | NDUFA6 | NADH dehydrogenase (ubiquinone) 1 alpha subcomplex, 6, 14kDa |
|  | CLTB | clathrin, light chain (Lcb) |
|  | CLTB | clathrin, light chain (Lcb) |
|  | CLTB | clathrin, light chain (Lcb) |
|  | NDUFS2 | NADH dehydrogenase (ubiquinone) Fe-S protein 2, 49kDa (NADH-coenzyme Q reductase) |
|  | COX7C | cytochrome c oxidase subunit VIIc |
|  | POLR2G | polymerase (RNA) II (DNA directed) polypeptide G |
|  | PPARG | peroxisome proliferator-activated receptor gamma |
|  | POLR2D | polymerase (RNA) II (DNA directed) polypeptide D |
|  | VDAC1 | voltage-dependent anion channel 1 |
|  | SLC25A5 | solute carrier family 25 (mitochondrial carrier; adenine nucleotide translocator), member 5 |
|  | SDHD | succinate dehydrogenase complex, subunit D, integral membrane protein |
|  | ATP5F1 | ATP synthase, H+ transporting, mitochondrial F0 complex, subunit B1 |
|  | POLR2C | polymerase (RNA) II (DNA directed) polypeptide C, 33kDa |
|  | CLTC | clathrin, heavy chain (Hc) |
|  | UQCRH | ubiquinol-cytochrome c reductase hinge protein |
|  | NDUFB7 | NADH dehydrogenase (ubiquinone) 1 beta subcomplex, 7, 18kDa |
|  | NDUFA8 | NADH dehydrogenase (ubiquinone) 1 alpha subcomplex, 8, 19kDa |
|  | AP2S1 | adaptor-related protein complex 2, sigma 1 subunit |
|  | AP2S1 | adaptor-related protein complex 2, sigma 1 subunit |
|  | TFAM | transcription factor A, mitochondrial |
|  | TFAM | transcription factor A, mitochondrial |
|  | AP2M1 | adaptor-related protein complex 2, mu 1 subunit |
|  | POLR2K | polymerase (RNA) II (DNA directed) polypeptide K, 7.0kDa |
|  | POLR2K | polymerase (RNA) II (DNA directed) polypeptide K, 7.0kDa |
|  | SDHC | succinate dehydrogenase complex, subunit C, integral membrane protein, 15kDa |
|  | SDHC | succinate dehydrogenase complex, subunit C, integral membrane protein, 15kDa |
|  | NDUFS6 | NADH dehydrogenase (ubiquinone) Fe-S protein 6, 13kDa (NADH-coenzyme Q reductase) |
|  | COX4I1 | cytochrome c oxidase subunit IV isoform 1 |
|  | NDUFA4 | NADH dehydrogenase (ubiquinone) 1 alpha subcomplex, 4, 9kDa |
|  | NDUFS7 | NADH dehydrogenase (ubiquinone) Fe-S protein 7, 20kDa (NADH-coenzyme Q reductase) |
|  | NDUFS4 | NADH dehydrogenase (ubiquinone) Fe-S protein 4, 18kDa (NADH-coenzyme Q reductase) |
|  | NDUFB4 | NADH dehydrogenase (ubiquinone) 1 beta subcomplex, 4, 15kDa |
|  | ATP5H | ATP synthase, H+ transporting, mitochondrial F0 complex, subunit d |
|  | NDUFB2 | NADH dehydrogenase (ubiquinone) 1 beta subcomplex, 2, 8kDa |
|  | NDUFB2 | NADH dehydrogenase (ubiquinone) 1 beta subcomplex, 2, 8kDa |
|  | DCTN4 | dynactin 4 (p62) |
|  | BAX | BCL2-associated X protein |
|  | BAX | BCL2-associated X protein |
|  | NDUFS8 | NADH dehydrogenase (ubiquinone) Fe-S protein 8, 23kDa (NADH-coenzyme Q reductase) |
|  | COX7B | cytochrome c oxidase subunit VIIb |
|  | CYC1 | cytochrome c-1 |
|  | CREB1 | cAMP responsive element binding protein 1 |
|  | SP1 | Sp1 transcription factor |
|  | ATP5G1 | ATP synthase, H+ transporting, mitochondrial F0 complex, subunit C1 (subunit 9) |
|  | POLR2I | polymerase (RNA) II (DNA directed) polypeptide I, 14.5kDa |
|  | CREB3L4 | cAMP responsive element binding protein 3-like 4 |
|  | HIP1 | huntingtin interacting protein 1 |
|  | HIP1 | huntingtin interacting protein 1 |
|  | RCOR1 | REST corepressor 1 |
|  | SDHB | succinate dehydrogenase complex, subunit B, iron sulfur (Ip) |
|  | DNALI1 | dynein, axonemal, light intermediate chain 1 |
|  | ATP5G3 | ATP synthase, H+ transporting, mitochondrial F0 complex, subunit C3 (subunit 9) |
|  | ATP5C1 | ATP synthase, H+ transporting, mitochondrial F1 complex, gamma polypeptide 1 |
|  | COX7A2L | cytochrome c oxidase subunit VIIa polypeptide 2 like |
|  | TAF4 | TAF4 RNA polymerase II, TATA box binding protein (TBP)-associated factor, 135kDa |
|  | ITPR1 | inositol 1,4,5-triphosphate receptor, type 1 |
|  | POLR2E | polymerase (RNA) II (DNA directed) polypeptide E, 25kDa |
|  | TBPL1 | TBP-like 1 |
|  | GNAQ | guanine nucleotide binding protein (G protein), q polypeptide |
|  | ATP5O | ATP synthase, H+ transporting, mitochondrial F1 complex, O subunit |
| **KEGG pathway----Purine metabolism----00230** | **Gene Symbol** | **Gene Name** |
|  | AK2 | adenylate kinase 2 |
|  | AK2 | adenylate kinase 2 |
|  | AK2 | adenylate kinase 2 |
|  | AK2 | adenylate kinase 2 |
|  | PAPSS2 | 3'-phosphoadenosine 5'-phosphosulfate synthase 2 |
|  | PAPSS2 | 3'-phosphoadenosine 5'-phosphosulfate synthase 2 |
|  | POLR2F | polymerase (RNA) II (DNA directed) polypeptide F |
|  | POLR2K | polymerase (RNA) II (DNA directed) polypeptide K, 7.0kDa |
|  | POLR2K | polymerase (RNA) II (DNA directed) polypeptide K, 7.0kDa |
|  | NPR2 | natriuretic peptide receptor B/guanylate cyclase B (atrionatriuretic peptide receptor B) |
|  | RRM1 | ribonucleotide reductase M1 |
|  | RRM1 | ribonucleotide reductase M1 |
|  | APRT | adenine phosphoribosyltransferase |
|  | POLD1 | polymerase (DNA directed), delta 1, catalytic subunit 125kDa |
|  | POLR3K | polymerase (RNA) III (DNA directed) polypeptide K, 12.3 kDa |
|  | ADA | adenosine deaminase |
|  | GART | phosphoribosylglycinamide formyltransferase, phosphoribosylglycinamide synthetase, phosphoribosylaminoimidazole synthetase |
|  | GART | phosphoribosylglycinamide formyltransferase, phosphoribosylglycinamide synthetase, phosphoribosylaminoimidazole synthetase |
|  | GART | phosphoribosylglycinamide formyltransferase, phosphoribosylglycinamide synthetase, phosphoribosylaminoimidazole synthetase |
|  | ENTPD4 | ectonucleoside triphosphate diphosphohydrolase 4 |
|  | PAICS | phosphoribosylaminoimidazole carboxylase, phosphoribosylaminoimidazole succinocarboxamide synthetase |
|  | PAICS | phosphoribosylaminoimidazole carboxylase, phosphoribosylaminoimidazole succinocarboxamide synthetase |
|  | GMPS | guanine monphosphate synthetase |
|  | ADCY3 | adenylate cyclase 3 |
|  | NUDT5 | nudix (nucleoside diphosphate linked moiety X)-type motif 5 |
|  | POLE3 | polymerase (DNA directed), epsilon 3 (p17 subunit) |
|  | POLR1B | polymerase (RNA) I polypeptide B, 128kDa |
|  | POLR3C | polymerase (RNA) III (DNA directed) polypeptide C (62kD) |
|  | ZNRD1 | zinc ribbon domain containing 1 |
|  | ZNRD1 | zinc ribbon domain containing 1 |
|  | PRPS1 | phosphoribosyl pyrophosphate synthetase 1 |
|  | NT5E | 5'-nucleotidase, ecto (CD73) |
|  | ADCY7 | adenylate cyclase 7 |
|  | POLR3F | polymerase (RNA) III (DNA directed) polypeptide F, 39 kDa |
|  | POLR2I | polymerase (RNA) II (DNA directed) polypeptide I, 14.5kDa |
|  | POLR2G | polymerase (RNA) II (DNA directed) polypeptide G |
|  | PRIM2 | primase, DNA, polypeptide 2 (58kDa) |
|  | PRIM2 | primase, DNA, polypeptide 2 (58kDa) |
|  | PRIM2 | primase, DNA, polypeptide 2 (58kDa) |
|  | NME6 | non-metastatic cells 6, protein expressed in (nucleoside-diphosphate kinase) |
|  | NP | nucleoside phosphorylase |
|  | PPAT | phosphoribosyl pyrophosphate amidotransferase |
|  | PPAT | phosphoribosyl pyrophosphate amidotransferase |
|  | POLA1 | polymerase (DNA directed), alpha 1, catalytic subunit |
|  | POLR2D | polymerase (RNA) II (DNA directed) polypeptide D |
|  | RRM2 | ribonucleotide reductase M2 |
|  | AK3L1 | adenylate kinase 3-like 1 |
|  | AK3L1 | adenylate kinase 3-like 1 |
|  | ADSL | adenylosuccinate lyase |
|  | ADSL | adenylosuccinate lyase |
|  | NUDT2 | nudix (nucleoside diphosphate linked moiety X)-type motif 2 |
|  | POLD3 | polymerase (DNA-directed), delta 3, accessory subunit |
|  | POLE4 | polymerase (DNA-directed), epsilon 4 (p12 subunit) |
|  | POLE4 | polymerase (DNA-directed), epsilon 4 (p12 subunit) |
|  | PRIM1 | primase, DNA, polypeptide 1 (49kDa) |
|  | POLR2C | polymerase (RNA) II (DNA directed) polypeptide C, 33kDa |
|  | GMPR2 | guanosine monophosphate reductase 2 |
|  | HPRT1 | hypoxanthine phosphoribosyltransferase 1 |
|  | NT5C2 | 5'-nucleotidase, cytosolic II |
|  | POLE2 | polymerase (DNA directed), epsilon 2 (p59 subunit) |
|  | NME1 | non-metastatic cells 1, protein (NM23A) expressed in |
|  | NME7 | non-metastatic cells 7, protein expressed in (nucleoside-diphosphate kinase) |
|  | POLR1D | polymerase (RNA) I polypeptide D, 16kDa |
|  | GUCY1B3 | guanylate cyclase 1, soluble, beta 3 |
|  | DCK | deoxycytidine kinase |
|  | PRPS2 | phosphoribosyl pyrophosphate synthetase 2 |
|  | PRPS2 | phosphoribosyl pyrophosphate synthetase 2 |
|  | POLR2E | polymerase (RNA) II (DNA directed) polypeptide E, 25kDa |
|  | PDE7A | phosphodiesterase 7A |
|  | CANT1 | calcium activated nucleotidase 1 |
|  | CANT1 | calcium activated nucleotidase 1 |
|  | ENTPD3 | ectonucleoside triphosphate diphosphohydrolase 3 |
|  | POLR1C | polymerase (RNA) I polypeptide C, 30kDa |
|  | POLR1C | polymerase (RNA) I polypeptide C, 30kDa |
|  | ADK | adenosine kinase |
|  | ADK | adenosine kinase |
|  | GUCY1A3 | guanylate cyclase 1, soluble, alpha 3 |
|  | PNPT1 | polyribonucleotide nucleotidyltransferase 1 |
|  | PDE9A | phosphodiesterase 9A |
|  | POLD2 | polymerase (DNA directed), delta 2, regulatory subunit 50kDa |
| **KEGG pathway----Alzheimer's disease----05010** | **Gene Symbol** | **Gene Name** |
|  | NDUFC2 | NADH dehydrogenase (ubiquinone) 1, subcomplex unknown, 2, 14.5kDa |
|  | NDUFB6 | NADH dehydrogenase (ubiquinone) 1 beta subcomplex, 6, 17kDa |
|  | UQCRB | ubiquinol-cytochrome c reductase binding protein |
|  | ADAM10 | ADAM metallopeptidase domain 10 |
|  | ADAM10 | ADAM metallopeptidase domain 10 |
|  | UCRC | ubiquinol-cytochrome c reductase complex (7.2 kD) |
|  | NDUFB5 | NADH dehydrogenase (ubiquinone) 1 beta subcomplex, 5, 16kDa |
|  | EIF2AK3 | eukaryotic translation initiation factor 2-alpha kinase 3 |
|  | NDUFAB1 | NADH dehydrogenase (ubiquinone) 1, alpha/beta subcomplex, 1, 8kDa |
|  | hCG_1776980 | hCG1776980 |
|  | NDUFB3 | NADH dehydrogenase (ubiquinone) 1 beta subcomplex, 3, 12kDa |
|  | UQCRFS1 | ubiquinol-cytochrome c reductase, Rieske iron-sulfur polypeptide 1 |
|  | CDK5 | cyclin-dependent kinase 5 |
|  | CALM2 | calmodulin 2 (phosphorylase kinase, delta) |
|  | ATP2A2 | ATPase, Ca++ transporting, cardiac muscle, slow twitch 2 |
|  | UQCRC2 | ubiquinol-cytochrome c reductase core protein II |
|  | NDUFB8 | NADH dehydrogenase (ubiquinone) 1 beta subcomplex, 8, 19kDa |
|  | NDUFA6 | NADH dehydrogenase (ubiquinone) 1 alpha subcomplex, 6, 14kDa |
|  | NDUFS2 | NADH dehydrogenase (ubiquinone) Fe-S protein 2, 49kDa (NADH-coenzyme Q reductase) |
|  | NAE1 | NEDD8 activating enzyme E1 subunit 1 |
|  | COX7C | cytochrome c oxidase subunit VIIc |
|  | APH1A | anterior pharynx defective 1 homolog A (C. elegans) |
|  | LRP1 | low density lipoprotein-related protein 1 (alpha-2-macroglobulin receptor) |
|  | ITPR3 | inositol 1,4,5-triphosphate receptor, type 3 |
|  | ATP5F1 | ATP synthase, H+ transporting, mitochondrial F0 complex, subunit B1 |
|  | SDHD | succinate dehydrogenase complex, subunit D, integral membrane protein |
|  | IDE | insulin-degrading enzyme |
|  | UQCRH | ubiquinol-cytochrome c reductase hinge protein |
|  | NDUFB7 | NADH dehydrogenase (ubiquinone) 1 beta subcomplex, 7, 18kDa |
|  | NDUFA8 | NADH dehydrogenase (ubiquinone) 1 alpha subcomplex, 8, 19kDa |
|  | MAPK1 | mitogen-activated protein kinase 1 |
|  | MAPK1 | mitogen-activated protein kinase 1 |
|  | CAPN1 | calpain 1, (mu/I) large subunit |
|  | SDHC | succinate dehydrogenase complex, subunit C, integral membrane protein, 15kDa |
|  | SDHC | succinate dehydrogenase complex, subunit C, integral membrane protein, 15kDa |
|  | ATP2A3 | ATPase, Ca++ transporting, ubiquitous |
|  | NDUFS6 | NADH dehydrogenase (ubiquinone) Fe-S protein 6, 13kDa (NADH-coenzyme Q reductase) |
|  | HSD17B10 | hydroxysteroid (17-beta) dehydrogenase 10 |
|  | COX4I1 | cytochrome c oxidase subunit IV isoform 1 |
|  | NDUFA4 | NADH dehydrogenase (ubiquinone) 1 alpha subcomplex, 4, 9kDa |
|  | PPP3CB | protein phosphatase 3 (formerly 2B), catalytic subunit, beta isoform |
|  | NDUFS7 | NADH dehydrogenase (ubiquinone) Fe-S protein 7, 20kDa (NADH-coenzyme Q reductase) |
|  | NDUFS4 | NADH dehydrogenase (ubiquinone) Fe-S protein 4, 18kDa (NADH-coenzyme Q reductase) |
|  | CAPN2 | calpain 2, (m/II) large subunit |
|  | BACE2 | beta-site APP-cleaving enzyme 2 |
|  | NDUFB4 | NADH dehydrogenase (ubiquinone) 1 beta subcomplex, 4, 15kDa |
|  | ATP5H | ATP synthase, H+ transporting, mitochondrial F0 complex, subunit d |
|  | NDUFB2 | NADH dehydrogenase (ubiquinone) 1 beta subcomplex, 2, 8kDa |
|  | NDUFB2 | NADH dehydrogenase (ubiquinone) 1 beta subcomplex, 2, 8kDa |
|  | APOE | apolipoprotein E |
|  | APOE | apolipoprotein E |
|  | NDUFS8 | NADH dehydrogenase (ubiquinone) Fe-S protein 8, 23kDa (NADH-coenzyme Q reductase) |
|  | COX7B | cytochrome c oxidase subunit VIIb |
|  | CYC1 | cytochrome c-1 |
|  | ATP5G1 | ATP synthase, H+ transporting, mitochondrial F0 complex, subunit C1 (subunit 9) |
|  | PPP3CA | protein phosphatase 3 (formerly 2B), catalytic subunit, alpha isoform |
|  | PPP3CA | protein phosphatase 3 (formerly 2B), catalytic subunit, alpha isoform |
|  | ADAM17 | ADAM metallopeptidase domain 17 |
|  | ADAM17 | ADAM metallopeptidase domain 17 |
|  | CALM1 | calmodulin 1 (phosphorylase kinase, delta) |
|  | CDK5R1 | cyclin-dependent kinase 5, regulatory subunit 1 (p35) |
|  | FAS | Fas (TNF receptor superfamily, member 6) |
|  | FAS | Fas (TNF receptor superfamily, member 6) |
|  | FAS | Fas (TNF receptor superfamily, member 6) |
|  | FAS | Fas (TNF receptor superfamily, member 6) |
|  | SDHB | succinate dehydrogenase complex, subunit B, iron sulfur (Ip) |
|  | ATP5G3 | ATP synthase, H+ transporting, mitochondrial F0 complex, subunit C3 (subunit 9) |
|  | ATP5C1 | ATP synthase, H+ transporting, mitochondrial F1 complex, gamma polypeptide 1 |
|  | COX7A2L | cytochrome c oxidase subunit VIIa polypeptide 2 like |
|  | ITPR1 | inositol 1,4,5-triphosphate receptor, type 1 |
|  | PPP3R1 | protein phosphatase 3 (formerly 2B), regulatory subunit B, alpha isoform |
|  | GNAQ | guanine nucleotide binding protein (G protein), q polypeptide |
|  | ATP5O | ATP synthase, H+ transporting, mitochondrial F1 complex, O subunit |
|  | MME | membrane metallo-endopeptidase |
| **KEGG pathway----p53 signaling pathway----04115** | **Gene Symbol** | **Gene Name** |
|  | LRDD | leucine-rich repeats and death domain containing |
|  | PPM1D | protein phosphatase 1D magnesium-dependent, delta isoform |
|  | RCHY1 | ring finger and CHY zinc finger domain containing 1 |
|  | RCHY1 | ring finger and CHY zinc finger domain containing 1 |
|  | CHEK1 | CHK1 checkpoint homolog (S. pombe) |
|  | CHEK1 | CHK1 checkpoint homolog (S. pombe) |
|  | CHEK1 | CHK1 checkpoint homolog (S. pombe) |
|  | CCNB1 | cyclin B1 |
|  | CCNB1 | cyclin B1 |
|  | CCNE1 | cyclin E1 |
|  | THBS1 | thrombospondin 1 |
|  | THBS1 | thrombospondin 1 |
|  | THBS1 | thrombospondin 1 |
|  | CCND1 | cyclin D1 |
|  | CCND1 | cyclin D1 |
|  | MDM2 | Mdm2 p53 binding protein homolog (mouse) |
|  | SIAH1 | seven in absentia homolog 1 (Drosophila) |
|  | SIAH1 | seven in absentia homolog 1 (Drosophila) |
|  | SIAH1 | seven in absentia homolog 1 (Drosophila) |
|  | PMAIP1 | phorbol-12-myristate-13-acetate-induced protein 1 |
|  | PMAIP1 | phorbol-12-myristate-13-acetate-induced protein 1 |
|  | BAX | BCL2-associated X protein |
|  | BAX | BCL2-associated X protein |
|  | GTSE1 | G-2 and S-phase expressed 1 |
|  | GTSE1 | G-2 and S-phase expressed 1 |
|  | STEAP3 | STEAP family member 3 |
|  | ATR | ataxia telangiectasia and Rad3 related |
|  | CCND2 | cyclin D2 |
|  | CDK6 | cyclin-dependent kinase 6 |
|  | CDK6 | cyclin-dependent kinase 6 |
|  | PERP | PERP, TP53 apoptosis effector |
|  | CCNG2 | cyclin G2 |
|  | IGFBP3 | insulin-like growth factor binding protein 3 |
|  | IGFBP3 | insulin-like growth factor binding protein 3 |
|  | DDB2 | damage-specific DNA binding protein 2, 48kDa |
|  | CDK4 | cyclin-dependent kinase 4 |
|  | TNFRSF10B | tumor necrosis factor receptor superfamily, member 10b |
|  | RRM2 | ribonucleotide reductase M2 |
|  | CDC2 | cell division cycle 2, G1 to S and G2 to M |
|  | CDC2 | cell division cycle 2, G1 to S and G2 to M |
|  | CDC2 | cell division cycle 2, G1 to S and G2 to M |
|  | TP53I3 | tumor protein p53 inducible protein 3 |
|  | FAS | Fas (TNF receptor superfamily, member 6) |
|  | FAS | Fas (TNF receptor superfamily, member 6) |
|  | FAS | Fas (TNF receptor superfamily, member 6) |
|  | FAS | Fas (TNF receptor superfamily, member 6) |
|  | RFWD2 | ring finger and WD repeat domain 2 |
|  | GADD45A | growth arrest and DNA-damage-inducible, alpha |
|  | GADD45B | growth arrest and DNA-damage-inducible, beta |
|  | GADD45B | growth arrest and DNA-damage-inducible, beta |
|  | SERPINB5 | serpin peptidase inhibitor, clade B (ovalbumin), member 5 |
|  | SERPINE1 | serpin peptidase inhibitor, clade E (nexin, plasminogen activator inhibitor type 1), member 1 |
|  | SERPINE1 | serpin peptidase inhibitor, clade E (nexin, plasminogen activator inhibitor type 1), member 1 |
|  | SERPINE1 | serpin peptidase inhibitor, clade E (nexin, plasminogen activator inhibitor type 1), member 1 |
|  | CCNB2 | cyclin B2 |
|  | CDK2 | cyclin-dependent kinase 2 |
|  | ZMAT3 | zinc finger, matrin type 3 |
|  | CDKN2A | cyclin-dependent kinase inhibitor 2A (melanoma, p16, inhibits CDK4) |
|  | SFN | stratifin |
|  | SFN | stratifin |
| **KEGG pathway----TGF-beta signaling pathway----04350** | **Gene Symbol** | **Gene Name** |
|  | DCN | decorin |
|  | DCN | decorin |
|  | DCN | decorin |
|  | DCN | decorin |
|  | DCN | decorin |
|  | LTBP1 | latent transforming growth factor beta binding protein 1 |
|  | LTBP1 | latent transforming growth factor beta binding protein 1 |
|  | ACVR1 | activin A receptor, type I |
|  | ID1 | inhibitor of DNA binding 1, dominant negative helix-loop-helix protein |
|  | BMPR2 | bone morphogenetic protein receptor, type II (serine/threonine kinase) |
|  | PPP2CB | protein phosphatase 2 (formerly 2A), catalytic subunit, beta isoform |
|  | ID2 | inhibitor of DNA binding 2, dominant negative helix-loop-helix protein |
|  | CUL1 | cullin 1 |
|  | ID4 | inhibitor of DNA binding 4, dominant negative helix-loop-helix protein |
|  | TGFBR1 | transforming growth factor, beta receptor 1 |
|  | TGFBR1 | transforming growth factor, beta receptor 1 |
|  | RBL2 | retinoblastoma-like 2 (p130) |
|  | PPP2R1B | protein phosphatase 2 (formerly 2A), regulatory subunit A, beta isoform |
|  | PPP2R1B | protein phosphatase 2 (formerly 2A), regulatory subunit A, beta isoform |
|  | PPP2R1B | protein phosphatase 2 (formerly 2A), regulatory subunit A, beta isoform |
|  | THBS1 | thrombospondin 1 |
|  | THBS1 | thrombospondin 1 |
|  | THBS1 | thrombospondin 1 |
|  | BMP2 | bone morphogenetic protein 2 |
|  | BMP7 | bone morphogenetic protein 7 |
|  | BMP7 | bone morphogenetic protein 7 |
|  | MYC | v-myc myelocytomatosis viral oncogene homolog (avian) |
|  | ACVR2A | activin A receptor, type IIA |
|  | SMAD6 | SMAD family member 6 |
|  | ROCK2 | Rho-associated, coiled-coil containing protein kinase 2 |
|  | INHBA | inhibin, beta A |
|  | SP1 | Sp1 transcription factor |
|  | TGFB1 | transforming growth factor, beta 1 |
|  | RHOA | ras homolog gene family, member A |
|  | RHOA | ras homolog gene family, member A |
|  | THBS2 | thrombospondin 2 |
|  | RBX1 | ring-box 1 |
|  | ACVR1B | activin A receptor, type IB |
|  | ACVR2B | activin A receptor, type IIB |
|  | SMURF2 | SMAD specific E3 ubiquitin protein ligase 2 |
|  | SMURF2 | SMAD specific E3 ubiquitin protein ligase 2 |
|  | PITX2 | paired-like homeodomain 2 |
|  | SMAD3 | SMAD family member 3 |
|  | SMAD3 | SMAD family member 3 |
|  | SMAD3 | SMAD family member 3 |
|  | ID3 | inhibitor of DNA binding 3, dominant negative helix-loop-helix protein |
|  | SMAD2 | SMAD family member 2 |
|  | SMAD2 | SMAD family member 2 |
|  | SMAD2 | SMAD family member 2 |
|  | TFDP1 | transcription factor Dp-1 |
|  | SKP1 | S-phase kinase-associated protein 1 |
|  | SKP1 | S-phase kinase-associated protein 1 |
|  | RPS6KB1 | ribosomal protein S6 kinase, 70kDa, polypeptide 1 |
|  | ROCK1 | Rho-associated, coiled-coil containing protein kinase 1 |
|  | ROCK1 | Rho-associated, coiled-coil containing protein kinase 1 |
|  | FST | follistatin |
|  | BMPR1A | bone morphogenetic protein receptor, type IA |
|  | SMURF1 | SMAD specific E3 ubiquitin protein ligase 1 |
|  | MAPK1 | mitogen-activated protein kinase 1 |
|  | MAPK1 | mitogen-activated protein kinase 1 |
|  | SMAD4 | SMAD family member 4 |
|  | SMAD4 | SMAD family member 4 |
| **KEGG pathway----ECM-receptor interaction----04512** | **Gene Symbol** | **Gene Name** |
|  | FN1 | fibronectin 1 |
|  | FN1 | fibronectin 1 |
|  | FN1 | fibronectin 1 |
|  | FN1 | fibronectin 1 |
|  | AGRN | agrin |
|  | AGRN | agrin |
|  | SDC4 | syndecan 4 |
|  | SDC2 | syndecan 2 |
|  | SDC2 | syndecan 2 |
|  | COL11A1 | collagen, type XI, alpha 1 |
|  | COL11A1 | collagen, type XI, alpha 1 |
|  | SDC1 | syndecan 1 |
|  | SDC1 | syndecan 1 |
|  | ITGB5 | integrin, beta 5 |
|  | ITGB5 | integrin, beta 5 |
|  | COL3A1 | collagen, type III, alpha 1 |
|  | COL3A1 | collagen, type III, alpha 1 |
|  | COL3A1 | collagen, type III, alpha 1 |
|  | TNC | tenascin C |
|  | COL6A3 | collagen, type VI, alpha 3 |
|  | THBS1 | thrombospondin 1 |
|  | THBS1 | thrombospondin 1 |
|  | THBS1 | thrombospondin 1 |
|  | LAMB3 | laminin, beta 3 |
|  | SPP1 | secreted phosphoprotein 1 |
|  | LAMA3 | laminin, alpha 3 |
|  | ITGA3 | integrin, alpha 3 (antigen CD49C, alpha 3 subunit of VLA-3 receptor) |
|  | ITGB8 | integrin, beta 8 |
|  | COL5A1 | collagen, type V, alpha 1 |
|  | COL5A1 | collagen, type V, alpha 1 |
|  | COL5A1 | collagen, type V, alpha 1 |
|  | COL6A1 | collagen, type VI, alpha 1 |
|  | COL4A1 | collagen, type IV, alpha 1 |
|  | CD44 | CD44 molecule (Indian blood group) |
|  | CD44 | CD44 molecule (Indian blood group) |
|  | CD44 | CD44 molecule (Indian blood group) |
|  | CD44 | CD44 molecule (Indian blood group) |
|  | CD44 | CD44 molecule (Indian blood group) |
|  | CD44 | CD44 molecule (Indian blood group) |
|  | ITGB6 | integrin, beta 6 |
|  | LAMA4 | laminin, alpha 4 |
|  | LAMA4 | laminin, alpha 4 |
|  | HMMR | hyaluronan-mediated motility receptor (RHAMM) |
|  | HMMR | hyaluronan-mediated motility receptor (RHAMM) |
|  | COL6A2 | collagen, type VI, alpha 2 |
|  | COL6A2 | collagen, type VI, alpha 2 |
|  | COL5A2 | collagen, type V, alpha 2 |
|  | LAMC1 | laminin, gamma 1 (formerly LAMB2) |
|  | THBS2 | thrombospondin 2 |
|  | COL1A1 | collagen, type I, alpha 1 |
|  | COL1A1 | collagen, type I, alpha 1 |
|  | ITGA5 | integrin, alpha 5 (fibronectin receptor, alpha polypeptide) |
|  | CD36 | CD36 molecule (thrombospondin receptor) |
|  | CD47 | CD47 molecule |
|  | LAMB1 | laminin, beta 1 |
|  | LAMB1 | laminin, beta 1 |
|  | ITGA2 | integrin, alpha 2 (CD49B, alpha 2 subunit of VLA-2 receptor) |
|  | ITGB1 | integrin, beta 1 (fibronectin receptor, beta polypeptide, antigen CD29 includes MDF2, MSK12) |
|  | ITGB1 | integrin, beta 1 (fibronectin receptor, beta polypeptide, antigen CD29 includes MDF2, MSK12) |
|  | VWF | von Willebrand factor |
|  | COL1A2 | collagen, type I, alpha 2 |
|  | COL1A2 | collagen, type I, alpha 2 |
|  | COL1A2 | collagen, type I, alpha 2 |
|  | ITGAV | integrin, alpha V (vitronectin receptor, alpha polypeptide, antigen CD51) |
|  | ITGB4 | integrin, beta 4 |
|  | LAMC2 | laminin, gamma 2 |
|  | LAMA5 | laminin, alpha 5 |
| **KEGG pathway----Proteasome----03050** | **Gene Symbol** | **Gene Name** |
|  | PSMB1 | proteasome (prosome, macropain) subunit, beta type, 1 |
|  | PSMA3 | proteasome (prosome, macropain) subunit, alpha type, 3 |
|  | PSMB4 | proteasome (prosome, macropain) subunit, beta type, 4 |
|  | PSMB4 | proteasome (prosome, macropain) subunit, beta type, 4 |
|  | PSMB4 | proteasome (prosome, macropain) subunit, beta type, 4 |
|  | PSMA2 | proteasome (prosome, macropain) subunit, alpha type, 2 |
|  | PSMC3 | proteasome (prosome, macropain) 26S subunit, ATPase, 3 |
|  | PSMB7 | proteasome (prosome, macropain) subunit, beta type, 7 |
|  | PSMD7 | proteasome (prosome, macropain) 26S subunit, non-ATPase, 7 |
|  | PSMA1 | proteasome (prosome, macropain) subunit, alpha type, 1 |
|  | POMP | proteasome maturation protein |
|  | PSMD4 | proteasome (prosome, macropain) 26S subunit, non-ATPase, 4 |
|  | PSMB9 | proteasome (prosome, macropain) subunit, beta type, 9 (large multifunctional peptidase 2) |
|  | PSMB3 | proteasome (prosome, macropain) subunit, beta type, 3 |
|  | PSMC4 | proteasome (prosome, macropain) 26S subunit, ATPase, 4 |
|  | PSMD2 | proteasome (prosome, macropain) 26S subunit, non-ATPase, 2 |
|  | PSMB10 | proteasome (prosome, macropain) subunit, beta type, 10 |
|  | PSMD1 | proteasome (prosome, macropain) 26S subunit, non-ATPase, 1 |
|  | PSMD1 | proteasome (prosome, macropain) 26S subunit, non-ATPase, 1 |
|  | PSMC6 | proteasome (prosome, macropain) 26S subunit, ATPase, 6 |
|  | PSMD8 | proteasome (prosome, macropain) 26S subunit, non-ATPase, 8 |
|  | PSMA7 | proteasome (prosome, macropain) subunit, alpha type, 7 |
|  | PSMC2 | proteasome (prosome, macropain) 26S subunit, ATPase, 2 |
|  | PSMC2 | proteasome (prosome, macropain) 26S subunit, ATPase, 2 |
|  | PSME4 | proteasome (prosome, macropain) activator subunit 4 |
|  | PSMD14 | proteasome (prosome, macropain) 26S subunit, non-ATPase, 14 |
|  | PSMB2 | proteasome (prosome, macropain) subunit, beta type, 2 |
|  | PSMD11 | proteasome (prosome, macropain) 26S subunit, non-ATPase, 11 |
|  | PSME3 | proteasome (prosome, macropain) activator subunit 3 (PA28 gamma; Ki) |
|  | PSME3 | proteasome (prosome, macropain) activator subunit 3 (PA28 gamma; Ki) |
|  | PSMD6 | proteasome (prosome, macropain) 26S subunit, non-ATPase, 6 |
|  | PSMA5 | proteasome (prosome, macropain) subunit, alpha type, 5 |
|  | PSMD12 | proteasome (prosome, macropain) 26S subunit, non-ATPase, 12 |
|  | PSMD12 | proteasome (prosome, macropain) 26S subunit, non-ATPase, 12 |
|  | PSMB6 | proteasome (prosome, macropain) subunit, beta type, 6 |
| **KEGG pathway----Wnt signaling pathway----04310** | **Gene Symbol** | **Gene Name** |
|  | MAPK8 | mitogen-activated protein kinase 8 |
|  | VANGL1 | vang-like 1 (van gogh, Drosophila) |
|  | WNT2 | wingless-type MMTV integration site family member 2 |
|  | PPP2CB | protein phosphatase 2 (formerly 2A), catalytic subunit, beta isoform |
|  | CSNK2A1 | casein kinase 2, alpha 1 polypeptide |
|  | CSNK2A1 | casein kinase 2, alpha 1 polypeptide |
|  | NLK | nemo-like kinase |
|  | CUL1 | cullin 1 |
|  | APC | adenomatous polyposis coli |
|  | CTBP2 | C-terminal binding protein 2 |
|  | CTBP2 | C-terminal binding protein 2 |
|  | CTBP2 | C-terminal binding protein 2 |
|  | PPP3CB | protein phosphatase 3 (formerly 2B), catalytic subunit, beta isoform |
|  | FZD2 | frizzled homolog 2 (Drosophila) |
|  | PRKCA | protein kinase C, alpha |
|  | CSNK1A1 | casein kinase 1, alpha 1 |
|  | CSNK1A1 | casein kinase 1, alpha 1 |
|  | NFAT5 | nuclear factor of activated T-cells 5, tonicity-responsive |
|  | NFAT5 | nuclear factor of activated T-cells 5, tonicity-responsive |
|  | PPP2R1B | protein phosphatase 2 (formerly 2A), regulatory subunit A, beta isoform |
|  | PPP2R1B | protein phosphatase 2 (formerly 2A), regulatory subunit A, beta isoform |
|  | PPP2R1B | protein phosphatase 2 (formerly 2A), regulatory subunit A, beta isoform |
|  | WNT5A | wingless-type MMTV integration site family, member 5A |
|  | WNT5A | wingless-type MMTV integration site family, member 5A |
|  | CCND1 | cyclin D1 |
|  | CCND1 | cyclin D1 |
|  | JUN | jun oncogene |
|  | JUN | jun oncogene |
|  | JUN | jun oncogene |
|  | RAC2 | ras-related C3 botulinum toxin substrate 2 (rho family, small GTP binding protein Rac2) |
|  | NFATC3 | nuclear factor of activated T-cells, cytoplasmic, calcineurin-dependent 3 |
|  | SIAH1 | seven in absentia homolog 1 (Drosophila) |
|  | SIAH1 | seven in absentia homolog 1 (Drosophila) |
|  | SIAH1 | seven in absentia homolog 1 (Drosophila) |
|  | WNT10A | wingless-type MMTV integration site family, member 10A |
|  | MYC | v-myc myelocytomatosis viral oncogene homolog (avian) |
|  | MAPK9 | mitogen-activated protein kinase 9 |
|  | MAP3K7 | mitogen-activated protein kinase kinase kinase 7 |
|  | MAP3K7 | mitogen-activated protein kinase kinase kinase 7 |
|  | CCND2 | cyclin D2 |
|  | ROCK2 | Rho-associated, coiled-coil containing protein kinase 2 |
|  | PRKACB | protein kinase, cAMP-dependent, catalytic, beta |
|  | CAMK2G | calcium/calmodulin-dependent protein kinase II gamma |
|  | CSNK2A2 | casein kinase 2, alpha prime polypeptide |
|  | FZD1 | frizzled homolog 1 (Drosophila) |
|  | PPP3CA | protein phosphatase 3 (formerly 2B), catalytic subunit, alpha isoform |
|  | PPP3CA | protein phosphatase 3 (formerly 2B), catalytic subunit, alpha isoform |
|  | RHOA | ras homolog gene family, member A |
|  | RHOA | ras homolog gene family, member A |
|  | RBX1 | ring-box 1 |
|  | SFRP2 | secreted frizzled-related protein 2 |
|  | TBL1XR1 | transducin (beta)-like 1 X-linked receptor 1 |
|  | TBL1XR1 | transducin (beta)-like 1 X-linked receptor 1 |
|  | TBL1XR1 | transducin (beta)-like 1 X-linked receptor 1 |
|  | TBL1XR1 | transducin (beta)-like 1 X-linked receptor 1 |
|  | FZD3 | frizzled homolog 3 (Drosophila) |
|  | TCF7L2 | transcription factor 7-like 2 (T-cell specific, HMG-box) |
|  | TCF7L2 | transcription factor 7-like 2 (T-cell specific, HMG-box) |
|  | TCF7L2 | transcription factor 7-like 2 (T-cell specific, HMG-box) |
|  | TCF7L2 | transcription factor 7-like 2 (T-cell specific, HMG-box) |
|  | TCF7L2 | transcription factor 7-like 2 (T-cell specific, HMG-box) |
|  | TCF7L2 | transcription factor 7-like 2 (T-cell specific, HMG-box) |
|  | FZD7 | frizzled homolog 7 (Drosophila) |
|  | FZD7 | frizzled homolog 7 (Drosophila) |
|  | SMAD3 | SMAD family member 3 |
|  | SMAD3 | SMAD family member 3 |
|  | SMAD3 | SMAD family member 3 |
|  | CAMK2D | calcium/calmodulin-dependent protein kinase II delta |
|  | SMAD2 | SMAD family member 2 |
|  | SMAD2 | SMAD family member 2 |
|  | SMAD2 | SMAD family member 2 |
|  | RUVBL1 | RuvB-like 1 (E. coli) |
|  | SFRP4 | secreted frizzled-related protein 4 |
|  | PPP3R1 | protein phosphatase 3 (formerly 2B), regulatory subunit B, alpha isoform |
|  | SKP1 | S-phase kinase-associated protein 1 |
|  | SKP1 | S-phase kinase-associated protein 1 |
|  | PPP2R5C | protein phosphatase 2, regulatory subunit B', gamma isoform |
|  | PPP2R5C | protein phosphatase 2, regulatory subunit B', gamma isoform |
|  | PPP2R5C | protein phosphatase 2, regulatory subunit B', gamma isoform |
|  | ROCK1 | Rho-associated, coiled-coil containing protein kinase 1 |
|  | ROCK1 | Rho-associated, coiled-coil containing protein kinase 1 |
|  | CACYBP | calcyclin binding protein |
|  | CACYBP | calcyclin binding protein |
|  | CACYBP | calcyclin binding protein |
|  | TBL1X | transducin (beta)-like 1X-linked |
|  | MAPK10 | mitogen-activated protein kinase 10 |
|  | CTBP1 | C-terminal binding protein 1 |
|  | SMAD4 | SMAD family member 4 |
|  | SMAD4 | SMAD family member 4 |
|  | BTRC | beta-transducin repeat containing |
| **KEGG pathway----Oxidative phosphorylation----00190** | **Gene Symbol** | **Gene Name** |
|  | COX15 | COX15 homolog, cytochrome c oxidase assembly protein (yeast) |
|  | NDUFC2 | NADH dehydrogenase (ubiquinone) 1, subcomplex unknown, 2, 14.5kDa |
|  | NDUFB6 | NADH dehydrogenase (ubiquinone) 1 beta subcomplex, 6, 17kDa |
|  | UQCRB | ubiquinol-cytochrome c reductase binding protein |
|  | SDHC | succinate dehydrogenase complex, subunit C, integral membrane protein, 15kDa |
|  | SDHC | succinate dehydrogenase complex, subunit C, integral membrane protein, 15kDa |
|  | ATP6V1A | ATPase, H+ transporting, lysosomal 70kDa, V1 subunit A |
|  | ATP6V1A | ATPase, H+ transporting, lysosomal 70kDa, V1 subunit A |
|  | UCRC | ubiquinol-cytochrome c reductase complex (7.2 kD) |
|  | NDUFS6 | NADH dehydrogenase (ubiquinone) Fe-S protein 6, 13kDa (NADH-coenzyme Q reductase) |
|  | NDUFB5 | NADH dehydrogenase (ubiquinone) 1 beta subcomplex, 5, 16kDa |
|  | NDUFA4 | NADH dehydrogenase (ubiquinone) 1 alpha subcomplex, 4, 9kDa |
|  | NDUFAB1 | NADH dehydrogenase (ubiquinone) 1, alpha/beta subcomplex, 1, 8kDa |
|  | COX4I1 | cytochrome c oxidase subunit IV isoform 1 |
|  | hCG_1776980 | hCG1776980 |
|  | ATP6V1B2 | ATPase, H+ transporting, lysosomal 56/58kDa, V1 subunit B2 |
|  | NDUFS7 | NADH dehydrogenase (ubiquinone) Fe-S protein 7, 20kDa (NADH-coenzyme Q reductase) |
|  | NDUFS4 | NADH dehydrogenase (ubiquinone) Fe-S protein 4, 18kDa (NADH-coenzyme Q reductase) |
|  | NDUFB3 | NADH dehydrogenase (ubiquinone) 1 beta subcomplex, 3, 12kDa |
|  | UQCRFS1 | ubiquinol-cytochrome c reductase, Rieske iron-sulfur polypeptide 1 |
|  | ATP6V0B | ATPase, H+ transporting, lysosomal 21kDa, V0 subunit b |
|  | NDUFB4 | NADH dehydrogenase (ubiquinone) 1 beta subcomplex, 4, 15kDa |
|  | ATP5H | ATP synthase, H+ transporting, mitochondrial F0 complex, subunit d |
|  | NDUFB2 | NADH dehydrogenase (ubiquinone) 1 beta subcomplex, 2, 8kDa |
|  | NDUFB2 | NADH dehydrogenase (ubiquinone) 1 beta subcomplex, 2, 8kDa |
|  | UQCRC2 | ubiquinol-cytochrome c reductase core protein II |
|  | NDUFB8 | NADH dehydrogenase (ubiquinone) 1 beta subcomplex, 8, 19kDa |
|  | NDUFS8 | NADH dehydrogenase (ubiquinone) Fe-S protein 8, 23kDa (NADH-coenzyme Q reductase) |
|  | COX7B | cytochrome c oxidase subunit VIIb |
|  | CYC1 | cytochrome c-1 |
|  | NDUFA6 | NADH dehydrogenase (ubiquinone) 1 alpha subcomplex, 6, 14kDa |
|  | ATP6V1E1 | ATPase, H+ transporting, lysosomal 31kDa, V1 subunit E1 |
|  | NDUFS2 | NADH dehydrogenase (ubiquinone) Fe-S protein 2, 49kDa (NADH-coenzyme Q reductase) |
|  | ATP6V1G1 | ATPase, H+ transporting, lysosomal 13kDa, V1 subunit G1 |
|  | ATP5G1 | ATP synthase, H+ transporting, mitochondrial F0 complex, subunit C1 (subunit 9) |
|  | COX7C | cytochrome c oxidase subunit VIIc |
|  | COX11 | COX11 homolog, cytochrome c oxidase assembly protein (yeast) |
|  | ATP5F1 | ATP synthase, H+ transporting, mitochondrial F0 complex, subunit B1 |
|  | SDHD | succinate dehydrogenase complex, subunit D, integral membrane protein |
|  | COX17 | COX17 cytochrome c oxidase assembly homolog (S. cerevisiae) |
|  | SDHB | succinate dehydrogenase complex, subunit B, iron sulfur (Ip) |
|  | TCIRG1 | T-cell, immune regulator 1, ATPase, H+ transporting, lysosomal V0 subunit A3 |
|  | ATP5G3 | ATP synthase, H+ transporting, mitochondrial F0 complex, subunit C3 (subunit 9) |
|  | ATP5C1 | ATP synthase, H+ transporting, mitochondrial F1 complex, gamma polypeptide 1 |
|  | NDUFA11 | NADH dehydrogenase (ubiquinone) 1 alpha subcomplex, 11, 14.7kDa |
|  | COX7A2L | cytochrome c oxidase subunit VIIa polypeptide 2 like |
|  | PPA2 | pyrophosphatase (inorganic) 2 |
|  | PPA2 | pyrophosphatase (inorganic) 2 |
|  | PPA2 | pyrophosphatase (inorganic) 2 |
|  | UQCRH | ubiquinol-cytochrome c reductase hinge protein |
|  | ATP6V1C1 | ATPase, H+ transporting, lysosomal 42kDa, V1 subunit C1 |
|  | ATP6V1C1 | ATPase, H+ transporting, lysosomal 42kDa, V1 subunit C1 |
|  | NDUFB7 | NADH dehydrogenase (ubiquinone) 1 beta subcomplex, 7, 18kDa |
|  | NDUFA8 | NADH dehydrogenase (ubiquinone) 1 alpha subcomplex, 8, 19kDa |
|  | ATP5O | ATP synthase, H+ transporting, mitochondrial F1 complex, O subunit |
|  | ATP6V0E1 | ATPase, H+ transporting, lysosomal 9kDa, V0 subunit e1 |
|  | ATP6V0E1 | ATPase, H+ transporting, lysosomal 9kDa, V0 subunit e1 |
| **KEGG pathway----Chronic myeloid leukemia----05220** | **Gene Symbol** | **Gene Name** |
|  | BRAF | v-raf murine sarcoma viral oncogene homolog B1 |
|  | MAP2K1 | mitogen-activated protein kinase kinase 1 |
|  | TGFBR1 | transforming growth factor, beta receptor 1 |
|  | TGFBR1 | transforming growth factor, beta receptor 1 |
|  | CTBP2 | C-terminal binding protein 2 |
|  | CTBP2 | C-terminal binding protein 2 |
|  | CTBP2 | C-terminal binding protein 2 |
|  | BCL2L1 | BCL2-like 1 |
|  | SHC2 | SHC (Src homology 2 domain containing) transforming protein 2 |
|  | CRKL | v-crk sarcoma virus CT10 oncogene homolog (avian)-like |
|  | CCND1 | cyclin D1 |
|  | CCND1 | cyclin D1 |
|  | MDM2 | Mdm2 p53 binding protein homolog (mouse) |
|  | SHC1 | SHC (Src homology 2 domain containing) transforming protein 1 |
|  | MYC | v-myc myelocytomatosis viral oncogene homolog (avian) |
|  | IKBKB | inhibitor of kappa light polypeptide gene enhancer in B-cells, kinase beta |
|  | IKBKB | inhibitor of kappa light polypeptide gene enhancer in B-cells, kinase beta |
|  | PIK3CA | phosphoinositide-3-kinase, catalytic, alpha polypeptide |
|  | CDKN1B | cyclin-dependent kinase inhibitor 1B (p27, Kip1) |
|  | CDK6 | cyclin-dependent kinase 6 |
|  | CDK6 | cyclin-dependent kinase 6 |
|  | GAB2 | GRB2-associated binding protein 2 |
|  | TGFB1 | transforming growth factor, beta 1 |
|  | BCR | breakpoint cluster region |
|  | NRAS | neuroblastoma RAS viral (v-ras) oncogene homolog |
|  | SOS1 | son of sevenless homolog 1 (Drosophila) |
|  | CDK4 | cyclin-dependent kinase 4 |
|  | ACVR1B | activin A receptor, type IB |
|  | E2F3 | E2F transcription factor 3 |
|  | CBLB | Cas-Br-M (murine) ecotropic retroviral transforming sequence b |
|  | MAP2K2 | mitogen-activated protein kinase kinase 2 |
|  | PIK3CB | phosphoinositide-3-kinase, catalytic, beta polypeptide |
|  | GRB2 | growth factor receptor-bound protein 2 |
|  | SMAD3 | SMAD family member 3 |
|  | SMAD3 | SMAD family member 3 |
|  | SMAD3 | SMAD family member 3 |
|  | AKT3 | v-akt murine thymoma viral oncogene homolog 3 (protein kinase B, gamma) |
|  | AKT3 | v-akt murine thymoma viral oncogene homolog 3 (protein kinase B, gamma) |
|  | AKT3 | v-akt murine thymoma viral oncogene homolog 3 (protein kinase B, gamma) |
|  | AKT3 | v-akt murine thymoma viral oncogene homolog 3 (protein kinase B, gamma) |
|  | NFKBIA | nuclear factor of kappa light polypeptide gene enhancer in B-cells inhibitor, alpha |
|  | RUNX1 | runt-related transcription factor 1 |
|  | MAPK1 | mitogen-activated protein kinase 1 |
|  | MAPK1 | mitogen-activated protein kinase 1 |
|  | SMAD4 | SMAD family member 4 |
|  | SMAD4 | SMAD family member 4 |
|  | CTBP1 | C-terminal binding protein 1 |
|  | CDKN2A | cyclin-dependent kinase inhibitor 2A (melanoma, p16, inhibits CDK4) |
| **KEGG pathway----Parkinson's disease----05012** | **Gene Symbol** | **Gene Name** |
|  | NDUFC2 | NADH dehydrogenase (ubiquinone) 1, subcomplex unknown, 2, 14.5kDa |
|  | PINK1 | PTEN induced putative kinase 1 |
|  | NDUFB6 | NADH dehydrogenase (ubiquinone) 1 beta subcomplex, 6, 17kDa |
|  | UQCRB | ubiquinol-cytochrome c reductase binding protein |
|  | SDHC | succinate dehydrogenase complex, subunit C, integral membrane protein, 15kDa |
|  | SDHC | succinate dehydrogenase complex, subunit C, integral membrane protein, 15kDa |
|  | UCRC | ubiquinol-cytochrome c reductase complex (7.2 kD) |
|  | NDUFS6 | NADH dehydrogenase (ubiquinone) Fe-S protein 6, 13kDa (NADH-coenzyme Q reductase) |
|  | NDUFB5 | NADH dehydrogenase (ubiquinone) 1 beta subcomplex, 5, 16kDa |
|  | NDUFA4 | NADH dehydrogenase (ubiquinone) 1 alpha subcomplex, 4, 9kDa |
|  | NDUFAB1 | NADH dehydrogenase (ubiquinone) 1, alpha/beta subcomplex, 1, 8kDa |
|  | COX4I1 | cytochrome c oxidase subunit IV isoform 1 |
|  | hCG_1776980 | hCG1776980 |
|  | NDUFS7 | NADH dehydrogenase (ubiquinone) Fe-S protein 7, 20kDa (NADH-coenzyme Q reductase) |
|  | NDUFS4 | NADH dehydrogenase (ubiquinone) Fe-S protein 4, 18kDa (NADH-coenzyme Q reductase) |
|  | NDUFB3 | NADH dehydrogenase (ubiquinone) 1 beta subcomplex, 3, 12kDa |
|  | UQCRFS1 | ubiquinol-cytochrome c reductase, Rieske iron-sulfur polypeptide 1 |
|  | NDUFB4 | NADH dehydrogenase (ubiquinone) 1 beta subcomplex, 4, 15kDa |
|  | PPID | peptidylprolyl isomerase D |
|  | PPID | peptidylprolyl isomerase D |
|  | PPID | peptidylprolyl isomerase D |
|  | UBA7 | ubiquitin-like modifier activating enzyme 7 |
|  | UBA7 | ubiquitin-like modifier activating enzyme 7 |
|  | ATP5H | ATP synthase, H+ transporting, mitochondrial F0 complex, subunit d |
|  | NDUFB2 | NADH dehydrogenase (ubiquinone) 1 beta subcomplex, 2, 8kDa |
|  | NDUFB2 | NADH dehydrogenase (ubiquinone) 1 beta subcomplex, 2, 8kDa |
|  | UQCRC2 | ubiquinol-cytochrome c reductase core protein II |
|  | UBE2J1 | ubiquitin-conjugating enzyme E2, J1 (UBC6 homolog, yeast) |
|  | UBE2J1 | ubiquitin-conjugating enzyme E2, J1 (UBC6 homolog, yeast) |
|  | UBE2J1 | ubiquitin-conjugating enzyme E2, J1 (UBC6 homolog, yeast) |
|  | UBE2J1 | ubiquitin-conjugating enzyme E2, J1 (UBC6 homolog, yeast) |
|  | NDUFB8 | NADH dehydrogenase (ubiquinone) 1 beta subcomplex, 8, 19kDa |
|  | VDAC3 | voltage-dependent anion channel 3 |
|  | UBE2G1 | ubiquitin-conjugating enzyme E2G 1 (UBC7 homolog, yeast) |
|  | UBE2G1 | ubiquitin-conjugating enzyme E2G 1 (UBC7 homolog, yeast) |
|  | UBE2G1 | ubiquitin-conjugating enzyme E2G 1 (UBC7 homolog, yeast) |
|  | NDUFS8 | NADH dehydrogenase (ubiquinone) Fe-S protein 8, 23kDa (NADH-coenzyme Q reductase) |
|  | CYC1 | cytochrome c-1 |
|  | COX7B | cytochrome c oxidase subunit VIIb |
|  | NDUFA6 | NADH dehydrogenase (ubiquinone) 1 alpha subcomplex, 6, 14kDa |
|  | NDUFS2 | NADH dehydrogenase (ubiquinone) Fe-S protein 2, 49kDa (NADH-coenzyme Q reductase) |
|  | HTRA2 | HtrA serine peptidase 2 |
|  | HTRA2 | HtrA serine peptidase 2 |
|  | UBE2G2 | ubiquitin-conjugating enzyme E2G 2 (UBC7 homolog, yeast) |
|  | ATP5G1 | ATP synthase, H+ transporting, mitochondrial F0 complex, subunit C1 (subunit 9) |
|  | COX7C | cytochrome c oxidase subunit VIIc |
|  | UBE2L3 | ubiquitin-conjugating enzyme E2L 3 |
|  | UBE2L3 | ubiquitin-conjugating enzyme E2L 3 |
|  | UBE2L3 | ubiquitin-conjugating enzyme E2L 3 |
|  | LRRK2 | leucine-rich repeat kinase 2 |
|  | VDAC1 | voltage-dependent anion channel 1 |
|  | SLC25A5 | solute carrier family 25 (mitochondrial carrier; adenine nucleotide translocator), member 5 |
|  | ATP5F1 | ATP synthase, H+ transporting, mitochondrial F0 complex, subunit B1 |
|  | SDHD | succinate dehydrogenase complex, subunit D, integral membrane protein |
|  | SDHB | succinate dehydrogenase complex, subunit B, iron sulfur (Ip) |
|  | ATP5G3 | ATP synthase, H+ transporting, mitochondrial F0 complex, subunit C3 (subunit 9) |
|  | ATP5C1 | ATP synthase, H+ transporting, mitochondrial F1 complex, gamma polypeptide 1 |
|  | COX7A2L | cytochrome c oxidase subunit VIIa polypeptide 2 like |
|  | UQCRH | ubiquinol-cytochrome c reductase hinge protein |
|  | NDUFB7 | NADH dehydrogenase (ubiquinone) 1 beta subcomplex, 7, 18kDa |
|  | NDUFA8 | NADH dehydrogenase (ubiquinone) 1 alpha subcomplex, 8, 19kDa |
|  | ATP5O | ATP synthase, H+ transporting, mitochondrial F1 complex, O subunit |
| **KEGG pathway----Small cell lung cancer----05222** | **Gene Symbol** | **Gene Name** |
|  | FN1 | fibronectin 1 |
|  | FN1 | fibronectin 1 |
|  | FN1 | fibronectin 1 |
|  | FN1 | fibronectin 1 |
|  | RXRA | retinoid X receptor, alpha |
|  | XIAP | X-linked inhibitor of apoptosis |
|  | XIAP | X-linked inhibitor of apoptosis |
|  | BCL2L1 | BCL2-like 1 |
|  | BIRC2 | baculoviral IAP repeat-containing 2 |
|  | CCNE1 | cyclin E1 |
|  | LAMB3 | laminin, beta 3 |
|  | CCND1 | cyclin D1 |
|  | CCND1 | cyclin D1 |
|  | LAMA3 | laminin, alpha 3 |
|  | ITGA3 | integrin, alpha 3 (antigen CD49C, alpha 3 subunit of VLA-3 receptor) |
|  | MYC | v-myc myelocytomatosis viral oncogene homolog (avian) |
|  | IKBKB | inhibitor of kappa light polypeptide gene enhancer in B-cells, kinase beta |
|  | IKBKB | inhibitor of kappa light polypeptide gene enhancer in B-cells, kinase beta |
|  | PIK3CA | phosphoinositide-3-kinase, catalytic, alpha polypeptide |
|  | CKS1B | CDC28 protein kinase regulatory subunit 1B |
|  | CDKN1B | cyclin-dependent kinase inhibitor 1B (p27, Kip1) |
|  | COL4A1 | collagen, type IV, alpha 1 |
|  | CDK6 | cyclin-dependent kinase 6 |
|  | CDK6 | cyclin-dependent kinase 6 |
|  | LAMA4 | laminin, alpha 4 |
|  | LAMA4 | laminin, alpha 4 |
|  | BIRC3 | baculoviral IAP repeat-containing 3 |
|  | LAMC1 | laminin, gamma 1 (formerly LAMB2) |
|  | CDK4 | cyclin-dependent kinase 4 |
|  | E2F3 | E2F transcription factor 3 |
|  | SKP2 | S-phase kinase-associated protein 2 (p45) |
|  | SKP2 | S-phase kinase-associated protein 2 (p45) |
|  | PTK2 | PTK2 protein tyrosine kinase 2 |
|  | PTK2 | PTK2 protein tyrosine kinase 2 |
|  | PIK3CB | phosphoinositide-3-kinase, catalytic, beta polypeptide |
|  | PIAS3 | protein inhibitor of activated STAT, 3 |
|  | PTGS2 | prostaglandin-endoperoxide synthase 2 (prostaglandin G/H synthase and cyclooxygenase) |
|  | PTGS2 | prostaglandin-endoperoxide synthase 2 (prostaglandin G/H synthase and cyclooxygenase) |
|  | AKT3 | v-akt murine thymoma viral oncogene homolog 3 (protein kinase B, gamma) |
|  | AKT3 | v-akt murine thymoma viral oncogene homolog 3 (protein kinase B, gamma) |
|  | AKT3 | v-akt murine thymoma viral oncogene homolog 3 (protein kinase B, gamma) |
|  | AKT3 | v-akt murine thymoma viral oncogene homolog 3 (protein kinase B, gamma) |
|  | NFKBIA | nuclear factor of kappa light polypeptide gene enhancer in B-cells inhibitor, alpha |
|  | LAMB1 | laminin, beta 1 |
|  | LAMB1 | laminin, beta 1 |
|  | ITGA2 | integrin, alpha 2 (CD49B, alpha 2 subunit of VLA-2 receptor) |
|  | ITGB1 | integrin, beta 1 (fibronectin receptor, beta polypeptide, antigen CD29 includes MDF2, MSK12) |
|  | ITGB1 | integrin, beta 1 (fibronectin receptor, beta polypeptide, antigen CD29 includes MDF2, MSK12) |
|  | ITGAV | integrin, alpha V (vitronectin receptor, alpha polypeptide, antigen CD51) |
|  | TRAF5 | TNF receptor-associated factor 5 |
|  | CDK2 | cyclin-dependent kinase 2 |
|  | LAMC2 | laminin, gamma 2 |
|  | LAMA5 | laminin, alpha 5 |
| **KEGG pathway----Colorectal cancer----05210** | **Gene Symbol** | **Gene Name** |
|  | MAPK8 | mitogen-activated protein kinase 8 |
|  | BRAF | v-raf murine sarcoma viral oncogene homolog B1 |
|  | MAP2K1 | mitogen-activated protein kinase kinase 1 |
|  | RALGDS | ral guanine nucleotide dissociation stimulator |
|  | RALGDS | ral guanine nucleotide dissociation stimulator |
|  | APC | adenomatous polyposis coli |
|  | TGFBR1 | transforming growth factor, beta receptor 1 |
|  | TGFBR1 | transforming growth factor, beta receptor 1 |
|  | FZD2 | frizzled homolog 2 (Drosophila) |
|  | PDGFRA | platelet-derived growth factor receptor, alpha polypeptide |
|  | CCND1 | cyclin D1 |
|  | CCND1 | cyclin D1 |
|  | JUN | jun oncogene |
|  | JUN | jun oncogene |
|  | JUN | jun oncogene |
|  | RAC2 | ras-related C3 botulinum toxin substrate 2 (rho family, small GTP binding protein Rac2) |
|  | MYC | v-myc myelocytomatosis viral oncogene homolog (avian) |
|  | MAPK9 | mitogen-activated protein kinase 9 |
|  | BAX | BCL2-associated X protein |
|  | BAX | BCL2-associated X protein |
|  | PIK3CA | phosphoinositide-3-kinase, catalytic, alpha polypeptide |
|  | MSH2 | mutS homolog 2, colon cancer, nonpolyposis type 1 (E. coli) |
|  | MSH6 | mutS homolog 6 (E. coli) |
|  | MSH6 | mutS homolog 6 (E. coli) |
|  | PDGFRB | platelet-derived growth factor receptor, beta polypeptide |
|  | FZD1 | frizzled homolog 1 (Drosophila) |
|  | TGFB1 | transforming growth factor, beta 1 |
|  | SOS1 | son of sevenless homolog 1 (Drosophila) |
|  | ACVR1B | activin A receptor, type IB |
|  | FZD3 | frizzled homolog 3 (Drosophila) |
|  | FOS | FBJ murine osteosarcoma viral oncogene homolog |
|  | APPL1 | adaptor protein, phosphotyrosine interaction, PH domain and leucine zipper containing 1 |
|  | APPL1 | adaptor protein, phosphotyrosine interaction, PH domain and leucine zipper containing 1 |
|  | PIK3CB | phosphoinositide-3-kinase, catalytic, beta polypeptide |
|  | EGFR | epidermal growth factor receptor (erythroblastic leukemia viral (v-erb-b) oncogene homolog, avian) |
|  | EGFR | epidermal growth factor receptor (erythroblastic leukemia viral (v-erb-b) oncogene homolog, avian) |
|  | EGFR | epidermal growth factor receptor (erythroblastic leukemia viral (v-erb-b) oncogene homolog, avian) |
|  | TCF7L2 | transcription factor 7-like 2 (T-cell specific, HMG-box) |
|  | TCF7L2 | transcription factor 7-like 2 (T-cell specific, HMG-box) |
|  | TCF7L2 | transcription factor 7-like 2 (T-cell specific, HMG-box) |
|  | TCF7L2 | transcription factor 7-like 2 (T-cell specific, HMG-box) |
|  | TCF7L2 | transcription factor 7-like 2 (T-cell specific, HMG-box) |
|  | TCF7L2 | transcription factor 7-like 2 (T-cell specific, HMG-box) |
|  | FZD7 | frizzled homolog 7 (Drosophila) |
|  | FZD7 | frizzled homolog 7 (Drosophila) |
|  | GRB2 | growth factor receptor-bound protein 2 |
|  | SMAD3 | SMAD family member 3 |
|  | SMAD3 | SMAD family member 3 |
|  | SMAD3 | SMAD family member 3 |
|  | SMAD2 | SMAD family member 2 |
|  | SMAD2 | SMAD family member 2 |
|  | SMAD2 | SMAD family member 2 |
|  | BIRC5 | baculoviral IAP repeat-containing 5 |
|  | AKT3 | v-akt murine thymoma viral oncogene homolog 3 (protein kinase B, gamma) |
|  | AKT3 | v-akt murine thymoma viral oncogene homolog 3 (protein kinase B, gamma) |
|  | AKT3 | v-akt murine thymoma viral oncogene homolog 3 (protein kinase B, gamma) |
|  | AKT3 | v-akt murine thymoma viral oncogene homolog 3 (protein kinase B, gamma) |
|  | MAPK10 | mitogen-activated protein kinase 10 |
|  | MAPK1 | mitogen-activated protein kinase 1 |
|  | MAPK1 | mitogen-activated protein kinase 1 |
|  | SMAD4 | SMAD family member 4 |
|  | SMAD4 | SMAD family member 4 |
| **KEGG pathway----RNA degradation----03018** | **Gene Symbol** | **Gene Name** |
|  | C1D | C1D nuclear receptor co-repressor |
|  | ENO2 | enolase 2 (gamma, neuronal) |
|  | WDR61 | WD repeat domain 61 |
|  | WDR61 | WD repeat domain 61 |
|  | EXOSC3 | exosome component 3 |
|  | EXOSC3 | exosome component 3 |
|  | CNOT8 | CCR4-NOT transcription complex, subunit 8 |
|  | LSM7 | LSM7 homolog, U6 small nuclear RNA associated (S. cerevisiae) |
|  | LSM8 | LSM8 homolog, U6 small nuclear RNA associated (S. cerevisiae) |
|  | LSM4 | LSM4 homolog, U6 small nuclear RNA associated (S. cerevisiae) |
|  | EXOSC9 | exosome component 9 |
|  | CNOT7 | CCR4-NOT transcription complex, subunit 7 |
|  | PATL1 | protein associated with topoisomerase II homolog 1 (yeast) |
|  | PATL1 | protein associated with topoisomerase II homolog 1 (yeast) |
|  | DCP2 | DCP2 decapping enzyme homolog (S. cerevisiae) |
|  | PAPOLA | poly(A) polymerase alpha |
|  | PAPOLA | poly(A) polymerase alpha |
|  | PAPOLA | poly(A) polymerase alpha |
|  | RQCD1 | RCD1 required for cell differentiation1 homolog (S. pombe) |
|  | LSM5 | LSM5 homolog, U6 small nuclear RNA associated (S. cerevisiae) |
|  | LSM5 | LSM5 homolog, U6 small nuclear RNA associated (S. cerevisiae) |
|  | LSM6 | LSM6 homolog, U6 small nuclear RNA associated (S. cerevisiae) |
|  | LSM3 | LSM3 homolog, U6 small nuclear RNA associated (S. cerevisiae) |
|  | EXOSC5 | exosome component 5 |
|  | LSM2 | LSM2 homolog, U6 small nuclear RNA associated (S. cerevisiae) |
|  | TTC37 | tetratricopeptide repeat domain 37 |
|  | DCP1A | DCP1 decapping enzyme homolog A (S. cerevisiae) |
|  | XRN2 | 5'-3' exoribonuclease 2 |
|  | XRN2 | 5'-3' exoribonuclease 2 |
|  | XRN1 | 5'-3' exoribonuclease 1 |
|  | PAPOLG | poly(A) polymerase gamma |
|  | DIS3 | DIS3 mitotic control homolog (S. cerevisiae) |
|  | DIS3 | DIS3 mitotic control homolog (S. cerevisiae) |
|  | CNOT6L | CCR4-NOT transcription complex, subunit 6-like |
|  | HSPD1 | heat shock 60kDa protein 1 (chaperonin) |
|  | ENO1 | enolase 1, (alpha) |
|  | PNPT1 | polyribonucleotide nucleotidyltransferase 1 |
|  | MPHOSPH6 | M-phase phosphoprotein 6 |
| **KEGG pathway----N-Glycan biosynthesis----00510** | **Gene Symbol** | **Gene Name** |
|  | ALG6 | asparagine-linked glycosylation 6, alpha-1,3-glucosyltransferase homolog (S. cerevisiae) |
|  | DPM1 | dolichyl-phosphate mannosyltransferase polypeptide 1, catalytic subunit |
|  | MAN1A2 | mannosidase, alpha, class 1A, member 2 |
|  | MAN1B1 | mannosidase, alpha, class 1B, member 1 |
|  | MAN1B1 | mannosidase, alpha, class 1B, member 1 |
|  | MAN1C1 | mannosidase, alpha, class 1C, member 1 |
|  | MAN1C1 | mannosidase, alpha, class 1C, member 1 |
|  | DPM3 | dolichyl-phosphate mannosyltransferase polypeptide 3 |
|  | RFT1 | RFT1 homolog (S. cerevisiae) |
|  | MAN1A1 | mannosidase, alpha, class 1A, member 1 |
|  | ALG10 | asparagine-linked glycosylation 10, alpha-1,2-glucosyltransferase homolog (S. pombe) |
|  | ALG3 | asparagine-linked glycosylation 3, alpha-1,3- mannosyltransferase homolog (S. cerevisiae) |
|  | MGAT2 | mannosyl (alpha-1,6-)-glycoprotein beta-1,2-N-acetylglucosaminyltransferase |
|  | MGAT2 | mannosyl (alpha-1,6-)-glycoprotein beta-1,2-N-acetylglucosaminyltransferase |
|  | TUSC3 | tumor suppressor candidate 3 |
|  | TUSC3 | tumor suppressor candidate 3 |
|  | RPN1 | ribophorin I |
|  | DDOST | dolichyl-diphosphooligosaccharide-protein glycosyltransferase |
|  | DDOST | dolichyl-diphosphooligosaccharide-protein glycosyltransferase |
|  | MGAT1 | mannosyl (alpha-1,3-)-glycoprotein beta-1,2-N-acetylglucosaminyltransferase |
|  | STT3A | STT3, subunit of the oligosaccharyltransferase complex, homolog A (S. cerevisiae) |
|  | DAD1 | defender against cell death 1 |
|  | ALG13 | asparagine-linked glycosylation 13 homolog (S. cerevisiae) |
|  | ALG13 | asparagine-linked glycosylation 13 homolog (S. cerevisiae) |
|  | MGAT4A | mannosyl (alpha-1,3-)-glycoprotein beta-1,4-N-acetylglucosaminyltransferase, isozyme A |
|  | ALG14 | asparagine-linked glycosylation 14 homolog (S. cerevisiae) |
|  | MAN2A1 | mannosidase, alpha, class 2A, member 1 |
|  | MAN2A1 | mannosidase, alpha, class 2A, member 1 |
|  | RPN2 | ribophorin II |
|  | ALG2 | asparagine-linked glycosylation 2, alpha-1,3-mannosyltransferase homolog (S. cerevisiae) |
|  | B4GALT3 | UDP-Gal:betaGlcNAc beta 1,4- galactosyltransferase, polypeptide 3 |
|  | B4GALT2 | UDP-Gal:betaGlcNAc beta 1,4- galactosyltransferase, polypeptide 2 |
|  | ALG10B | asparagine-linked glycosylation 10, alpha-1,2-glucosyltransferase homolog B (yeast) |
|  | ALG10B | asparagine-linked glycosylation 10, alpha-1,2-glucosyltransferase homolog B (yeast) |
| **KEGG pathway----Pancreatic cancer----05212** | **Gene Symbol** | **Gene Name** |
|  | MAPK8 | mitogen-activated protein kinase 8 |
|  | TGFB1 | transforming growth factor, beta 1 |
|  | VEGFC | vascular endothelial growth factor C |
|  | BRAF | v-raf murine sarcoma viral oncogene homolog B1 |
|  | ACVR1B | activin A receptor, type IB |
|  | RALBP1 | ralA binding protein 1 |
|  | RALBP1 | ralA binding protein 1 |
|  | CDK4 | cyclin-dependent kinase 4 |
|  | MAP2K1 | mitogen-activated protein kinase kinase 1 |
|  | E2F3 | E2F transcription factor 3 |
|  | RALGDS | ral guanine nucleotide dissociation stimulator |
|  | RALGDS | ral guanine nucleotide dissociation stimulator |
|  | TGFBR1 | transforming growth factor, beta receptor 1 |
|  | TGFBR1 | transforming growth factor, beta receptor 1 |
|  | BCL2L1 | BCL2-like 1 |
|  | VEGFA | vascular endothelial growth factor A |
|  | VEGFA | vascular endothelial growth factor A |
|  | VEGFA | vascular endothelial growth factor A |
|  | PIK3CB | phosphoinositide-3-kinase, catalytic, beta polypeptide |
|  | EGFR | epidermal growth factor receptor (erythroblastic leukemia viral (v-erb-b) oncogene homolog, avian) |
|  | EGFR | epidermal growth factor receptor (erythroblastic leukemia viral (v-erb-b) oncogene homolog, avian) |
|  | EGFR | epidermal growth factor receptor (erythroblastic leukemia viral (v-erb-b) oncogene homolog, avian) |
|  | SMAD3 | SMAD family member 3 |
|  | SMAD3 | SMAD family member 3 |
|  | SMAD3 | SMAD family member 3 |
|  | CCND1 | cyclin D1 |
|  | CCND1 | cyclin D1 |
|  | RAC2 | ras-related C3 botulinum toxin substrate 2 (rho family, small GTP binding protein Rac2) |
|  | BRCA2 | breast cancer 2, early onset |
|  | SMAD2 | SMAD family member 2 |
|  | SMAD2 | SMAD family member 2 |
|  | SMAD2 | SMAD family member 2 |
|  | JAK1 | Janus kinase 1 |
|  | RAD51 | RAD51 homolog (RecA homolog, E. coli) (S. cerevisiae) |
|  | RALB | v-ral simian leukemia viral oncogene homolog B (ras related; GTP binding protein) |
|  | RALB | v-ral simian leukemia viral oncogene homolog B (ras related; GTP binding protein) |
|  | ARHGEF6 | Rac/Cdc42 guanine nucleotide exchange factor (GEF) 6 |
|  | AKT3 | v-akt murine thymoma viral oncogene homolog 3 (protein kinase B, gamma) |
|  | AKT3 | v-akt murine thymoma viral oncogene homolog 3 (protein kinase B, gamma) |
|  | AKT3 | v-akt murine thymoma viral oncogene homolog 3 (protein kinase B, gamma) |
|  | AKT3 | v-akt murine thymoma viral oncogene homolog 3 (protein kinase B, gamma) |
|  | IKBKB | inhibitor of kappa light polypeptide gene enhancer in B-cells, kinase beta |
|  | IKBKB | inhibitor of kappa light polypeptide gene enhancer in B-cells, kinase beta |
|  | MAPK9 | mitogen-activated protein kinase 9 |
|  | PIK3CA | phosphoinositide-3-kinase, catalytic, alpha polypeptide |
|  | MAPK10 | mitogen-activated protein kinase 10 |
|  | MAPK1 | mitogen-activated protein kinase 1 |
|  | MAPK1 | mitogen-activated protein kinase 1 |
|  | CDK6 | cyclin-dependent kinase 6 |
|  | CDK6 | cyclin-dependent kinase 6 |
|  | SMAD4 | SMAD family member 4 |
|  | SMAD4 | SMAD family member 4 |
|  | CDKN2A | cyclin-dependent kinase inhibitor 2A (melanoma, p16, inhibits CDK4) |
| **KEGG pathway----Nucleotide excision repair----03420** | **Gene Symbol** | **Gene Name** |
|  | RFC4 | replication factor C (activator 1) 4, 37kDa |
|  | DDB2 | damage-specific DNA binding protein 2, 48kDa |
|  | RFC3 | replication factor C (activator 1) 3, 38kDa |
|  | CUL4A | cullin 4A |
|  | RBX1 | ring-box 1 |
|  | CUL4B | cullin 4B |
|  | CUL4B | cullin 4B |
|  | CUL4B | cullin 4B |
|  | CUL4B | cullin 4B |
|  | ERCC4 | excision repair cross-complementing rodent repair deficiency, complementation group 4 |
|  | CDK7 | cyclin-dependent kinase 7 |
|  | POLD3 | polymerase (DNA-directed), delta 3, accessory subunit |
|  | POLD1 | polymerase (DNA directed), delta 1, catalytic subunit 125kDa |
|  | RFC2 | replication factor C (activator 1) 2, 40kDa |
|  | RFC2 | replication factor C (activator 1) 2, 40kDa |
|  | RPA3 | replication protein A3, 14kDa |
|  | POLE4 | polymerase (DNA-directed), epsilon 4 (p12 subunit) |
|  | POLE4 | polymerase (DNA-directed), epsilon 4 (p12 subunit) |
|  | RAD23A | RAD23 homolog A (S. cerevisiae) |
|  | RPA1 | replication protein A1, 70kDa |
|  | RPA2 | replication protein A2, 32kDa |
|  | CETN2 | centrin, EF-hand protein, 2 |
|  | PCNA | proliferating cell nuclear antigen |
|  | RFC5 | replication factor C (activator 1) 5, 36.5kDa |
|  | RFC5 | replication factor C (activator 1) 5, 36.5kDa |
|  | POLE2 | polymerase (DNA directed), epsilon 2 (p59 subunit) |
|  | RAD23B | RAD23 homolog B (S. cerevisiae) |
|  | RAD23B | RAD23 homolog B (S. cerevisiae) |
|  | POLE3 | polymerase (DNA directed), epsilon 3 (p17 subunit) |
|  | RFC1 | replication factor C (activator 1) 1, 145kDa |
|  | RFC1 | replication factor C (activator 1) 1, 145kDa |
|  | GTF2H2 | general transcription factor IIH, polypeptide 2, 44kDa |
|  | POLD2 | polymerase (DNA directed), delta 2, regulatory subunit 50kDa |
| **KEGG pathway----Adherens junction----04520** | **Gene Symbol** | **Gene Name** |
|  | LMO7 | LIM domain 7 |
|  | LMO7 | LIM domain 7 |
|  | SORBS1 | sorbin and SH3 domain containing 1 |
|  | SORBS1 | sorbin and SH3 domain containing 1 |
|  | WASF1 | WAS protein family, member 1 |
|  | CTNNA1 | catenin (cadherin-associated protein), alpha 1, 102kDa |
|  | CSNK2A1 | casein kinase 2, alpha 1 polypeptide |
|  | CSNK2A1 | casein kinase 2, alpha 1 polypeptide |
|  | NLK | nemo-like kinase |
|  | TGFBR1 | transforming growth factor, beta receptor 1 |
|  | TGFBR1 | transforming growth factor, beta receptor 1 |
|  | SRC | v-src sarcoma (Schmidt-Ruppin A-2) viral oncogene homolog (avian) |
|  | SSX2IP | synovial sarcoma, X breakpoint 2 interacting protein |
|  | SSX2IP | synovial sarcoma, X breakpoint 2 interacting protein |
|  | RAC2 | ras-related C3 botulinum toxin substrate 2 (rho family, small GTP binding protein Rac2) |
|  | CDH1 | cadherin 1, type 1, E-cadherin (epithelial) |
|  | PVRL1 | poliovirus receptor-related 1 (herpesvirus entry mediator C) |
|  | MAP3K7 | mitogen-activated protein kinase kinase kinase 7 |
|  | MAP3K7 | mitogen-activated protein kinase kinase kinase 7 |
|  | SNAI2 | snail homolog 2 (Drosophila) |
|  | CSNK2A2 | casein kinase 2, alpha prime polypeptide |
|  | FARP2 | FERM, RhoGEF and pleckstrin domain protein 2 |
|  | RHOA | ras homolog gene family, member A |
|  | RHOA | ras homolog gene family, member A |
|  | ACVR1B | activin A receptor, type IB |
|  | PTPN6 | protein tyrosine phosphatase, non-receptor type 6 |
|  | PTPRM | protein tyrosine phosphatase, receptor type, M |
|  | ACTN1 | actinin, alpha 1 |
|  | ACTN1 | actinin, alpha 1 |
|  | ACTN1 | actinin, alpha 1 |
|  | TCF7L2 | transcription factor 7-like 2 (T-cell specific, HMG-box) |
|  | TCF7L2 | transcription factor 7-like 2 (T-cell specific, HMG-box) |
|  | TCF7L2 | transcription factor 7-like 2 (T-cell specific, HMG-box) |
|  | TCF7L2 | transcription factor 7-like 2 (T-cell specific, HMG-box) |
|  | TCF7L2 | transcription factor 7-like 2 (T-cell specific, HMG-box) |
|  | TCF7L2 | transcription factor 7-like 2 (T-cell specific, HMG-box) |
|  | BAIAP2 | BAI1-associated protein 2 |
|  | FGFR1 | fibroblast growth factor receptor 1 |
|  | EGFR | epidermal growth factor receptor (erythroblastic leukemia viral (v-erb-b) oncogene homolog, avian) |
|  | EGFR | epidermal growth factor receptor (erythroblastic leukemia viral (v-erb-b) oncogene homolog, avian) |
|  | EGFR | epidermal growth factor receptor (erythroblastic leukemia viral (v-erb-b) oncogene homolog, avian) |
|  | PTPRF | protein tyrosine phosphatase, receptor type, F |
|  | PTPRF | protein tyrosine phosphatase, receptor type, F |
|  | SMAD3 | SMAD family member 3 |
|  | SMAD3 | SMAD family member 3 |
|  | SMAD3 | SMAD family member 3 |
|  | INSR | insulin receptor |
|  | INSR | insulin receptor |
|  | SMAD2 | SMAD family member 2 |
|  | SMAD2 | SMAD family member 2 |
|  | SMAD2 | SMAD family member 2 |
|  | FYN | FYN oncogene related to SRC, FGR, YES |
|  | ACP1 | acid phosphatase 1, soluble |
|  | ACP1 | acid phosphatase 1, soluble |
|  | ACP1 | acid phosphatase 1, soluble |
|  | CTNND1 | catenin (cadherin-associated protein), delta 1 |
|  | MAPK1 | mitogen-activated protein kinase 1 |
|  | MAPK1 | mitogen-activated protein kinase 1 |
|  | SMAD4 | SMAD family member 4 |
|  | SMAD4 | SMAD family member 4 |
| **KEGG pathway----Bladder cancer----05219** | **Gene Symbol** | **Gene Name** |
|  | MMP9 | matrix metallopeptidase 9 (gelatinase B, 92kDa gelatinase, 92kDa type IV collagenase) |
|  | VEGFC | vascular endothelial growth factor C |
|  | BRAF | v-raf murine sarcoma viral oncogene homolog B1 |
|  | NRAS | neuroblastoma RAS viral (v-ras) oncogene homolog |
|  | CDK4 | cyclin-dependent kinase 4 |
|  | MAP2K1 | mitogen-activated protein kinase kinase 1 |
|  | E2F3 | E2F transcription factor 3 |
|  | MMP1 | matrix metallopeptidase 1 (interstitial collagenase) |
|  | VEGFA | vascular endothelial growth factor A |
|  | VEGFA | vascular endothelial growth factor A |
|  | VEGFA | vascular endothelial growth factor A |
|  | RPS6KA5 | ribosomal protein S6 kinase, 90kDa, polypeptide 5 |
|  | RPS6KA5 | ribosomal protein S6 kinase, 90kDa, polypeptide 5 |
|  | MAP2K2 | mitogen-activated protein kinase kinase 2 |
|  | EGFR | epidermal growth factor receptor (erythroblastic leukemia viral (v-erb-b) oncogene homolog, avian) |
|  | EGFR | epidermal growth factor receptor (erythroblastic leukemia viral (v-erb-b) oncogene homolog, avian) |
|  | EGFR | epidermal growth factor receptor (erythroblastic leukemia viral (v-erb-b) oncogene homolog, avian) |
|  | THBS1 | thrombospondin 1 |
|  | THBS1 | thrombospondin 1 |
|  | THBS1 | thrombospondin 1 |
|  | CCND1 | cyclin D1 |
|  | CCND1 | cyclin D1 |
|  | FGFR3 | fibroblast growth factor receptor 3 |
|  | FGFR3 | fibroblast growth factor receptor 3 |
|  | TYMP | thymidine phosphorylase |
|  | MDM2 | Mdm2 p53 binding protein homolog (mouse) |
|  | CDH1 | cadherin 1, type 1, E-cadherin (epithelial) |
|  | MYC | v-myc myelocytomatosis viral oncogene homolog (avian) |
|  | IL8 | interleukin 8 |
|  | MMP2 | matrix metallopeptidase 2 (gelatinase A, 72kDa gelatinase, 72kDa type IV collagenase) |
|  | DAPK1 | death-associated protein kinase 1 |
|  | MAPK1 | mitogen-activated protein kinase 1 |
|  | MAPK1 | mitogen-activated protein kinase 1 |
|  | CDKN2A | cyclin-dependent kinase inhibitor 2A (melanoma, p16, inhibits CDK4) |
| **KEGG pathway----Axon guidance----04360** | **Gene Symbol** | **Gene Name** |
|  | EFNB2 | ephrin-B2 |
|  | PLXNB3 | plexin B3 |
|  | LRRC4C | leucine rich repeat containing 4C |
|  | PLXNC1 | plexin C1 |
|  | NTN3 | netrin 3 |
|  | NTN3 | netrin 3 |
|  | ROBO1 | roundabout, axon guidance receptor, homolog 1 (Drosophila) |
|  | PPP3CB | protein phosphatase 3 (formerly 2B), catalytic subunit, beta isoform |
|  | SEMA6A | sema domain, transmembrane domain (TM), and cytoplasmic domain, (semaphorin) 6A |
|  | NFAT5 | nuclear factor of activated T-cells 5, tonicity-responsive |
|  | NFAT5 | nuclear factor of activated T-cells 5, tonicity-responsive |
|  | PLXNB1 | plexin B1 |
|  | CDK5 | cyclin-dependent kinase 5 |
|  | ABLIM1 | actin binding LIM protein 1 |
|  | ABLIM1 | actin binding LIM protein 1 |
|  | RAC2 | ras-related C3 botulinum toxin substrate 2 (rho family, small GTP binding protein Rac2) |
|  | NFATC3 | nuclear factor of activated T-cells, cytoplasmic, calcineurin-dependent 3 |
|  | EPHB2 | EPH receptor B2 |
|  | DPYSL2 | dihydropyrimidinase-like 2 |
|  | PAK2 | p21 protein (Cdc42/Rac)-activated kinase 2 |
|  | PAK2 | p21 protein (Cdc42/Rac)-activated kinase 2 |
|  | PAK2 | p21 protein (Cdc42/Rac)-activated kinase 2 |
|  | NTN4 | netrin 4 |
|  | EPHB6 | EPH receptor B6 |
|  | ROCK2 | Rho-associated, coiled-coil containing protein kinase 2 |
|  | CFL1 | cofilin 1 (non-muscle) |
|  | LIMK2 | LIM domain kinase 2 |
|  | LIMK2 | LIM domain kinase 2 |
|  | PAK1 | p21 protein (Cdc42/Rac)-activated kinase 1 |
|  | PAK1 | p21 protein (Cdc42/Rac)-activated kinase 1 |
|  | PPP3CA | protein phosphatase 3 (formerly 2B), catalytic subunit, alpha isoform |
|  | PPP3CA | protein phosphatase 3 (formerly 2B), catalytic subunit, alpha isoform |
|  | NRAS | neuroblastoma RAS viral (v-ras) oncogene homolog |
|  | RHOA | ras homolog gene family, member A |
|  | RHOA | ras homolog gene family, member A |
|  | NCK1 | NCK adaptor protein 1 |
|  | NCK1 | NCK adaptor protein 1 |
|  | SEMA3F | sema domain, immunoglobulin domain (Ig), short basic domain, secreted, (semaphorin) 3F |
|  | PTK2 | PTK2 protein tyrosine kinase 2 |
|  | PTK2 | PTK2 protein tyrosine kinase 2 |
|  | GNAI3 | guanine nucleotide binding protein (G protein), alpha inhibiting activity polypeptide 3 |
|  | GNAI3 | guanine nucleotide binding protein (G protein), alpha inhibiting activity polypeptide 3 |
|  | GNAI3 | guanine nucleotide binding protein (G protein), alpha inhibiting activity polypeptide 3 |
|  | PLXNB2 | plexin B2 |
|  | RASA1 | RAS p21 protein activator (GTPase activating protein) 1 |
|  | SEMA5A | sema domain, seven thrombospondin repeats (type 1 and type 1-like), transmembrane domain (TM) and short cytoplasmic domain, (semaphorin) 5A |
|  | SEMA5A | sema domain, seven thrombospondin repeats (type 1 and type 1-like), transmembrane domain (TM) and short cytoplasmic domain, (semaphorin) 5A |
|  | SEMA5A | sema domain, seven thrombospondin repeats (type 1 and type 1-like), transmembrane domain (TM) and short cytoplasmic domain, (semaphorin) 5A |
|  | CFL2 | cofilin 2 (muscle) |
|  | CFL2 | cofilin 2 (muscle) |
|  | FYN | FYN oncogene related to SRC, FGR, YES |
|  | CXCR4 | chemokine (C-X-C motif) receptor 4 |
|  | CXCR4 | chemokine (C-X-C motif) receptor 4 |
|  | EPHA2 | EPH receptor A2 |
|  | SEMA4A | sema domain, immunoglobulin domain (Ig), transmembrane domain (TM) and short cytoplasmic domain, (semaphorin) 4A |
|  | PPP3R1 | protein phosphatase 3 (formerly 2B), regulatory subunit B, alpha isoform |
|  | SEMA4G | sema domain, immunoglobulin domain (Ig), transmembrane domain (TM) and short cytoplasmic domain, (semaphorin) 4G |
|  | ITGB1 | integrin, beta 1 (fibronectin receptor, beta polypeptide, antigen CD29 includes MDF2, MSK12) |
|  | ITGB1 | integrin, beta 1 (fibronectin receptor, beta polypeptide, antigen CD29 includes MDF2, MSK12) |
|  | SEMA3C | sema domain, immunoglobulin domain (Ig), short basic domain, secreted, (semaphorin) 3C |
|  | ROCK1 | Rho-associated, coiled-coil containing protein kinase 1 |
|  | ROCK1 | Rho-associated, coiled-coil containing protein kinase 1 |
|  | UNC5B | unc-5 homolog B (C. elegans) |
|  | MAPK1 | mitogen-activated protein kinase 1 |
|  | MAPK1 | mitogen-activated protein kinase 1 |
|  | SEMA4B | sema domain, immunoglobulin domain (Ig), transmembrane domain (TM) and short cytoplasmic domain, (semaphorin) 4B |
| **KEGG pathway----ErbB signaling pathway----04012** | **Gene Symbol** | **Gene Name** |
|  | MAPK8 | mitogen-activated protein kinase 8 |
|  | BRAF | v-raf murine sarcoma viral oncogene homolog B1 |
|  | MAP2K1 | mitogen-activated protein kinase kinase 1 |
|  | PRKCA | protein kinase C, alpha |
|  | SRC | v-src sarcoma (Schmidt-Ruppin A-2) viral oncogene homolog (avian) |
|  | ERBB4 | v-erb-a erythroblastic leukemia viral oncogene homolog 4 (avian) |
|  | SHC2 | SHC (Src homology 2 domain containing) transforming protein 2 |
|  | CRKL | v-crk sarcoma virus CT10 oncogene homolog (avian)-like |
|  | ABL2 | v-abl Abelson murine leukemia viral oncogene homolog 2 (arg, Abelson-related gene) |
|  | JUN | jun oncogene |
|  | JUN | jun oncogene |
|  | JUN | jun oncogene |
|  | SHC1 | SHC (Src homology 2 domain containing) transforming protein 1 |
|  | MYC | v-myc myelocytomatosis viral oncogene homolog (avian) |
|  | PAK2 | p21 protein (Cdc42/Rac)-activated kinase 2 |
|  | PAK2 | p21 protein (Cdc42/Rac)-activated kinase 2 |
|  | PAK2 | p21 protein (Cdc42/Rac)-activated kinase 2 |
|  | MAPK9 | mitogen-activated protein kinase 9 |
|  | PIK3CA | phosphoinositide-3-kinase, catalytic, alpha polypeptide |
|  | CDKN1B | cyclin-dependent kinase inhibitor 1B (p27, Kip1) |
|  | ERBB3 | v-erb-b2 erythroblastic leukemia viral oncogene homolog 3 (avian) |
|  | ERBB3 | v-erb-b2 erythroblastic leukemia viral oncogene homolog 3 (avian) |
|  | CAMK2G | calcium/calmodulin-dependent protein kinase II gamma |
|  | PAK1 | p21 protein (Cdc42/Rac)-activated kinase 1 |
|  | PAK1 | p21 protein (Cdc42/Rac)-activated kinase 1 |
|  | NRAS | neuroblastoma RAS viral (v-ras) oncogene homolog |
|  | SOS1 | son of sevenless homolog 1 (Drosophila) |
|  | NCK1 | NCK adaptor protein 1 |
|  | NCK1 | NCK adaptor protein 1 |
|  | CBLB | Cas-Br-M (murine) ecotropic retroviral transforming sequence b |
|  | PTK2 | PTK2 protein tyrosine kinase 2 |
|  | PTK2 | PTK2 protein tyrosine kinase 2 |
|  | MAP2K2 | mitogen-activated protein kinase kinase 2 |
|  | PIK3CB | phosphoinositide-3-kinase, catalytic, beta polypeptide |
|  | EGFR | epidermal growth factor receptor (erythroblastic leukemia viral (v-erb-b) oncogene homolog, avian) |
|  | EGFR | epidermal growth factor receptor (erythroblastic leukemia viral (v-erb-b) oncogene homolog, avian) |
|  | EGFR | epidermal growth factor receptor (erythroblastic leukemia viral (v-erb-b) oncogene homolog, avian) |
|  | GRB2 | growth factor receptor-bound protein 2 |
|  | CAMK2D | calcium/calmodulin-dependent protein kinase II delta |
|  | AREG | amphiregulin |
|  | EIF4EBP1 | eukaryotic translation initiation factor 4E binding protein 1 |
|  | AKT3 | v-akt murine thymoma viral oncogene homolog 3 (protein kinase B, gamma) |
|  | AKT3 | v-akt murine thymoma viral oncogene homolog 3 (protein kinase B, gamma) |
|  | AKT3 | v-akt murine thymoma viral oncogene homolog 3 (protein kinase B, gamma) |
|  | AKT3 | v-akt murine thymoma viral oncogene homolog 3 (protein kinase B, gamma) |
|  | RPS6KB1 | ribosomal protein S6 kinase, 70kDa, polypeptide 1 |
|  | MAPK1 | mitogen-activated protein kinase 1 |
|  | MAPK1 | mitogen-activated protein kinase 1 |
|  | MAPK10 | mitogen-activated protein kinase 10 |
|  | MAP2K4 | mitogen-activated protein kinase kinase 4 |
| **KEGG pathway----Neurotrophin signaling pathway----04722** | **Gene Symbol** | **Gene Name** |
|  | MAPK8 | mitogen-activated protein kinase 8 |
|  | BRAF | v-raf murine sarcoma viral oncogene homolog B1 |
|  | MAGED1 | melanoma antigen family D, 1 |
|  | MAP2K1 | mitogen-activated protein kinase kinase 1 |
|  | YWHAE | tyrosine 3-monooxygenase/tryptophan 5-monooxygenase activation protein, epsilon polypeptide |
|  | RPS6KA5 | ribosomal protein S6 kinase, 90kDa, polypeptide 5 |
|  | RPS6KA5 | ribosomal protein S6 kinase, 90kDa, polypeptide 5 |
|  | SHC2 | SHC (Src homology 2 domain containing) transforming protein 2 |
|  | CRKL | v-crk sarcoma virus CT10 oncogene homolog (avian)-like |
|  | SH2B3 | SH2B adaptor protein 3 |
|  | JUN | jun oncogene |
|  | JUN | jun oncogene |
|  | JUN | jun oncogene |
|  | CALM2 | calmodulin 2 (phosphorylase kinase, delta) |
|  | RAP1A | RAP1A, member of RAS oncogene family |
|  | SHC1 | SHC (Src homology 2 domain containing) transforming protein 1 |
|  | IRAK1 | interleukin-1 receptor-associated kinase 1 |
|  | IKBKB | inhibitor of kappa light polypeptide gene enhancer in B-cells, kinase beta |
|  | IKBKB | inhibitor of kappa light polypeptide gene enhancer in B-cells, kinase beta |
|  | MAPK9 | mitogen-activated protein kinase 9 |
|  | BAX | BCL2-associated X protein |
|  | BAX | BCL2-associated X protein |
|  | PIK3CA | phosphoinositide-3-kinase, catalytic, alpha polypeptide |
|  | FRS2 | fibroblast growth factor receptor substrate 2 |
|  | FRS2 | fibroblast growth factor receptor substrate 2 |
|  | CAMK2G | calcium/calmodulin-dependent protein kinase II gamma |
|  | NRAS | neuroblastoma RAS viral (v-ras) oncogene homolog |
|  | RHOA | ras homolog gene family, member A |
|  | RHOA | ras homolog gene family, member A |
|  | PDK1 | pyruvate dehydrogenase kinase, isozyme 1 |
|  | SOS1 | son of sevenless homolog 1 (Drosophila) |
|  | NTF4 | neurotrophin 4 |
|  | CALM1 | calmodulin 1 (phosphorylase kinase, delta) |
|  | YWHAQ | tyrosine 3-monooxygenase/tryptophan 5-monooxygenase activation protein, theta polypeptide |
|  | YWHAQ | tyrosine 3-monooxygenase/tryptophan 5-monooxygenase activation protein, theta polypeptide |
|  | ARHGDIB | Rho GDP dissociation inhibitor (GDI) beta |
|  | PIK3CB | phosphoinositide-3-kinase, catalytic, beta polypeptide |
|  | MAP2K2 | mitogen-activated protein kinase kinase 2 |
|  | GRB2 | growth factor receptor-bound protein 2 |
|  | IRS1 | insulin receptor substrate 1 |
|  | IRS2 | insulin receptor substrate 2 |
|  | IRS2 | insulin receptor substrate 2 |
|  | MAPKAPK2 | mitogen-activated protein kinase-activated protein kinase 2 |
|  | CAMK2D | calcium/calmodulin-dependent protein kinase II delta |
|  | NGFRAP1 | nerve growth factor receptor (TNFRSF16) associated protein 1 |
|  | AKT3 | v-akt murine thymoma viral oncogene homolog 3 (protein kinase B, gamma) |
|  | AKT3 | v-akt murine thymoma viral oncogene homolog 3 (protein kinase B, gamma) |
|  | AKT3 | v-akt murine thymoma viral oncogene homolog 3 (protein kinase B, gamma) |
|  | AKT3 | v-akt murine thymoma viral oncogene homolog 3 (protein kinase B, gamma) |
|  | NFKBIA | nuclear factor of kappa light polypeptide gene enhancer in B-cells inhibitor, alpha |
|  | YWHAZ | tyrosine 3-monooxygenase/tryptophan 5-monooxygenase activation protein, zeta polypeptide |
|  | YWHAZ | tyrosine 3-monooxygenase/tryptophan 5-monooxygenase activation protein, zeta polypeptide |
|  | MAP3K5 | mitogen-activated protein kinase kinase kinase 5 |
|  | MAP3K5 | mitogen-activated protein kinase kinase kinase 5 |
|  | MAPK10 | mitogen-activated protein kinase 10 |
|  | YWHAH | tyrosine 3-monooxygenase/tryptophan 5-monooxygenase activation protein, eta polypeptide |
|  | MAPK1 | mitogen-activated protein kinase 1 |
|  | MAPK1 | mitogen-activated protein kinase 1 |
|  | YWHAB | tyrosine 3-monooxygenase/tryptophan 5-monooxygenase activation protein, beta polypeptide |
|  | RPS6KA3 | ribosomal protein S6 kinase, 90kDa, polypeptide 3 |
|  | RPS6KA3 | ribosomal protein S6 kinase, 90kDa, polypeptide 3 |
| **KEGG pathway----Valine, leucine and isoleucine degradation----00280** | **Gene Symbol** | **Gene Name** |
|  | MCEE | methylmalonyl CoA epimerase |
|  | PCCB | propionyl Coenzyme A carboxylase, beta polypeptide |
|  | ALDH2 | aldehyde dehydrogenase 2 family (mitochondrial) |
|  | EHHADH | enoyl-Coenzyme A, hydratase/3-hydroxyacyl Coenzyme A dehydrogenase |
|  | MUT | methylmalonyl Coenzyme A mutase |
|  | ALDH6A1 | aldehyde dehydrogenase 6 family, member A1 |
|  | BCKDHA | branched chain keto acid dehydrogenase E1, alpha polypeptide |
|  | HADHA | hydroxyacyl-Coenzyme A dehydrogenase/3-ketoacyl-Coenzyme A thiolase/enoyl-Coenzyme A hydratase (trifunctional protein), alpha subunit |
|  | HSD17B10 | hydroxysteroid (17-beta) dehydrogenase 10 |
|  | BCAT1 | branched chain aminotransferase 1, cytosolic |
|  | BCAT1 | branched chain aminotransferase 1, cytosolic |
|  | ABAT | 4-aminobutyrate aminotransferase |
|  | BCAT2 | branched chain aminotransferase 2, mitochondrial |
|  | ACAT2 | acetyl-Coenzyme A acetyltransferase 2 |
|  | ACAT2 | acetyl-Coenzyme A acetyltransferase 2 |
|  | ACAT2 | acetyl-Coenzyme A acetyltransferase 2 |
|  | HADH | hydroxyacyl-Coenzyme A dehydrogenase |
|  | OXCT1 | 3-oxoacid CoA transferase 1 |
|  | ACAA2 | acetyl-Coenzyme A acyltransferase 2 |
|  | ACAT1 | acetyl-Coenzyme A acetyltransferase 1 |
|  | ALDH7A1 | aldehyde dehydrogenase 7 family, member A1 |
|  | ALDH7A1 | aldehyde dehydrogenase 7 family, member A1 |
|  | HIBCH | 3-hydroxyisobutyryl-Coenzyme A hydrolase |
|  | ACADM | acyl-Coenzyme A dehydrogenase, C-4 to C-12 straight chain |
|  | DLD | dihydrolipoamide dehydrogenase |
|  | DBT | dihydrolipoamide branched chain transacylase E2 |
|  | ALDH3A2 | aldehyde dehydrogenase 3 family, member A2 |
|  | HIBADH | 3-hydroxyisobutyrate dehydrogenase |
| **KEGG pathway----Pathogenic Escherichia coli infection----05130** | **Gene Symbol** | **Gene Name** |
|  | CLDN1 | claudin 1 |
|  | CLDN1 | claudin 1 |
|  | TUBB2A | tubulin, beta 2A |
|  | RHOA | ras homolog gene family, member A |
|  | RHOA | ras homolog gene family, member A |
|  | TUBA1A | tubulin, alpha 1a |
|  | NCK1 | NCK adaptor protein 1 |
|  | NCK1 | NCK adaptor protein 1 |
|  | YWHAQ | tyrosine 3-monooxygenase/tryptophan 5-monooxygenase activation protein, theta polypeptide |
|  | YWHAQ | tyrosine 3-monooxygenase/tryptophan 5-monooxygenase activation protein, theta polypeptide |
|  | EZR | ezrin |
|  | TUBA1C | tubulin, alpha 1c |
|  | ARPC4 | actin related protein 2/3 complex, subunit 4, 20kDa |
|  | CD14 | CD14 molecule |
|  | TUBB6 | tubulin, beta 6 |
|  | PRKCA | protein kinase C, alpha |
|  | ARPC1A | actin related protein 2/3 complex, subunit 1A, 41kDa |
|  | TUBA1B | tubulin, alpha 1b |
|  | FYN | FYN oncogene related to SRC, FGR, YES |
|  | CDH1 | cadherin 1, type 1, E-cadherin (epithelial) |
|  | HCLS1 | hematopoietic cell-specific Lyn substrate 1 |
|  | ARPC5 | actin related protein 2/3 complex, subunit 5, 16kDa |
|  | ARPC5 | actin related protein 2/3 complex, subunit 5, 16kDa |
|  | ITGB1 | integrin, beta 1 (fibronectin receptor, beta polypeptide, antigen CD29 includes MDF2, MSK12) |
|  | ITGB1 | integrin, beta 1 (fibronectin receptor, beta polypeptide, antigen CD29 includes MDF2, MSK12) |
|  | YWHAZ | tyrosine 3-monooxygenase/tryptophan 5-monooxygenase activation protein, zeta polypeptide |
|  | YWHAZ | tyrosine 3-monooxygenase/tryptophan 5-monooxygenase activation protein, zeta polypeptide |
|  | ROCK1 | Rho-associated, coiled-coil containing protein kinase 1 |
|  | ROCK1 | Rho-associated, coiled-coil containing protein kinase 1 |
|  | LY96 | lymphocyte antigen 96 |
|  | CTTN | cortactin |
|  | TUBA3D | tubulin, alpha 3d |
|  | KRT18 | keratin 18 |
|  | ROCK2 | Rho-associated, coiled-coil containing protein kinase 2 |
|  | TUBB | tubulin, beta |
|  | TUBB | tubulin, beta |
|  | TUBB | tubulin, beta |
|  | TUBB3 | tubulin, beta 3 |
| **KEGG pathway----Tight junction----04530** | **Gene Symbol** | **Gene Name** |
|  | MYL9 | myosin, light chain 9, regulatory |
|  | CLDN1 | claudin 1 |
|  | CLDN1 | claudin 1 |
|  | CLDN5 | claudin 5 |
|  | AMOTL1 | angiomotin like 1 |
|  | PARD6G | par-6 partitioning defective 6 homolog gamma (C. elegans) |
|  | CTNNA1 | catenin (cadherin-associated protein), alpha 1, 102kDa |
|  | PPP2CB | protein phosphatase 2 (formerly 2A), catalytic subunit, beta isoform |
|  | PPP2R2A | protein phosphatase 2 (formerly 2A), regulatory subunit B, alpha isoform |
|  | CSNK2A1 | casein kinase 2, alpha 1 polypeptide |
|  | CSNK2A1 | casein kinase 2, alpha 1 polypeptide |
|  | MYH9 | myosin, heavy chain 9, non-muscle |
|  | EPB41L2 | erythrocyte membrane protein band 4.1-like 2 |
|  | PRKCA | protein kinase C, alpha |
|  | MYH10 | myosin, heavy chain 10, non-muscle |
|  | SRC | v-src sarcoma (Schmidt-Ruppin A-2) viral oncogene homolog (avian) |
|  | PPP2R1B | protein phosphatase 2 (formerly 2A), regulatory subunit A, beta isoform |
|  | PPP2R1B | protein phosphatase 2 (formerly 2A), regulatory subunit A, beta isoform |
|  | PPP2R1B | protein phosphatase 2 (formerly 2A), regulatory subunit A, beta isoform |
|  | TJP2 | tight junction protein 2 (zona occludens 2) |
|  | TJP2 | tight junction protein 2 (zona occludens 2) |
|  | LLGL2 | lethal giant larvae homolog 2 (Drosophila) |
|  | CLDN4 | claudin 4 |
|  | MYH14 | myosin, heavy chain 14 |
|  | MYH14 | myosin, heavy chain 14 |
|  | CTTN | cortactin |
|  | RRAS2 | related RAS viral (r-ras) oncogene homolog 2 |
|  | CLDN18 | claudin 18 |
|  | CLDN18 | claudin 18 |
|  | CLDN7 | claudin 7 |
|  | CSNK2A2 | casein kinase 2, alpha prime polypeptide |
|  | SPTAN1 | spectrin, alpha, non-erythrocytic 1 (alpha-fodrin) |
|  | SPTAN1 | spectrin, alpha, non-erythrocytic 1 (alpha-fodrin) |
|  | SPTAN1 | spectrin, alpha, non-erythrocytic 1 (alpha-fodrin) |
|  | NRAS | neuroblastoma RAS viral (v-ras) oncogene homolog |
|  | RHOA | ras homolog gene family, member A |
|  | RHOA | ras homolog gene family, member A |
|  | RRAS | related RAS viral (r-ras) oncogene homolog |
|  | CLDN23 | claudin 23 |
|  | CLDN23 | claudin 23 |
|  | EPB41L1 | erythrocyte membrane protein band 4.1-like 1 |
|  | CDK4 | cyclin-dependent kinase 4 |
|  | ACTN1 | actinin, alpha 1 |
|  | ACTN1 | actinin, alpha 1 |
|  | ACTN1 | actinin, alpha 1 |
|  | GNAI3 | guanine nucleotide binding protein (G protein), alpha inhibiting activity polypeptide 3 |
|  | GNAI3 | guanine nucleotide binding protein (G protein), alpha inhibiting activity polypeptide 3 |
|  | GNAI3 | guanine nucleotide binding protein (G protein), alpha inhibiting activity polypeptide 3 |
|  | SYMPK | symplekin |
|  | INADL | InaD-like (Drosophila) |
|  | INADL | InaD-like (Drosophila) |
|  | INADL | InaD-like (Drosophila) |
|  | INADL | InaD-like (Drosophila) |
|  | MYH11 | myosin, heavy chain 11, smooth muscle |
|  | MYH11 | myosin, heavy chain 11, smooth muscle |
|  | TJP3 | tight junction protein 3 (zona occludens 3) |
|  | HCLS1 | hematopoietic cell-specific Lyn substrate 1 |
|  | CLDN2 | claudin 2 |
|  | AKT3 | v-akt murine thymoma viral oncogene homolog 3 (protein kinase B, gamma) |
|  | AKT3 | v-akt murine thymoma viral oncogene homolog 3 (protein kinase B, gamma) |
|  | AKT3 | v-akt murine thymoma viral oncogene homolog 3 (protein kinase B, gamma) |
|  | AKT3 | v-akt murine thymoma viral oncogene homolog 3 (protein kinase B, gamma) |
|  | PRKCZ | protein kinase C, zeta |
|  | VAPA | VAMP (vesicle-associated membrane protein)-associated protein A, 33kDa |
|  | MAGI1 | membrane associated guanylate kinase, WW and PDZ domain containing 1 |
|  | ZAK | sterile alpha motif and leucine zipper containing kinase AZK |
|  | ZAK | sterile alpha motif and leucine zipper containing kinase AZK |
|  | ZAK | sterile alpha motif and leucine zipper containing kinase AZK |
|  | CLDN10 | claudin 10 |
| **KEGG pathway----Vascular smooth muscle contraction----04270** | **Gene Symbol** | **Gene Name** |
|  | MYL9 | myosin, light chain 9, regulatory |
|  | BRAF | v-raf murine sarcoma viral oncogene homolog B1 |
|  | PPP1R12A | protein phosphatase 1, regulatory (inhibitor) subunit 12A |
|  | NPR2 | natriuretic peptide receptor B/guanylate cyclase B (atrionatriuretic peptide receptor B) |
|  | JMJD7-PLA2G4B | JMJD7-PLA2G4B readthrough |
|  | MAP2K1 | mitogen-activated protein kinase kinase 1 |
|  | PLA2G12A | phospholipase A2, group XIIA |
|  | ACTG2 | actin, gamma 2, smooth muscle, enteric |
|  | RAMP1 | receptor (G protein-coupled) activity modifying protein 1 |
|  | PRKCA | protein kinase C, alpha |
|  | MYL6B | myosin, light chain 6B, alkali, smooth muscle and non-muscle |
|  | PPP1CC | protein phosphatase 1, catalytic subunit, gamma isoform |
|  | CALM2 | calmodulin 2 (phosphorylase kinase, delta) |
|  | CALD1 | caldesmon 1 |
|  | CALD1 | caldesmon 1 |
|  | CALD1 | caldesmon 1 |
|  | CALD1 | caldesmon 1 |
|  | ADCY3 | adenylate cyclase 3 |
|  | ACTA2 | actin, alpha 2, smooth muscle, aorta |
|  | MYL6 | myosin, light chain 6, alkali, smooth muscle and non-muscle |
|  | PLA2G6 | phospholipase A2, group VI (cytosolic, calcium-independent) |
|  | ROCK2 | Rho-associated, coiled-coil containing protein kinase 2 |
|  | PRKACB | protein kinase, cAMP-dependent, catalytic, beta |
|  | ADCY7 | adenylate cyclase 7 |
|  | RHOA | ras homolog gene family, member A |
|  | RHOA | ras homolog gene family, member A |
|  | CALM1 | calmodulin 1 (phosphorylase kinase, delta) |
|  | MRVI1 | murine retrovirus integration site 1 homolog |
|  | ITPR3 | inositol 1,4,5-triphosphate receptor, type 3 |
|  | MAP2K2 | mitogen-activated protein kinase kinase 2 |
|  | EDNRA | endothelin receptor type A |
|  | PLA2G2A | phospholipase A2, group IIA (platelets, synovial fluid) |
|  | MYH11 | myosin, heavy chain 11, smooth muscle |
|  | MYH11 | myosin, heavy chain 11, smooth muscle |
|  | GNA12 | guanine nucleotide binding protein (G protein) alpha 12 |
|  | GUCY1B3 | guanylate cyclase 1, soluble, beta 3 |
|  | PLA2G1B | phospholipase A2, group IB (pancreas) |
|  | MYLK | myosin light chain kinase |
|  | ITPR1 | inositol 1,4,5-triphosphate receptor, type 1 |
|  | PPP1CB | protein phosphatase 1, catalytic subunit, beta isoform |
|  | PPP1CB | protein phosphatase 1, catalytic subunit, beta isoform |
|  | GNAQ | guanine nucleotide binding protein (G protein), q polypeptide |
|  | ROCK1 | Rho-associated, coiled-coil containing protein kinase 1 |
|  | ROCK1 | Rho-associated, coiled-coil containing protein kinase 1 |
|  | MAPK1 | mitogen-activated protein kinase 1 |
|  | MAPK1 | mitogen-activated protein kinase 1 |
|  | GUCY1A3 | guanylate cyclase 1, soluble, alpha 3 |
|  | GNA13 | guanine nucleotide binding protein (G protein), alpha 13 |
| **KEGG pathway----Propanoate metabolism----00640** | **Gene Symbol** | **Gene Name** |
|  | MCEE | methylmalonyl CoA epimerase |
|  | SUCLA2 | succinate-CoA ligase, ADP-forming, beta subunit |
|  | PCCB | propionyl Coenzyme A carboxylase, beta polypeptide |
|  | ACACB | acetyl-Coenzyme A carboxylase beta |
|  | ALDH2 | aldehyde dehydrogenase 2 family (mitochondrial) |
|  | EHHADH | enoyl-Coenzyme A, hydratase/3-hydroxyacyl Coenzyme A dehydrogenase |
|  | MUT | methylmalonyl Coenzyme A mutase |
|  | ALDH6A1 | aldehyde dehydrogenase 6 family, member A1 |
|  | SUCLG1 | succinate-CoA ligase, alpha subunit |
|  | HADHA | hydroxyacyl-Coenzyme A dehydrogenase/3-ketoacyl-Coenzyme A thiolase/enoyl-Coenzyme A hydratase (trifunctional protein), alpha subunit |
|  | ABAT | 4-aminobutyrate aminotransferase |
|  | ACAT2 | acetyl-Coenzyme A acetyltransferase 2 |
|  | ACAT2 | acetyl-Coenzyme A acetyltransferase 2 |
|  | ACAT2 | acetyl-Coenzyme A acetyltransferase 2 |
|  | ACAT1 | acetyl-Coenzyme A acetyltransferase 1 |
|  | ALDH7A1 | aldehyde dehydrogenase 7 family, member A1 |
|  | ALDH7A1 | aldehyde dehydrogenase 7 family, member A1 |
|  | HIBCH | 3-hydroxyisobutyryl-Coenzyme A hydrolase |
|  | ACADM | acyl-Coenzyme A dehydrogenase, C-4 to C-12 straight chain |
|  | ALDH3A2 | aldehyde dehydrogenase 3 family, member A2 |
|  | ACACA | acetyl-Coenzyme A carboxylase alpha |
|  | LDHB | lactate dehydrogenase B |
| **KEGG pathway----Vibrio cholerae infection----05110** | **Gene Symbol** | **Gene Name** |
|  | SEC61B | Sec61 beta subunit |
|  | ATP6V1A | ATPase, H+ transporting, lysosomal 70kDa, V1 subunit A |
|  | ATP6V1A | ATPase, H+ transporting, lysosomal 70kDa, V1 subunit A |
|  | SEC61G | Sec61 gamma subunit |
|  | ERO1L | ERO1-like (S. cerevisiae) |
|  | ERO1L | ERO1-like (S. cerevisiae) |
|  | KDELR3 | KDEL (Lys-Asp-Glu-Leu) endoplasmic reticulum protein retention receptor 3 |
|  | KDELR3 | KDEL (Lys-Asp-Glu-Leu) endoplasmic reticulum protein retention receptor 3 |
|  | MUC2 | mucin 2, oligomeric mucus/gel-forming |
|  | KCNQ1 | potassium voltage-gated channel, KQT-like subfamily, member 1 |
|  | ARF1 | ADP-ribosylation factor 1 |
|  | CFTR | cystic fibrosis transmembrane conductance regulator (ATP-binding cassette sub-family C, member 7) |
|  | ATP6V1B2 | ATPase, H+ transporting, lysosomal 56/58kDa, V1 subunit B2 |
|  | PRKCA | protein kinase C, alpha |
|  | KDELR2 | KDEL (Lys-Asp-Glu-Leu) endoplasmic reticulum protein retention receptor 2 |
|  | KDELR2 | KDEL (Lys-Asp-Glu-Leu) endoplasmic reticulum protein retention receptor 2 |
|  | PDIA4 | protein disulfide isomerase family A, member 4 |
|  | TJP2 | tight junction protein 2 (zona occludens 2) |
|  | TJP2 | tight junction protein 2 (zona occludens 2) |
|  | ATP6V0B | ATPase, H+ transporting, lysosomal 21kDa, V0 subunit b |
|  | TCIRG1 | T-cell, immune regulator 1, ATPase, H+ transporting, lysosomal V0 subunit A3 |
|  | SEC61A1 | Sec61 alpha 1 subunit (S. cerevisiae) |
|  | ADCY3 | adenylate cyclase 3 |
|  | ATP6V1C1 | ATPase, H+ transporting, lysosomal 42kDa, V1 subunit C1 |
|  | ATP6V1C1 | ATPase, H+ transporting, lysosomal 42kDa, V1 subunit C1 |
|  | ATP6V1E1 | ATPase, H+ transporting, lysosomal 31kDa, V1 subunit E1 |
|  | SLC12A2 | solute carrier family 12 (sodium/potassium/chloride transporters), member 2 |
|  | ATP6V0E1 | ATPase, H+ transporting, lysosomal 9kDa, V0 subunit e1 |
|  | ATP6V0E1 | ATPase, H+ transporting, lysosomal 9kDa, V0 subunit e1 |
|  | PRKACB | protein kinase, cAMP-dependent, catalytic, beta |
|  | KDELR1 | KDEL (Lys-Asp-Glu-Leu) endoplasmic reticulum protein retention receptor 1 |
|  | ATP6V1G1 | ATPase, H+ transporting, lysosomal 13kDa, V1 subunit G1 |
| **KEGG pathway----Steroid biosynthesis----00100** | **Gene Symbol** | **Gene Name** |
|  | CYP51A1 | cytochrome P450, family 51, subfamily A, polypeptide 1 |
|  | SOAT1 | sterol O-acyltransferase 1 |
|  | LSS | lanosterol synthase (2,3-oxidosqualene-lanosterol cyclase) |
|  | SC4MOL | sterol-C4-methyl oxidase-like |
|  | NSDHL | NAD(P) dependent steroid dehydrogenase-like |
|  | DHCR24 | 24-dehydrocholesterol reductase |
|  | CEL | carboxyl ester lipase (bile salt-stimulated lipase) |
|  | CEL | carboxyl ester lipase (bile salt-stimulated lipase) |
|  | TM7SF2 | transmembrane 7 superfamily member 2 |
|  | SQLE | squalene epoxidase |
|  | SQLE | squalene epoxidase |
|  | SQLE | squalene epoxidase |
|  | LIPA | lipase A, lysosomal acid, cholesterol esterase |
|  | DHCR7 | 7-dehydrocholesterol reductase |
|  | DHCR7 | 7-dehydrocholesterol reductase |
|  | FDFT1 | farnesyl-diphosphate farnesyltransferase 1 |
|  | FDFT1 | farnesyl-diphosphate farnesyltransferase 1 |
|  | EBP | emopamil binding protein (sterol isomerase) |
| **KEGG pathway----T cell receptor signaling pathway----04660** | **Gene Symbol** | **Gene Name** |
|  | CARD11 | caspase recruitment domain family, member 11 |
|  | MAP2K1 | mitogen-activated protein kinase kinase 1 |
|  | PPP3CB | protein phosphatase 3 (formerly 2B), catalytic subunit, beta isoform |
|  | LCP2 | lymphocyte cytosolic protein 2 (SH2 domain containing leukocyte protein of 76kDa) |
|  | NFAT5 | nuclear factor of activated T-cells 5, tonicity-responsive |
|  | NFAT5 | nuclear factor of activated T-cells 5, tonicity-responsive |
|  | JUN | jun oncogene |
|  | JUN | jun oncogene |
|  | JUN | jun oncogene |
|  | NFATC3 | nuclear factor of activated T-cells, cytoplasmic, calcineurin-dependent 3 |
|  | IKBKB | inhibitor of kappa light polypeptide gene enhancer in B-cells, kinase beta |
|  | IKBKB | inhibitor of kappa light polypeptide gene enhancer in B-cells, kinase beta |
|  | PAK2 | p21 protein (Cdc42/Rac)-activated kinase 2 |
|  | PAK2 | p21 protein (Cdc42/Rac)-activated kinase 2 |
|  | PAK2 | p21 protein (Cdc42/Rac)-activated kinase 2 |
|  | MAPK9 | mitogen-activated protein kinase 9 |
|  | DLG1 | discs, large homolog 1 (Drosophila) |
|  | DLG1 | discs, large homolog 1 (Drosophila) |
|  | PIK3CA | phosphoinositide-3-kinase, catalytic, alpha polypeptide |
|  | CD3D | CD3d molecule, delta (CD3-TCR complex) |
|  | MAP3K7 | mitogen-activated protein kinase kinase kinase 7 |
|  | MAP3K7 | mitogen-activated protein kinase kinase kinase 7 |
|  | RASGRP1 | RAS guanyl releasing protein 1 (calcium and DAG-regulated) |
|  | PAK1 | p21 protein (Cdc42/Rac)-activated kinase 1 |
|  | PAK1 | p21 protein (Cdc42/Rac)-activated kinase 1 |
|  | VAV3 | vav 3 guanine nucleotide exchange factor |
|  | PPP3CA | protein phosphatase 3 (formerly 2B), catalytic subunit, alpha isoform |
|  | PPP3CA | protein phosphatase 3 (formerly 2B), catalytic subunit, alpha isoform |
|  | NRAS | neuroblastoma RAS viral (v-ras) oncogene homolog |
|  | RHOA | ras homolog gene family, member A |
|  | RHOA | ras homolog gene family, member A |
|  | PDK1 | pyruvate dehydrogenase kinase, isozyme 1 |
|  | SOS1 | son of sevenless homolog 1 (Drosophila) |
|  | NCK1 | NCK adaptor protein 1 |
|  | NCK1 | NCK adaptor protein 1 |
|  | CDK4 | cyclin-dependent kinase 4 |
|  | PTPN6 | protein tyrosine phosphatase, non-receptor type 6 |
|  | CBLB | Cas-Br-M (murine) ecotropic retroviral transforming sequence b |
|  | MALT1 | mucosa associated lymphoid tissue lymphoma translocation gene 1 |
|  | MALT1 | mucosa associated lymphoid tissue lymphoma translocation gene 1 |
|  | FOS | FBJ murine osteosarcoma viral oncogene homolog |
|  | MAP2K2 | mitogen-activated protein kinase kinase 2 |
|  | PIK3CB | phosphoinositide-3-kinase, catalytic, beta polypeptide |
|  | GRB2 | growth factor receptor-bound protein 2 |
|  | PTPRC | protein tyrosine phosphatase, receptor type, C |
|  | PTPRC | protein tyrosine phosphatase, receptor type, C |
|  | FYN | FYN oncogene related to SRC, FGR, YES |
|  | AKT3 | v-akt murine thymoma viral oncogene homolog 3 (protein kinase B, gamma) |
|  | AKT3 | v-akt murine thymoma viral oncogene homolog 3 (protein kinase B, gamma) |
|  | AKT3 | v-akt murine thymoma viral oncogene homolog 3 (protein kinase B, gamma) |
|  | AKT3 | v-akt murine thymoma viral oncogene homolog 3 (protein kinase B, gamma) |
|  | NFKBIA | nuclear factor of kappa light polypeptide gene enhancer in B-cells inhibitor, alpha |
|  | PPP3R1 | protein phosphatase 3 (formerly 2B), regulatory subunit B, alpha isoform |
|  | MAPK1 | mitogen-activated protein kinase 1 |
|  | MAPK1 | mitogen-activated protein kinase 1 |
| **KEGG pathway----Gap junction----04540** | **Gene Symbol** | **Gene Name** |
|  | PDGFC | platelet derived growth factor C |
|  | TUBB2A | tubulin, beta 2A |
|  | NRAS | neuroblastoma RAS viral (v-ras) oncogene homolog |
|  | TUBA1A | tubulin, alpha 1a |
|  | GJA1 | gap junction protein, alpha 1, 43kDa |
|  | SOS1 | son of sevenless homolog 1 (Drosophila) |
|  | MAP2K1 | mitogen-activated protein kinase kinase 1 |
|  | TUBA1C | tubulin, alpha 1c |
|  | TUBB6 | tubulin, beta 6 |
|  | PRKCA | protein kinase C, alpha |
|  | ITPR3 | inositol 1,4,5-triphosphate receptor, type 3 |
|  | PDGFRA | platelet-derived growth factor receptor, alpha polypeptide |
|  | GNAI3 | guanine nucleotide binding protein (G protein), alpha inhibiting activity polypeptide 3 |
|  | GNAI3 | guanine nucleotide binding protein (G protein), alpha inhibiting activity polypeptide 3 |
|  | GNAI3 | guanine nucleotide binding protein (G protein), alpha inhibiting activity polypeptide 3 |
|  | SRC | v-src sarcoma (Schmidt-Ruppin A-2) viral oncogene homolog (avian) |
|  | CDC2 | cell division cycle 2, G1 to S and G2 to M |
|  | CDC2 | cell division cycle 2, G1 to S and G2 to M |
|  | CDC2 | cell division cycle 2, G1 to S and G2 to M |
|  | MAP2K2 | mitogen-activated protein kinase kinase 2 |
|  | EGFR | epidermal growth factor receptor (erythroblastic leukemia viral (v-erb-b) oncogene homolog, avian) |
|  | EGFR | epidermal growth factor receptor (erythroblastic leukemia viral (v-erb-b) oncogene homolog, avian) |
|  | EGFR | epidermal growth factor receptor (erythroblastic leukemia viral (v-erb-b) oncogene homolog, avian) |
|  | TUBA1B | tubulin, alpha 1b |
|  | GRB2 | growth factor receptor-bound protein 2 |
|  | LPAR1 | lysophosphatidic acid receptor 1 |
|  | GUCY1B3 | guanylate cyclase 1, soluble, beta 3 |
|  | ITPR1 | inositol 1,4,5-triphosphate receptor, type 1 |
|  | ADCY3 | adenylate cyclase 3 |
|  | GNAQ | guanine nucleotide binding protein (G protein), q polypeptide |
|  | TUBA3D | tubulin, alpha 3d |
|  | GUCY1A3 | guanylate cyclase 1, soluble, alpha 3 |
|  | MAPK1 | mitogen-activated protein kinase 1 |
|  | MAPK1 | mitogen-activated protein kinase 1 |
|  | PRKACB | protein kinase, cAMP-dependent, catalytic, beta |
|  | CSNK1D | casein kinase 1, delta |
|  | TUBB | tubulin, beta |
|  | TUBB | tubulin, beta |
|  | TUBB | tubulin, beta |
|  | PDGFRB | platelet-derived growth factor receptor, beta polypeptide |
|  | TUBB3 | tubulin, beta 3 |
|  | ADCY7 | adenylate cyclase 7 |
| **KEGG pathway----Progesterone-mediated oocyte maturation----04914** | **Gene Symbol** | **Gene Name** |
|  | MAPK8 | mitogen-activated protein kinase 8 |
|  | BRAF | v-raf murine sarcoma viral oncogene homolog B1 |
|  | MAP2K1 | mitogen-activated protein kinase kinase 1 |
|  | CCNB1 | cyclin B1 |
|  | CCNB1 | cyclin B1 |
|  | MAD2L2 | MAD2 mitotic arrest deficient-like 2 (yeast) |
|  | CCNA2 | cyclin A2 |
|  | CCNA2 | cyclin A2 |
|  | ANAPC11 | anaphase promoting complex subunit 11 |
|  | MAPK9 | mitogen-activated protein kinase 9 |
|  | ANAPC10 | anaphase promoting complex subunit 10 |
|  | PIK3CA | phosphoinositide-3-kinase, catalytic, alpha polypeptide |
|  | ADCY3 | adenylate cyclase 3 |
|  | BUB1 | budding uninhibited by benzimidazoles 1 homolog (yeast) |
|  | CDC25B | cell division cycle 25 homolog B (S. pombe) |
|  | PRKACB | protein kinase, cAMP-dependent, catalytic, beta |
|  | ADCY7 | adenylate cyclase 7 |
|  | CDC25A | cell division cycle 25 homolog A (S. pombe) |
|  | HSP90AB1 | heat shock protein 90kDa alpha (cytosolic), class B member 1 |
|  | HSP90AB1 | heat shock protein 90kDa alpha (cytosolic), class B member 1 |
|  | CDC23 | cell division cycle 23 homolog (S. cerevisiae) |
|  | CDC25C | cell division cycle 25 homolog C (S. pombe) |
|  | GNAI3 | guanine nucleotide binding protein (G protein), alpha inhibiting activity polypeptide 3 |
|  | GNAI3 | guanine nucleotide binding protein (G protein), alpha inhibiting activity polypeptide 3 |
|  | GNAI3 | guanine nucleotide binding protein (G protein), alpha inhibiting activity polypeptide 3 |
|  | CDC26 | cell division cycle 26 homolog (S. cerevisiae) |
|  | CDC2 | cell division cycle 2, G1 to S and G2 to M |
|  | CDC2 | cell division cycle 2, G1 to S and G2 to M |
|  | CDC2 | cell division cycle 2, G1 to S and G2 to M |
|  | PIK3CB | phosphoinositide-3-kinase, catalytic, beta polypeptide |
|  | INS | insulin |
|  | CDC27 | cell division cycle 27 homolog (S. cerevisiae) |
|  | CDC27 | cell division cycle 27 homolog (S. cerevisiae) |
|  | AKT3 | v-akt murine thymoma viral oncogene homolog 3 (protein kinase B, gamma) |
|  | AKT3 | v-akt murine thymoma viral oncogene homolog 3 (protein kinase B, gamma) |
|  | AKT3 | v-akt murine thymoma viral oncogene homolog 3 (protein kinase B, gamma) |
|  | AKT3 | v-akt murine thymoma viral oncogene homolog 3 (protein kinase B, gamma) |
|  | MAD2L1 | MAD2 mitotic arrest deficient-like 1 (yeast) |
|  | MAD2L1 | MAD2 mitotic arrest deficient-like 1 (yeast) |
|  | MAPK1 | mitogen-activated protein kinase 1 |
|  | MAPK1 | mitogen-activated protein kinase 1 |
|  | CCNB2 | cyclin B2 |
|  | CDK2 | cyclin-dependent kinase 2 |
|  | MAPK10 | mitogen-activated protein kinase 10 |
|  | RPS6KA3 | ribosomal protein S6 kinase, 90kDa, polypeptide 3 |
|  | RPS6KA3 | ribosomal protein S6 kinase, 90kDa, polypeptide 3 |
| **KEGG pathway----Leukocyte transendothelial migration----04670** | **Gene Symbol** | **Gene Name** |
|  | MMP9 | matrix metallopeptidase 9 (gelatinase B, 92kDa gelatinase, 92kDa type IV collagenase) |
|  | MYL9 | myosin, light chain 9, regulatory |
|  | CLDN1 | claudin 1 |
|  | CLDN1 | claudin 1 |
|  | CD99 | CD99 molecule |
|  | CD99 | CD99 molecule |
|  | CLDN5 | claudin 5 |
|  | CTNNA1 | catenin (cadherin-associated protein), alpha 1, 102kDa |
|  | PECAM1 | platelet/endothelial cell adhesion molecule |
|  | PRKCA | protein kinase C, alpha |
|  | ICAM1 | intercellular adhesion molecule 1 |
|  | RAC2 | ras-related C3 botulinum toxin substrate 2 (rho family, small GTP binding protein Rac2) |
|  | RAP1A | RAP1A, member of RAS oncogene family |
|  | MMP2 | matrix metallopeptidase 2 (gelatinase A, 72kDa gelatinase, 72kDa type IV collagenase) |
|  | PIK3CA | phosphoinositide-3-kinase, catalytic, alpha polypeptide |
|  | CLDN4 | claudin 4 |
|  | RAPGEF3 | Rap guanine nucleotide exchange factor (GEF) 3 |
|  | CLDN18 | claudin 18 |
|  | CLDN18 | claudin 18 |
|  | ROCK2 | Rho-associated, coiled-coil containing protein kinase 2 |
|  | CLDN7 | claudin 7 |
|  | VAV3 | vav 3 guanine nucleotide exchange factor |
|  | RHOA | ras homolog gene family, member A |
|  | RHOA | ras homolog gene family, member A |
|  | PXN | paxillin |
|  | CLDN23 | claudin 23 |
|  | CLDN23 | claudin 23 |
|  | ARHGAP5 | Rho GTPase activating protein 5 |
|  | EZR | ezrin |
|  | ACTN1 | actinin, alpha 1 |
|  | ACTN1 | actinin, alpha 1 |
|  | ACTN1 | actinin, alpha 1 |
|  | PTK2 | PTK2 protein tyrosine kinase 2 |
|  | PTK2 | PTK2 protein tyrosine kinase 2 |
|  | GNAI3 | guanine nucleotide binding protein (G protein), alpha inhibiting activity polypeptide 3 |
|  | GNAI3 | guanine nucleotide binding protein (G protein), alpha inhibiting activity polypeptide 3 |
|  | GNAI3 | guanine nucleotide binding protein (G protein), alpha inhibiting activity polypeptide 3 |
|  | PIK3CB | phosphoinositide-3-kinase, catalytic, beta polypeptide |
|  | ITGB2 | integrin, beta 2 (complement component 3 receptor 3 and 4 subunit) |
|  | THY1 | Thy-1 cell surface antigen |
|  | THY1 | Thy-1 cell surface antigen |
|  | THY1 | Thy-1 cell surface antigen |
|  | CXCR4 | chemokine (C-X-C motif) receptor 4 |
|  | CXCR4 | chemokine (C-X-C motif) receptor 4 |
|  | CYBA | cytochrome b-245, alpha polypeptide |
|  | CLDN2 | claudin 2 |
|  | ITGB1 | integrin, beta 1 (fibronectin receptor, beta polypeptide, antigen CD29 includes MDF2, MSK12) |
|  | ITGB1 | integrin, beta 1 (fibronectin receptor, beta polypeptide, antigen CD29 includes MDF2, MSK12) |
|  | VCAM1 | vascular cell adhesion molecule 1 |
|  | CTNND1 | catenin (cadherin-associated protein), delta 1 |
|  | ROCK1 | Rho-associated, coiled-coil containing protein kinase 1 |
|  | ROCK1 | Rho-associated, coiled-coil containing protein kinase 1 |
|  | MSN | moesin |
|  | CLDN10 | claudin 10 |
| **KEGG pathway----Mismatch repair----03430** | **Gene Symbol** | **Gene Name** |
|  | RPA2 | replication protein A2, 32kDa |
|  | RFC4 | replication factor C (activator 1) 4, 37kDa |
|  | RFC3 | replication factor C (activator 1) 3, 38kDa |
|  | PCNA | proliferating cell nuclear antigen |
|  | RFC5 | replication factor C (activator 1) 5, 36.5kDa |
|  | RFC5 | replication factor C (activator 1) 5, 36.5kDa |
|  | MSH2 | mutS homolog 2, colon cancer, nonpolyposis type 1 (E. coli) |
|  | SSBP1 | single-stranded DNA binding protein 1 |
|  | SSBP1 | single-stranded DNA binding protein 1 |
|  | RFC1 | replication factor C (activator 1) 1, 145kDa |
|  | RFC1 | replication factor C (activator 1) 1, 145kDa |
|  | POLD3 | polymerase (DNA-directed), delta 3, accessory subunit |
|  | POLD1 | polymerase (DNA directed), delta 1, catalytic subunit 125kDa |
|  | RFC2 | replication factor C (activator 1) 2, 40kDa |
|  | RFC2 | replication factor C (activator 1) 2, 40kDa |
|  | RPA3 | replication protein A3, 14kDa |
|  | POLD2 | polymerase (DNA directed), delta 2, regulatory subunit 50kDa |
|  | MSH6 | mutS homolog 6 (E. coli) |
|  | MSH6 | mutS homolog 6 (E. coli) |
|  | RPA1 | replication protein A1, 70kDa |
| **KEGG pathway----NOD-like receptor signaling pathway----04621** | **Gene Symbol** | **Gene Name** |
|  | MAPK8 | mitogen-activated protein kinase 8 |
|  | CARD6 | caspase recruitment domain family, member 6 |
|  | HSP90B1 | heat shock protein 90kDa beta (Grp94), member 1 |
|  | HSP90B1 | heat shock protein 90kDa beta (Grp94), member 1 |
|  | HSP90B1 | heat shock protein 90kDa beta (Grp94), member 1 |
|  | TNFAIP3 | tumor necrosis factor, alpha-induced protein 3 |
|  | TNFAIP3 | tumor necrosis factor, alpha-induced protein 3 |
|  | NLRP1 | NLR family, pyrin domain containing 1 |
|  | BIRC3 | baculoviral IAP repeat-containing 3 |
|  | CCL2 | chemokine (C-C motif) ligand 2 |
|  | MAP3K7IP3 | mitogen-activated protein kinase kinase kinase 7 interacting protein 3 |
|  | HSP90AB1 | heat shock protein 90kDa alpha (cytosolic), class B member 1 |
|  | HSP90AB1 | heat shock protein 90kDa alpha (cytosolic), class B member 1 |
|  | XIAP | X-linked inhibitor of apoptosis |
|  | XIAP | X-linked inhibitor of apoptosis |
|  | CXCL2 | chemokine (C-X-C motif) ligand 2 |
|  | CCL5 | chemokine (C-C motif) ligand 5 |
|  | CCL5 | chemokine (C-C motif) ligand 5 |
|  | BIRC2 | baculoviral IAP repeat-containing 2 |
|  | SUGT1 | SGT1, suppressor of G2 allele of SKP1 (S. cerevisiae) |
|  | CARD8 | caspase recruitment domain family, member 8 |
|  | MAP3K7IP2 | mitogen-activated protein kinase kinase kinase 7 interacting protein 2 |
|  | MAP3K7IP2 | mitogen-activated protein kinase kinase kinase 7 interacting protein 2 |
|  | NFKBIA | nuclear factor of kappa light polypeptide gene enhancer in B-cells inhibitor, alpha |
|  | PYCARD | PYD and CARD domain containing |
|  | IL8 | interleukin 8 |
|  | IKBKB | inhibitor of kappa light polypeptide gene enhancer in B-cells, kinase beta |
|  | IKBKB | inhibitor of kappa light polypeptide gene enhancer in B-cells, kinase beta |
|  | MAPK9 | mitogen-activated protein kinase 9 |
|  | CXCL1 | chemokine (C-X-C motif) ligand 1 (melanoma growth stimulating activity, alpha) |
|  | MAP3K7 | mitogen-activated protein kinase kinase kinase 7 |
|  | MAP3K7 | mitogen-activated protein kinase kinase kinase 7 |
|  | MAPK10 | mitogen-activated protein kinase 10 |
|  | MAPK1 | mitogen-activated protein kinase 1 |
|  | MAPK1 | mitogen-activated protein kinase 1 |
|  | CASP1 | caspase 1, apoptosis-related cysteine peptidase (interleukin 1, beta, convertase) |
| **KEGG pathway----Pyruvate metabolism----00620** | **Gene Symbol** | **Gene Name** |
|  | PDHA1 | pyruvate dehydrogenase (lipoamide) alpha 1 |
|  | ACACB | acetyl-Coenzyme A carboxylase beta |
|  | ALDH2 | aldehyde dehydrogenase 2 family (mitochondrial) |
|  | ME3 | malic enzyme 3, NADP(+)-dependent, mitochondrial |
|  | MDH2 | malate dehydrogenase 2, NAD (mitochondrial) |
|  | GLO1 | glyoxalase I |
|  | PDHB | pyruvate dehydrogenase (lipoamide) beta |
|  | GRHPR | glyoxylate reductase/hydroxypyruvate reductase |
|  | GRHPR | glyoxylate reductase/hydroxypyruvate reductase |
|  | GRHPR | glyoxylate reductase/hydroxypyruvate reductase |
|  | ACAT2 | acetyl-Coenzyme A acetyltransferase 2 |
|  | ACAT2 | acetyl-Coenzyme A acetyltransferase 2 |
|  | ACAT2 | acetyl-Coenzyme A acetyltransferase 2 |
|  | AKR1B1 | aldo-keto reductase family 1, member B1 (aldose reductase) |
|  | ACAT1 | acetyl-Coenzyme A acetyltransferase 1 |
|  | ME1 | malic enzyme 1, NADP(+)-dependent, cytosolic |
|  | ALDH7A1 | aldehyde dehydrogenase 7 family, member A1 |
|  | ALDH7A1 | aldehyde dehydrogenase 7 family, member A1 |
|  | MDH1 | malate dehydrogenase 1, NAD (soluble) |
|  | ME2 | malic enzyme 2, NAD(+)-dependent, mitochondrial |
|  | ME2 | malic enzyme 2, NAD(+)-dependent, mitochondrial |
|  | DLD | dihydrolipoamide dehydrogenase |
|  | ALDH3A2 | aldehyde dehydrogenase 3 family, member A2 |
|  | ACACA | acetyl-Coenzyme A carboxylase alpha |
|  | DLAT | dihydrolipoamide S-acetyltransferase |
|  | LDHB | lactate dehydrogenase B |
| **KEGG pathway----Prostate cancer----05215** | **Gene Symbol** | **Gene Name** |
|  | BRAF | v-raf murine sarcoma viral oncogene homolog B1 |
|  | HSP90B1 | heat shock protein 90kDa beta (Grp94), member 1 |
|  | HSP90B1 | heat shock protein 90kDa beta (Grp94), member 1 |
|  | HSP90B1 | heat shock protein 90kDa beta (Grp94), member 1 |
|  | FOXO1 | forkhead box O1 |
|  | MAP2K1 | mitogen-activated protein kinase kinase 1 |
|  | PDGFRA | platelet-derived growth factor receptor, alpha polypeptide |
|  | CCNE1 | cyclin E1 |
|  | CCND1 | cyclin D1 |
|  | CCND1 | cyclin D1 |
|  | MDM2 | Mdm2 p53 binding protein homolog (mouse) |
|  | IKBKB | inhibitor of kappa light polypeptide gene enhancer in B-cells, kinase beta |
|  | IKBKB | inhibitor of kappa light polypeptide gene enhancer in B-cells, kinase beta |
|  | PIK3CA | phosphoinositide-3-kinase, catalytic, alpha polypeptide |
|  | CDKN1B | cyclin-dependent kinase inhibitor 1B (p27, Kip1) |
|  | CREB1 | cAMP responsive element binding protein 1 |
|  | PDGFRB | platelet-derived growth factor receptor, beta polypeptide |
|  | PDPK1 | 3-phosphoinositide dependent protein kinase-1 |
|  | PDGFC | platelet derived growth factor C |
|  | NRAS | neuroblastoma RAS viral (v-ras) oncogene homolog |
|  | CREB3L4 | cAMP responsive element binding protein 3-like 4 |
|  | SOS1 | son of sevenless homolog 1 (Drosophila) |
|  | HSP90AB1 | heat shock protein 90kDa alpha (cytosolic), class B member 1 |
|  | HSP90AB1 | heat shock protein 90kDa alpha (cytosolic), class B member 1 |
|  | E2F3 | E2F transcription factor 3 |
|  | MAP2K2 | mitogen-activated protein kinase kinase 2 |
|  | PIK3CB | phosphoinositide-3-kinase, catalytic, beta polypeptide |
|  | TCF7L2 | transcription factor 7-like 2 (T-cell specific, HMG-box) |
|  | TCF7L2 | transcription factor 7-like 2 (T-cell specific, HMG-box) |
|  | TCF7L2 | transcription factor 7-like 2 (T-cell specific, HMG-box) |
|  | TCF7L2 | transcription factor 7-like 2 (T-cell specific, HMG-box) |
|  | TCF7L2 | transcription factor 7-like 2 (T-cell specific, HMG-box) |
|  | TCF7L2 | transcription factor 7-like 2 (T-cell specific, HMG-box) |
|  | INS | insulin |
|  | FGFR1 | fibroblast growth factor receptor 1 |
|  | EGFR | epidermal growth factor receptor (erythroblastic leukemia viral (v-erb-b) oncogene homolog, avian) |
|  | EGFR | epidermal growth factor receptor (erythroblastic leukemia viral (v-erb-b) oncogene homolog, avian) |
|  | EGFR | epidermal growth factor receptor (erythroblastic leukemia viral (v-erb-b) oncogene homolog, avian) |
|  | GRB2 | growth factor receptor-bound protein 2 |
|  | FGFR2 | fibroblast growth factor receptor 2 |
|  | AKT3 | v-akt murine thymoma viral oncogene homolog 3 (protein kinase B, gamma) |
|  | AKT3 | v-akt murine thymoma viral oncogene homolog 3 (protein kinase B, gamma) |
|  | AKT3 | v-akt murine thymoma viral oncogene homolog 3 (protein kinase B, gamma) |
|  | AKT3 | v-akt murine thymoma viral oncogene homolog 3 (protein kinase B, gamma) |
|  | NFKBIA | nuclear factor of kappa light polypeptide gene enhancer in B-cells inhibitor, alpha |
|  | MAPK1 | mitogen-activated protein kinase 1 |
|  | MAPK1 | mitogen-activated protein kinase 1 |
|  | CDK2 | cyclin-dependent kinase 2 |
| **KEGG pathway----Glycerolipid metabolism----00561** | **Gene Symbol** | **Gene Name** |
|  | LIPF | lipase, gastric |
|  | ALDH2 | aldehyde dehydrogenase 2 family (mitochondrial) |
|  | DGKQ | diacylglycerol kinase, theta 110kDa |
|  | DGKA | diacylglycerol kinase, alpha 80kDa |
|  | PPAP2C | phosphatidic acid phosphatase type 2C |
|  | CEL | carboxyl ester lipase (bile salt-stimulated lipase) |
|  | CEL | carboxyl ester lipase (bile salt-stimulated lipase) |
|  | PNLIPRP1 | pancreatic lipase-related protein 1 |
|  | AGPAT3 | 1-acylglycerol-3-phosphate O-acyltransferase 3 |
|  | LCLAT1 | lysocardiolipin acyltransferase 1 |
|  | AKR1A1 | aldo-keto reductase family 1, member A1 (aldehyde reductase) |
|  | PPAP2A | phosphatidic acid phosphatase type 2A |
|  | PPAP2A | phosphatidic acid phosphatase type 2A |
|  | AGPAT2 | 1-acylglycerol-3-phosphate O-acyltransferase 2 (lysophosphatidic acid acyltransferase, beta) |
|  | PNLIPRP2 | pancreatic lipase-related protein 2 |
|  | PPAP2B | phosphatidic acid phosphatase type 2B |
|  | PPAP2B | phosphatidic acid phosphatase type 2B |
|  | PNLIP | pancreatic lipase |
|  | AKR1B1 | aldo-keto reductase family 1, member B1 (aldose reductase) |
|  | ALDH7A1 | aldehyde dehydrogenase 7 family, member A1 |
|  | ALDH7A1 | aldehyde dehydrogenase 7 family, member A1 |
|  | GLA | galactosidase, alpha |
|  | GPAM | glycerol-3-phosphate acyltransferase, mitochondrial |
|  | GPAM | glycerol-3-phosphate acyltransferase, mitochondrial |
|  | MGLL | monoglyceride lipase |
|  | MGLL | monoglyceride lipase |
|  | ALDH3A2 | aldehyde dehydrogenase 3 family, member A2 |
| **KEGG pathway----Glioma----05214** | **Gene Symbol** | **Gene Name** |
|  | BRAF | v-raf murine sarcoma viral oncogene homolog B1 |
|  | NRAS | neuroblastoma RAS viral (v-ras) oncogene homolog |
|  | SOS1 | son of sevenless homolog 1 (Drosophila) |
|  | CDK4 | cyclin-dependent kinase 4 |
|  | CALM1 | calmodulin 1 (phosphorylase kinase, delta) |
|  | MAP2K1 | mitogen-activated protein kinase kinase 1 |
|  | E2F3 | E2F transcription factor 3 |
|  | PRKCA | protein kinase C, alpha |
|  | PDGFRA | platelet-derived growth factor receptor, alpha polypeptide |
|  | PIK3CB | phosphoinositide-3-kinase, catalytic, beta polypeptide |
|  | MAP2K2 | mitogen-activated protein kinase kinase 2 |
|  | EGFR | epidermal growth factor receptor (erythroblastic leukemia viral (v-erb-b) oncogene homolog, avian) |
|  | EGFR | epidermal growth factor receptor (erythroblastic leukemia viral (v-erb-b) oncogene homolog, avian) |
|  | EGFR | epidermal growth factor receptor (erythroblastic leukemia viral (v-erb-b) oncogene homolog, avian) |
|  | SHC2 | SHC (Src homology 2 domain containing) transforming protein 2 |
|  | GRB2 | growth factor receptor-bound protein 2 |
|  | CCND1 | cyclin D1 |
|  | CCND1 | cyclin D1 |
|  | CALM2 | calmodulin 2 (phosphorylase kinase, delta) |
|  | CAMK2D | calcium/calmodulin-dependent protein kinase II delta |
|  | MDM2 | Mdm2 p53 binding protein homolog (mouse) |
|  | SHC1 | SHC (Src homology 2 domain containing) transforming protein 1 |
|  | AKT3 | v-akt murine thymoma viral oncogene homolog 3 (protein kinase B, gamma) |
|  | AKT3 | v-akt murine thymoma viral oncogene homolog 3 (protein kinase B, gamma) |
|  | AKT3 | v-akt murine thymoma viral oncogene homolog 3 (protein kinase B, gamma) |
|  | AKT3 | v-akt murine thymoma viral oncogene homolog 3 (protein kinase B, gamma) |
|  | PIK3CA | phosphoinositide-3-kinase, catalytic, alpha polypeptide |
|  | MAPK1 | mitogen-activated protein kinase 1 |
|  | MAPK1 | mitogen-activated protein kinase 1 |
|  | CDK6 | cyclin-dependent kinase 6 |
|  | CDK6 | cyclin-dependent kinase 6 |
|  | CAMK2G | calcium/calmodulin-dependent protein kinase II gamma |
|  | CDKN2A | cyclin-dependent kinase inhibitor 2A (melanoma, p16, inhibits CDK4) |
|  | PDGFRB | platelet-derived growth factor receptor, beta polypeptide |
| **KEGG pathway----Fc gamma R-mediated phagocytosis----04666** | **Gene Symbol** | **Gene Name** |
|  | WASF1 | WAS protein family, member 1 |
|  | INPP5D | inositol polyphosphate-5-phosphatase, 145kDa |
|  | MAP2K1 | mitogen-activated protein kinase kinase 1 |
|  | ARPC4 | actin related protein 2/3 complex, subunit 4, 20kDa |
|  | PRKCA | protein kinase C, alpha |
|  | CRKL | v-crk sarcoma virus CT10 oncogene homolog (avian)-like |
|  | RAC2 | ras-related C3 botulinum toxin substrate 2 (rho family, small GTP binding protein Rac2) |
|  | PPAP2A | phosphatidic acid phosphatase type 2A |
|  | PPAP2A | phosphatidic acid phosphatase type 2A |
|  | ASAP1 | ArfGAP with SH3 domain, ankyrin repeat and PH domain 1 |
|  | ASAP1 | ArfGAP with SH3 domain, ankyrin repeat and PH domain 1 |
|  | ASAP1 | ArfGAP with SH3 domain, ankyrin repeat and PH domain 1 |
|  | ARPC5 | actin related protein 2/3 complex, subunit 5, 16kDa |
|  | ARPC5 | actin related protein 2/3 complex, subunit 5, 16kDa |
|  | DNM2 | dynamin 2 |
|  | PIK3CA | phosphoinositide-3-kinase, catalytic, alpha polypeptide |
|  | PLA2G6 | phospholipase A2, group VI (cytosolic, calcium-independent) |
|  | SYK | spleen tyrosine kinase |
|  | SYK | spleen tyrosine kinase |
|  | LIMK2 | LIM domain kinase 2 |
|  | LIMK2 | LIM domain kinase 2 |
|  | CFL1 | cofilin 1 (non-muscle) |
|  | GAB2 | GRB2-associated binding protein 2 |
|  | PAK1 | p21 protein (Cdc42/Rac)-activated kinase 1 |
|  | PAK1 | p21 protein (Cdc42/Rac)-activated kinase 1 |
|  | VAV3 | vav 3 guanine nucleotide exchange factor |
|  | PPAP2C | phosphatidic acid phosphatase type 2C |
|  | ARPC1A | actin related protein 2/3 complex, subunit 1A, 41kDa |
|  | HCK | hemopoietic cell kinase |
|  | PIK3CB | phosphoinositide-3-kinase, catalytic, beta polypeptide |
|  | CFL2 | cofilin 2 (muscle) |
|  | CFL2 | cofilin 2 (muscle) |
|  | PTPRC | protein tyrosine phosphatase, receptor type, C |
|  | PTPRC | protein tyrosine phosphatase, receptor type, C |
|  | GSN | gelsolin (amyloidosis, Finnish type) |
|  | GSN | gelsolin (amyloidosis, Finnish type) |
|  | DNM1L | dynamin 1-like |
|  | AKT3 | v-akt murine thymoma viral oncogene homolog 3 (protein kinase B, gamma) |
|  | AKT3 | v-akt murine thymoma viral oncogene homolog 3 (protein kinase B, gamma) |
|  | AKT3 | v-akt murine thymoma viral oncogene homolog 3 (protein kinase B, gamma) |
|  | AKT3 | v-akt murine thymoma viral oncogene homolog 3 (protein kinase B, gamma) |
|  | PPAP2B | phosphatidic acid phosphatase type 2B |
|  | PPAP2B | phosphatidic acid phosphatase type 2B |
|  | FCGR2A | Fc fragment of IgG, low affinity IIa, receptor (CD32) |
|  | RPS6KB1 | ribosomal protein S6 kinase, 70kDa, polypeptide 1 |
|  | ARF6 | ADP-ribosylation factor 6 |
|  | ARF6 | ADP-ribosylation factor 6 |
|  | MAPK1 | mitogen-activated protein kinase 1 |
|  | MAPK1 | mitogen-activated protein kinase 1 |
| **KEGG pathway----Base excision repair----03410** | **Gene Symbol** | **Gene Name** |
|  | POLB | polymerase (DNA directed), beta |
|  | HMGB1 | high-mobility group box 1 |
|  | PARP1 | poly (ADP-ribose) polymerase 1 |
|  | PARP2 | poly (ADP-ribose) polymerase 2 |
|  | PARP4 | poly (ADP-ribose) polymerase family, member 4 |
|  | POLD3 | polymerase (DNA-directed), delta 3, accessory subunit |
|  | POLD1 | polymerase (DNA directed), delta 1, catalytic subunit 125kDa |
|  | XRCC1 | X-ray repair complementing defective repair in Chinese hamster cells 1 |
|  | POLE4 | polymerase (DNA-directed), epsilon 4 (p12 subunit) |
|  | POLE4 | polymerase (DNA-directed), epsilon 4 (p12 subunit) |
|  | MBD4 | methyl-CpG binding domain protein 4 |
|  | MBD4 | methyl-CpG binding domain protein 4 |
|  | TDG | thymine-DNA glycosylase |
|  | PCNA | proliferating cell nuclear antigen |
|  | NEIL1 | nei endonuclease VIII-like 1 (E. coli) |
|  | POLE2 | polymerase (DNA directed), epsilon 2 (p59 subunit) |
|  | APEX2 | APEX nuclease (apurinic/apyrimidinic endonuclease) 2 |
|  | POLE3 | polymerase (DNA directed), epsilon 3 (p17 subunit) |
|  | POLD2 | polymerase (DNA directed), delta 2, regulatory subunit 50kDa |
|  | FEN1 | flap structure-specific endonuclease 1 |
|  | FEN1 | flap structure-specific endonuclease 1 |
| **KEGG pathway----B cell receptor signaling pathway----04662** | **Gene Symbol** | **Gene Name** |
|  | PPP3CA | protein phosphatase 3 (formerly 2B), catalytic subunit, alpha isoform |
|  | PPP3CA | protein phosphatase 3 (formerly 2B), catalytic subunit, alpha isoform |
|  | VAV3 | vav 3 guanine nucleotide exchange factor |
|  | CARD11 | caspase recruitment domain family, member 11 |
|  | NRAS | neuroblastoma RAS viral (v-ras) oncogene homolog |
|  | SOS1 | son of sevenless homolog 1 (Drosophila) |
|  | PTPN6 | protein tyrosine phosphatase, non-receptor type 6 |
|  | INPP5D | inositol polyphosphate-5-phosphatase, 145kDa |
|  | MAP2K1 | mitogen-activated protein kinase kinase 1 |
|  | MALT1 | mucosa associated lymphoid tissue lymphoma translocation gene 1 |
|  | MALT1 | mucosa associated lymphoid tissue lymphoma translocation gene 1 |
|  | PPP3CB | protein phosphatase 3 (formerly 2B), catalytic subunit, beta isoform |
|  | FOS | FBJ murine osteosarcoma viral oncogene homolog |
|  | PIK3CB | phosphoinositide-3-kinase, catalytic, beta polypeptide |
|  | BLNK | B-cell linker |
|  | NFAT5 | nuclear factor of activated T-cells 5, tonicity-responsive |
|  | NFAT5 | nuclear factor of activated T-cells 5, tonicity-responsive |
|  | MAP2K2 | mitogen-activated protein kinase kinase 2 |
|  | IFITM1 | interferon induced transmembrane protein 1 (9-27) |
|  | IFITM1 | interferon induced transmembrane protein 1 (9-27) |
|  | GRB2 | growth factor receptor-bound protein 2 |
|  | JUN | jun oncogene |
|  | JUN | jun oncogene |
|  | JUN | jun oncogene |
|  | RAC2 | ras-related C3 botulinum toxin substrate 2 (rho family, small GTP binding protein Rac2) |
|  | NFATC3 | nuclear factor of activated T-cells, cytoplasmic, calcineurin-dependent 3 |
|  | AKT3 | v-akt murine thymoma viral oncogene homolog 3 (protein kinase B, gamma) |
|  | AKT3 | v-akt murine thymoma viral oncogene homolog 3 (protein kinase B, gamma) |
|  | AKT3 | v-akt murine thymoma viral oncogene homolog 3 (protein kinase B, gamma) |
|  | AKT3 | v-akt murine thymoma viral oncogene homolog 3 (protein kinase B, gamma) |
|  | NFKBIA | nuclear factor of kappa light polypeptide gene enhancer in B-cells inhibitor, alpha |
|  | IKBKB | inhibitor of kappa light polypeptide gene enhancer in B-cells, kinase beta |
|  | IKBKB | inhibitor of kappa light polypeptide gene enhancer in B-cells, kinase beta |
|  | PPP3R1 | protein phosphatase 3 (formerly 2B), regulatory subunit B, alpha isoform |
|  | PIK3CA | phosphoinositide-3-kinase, catalytic, alpha polypeptide |
|  | DAPP1 | dual adaptor of phosphotyrosine and 3-phosphoinositides |
|  | DAPP1 | dual adaptor of phosphotyrosine and 3-phosphoinositides |
|  | MAPK1 | mitogen-activated protein kinase 1 |
|  | MAPK1 | mitogen-activated protein kinase 1 |
|  | SYK | spleen tyrosine kinase |
|  | SYK | spleen tyrosine kinase |
| **KEGG pathway----Complement and coagulation cascades----04610** | **Gene Symbol** | **Gene Name** |
|  | C3 | complement component 3 |
|  | CD55 | CD55 molecule, decay accelerating factor for complement (Cromer blood group) |
|  | CD55 | CD55 molecule, decay accelerating factor for complement (Cromer blood group) |
|  | C4A | complement component 4A (Rodgers blood group) |
|  | PLAUR | plasminogen activator, urokinase receptor |
|  | PLAUR | plasminogen activator, urokinase receptor |
|  | PLAUR | plasminogen activator, urokinase receptor |
|  | CFI | complement factor I |
|  | TFPI | tissue factor pathway inhibitor (lipoprotein-associated coagulation inhibitor) |
|  | TFPI | tissue factor pathway inhibitor (lipoprotein-associated coagulation inhibitor) |
|  | TFPI | tissue factor pathway inhibitor (lipoprotein-associated coagulation inhibitor) |
|  | F13A1 | coagulation factor XIII, A1 polypeptide |
|  | C1R | complement component 1, r subcomponent |
|  | FGG | fibrinogen gamma chain |
|  | FGG | fibrinogen gamma chain |
|  | PLAT | plasminogen activator, tissue |
|  | F3 | coagulation factor III (thromboplastin, tissue factor) |
|  | C1QB | complement component 1, q subcomponent, B chain |
|  | CD59 | CD59 molecule, complement regulatory protein |
|  | CD59 | CD59 molecule, complement regulatory protein |
|  | SERPINA5 | serpin peptidase inhibitor, clade A (alpha-1 antiproteinase, antitrypsin), member 5 |
|  | PLAU | plasminogen activator, urokinase |
|  | PLAU | plasminogen activator, urokinase |
|  | SERPINA1 | serpin peptidase inhibitor, clade A (alpha-1 antiproteinase, antitrypsin), member 1 |
|  | SERPINA1 | serpin peptidase inhibitor, clade A (alpha-1 antiproteinase, antitrypsin), member 1 |
|  | SERPINA1 | serpin peptidase inhibitor, clade A (alpha-1 antiproteinase, antitrypsin), member 1 |
|  | SERPING1 | serpin peptidase inhibitor, clade G (C1 inhibitor), member 1 |
|  | C1QA | complement component 1, q subcomponent, A chain |
|  | VWF | von Willebrand factor |
|  | SERPINE1 | serpin peptidase inhibitor, clade E (nexin, plasminogen activator inhibitor type 1), member 1 |
|  | SERPINE1 | serpin peptidase inhibitor, clade E (nexin, plasminogen activator inhibitor type 1), member 1 |
|  | SERPINE1 | serpin peptidase inhibitor, clade E (nexin, plasminogen activator inhibitor type 1), member 1 |
|  | C1S | complement component 1, s subcomponent |
|  | CFB | complement factor B |
|  | PROS1 | protein S (alpha) |
|  | F2R | coagulation factor II (thrombin) receptor |
|  | C3AR1 | complement component 3a receptor 1 |
|  | THBD | thrombomodulin |
|  | THBD | thrombomodulin |
| **KEGG pathway----Fc epsilon RI signaling pathway----04664** | **Gene Symbol** | **Gene Name** |
|  | MAPK8 | mitogen-activated protein kinase 8 |
|  | VAV3 | vav 3 guanine nucleotide exchange factor |
|  | NRAS | neuroblastoma RAS viral (v-ras) oncogene homolog |
|  | PDK1 | pyruvate dehydrogenase kinase, isozyme 1 |
|  | FCER1G | Fc fragment of IgE, high affinity I, receptor for; gamma polypeptide |
|  | SOS1 | son of sevenless homolog 1 (Drosophila) |
|  | JMJD7-PLA2G4B | JMJD7-PLA2G4B readthrough |
|  | INPP5D | inositol polyphosphate-5-phosphatase, 145kDa |
|  | MAP2K1 | mitogen-activated protein kinase kinase 1 |
|  | PLA2G12A | phospholipase A2, group XIIA |
|  | LCP2 | lymphocyte cytosolic protein 2 (SH2 domain containing leukocyte protein of 76kDa) |
|  | PRKCA | protein kinase C, alpha |
|  | PIK3CB | phosphoinositide-3-kinase, catalytic, beta polypeptide |
|  | MAP2K2 | mitogen-activated protein kinase kinase 2 |
|  | PLA2G2A | phospholipase A2, group IIA (platelets, synovial fluid) |
|  | GRB2 | growth factor receptor-bound protein 2 |
|  | RAC2 | ras-related C3 botulinum toxin substrate 2 (rho family, small GTP binding protein Rac2) |
|  | FYN | FYN oncogene related to SRC, FGR, YES |
|  | AKT3 | v-akt murine thymoma viral oncogene homolog 3 (protein kinase B, gamma) |
|  | AKT3 | v-akt murine thymoma viral oncogene homolog 3 (protein kinase B, gamma) |
|  | AKT3 | v-akt murine thymoma viral oncogene homolog 3 (protein kinase B, gamma) |
|  | AKT3 | v-akt murine thymoma viral oncogene homolog 3 (protein kinase B, gamma) |
|  | PLA2G1B | phospholipase A2, group IB (pancreas) |
|  | MAPK9 | mitogen-activated protein kinase 9 |
|  | PIK3CA | phosphoinositide-3-kinase, catalytic, alpha polypeptide |
|  | MAPK10 | mitogen-activated protein kinase 10 |
|  | MAPK1 | mitogen-activated protein kinase 1 |
|  | MAPK1 | mitogen-activated protein kinase 1 |
|  | PLA2G6 | phospholipase A2, group VI (cytosolic, calcium-independent) |
|  | MAP2K4 | mitogen-activated protein kinase kinase 4 |
|  | SYK | spleen tyrosine kinase |
|  | SYK | spleen tyrosine kinase |
|  | GAB2 | GRB2-associated binding protein 2 |
| **KEGG pathway----Fatty acid metabolism----00071** | **Gene Symbol** | **Gene Name** |
|  | ACSL5 | acyl-CoA synthetase long-chain family member 5 |
|  | ACSL5 | acyl-CoA synthetase long-chain family member 5 |
|  | ADH7 | alcohol dehydrogenase 7 (class IV), mu or sigma polypeptide |
|  | ACSL1 | acyl-CoA synthetase long-chain family member 1 |
|  | ACSL1 | acyl-CoA synthetase long-chain family member 1 |
|  | ALDH2 | aldehyde dehydrogenase 2 family (mitochondrial) |
|  | EHHADH | enoyl-Coenzyme A, hydratase/3-hydroxyacyl Coenzyme A dehydrogenase |
|  | CPT2 | carnitine palmitoyltransferase 2 |
|  | HADHA | hydroxyacyl-Coenzyme A dehydrogenase/3-ketoacyl-Coenzyme A thiolase/enoyl-Coenzyme A hydratase (trifunctional protein), alpha subunit |
|  | DCI | dodecenoyl-Coenzyme A delta isomerase (3,2 trans-enoyl-Coenzyme A isomerase) |
|  | ADH5 | alcohol dehydrogenase 5 (class III), chi polypeptide |
|  | ADH5 | alcohol dehydrogenase 5 (class III), chi polypeptide |
|  | ACAT2 | acetyl-Coenzyme A acetyltransferase 2 |
|  | ACAT2 | acetyl-Coenzyme A acetyltransferase 2 |
|  | ACAT2 | acetyl-Coenzyme A acetyltransferase 2 |
|  | HADH | hydroxyacyl-Coenzyme A dehydrogenase |
|  | ACOX1 | acyl-Coenzyme A oxidase 1, palmitoyl |
|  | ACOX1 | acyl-Coenzyme A oxidase 1, palmitoyl |
|  | ACOX1 | acyl-Coenzyme A oxidase 1, palmitoyl |
|  | ACAA2 | acetyl-Coenzyme A acyltransferase 2 |
|  | ACAT1 | acetyl-Coenzyme A acetyltransferase 1 |
|  | ALDH7A1 | aldehyde dehydrogenase 7 family, member A1 |
|  | ALDH7A1 | aldehyde dehydrogenase 7 family, member A1 |
|  | ACSL4 | acyl-CoA synthetase long-chain family member 4 |
|  | ACADM | acyl-Coenzyme A dehydrogenase, C-4 to C-12 straight chain |
|  | ACADVL | acyl-Coenzyme A dehydrogenase, very long chain |
|  | ALDH3A2 | aldehyde dehydrogenase 3 family, member A2 |
| **KEGG pathway----Arginine and proline metabolism----00330** | **Gene Symbol** | **Gene Name** |
|  | ALDH2 | aldehyde dehydrogenase 2 family (mitochondrial) |
|  | GLUD2 | glutamate dehydrogenase 2 |
|  | LAP3 | leucine aminopeptidase 3 |
|  | ABP1 | amiloride binding protein 1 (amine oxidase (copper-containing)) |
|  | AMD1 | adenosylmethionine decarboxylase 1 |
|  | AMD1 | adenosylmethionine decarboxylase 1 |
|  | SAT1 | spermidine/spermine N1-acetyltransferase 1 |
|  | ASS1 | argininosuccinate synthetase 1 |
|  | GLUD1 | glutamate dehydrogenase 1 |
|  | OAT | ornithine aminotransferase (gyrate atrophy) |
|  | ODC1 | ornithine decarboxylase 1 |
|  | PYCR1 | pyrroline-5-carboxylate reductase 1 |
|  | GLS | glutaminase |
|  | GLS | glutaminase |
|  | GLS | glutaminase |
|  | CKMT1B | creatine kinase, mitochondrial 1B |
|  | ACY1 | aminoacylase 1 |
|  | P4HA2 | prolyl 4-hydroxylase, alpha polypeptide II |
|  | MAOA | monoamine oxidase A |
|  | MAOA | monoamine oxidase A |
|  | MAOA | monoamine oxidase A |
|  | ALDH7A1 | aldehyde dehydrogenase 7 family, member A1 |
|  | ALDH7A1 | aldehyde dehydrogenase 7 family, member A1 |
|  | CKB | creatine kinase, brain |
|  | ALDH18A1 | aldehyde dehydrogenase 18 family, member A1 |
|  | ALDH18A1 | aldehyde dehydrogenase 18 family, member A1 |
|  | ALDH3A2 | aldehyde dehydrogenase 3 family, member A2 |
|  | GATM | glycine amidinotransferase (L-arginine:glycine amidinotransferase) |
|  | SMS | spermine synthase |
| **KEGG pathway----Glutathione metabolism----00480** | **Gene Symbol** | **Gene Name** |
|  | GCLM | glutamate-cysteine ligase, modifier subunit |
|  | GCLM | glutamate-cysteine ligase, modifier subunit |
|  | GPX3 | glutathione peroxidase 3 (plasma) |
|  | GSTM2 | glutathione S-transferase mu 2 (muscle) |
|  | LAP3 | leucine aminopeptidase 3 |
|  | GCLC | glutamate-cysteine ligase, catalytic subunit |
|  | GCLC | glutamate-cysteine ligase, catalytic subunit |
|  | MGST3 | microsomal glutathione S-transferase 3 |
|  | RRM1 | ribonucleotide reductase M1 |
|  | RRM1 | ribonucleotide reductase M1 |
|  | RRM2 | ribonucleotide reductase M2 |
|  | MGST2 | microsomal glutathione S-transferase 2 |
|  | IDH1 | isocitrate dehydrogenase 1 (NADP+), soluble |
|  | IDH1 | isocitrate dehydrogenase 1 (NADP+), soluble |
|  | ODC1 | ornithine decarboxylase 1 |
|  | MGST1 | microsomal glutathione S-transferase 1 |
|  | MGST1 | microsomal glutathione S-transferase 1 |
|  | MGST1 | microsomal glutathione S-transferase 1 |
|  | PGD | phosphogluconate dehydrogenase |
|  | GSTK1 | glutathione S-transferase kappa 1 |
|  | GGCT | gamma-glutamyl cyclotransferase |
|  | GPX2 | glutathione peroxidase 2 (gastrointestinal) |
|  | GPX2 | glutathione peroxidase 2 (gastrointestinal) |
|  | GSTA4 | glutathione S-transferase alpha 4 |
|  | TXNDC12 | thioredoxin domain containing 12 (endoplasmic reticulum) |
|  | GSTM1 | glutathione S-transferase mu 1 |
|  | GPX7 | glutathione peroxidase 7 |
|  | SMS | spermine synthase |
| **KEGG pathway----Homologous recombination----03440** | **Gene Symbol** | **Gene Name** |
|  | RPA2 | replication protein A2, 32kDa |
|  | RAD51C | RAD51 homolog C (S. cerevisiae) |
|  | BRCA2 | breast cancer 2, early onset |
|  | RAD51 | RAD51 homolog (RecA homolog, E. coli) (S. cerevisiae) |
|  | XRCC2 | X-ray repair complementing defective repair in Chinese hamster cells 2 |
|  | MRE11A | MRE11 meiotic recombination 11 homolog A (S. cerevisiae) |
|  | SSBP1 | single-stranded DNA binding protein 1 |
|  | SSBP1 | single-stranded DNA binding protein 1 |
|  | EME1 | essential meiotic endonuclease 1 homolog 1 (S. pombe) |
|  | POLD3 | polymerase (DNA-directed), delta 3, accessory subunit |
|  | POLD1 | polymerase (DNA directed), delta 1, catalytic subunit 125kDa |
|  | RPA3 | replication protein A3, 14kDa |
|  | POLD2 | polymerase (DNA directed), delta 2, regulatory subunit 50kDa |
|  | RAD54B | RAD54 homolog B (S. cerevisiae) |
|  | NBN | nibrin |
|  | RPA1 | replication protein A1, 70kDa |
| **KEGG pathway----Citrate cycle (TCA cycle)----00020** | **Gene Symbol** | **Gene Name** |
|  | Gene Symbol | Gene Name |
|  | SUCLA2 | succinate-CoA ligase, ADP-forming, beta subunit |
|  | PDHA1 | pyruvate dehydrogenase (lipoamide) alpha 1 |
|  | SUCLG1 | succinate-CoA ligase, alpha subunit |
|  | SDHC | succinate dehydrogenase complex, subunit C, integral membrane protein, 15kDa |
|  | SDHC | succinate dehydrogenase complex, subunit C, integral membrane protein, 15kDa |
|  | MDH2 | malate dehydrogenase 2, NAD (mitochondrial) |
|  | IDH3A | isocitrate dehydrogenase 3 (NAD+) alpha |
|  | FH | fumarate hydratase |
|  | hCG_1776980 | hCG1776980 |
|  | IDH1 | isocitrate dehydrogenase 1 (NADP+), soluble |
|  | IDH1 | isocitrate dehydrogenase 1 (NADP+), soluble |
|  | PDHB | pyruvate dehydrogenase (lipoamide) beta |
|  | SDHD | succinate dehydrogenase complex, subunit D, integral membrane protein |
|  | SDHB | succinate dehydrogenase complex, subunit B, iron sulfur (Ip) |
|  | ACLY | ATP citrate lyase |
|  | MDH1 | malate dehydrogenase 1, NAD (soluble) |
|  | DLD | dihydrolipoamide dehydrogenase |
|  | DLAT | dihydrolipoamide S-acetyltransferase |
| **KEGG pathway----mTOR signaling pathway----04150** | **Gene Symbol** | **Gene Name** |
|  | PDPK1 | 3-phosphoinositide dependent protein kinase-1 |
|  | VEGFC | vascular endothelial growth factor C |
|  | BRAF | v-raf murine sarcoma viral oncogene homolog B1 |
|  | EIF4E2 | eukaryotic translation initiation factor 4E family member 2 |
|  | PRKAA1 | protein kinase, AMP-activated, alpha 1 catalytic subunit |
|  | VEGFA | vascular endothelial growth factor A |
|  | VEGFA | vascular endothelial growth factor A |
|  | VEGFA | vascular endothelial growth factor A |
|  | PIK3CB | phosphoinositide-3-kinase, catalytic, beta polypeptide |
|  | ULK2 | unc-51-like kinase 2 (C. elegans) |
|  | RHEB | Ras homolog enriched in brain |
|  | INS | insulin |
|  | DDIT4 | DNA-damage-inducible transcript 4 |
|  | TSC1 | tuberous sclerosis 1 |
|  | EIF4EBP1 | eukaryotic translation initiation factor 4E binding protein 1 |
|  | AKT3 | v-akt murine thymoma viral oncogene homolog 3 (protein kinase B, gamma) |
|  | AKT3 | v-akt murine thymoma viral oncogene homolog 3 (protein kinase B, gamma) |
|  | AKT3 | v-akt murine thymoma viral oncogene homolog 3 (protein kinase B, gamma) |
|  | AKT3 | v-akt murine thymoma viral oncogene homolog 3 (protein kinase B, gamma) |
|  | PIK3CA | phosphoinositide-3-kinase, catalytic, alpha polypeptide |
|  | CAB39 | calcium binding protein 39 |
|  | RPS6KB1 | ribosomal protein S6 kinase, 70kDa, polypeptide 1 |
|  | CAB39L | calcium binding protein 39-like |
|  | EIF4E | eukaryotic translation initiation factor 4E |
|  | EIF4E | eukaryotic translation initiation factor 4E |
|  | MAPK1 | mitogen-activated protein kinase 1 |
|  | MAPK1 | mitogen-activated protein kinase 1 |
|  | RPS6KA3 | ribosomal protein S6 kinase, 90kDa, polypeptide 3 |
|  | RPS6KA3 | ribosomal protein S6 kinase, 90kDa, polypeptide 3 |
| **KEGG pathway----Amino sugar and nucleotide sugar metabolism----00520** | **Gene Symbol** | **Gene Name** |
|  | GFPT1 | glutamine-fructose-6-phosphate transaminase 1 |
|  | GFPT1 | glutamine-fructose-6-phosphate transaminase 1 |
|  | CMAS | cytidine monophosphate N-acetylneuraminic acid synthetase |
|  | GNPNAT1 | glucosamine-phosphate N-acetyltransferase 1 |
|  | UGP2 | UDP-glucose pyrophosphorylase 2 |
|  | UGP2 | UDP-glucose pyrophosphorylase 2 |
|  | PGM2 | phosphoglucomutase 2 |
|  | PGM2 | phosphoglucomutase 2 |
|  | PGM2 | phosphoglucomutase 2 |
|  | UGDH | UDP-glucose dehydrogenase |
|  | GNE | glucosamine (UDP-N-acetyl)-2-epimerase/N-acetylmannosamine kinase |
|  | HEXB | hexosaminidase B (beta polypeptide) |
|  | GALK2 | galactokinase 2 |
|  | UXS1 | UDP-glucuronate decarboxylase 1 |
|  | UXS1 | UDP-glucuronate decarboxylase 1 |
|  | UAP1 | UDP-N-acteylglucosamine pyrophosphorylase 1 |
|  | PGM3 | phosphoglucomutase 3 |
|  | GNPDA2 | glucosamine-6-phosphate deaminase 2 |
|  | FPGT | fucose-1-phosphate guanylyltransferase |
|  | CYB5R1 | cytochrome b5 reductase 1 |
|  | GMDS | GDP-mannose 4,6-dehydratase |
|  | NANP | N-acetylneuraminic acid phosphatase |
|  | NAGK | N-acetylglucosamine kinase |
|  | GALE | UDP-galactose-4-epimerase |
| **KEGG pathway----Metabolism of xenobiotics by cytochrome P450----00980** | **Gene Symbol** | **Gene Name** |
|  | UGT1A1 | UDP glucuronosyltransferase 1 family, polypeptide A1 |
|  | ADH7 | alcohol dehydrogenase 7 (class IV), mu or sigma polypeptide |
|  | GSTM2 | glutathione S-transferase mu 2 (muscle) |
|  | CYP2C9 | cytochrome P450, family 2, subfamily C, polypeptide 9 |
|  | MGST3 | microsomal glutathione S-transferase 3 |
|  | CYP3A7 | cytochrome P450, family 3, subfamily A, polypeptide 7 |
|  | AKR1C1 | aldo-keto reductase family 1, member C1 (dihydrodiol dehydrogenase 1; 20-alpha (3-alpha)-hydroxysteroid dehydrogenase) |
|  | AKR1C1 | aldo-keto reductase family 1, member C1 (dihydrodiol dehydrogenase 1; 20-alpha (3-alpha)-hydroxysteroid dehydrogenase) |
|  | CYP3A43 | cytochrome P450, family 3, subfamily A, polypeptide 43 |
|  | MGST2 | microsomal glutathione S-transferase 2 |
|  | AKR1C3 | aldo-keto reductase family 1, member C3 (3-alpha hydroxysteroid dehydrogenase, type II) |
|  | MGST1 | microsomal glutathione S-transferase 1 |
|  | MGST1 | microsomal glutathione S-transferase 1 |
|  | MGST1 | microsomal glutathione S-transferase 1 |
|  | CYP3A4 | cytochrome P450, family 3, subfamily A, polypeptide 4 |
|  | UGT1A3 | UDP glucuronosyltransferase 1 family, polypeptide A3 |
|  | ALDH3B2 | aldehyde dehydrogenase 3 family, member B2 |
|  | ADH5 | alcohol dehydrogenase 5 (class III), chi polypeptide |
|  | ADH5 | alcohol dehydrogenase 5 (class III), chi polypeptide |
|  | GSTK1 | glutathione S-transferase kappa 1 |
|  | ALDH3B1 | aldehyde dehydrogenase 3 family, member B1 |
|  | ALDH3B1 | aldehyde dehydrogenase 3 family, member B1 |
|  | AKR1C2 | aldo-keto reductase family 1, member C2 (dihydrodiol dehydrogenase 2; bile acid binding protein; 3-alpha hydroxysteroid dehydrogenase, type III) |
|  | AKR1C2 | aldo-keto reductase family 1, member C2 (dihydrodiol dehydrogenase 2; bile acid binding protein; 3-alpha hydroxysteroid dehydrogenase, type III) |
|  | UGT1A9 | UDP glucuronosyltransferase 1 family, polypeptide A9 |
|  | ALDH1A3 | aldehyde dehydrogenase 1 family, member A3 |
|  | CYP3A5 | cytochrome P450, family 3, subfamily A, polypeptide 5 |
|  | UGT1A6 | UDP glucuronosyltransferase 1 family, polypeptide A6 |
|  | GSTA4 | glutathione S-transferase alpha 4 |
|  | GSTM1 | glutathione S-transferase mu 1 |
|  | CYP1B1 | cytochrome P450, family 1, subfamily B, polypeptide 1 |
|  | CYP1B1 | cytochrome P450, family 1, subfamily B, polypeptide 1 |
| **KEGG pathway----Renal cell carcinoma----05211** | **Gene Symbol** | **Gene Name** |
|  | PAK1 | p21 protein (Cdc42/Rac)-activated kinase 1 |
|  | PAK1 | p21 protein (Cdc42/Rac)-activated kinase 1 |
|  | TGFB1 | transforming growth factor, beta 1 |
|  | VEGFC | vascular endothelial growth factor C |
|  | BRAF | v-raf murine sarcoma viral oncogene homolog B1 |
|  | NRAS | neuroblastoma RAS viral (v-ras) oncogene homolog |
|  | EPAS1 | endothelial PAS domain protein 1 |
|  | SOS1 | son of sevenless homolog 1 (Drosophila) |
|  | RBX1 | ring-box 1 |
|  | FH | fumarate hydratase |
|  | MAP2K1 | mitogen-activated protein kinase kinase 1 |
|  | TCEB1 | transcription elongation factor B (SIII), polypeptide 1 (15kDa, elongin C) |
|  | TCEB1 | transcription elongation factor B (SIII), polypeptide 1 (15kDa, elongin C) |
|  | VEGFA | vascular endothelial growth factor A |
|  | VEGFA | vascular endothelial growth factor A |
|  | VEGFA | vascular endothelial growth factor A |
|  | PIK3CB | phosphoinositide-3-kinase, catalytic, beta polypeptide |
|  | MAP2K2 | mitogen-activated protein kinase kinase 2 |
|  | GRB2 | growth factor receptor-bound protein 2 |
|  | CRKL | v-crk sarcoma virus CT10 oncogene homolog (avian)-like |
|  | JUN | jun oncogene |
|  | JUN | jun oncogene |
|  | JUN | jun oncogene |
|  | RAP1A | RAP1A, member of RAS oncogene family |
|  | AKT3 | v-akt murine thymoma viral oncogene homolog 3 (protein kinase B, gamma) |
|  | AKT3 | v-akt murine thymoma viral oncogene homolog 3 (protein kinase B, gamma) |
|  | AKT3 | v-akt murine thymoma viral oncogene homolog 3 (protein kinase B, gamma) |
|  | AKT3 | v-akt murine thymoma viral oncogene homolog 3 (protein kinase B, gamma) |
|  | CUL2 | cullin 2 |
|  | CUL2 | cullin 2 |
|  | PAK2 | p21 protein (Cdc42/Rac)-activated kinase 2 |
|  | PAK2 | p21 protein (Cdc42/Rac)-activated kinase 2 |
|  | PAK2 | p21 protein (Cdc42/Rac)-activated kinase 2 |
|  | PIK3CA | phosphoinositide-3-kinase, catalytic, alpha polypeptide |
|  | EGLN3 | egl nine homolog 3 (C. elegans) |
|  | EGLN3 | egl nine homolog 3 (C. elegans) |
|  | MAPK1 | mitogen-activated protein kinase 1 |
|  | MAPK1 | mitogen-activated protein kinase 1 |
|  | EGLN1 | egl nine homolog 1 (C. elegans) |
| **KEGG pathway----Cell adhesion molecules (CAMs)----04514** | **Gene Symbol** | **Gene Name** |
|  | HLA-DRA | major histocompatibility complex, class II, DR alpha |
|  | HLA-DRA | major histocompatibility complex, class II, DR alpha |
|  | CLDN1 | claudin 1 |
|  | CLDN1 | claudin 1 |
|  | CD99 | CD99 molecule |
|  | CD99 | CD99 molecule |
|  | CLDN5 | claudin 5 |
|  | MPZL1 | myelin protein zero-like 1 |
|  | MPZL1 | myelin protein zero-like 1 |
|  | MPZL1 | myelin protein zero-like 1 |
|  | SDC4 | syndecan 4 |
|  | SDC2 | syndecan 2 |
|  | SDC2 | syndecan 2 |
|  | PECAM1 | platelet/endothelial cell adhesion molecule |
|  | SDC1 | syndecan 1 |
|  | SDC1 | syndecan 1 |
|  | ICAM1 | intercellular adhesion molecule 1 |
|  | NEO1 | neogenin homolog 1 (chicken) |
|  | CD58 | CD58 molecule |
|  | CD58 | CD58 molecule |
|  | CD58 | CD58 molecule |
|  | HLA-DPA1 | major histocompatibility complex, class II, DP alpha 1 |
|  | CDH1 | cadherin 1, type 1, E-cadherin (epithelial) |
|  | ITGB8 | integrin, beta 8 |
|  | HLA-DQB1 | major histocompatibility complex, class II, DQ beta 1 |
|  | HLA-DQB1 | major histocompatibility complex, class II, DQ beta 1 |
|  | PVRL1 | poliovirus receptor-related 1 (herpesvirus entry mediator C) |
|  | CLDN4 | claudin 4 |
|  | CDH3 | cadherin 3, type 1, P-cadherin (placental) |
|  | CLDN18 | claudin 18 |
|  | CLDN18 | claudin 18 |
|  | CLDN7 | claudin 7 |
|  | CLDN23 | claudin 23 |
|  | CLDN23 | claudin 23 |
|  | HLA-DQA1 | major histocompatibility complex, class II, DQ alpha 1 |
|  | HLA-DQA1 | major histocompatibility complex, class II, DQ alpha 1 |
|  | PTPRM | protein tyrosine phosphatase, receptor type, M |
|  | ICAM3 | intercellular adhesion molecule 3 |
|  | CADM1 | cell adhesion molecule 1 |
|  | CADM1 | cell adhesion molecule 1 |
|  | ICAM2 | intercellular adhesion molecule 2 |
|  | PTPRF | protein tyrosine phosphatase, receptor type, F |
|  | PTPRF | protein tyrosine phosphatase, receptor type, F |
|  | NRXN3 | neurexin 3 |
|  | PTPRC | protein tyrosine phosphatase, receptor type, C |
|  | PTPRC | protein tyrosine phosphatase, receptor type, C |
|  | ITGB2 | integrin, beta 2 (complement component 3 receptor 3 and 4 subunit) |
|  | VCAN | versican |
|  | VCAN | versican |
|  | VCAN | versican |
|  | VCAN | versican |
|  | VCAN | versican |
|  | CLDN2 | claudin 2 |
|  | ITGB1 | integrin, beta 1 (fibronectin receptor, beta polypeptide, antigen CD29 includes MDF2, MSK12) |
|  | ITGB1 | integrin, beta 1 (fibronectin receptor, beta polypeptide, antigen CD29 includes MDF2, MSK12) |
|  | VCAM1 | vascular cell adhesion molecule 1 |
|  | ITGAV | integrin, alpha V (vitronectin receptor, alpha polypeptide, antigen CD51) |
|  | HLA-DRB5 | major histocompatibility complex, class II, DR beta 5 |
|  | CLDN10 | claudin 10 |
| **KEGG pathway----Glycolysis / Gluconeogenesis----00010** | **Gene Symbol** | **Gene Name** |
|  | PDHA1 | pyruvate dehydrogenase (lipoamide) alpha 1 |
|  | ADH7 | alcohol dehydrogenase 7 (class IV), mu or sigma polypeptide |
|  | ALDH2 | aldehyde dehydrogenase 2 family (mitochondrial) |
|  | ENO2 | enolase 2 (gamma, neuronal) |
|  | PGM2 | phosphoglucomutase 2 |
|  | PGM2 | phosphoglucomutase 2 |
|  | PGM2 | phosphoglucomutase 2 |
|  | PFKM | phosphofructokinase, muscle |
|  | PDHB | pyruvate dehydrogenase (lipoamide) beta |
|  | ALDH3B2 | aldehyde dehydrogenase 3 family, member B2 |
|  | AKR1A1 | aldo-keto reductase family 1, member A1 (aldehyde reductase) |
|  | ADH5 | alcohol dehydrogenase 5 (class III), chi polypeptide |
|  | ADH5 | alcohol dehydrogenase 5 (class III), chi polypeptide |
|  | ALDH3B1 | aldehyde dehydrogenase 3 family, member B1 |
|  | ALDH3B1 | aldehyde dehydrogenase 3 family, member B1 |
|  | PGK1 | phosphoglycerate kinase 1 |
|  | PGK1 | phosphoglycerate kinase 1 |
|  | PGK1 | phosphoglycerate kinase 1 |
|  | PGK1 | phosphoglycerate kinase 1 |
|  | FBP1 | fructose-1,6-bisphosphatase 1 |
|  | ALDOC | aldolase C, fructose-bisphosphate |
|  | ALDH1A3 | aldehyde dehydrogenase 1 family, member A3 |
|  | ALDH7A1 | aldehyde dehydrogenase 7 family, member A1 |
|  | ALDH7A1 | aldehyde dehydrogenase 7 family, member A1 |
|  | DLD | dihydrolipoamide dehydrogenase |
|  | ENO1 | enolase 1, (alpha) |
|  | ALDOB | aldolase B, fructose-bisphosphate |
|  | ALDH3A2 | aldehyde dehydrogenase 3 family, member A2 |
|  | PFKP | phosphofructokinase, platelet |
|  | DLAT | dihydrolipoamide S-acetyltransferase |
|  | LDHB | lactate dehydrogenase B |
| **KEGG pathway----VEGF signaling pathway----04370** | **Gene Symbol** | **Gene Name** |
|  | PPP3CA | protein phosphatase 3 (formerly 2B), catalytic subunit, alpha isoform |
|  | PPP3CA | protein phosphatase 3 (formerly 2B), catalytic subunit, alpha isoform |
|  | PXN | paxillin |
|  | NRAS | neuroblastoma RAS viral (v-ras) oncogene homolog |
|  | JMJD7-PLA2G4B | JMJD7-PLA2G4B readthrough |
|  | MAP2K1 | mitogen-activated protein kinase kinase 1 |
|  | PLA2G12A | phospholipase A2, group XIIA |
|  | PTK2 | PTK2 protein tyrosine kinase 2 |
|  | PTK2 | PTK2 protein tyrosine kinase 2 |
|  | PPP3CB | protein phosphatase 3 (formerly 2B), catalytic subunit, beta isoform |
|  | VEGFA | vascular endothelial growth factor A |
|  | VEGFA | vascular endothelial growth factor A |
|  | VEGFA | vascular endothelial growth factor A |
|  | PRKCA | protein kinase C, alpha |
|  | SRC | v-src sarcoma (Schmidt-Ruppin A-2) viral oncogene homolog (avian) |
|  | PIK3CB | phosphoinositide-3-kinase, catalytic, beta polypeptide |
|  | NFAT5 | nuclear factor of activated T-cells 5, tonicity-responsive |
|  | NFAT5 | nuclear factor of activated T-cells 5, tonicity-responsive |
|  | MAP2K2 | mitogen-activated protein kinase kinase 2 |
|  | SHC2 | SHC (Src homology 2 domain containing) transforming protein 2 |
|  | PLA2G2A | phospholipase A2, group IIA (platelets, synovial fluid) |
|  | MAPKAPK2 | mitogen-activated protein kinase-activated protein kinase 2 |
|  | RAC2 | ras-related C3 botulinum toxin substrate 2 (rho family, small GTP binding protein Rac2) |
|  | PTGS2 | prostaglandin-endoperoxide synthase 2 (prostaglandin G/H synthase and cyclooxygenase) |
|  | PTGS2 | prostaglandin-endoperoxide synthase 2 (prostaglandin G/H synthase and cyclooxygenase) |
|  | NFATC3 | nuclear factor of activated T-cells, cytoplasmic, calcineurin-dependent 3 |
|  | AKT3 | v-akt murine thymoma viral oncogene homolog 3 (protein kinase B, gamma) |
|  | AKT3 | v-akt murine thymoma viral oncogene homolog 3 (protein kinase B, gamma) |
|  | AKT3 | v-akt murine thymoma viral oncogene homolog 3 (protein kinase B, gamma) |
|  | AKT3 | v-akt murine thymoma viral oncogene homolog 3 (protein kinase B, gamma) |
|  | PLA2G1B | phospholipase A2, group IB (pancreas) |
|  | PPP3R1 | protein phosphatase 3 (formerly 2B), regulatory subunit B, alpha isoform |
|  | PIK3CA | phosphoinositide-3-kinase, catalytic, alpha polypeptide |
|  | MAPK1 | mitogen-activated protein kinase 1 |
|  | MAPK1 | mitogen-activated protein kinase 1 |
|  | PLA2G6 | phospholipase A2, group VI (cytosolic, calcium-independent) |
| **KEGG pathway----GnRH signaling pathway----04912** | **Gene Symbol** | **Gene Name** |
|  | MAPK8 | mitogen-activated protein kinase 8 |
|  | NRAS | neuroblastoma RAS viral (v-ras) oncogene homolog |
|  | SOS1 | son of sevenless homolog 1 (Drosophila) |
|  | JMJD7-PLA2G4B | JMJD7-PLA2G4B readthrough |
|  | CALM1 | calmodulin 1 (phosphorylase kinase, delta) |
|  | MAP2K1 | mitogen-activated protein kinase kinase 1 |
|  | PLA2G12A | phospholipase A2, group XIIA |
|  | ITPR3 | inositol 1,4,5-triphosphate receptor, type 3 |
|  | PRKCA | protein kinase C, alpha |
|  | SRC | v-src sarcoma (Schmidt-Ruppin A-2) viral oncogene homolog (avian) |
|  | MAP2K2 | mitogen-activated protein kinase kinase 2 |
|  | EGFR | epidermal growth factor receptor (erythroblastic leukemia viral (v-erb-b) oncogene homolog, avian) |
|  | EGFR | epidermal growth factor receptor (erythroblastic leukemia viral (v-erb-b) oncogene homolog, avian) |
|  | EGFR | epidermal growth factor receptor (erythroblastic leukemia viral (v-erb-b) oncogene homolog, avian) |
|  | PLA2G2A | phospholipase A2, group IIA (platelets, synovial fluid) |
|  | GRB2 | growth factor receptor-bound protein 2 |
|  | JUN | jun oncogene |
|  | JUN | jun oncogene |
|  | JUN | jun oncogene |
|  | CALM2 | calmodulin 2 (phosphorylase kinase, delta) |
|  | CAMK2D | calcium/calmodulin-dependent protein kinase II delta |
|  | PLA2G1B | phospholipase A2, group IB (pancreas) |
|  | ITPR1 | inositol 1,4,5-triphosphate receptor, type 1 |
|  | MAPK9 | mitogen-activated protein kinase 9 |
|  | MMP2 | matrix metallopeptidase 2 (gelatinase A, 72kDa gelatinase, 72kDa type IV collagenase) |
|  | MMP14 | matrix metallopeptidase 14 (membrane-inserted) |
|  | ADCY3 | adenylate cyclase 3 |
|  | GNAQ | guanine nucleotide binding protein (G protein), q polypeptide |
|  | MAPK10 | mitogen-activated protein kinase 10 |
|  | MAPK1 | mitogen-activated protein kinase 1 |
|  | MAPK1 | mitogen-activated protein kinase 1 |
|  | PLA2G6 | phospholipase A2, group VI (cytosolic, calcium-independent) |
|  | MAP2K4 | mitogen-activated protein kinase kinase 4 |
|  | PRKACB | protein kinase, cAMP-dependent, catalytic, beta |
|  | CAMK2G | calcium/calmodulin-dependent protein kinase II gamma |
|  | ADCY7 | adenylate cyclase 7 |
| **KEGG pathway----Prion diseases----05020** | **Gene Symbol** | **Gene Name** |
|  | C1QB | complement component 1, q subcomponent, B chain |
|  | NOTCH1 | Notch homolog 1, translocation-associated (Drosophila) |
|  | STIP1 | stress-induced-phosphoprotein 1 |
|  | STIP1 | stress-induced-phosphoprotein 1 |
|  | FYN | FYN oncogene related to SRC, FGR, YES |
|  | EGR1 | early growth response 1 |
|  | LAMC1 | laminin, gamma 1 (formerly LAMB2) |
|  | C1QA | complement component 1, q subcomponent, A chain |
|  | HSPA5 | heat shock 70kDa protein 5 (glucose-regulated protein, 78kDa) |
|  | MAP2K1 | mitogen-activated protein kinase kinase 1 |
|  | BAX | BCL2-associated X protein |
|  | BAX | BCL2-associated X protein |
|  | CCL5 | chemokine (C-C motif) ligand 5 |
|  | CCL5 | chemokine (C-C motif) ligand 5 |
|  | MAPK1 | mitogen-activated protein kinase 1 |
|  | MAPK1 | mitogen-activated protein kinase 1 |
|  | MAP2K2 | mitogen-activated protein kinase kinase 2 |
|  | HSPA1A | heat shock 70kDa protein 1A |
|  | PRKACB | protein kinase, cAMP-dependent, catalytic, beta |
|  | PRNP | prion protein |
| **KEGG pathway----Apoptosis----04210** | **Gene Symbol** | **Gene Name** |
|  | PPP3CA | protein phosphatase 3 (formerly 2B), catalytic subunit, alpha isoform |
|  | PPP3CA | protein phosphatase 3 (formerly 2B), catalytic subunit, alpha isoform |
|  | BIRC3 | baculoviral IAP repeat-containing 3 |
|  | CASP6 | caspase 6, apoptosis-related cysteine peptidase |
|  | TNFRSF10B | tumor necrosis factor receptor superfamily, member 10b |
|  | XIAP | X-linked inhibitor of apoptosis |
|  | XIAP | X-linked inhibitor of apoptosis |
|  | AIFM1 | apoptosis-inducing factor, mitochondrion-associated, 1 |
|  | TNFSF10 | tumor necrosis factor (ligand) superfamily, member 10 |
|  | PPP3CB | protein phosphatase 3 (formerly 2B), catalytic subunit, beta isoform |
|  | BCL2L1 | BCL2-like 1 |
|  | PIK3CB | phosphoinositide-3-kinase, catalytic, beta polypeptide |
|  | BIRC2 | baculoviral IAP repeat-containing 2 |
|  | FAS | Fas (TNF receptor superfamily, member 6) |
|  | FAS | Fas (TNF receptor superfamily, member 6) |
|  | FAS | Fas (TNF receptor superfamily, member 6) |
|  | FAS | Fas (TNF receptor superfamily, member 6) |
|  | CFLAR | CASP8 and FADD-like apoptosis regulator |
|  | CFLAR | CASP8 and FADD-like apoptosis regulator |
|  | CFLAR | CASP8 and FADD-like apoptosis regulator |
|  | IL1R1 | interleukin 1 receptor, type I |
|  | ENDOD1 | endonuclease domain containing 1 |
|  | CAPN2 | calpain 2, (m/II) large subunit |
|  | IRAK1 | interleukin-1 receptor-associated kinase 1 |
|  | AKT3 | v-akt murine thymoma viral oncogene homolog 3 (protein kinase B, gamma) |
|  | AKT3 | v-akt murine thymoma viral oncogene homolog 3 (protein kinase B, gamma) |
|  | AKT3 | v-akt murine thymoma viral oncogene homolog 3 (protein kinase B, gamma) |
|  | AKT3 | v-akt murine thymoma viral oncogene homolog 3 (protein kinase B, gamma) |
|  | NFKBIA | nuclear factor of kappa light polypeptide gene enhancer in B-cells inhibitor, alpha |
|  | IKBKB | inhibitor of kappa light polypeptide gene enhancer in B-cells, kinase beta |
|  | IKBKB | inhibitor of kappa light polypeptide gene enhancer in B-cells, kinase beta |
|  | PPP3R1 | protein phosphatase 3 (formerly 2B), regulatory subunit B, alpha isoform |
|  | BAX | BCL2-associated X protein |
|  | BAX | BCL2-associated X protein |
|  | PIK3CA | phosphoinositide-3-kinase, catalytic, alpha polypeptide |
|  | PRKAR2A | protein kinase, cAMP-dependent, regulatory, type II, alpha |
|  | IL1RAP | interleukin 1 receptor accessory protein |
|  | PRKACB | protein kinase, cAMP-dependent, catalytic, beta |
|  | CAPN1 | calpain 1, (mu/I) large subunit |
|  | PRKAR1A | protein kinase, cAMP-dependent, regulatory, type I, alpha (tissue specific extinguisher 1) |
|  | PRKAR1A | protein kinase, cAMP-dependent, regulatory, type I, alpha (tissue specific extinguisher 1) |
|  | PRKAR1A | protein kinase, cAMP-dependent, regulatory, type I, alpha (tissue specific extinguisher 1) |
| **KEGG pathway----Glycerophospholipid metabolism----00564** | **Gene Symbol** | **Gene Name** |
|  | CDS2 | CDP-diacylglycerol synthase (phosphatidate cytidylyltransferase) 2 |
|  | JMJD7-PLA2G4B | JMJD7-PLA2G4B readthrough |
|  | DGKQ | diacylglycerol kinase, theta 110kDa |
|  | DGKA | diacylglycerol kinase, alpha 80kDa |
|  | PPAP2C | phosphatidic acid phosphatase type 2C |
|  | ETNK1 | ethanolamine kinase 1 |
|  | ETNK1 | ethanolamine kinase 1 |
|  | ETNK1 | ethanolamine kinase 1 |
|  | ETNK1 | ethanolamine kinase 1 |
|  | AGPAT3 | 1-acylglycerol-3-phosphate O-acyltransferase 3 |
|  | PLA2G12A | phospholipase A2, group XIIA |
|  | CHKB | choline kinase beta |
|  | LCLAT1 | lysocardiolipin acyltransferase 1 |
|  | PLA2G2A | phospholipase A2, group IIA (platelets, synovial fluid) |
|  | PPAP2A | phosphatidic acid phosphatase type 2A |
|  | PPAP2A | phosphatidic acid phosphatase type 2A |
|  | AGPAT2 | 1-acylglycerol-3-phosphate O-acyltransferase 2 (lysophosphatidic acid acyltransferase, beta) |
|  | GDE1 | glycerophosphodiester phosphodiesterase 1 |
|  | GDE1 | glycerophosphodiester phosphodiesterase 1 |
|  | PPAP2B | phosphatidic acid phosphatase type 2B |
|  | PPAP2B | phosphatidic acid phosphatase type 2B |
|  | PLA2G1B | phospholipase A2, group IB (pancreas) |
|  | PEMT | phosphatidylethanolamine N-methyltransferase |
|  | GPD2 | glycerol-3-phosphate dehydrogenase 2 (mitochondrial) |
|  | LYPLA1 | lysophospholipase I |
|  | GPAM | glycerol-3-phosphate acyltransferase, mitochondrial |
|  | GPAM | glycerol-3-phosphate acyltransferase, mitochondrial |
|  | NAT5 | N-acetyltransferase 5 (GCN5-related, putative) |
|  | CHPT1 | choline phosphotransferase 1 |
|  | GPD1L | glycerol-3-phosphate dehydrogenase 1-like |
|  | PLA2G6 | phospholipase A2, group VI (cytosolic, calcium-independent) |
| **KEGG pathway----Ether lipid metabolism----00565** | **Gene Symbol** | **Gene Name** |
|  | PPAP2A | phosphatidic acid phosphatase type 2A |
|  | PPAP2A | phosphatidic acid phosphatase type 2A |
|  | PAFAH1B3 | platelet-activating factor acetylhydrolase, isoform Ib, subunit 3 (29kDa) |
|  | AGPAT2 | 1-acylglycerol-3-phosphate O-acyltransferase 2 (lysophosphatidic acid acyltransferase, beta) |
|  | AGPS | alkylglycerone phosphate synthase |
|  | AGPS | alkylglycerone phosphate synthase |
|  | AGPS | alkylglycerone phosphate synthase |
|  | PAFAH1B1 | platelet-activating factor acetylhydrolase, isoform Ib, subunit 1 (45kDa) |
|  | PPAP2B | phosphatidic acid phosphatase type 2B |
|  | PPAP2B | phosphatidic acid phosphatase type 2B |
|  | PLA2G1B | phospholipase A2, group IB (pancreas) |
|  | JMJD7-PLA2G4B | JMJD7-PLA2G4B readthrough |
|  | PPAP2C | phosphatidic acid phosphatase type 2C |
|  | AGPAT3 | 1-acylglycerol-3-phosphate O-acyltransferase 3 |
|  | PLA2G12A | phospholipase A2, group XIIA |
|  | ENPP2 | ectonucleotide pyrophosphatase/phosphodiesterase 2 |
|  | LCLAT1 | lysocardiolipin acyltransferase 1 |
|  | CHPT1 | choline phosphotransferase 1 |
|  | PLA2G6 | phospholipase A2, group VI (cytosolic, calcium-independent) |
|  | PLA2G2A | phospholipase A2, group IIA (platelets, synovial fluid) |
| **KEGG pathway----Sphingolipid metabolism----00600** | **Gene Symbol** | **Gene Name** |
|  | SGMS1 | sphingomyelin synthase 1 |
|  | PPAP2C | phosphatidic acid phosphatase type 2C |
|  | GBA | glucosidase, beta; acid (includes glucosylceramidase) |
|  | ASAH1 | N-acylsphingosine amidohydrolase (acid ceramidase) 1 |
|  | ENPP7 | ectonucleotide pyrophosphatase/phosphodiesterase 7 |
|  | SPTLC2 | serine palmitoyltransferase, long chain base subunit 2 |
|  | NEU1 | sialidase 1 (lysosomal sialidase) |
|  | SGPP1 | sphingosine-1-phosphate phosphatase 1 |
|  | SGPP1 | sphingosine-1-phosphate phosphatase 1 |
|  | SGPL1 | sphingosine-1-phosphate lyase 1 |
|  | SGPL1 | sphingosine-1-phosphate lyase 1 |
|  | KDSR | 3-ketodihydrosphingosine reductase |
|  | KDSR | 3-ketodihydrosphingosine reductase |
|  | PPAP2A | phosphatidic acid phosphatase type 2A |
|  | PPAP2A | phosphatidic acid phosphatase type 2A |
|  | PPAP2B | phosphatidic acid phosphatase type 2B |
|  | PPAP2B | phosphatidic acid phosphatase type 2B |
|  | DEGS1 | degenerative spermatocyte homolog 1, lipid desaturase (Drosophila) |
|  | DEGS1 | degenerative spermatocyte homolog 1, lipid desaturase (Drosophila) |
|  | SGMS2 | sphingomyelin synthase 2 |
|  | GLB1 | galactosidase, beta 1 |
|  | GLA | galactosidase, alpha |
|  | SPTLC1 | serine palmitoyltransferase, long chain base subunit 1 |
| **KEGG pathway----Arrhythmogenic right ventricular cardiomyopathy (ARVC)----05412** | **Gene Symbol** | **Gene Name** |
|  | GJA1 | gap junction protein, alpha 1, 43kDa |
|  | DMD | dystrophin |
|  | JUP | junction plakoglobin |
|  | DES | desmin |
|  | CTNNA1 | catenin (cadherin-associated protein), alpha 1, 102kDa |
|  | PKP2 | plakophilin 2 |
|  | ITGA5 | integrin, alpha 5 (fibronectin receptor, alpha polypeptide) |
|  | ACTN1 | actinin, alpha 1 |
|  | ACTN1 | actinin, alpha 1 |
|  | ACTN1 | actinin, alpha 1 |
|  | ITGB5 | integrin, beta 5 |
|  | ITGB5 | integrin, beta 5 |
|  | DSP | desmoplakin |
|  | SLC8A1 | solute carrier family 8 (sodium/calcium exchanger), member 1 |
|  | TCF7L2 | transcription factor 7-like 2 (T-cell specific, HMG-box) |
|  | TCF7L2 | transcription factor 7-like 2 (T-cell specific, HMG-box) |
|  | TCF7L2 | transcription factor 7-like 2 (T-cell specific, HMG-box) |
|  | TCF7L2 | transcription factor 7-like 2 (T-cell specific, HMG-box) |
|  | TCF7L2 | transcription factor 7-like 2 (T-cell specific, HMG-box) |
|  | TCF7L2 | transcription factor 7-like 2 (T-cell specific, HMG-box) |
|  | ITGA3 | integrin, alpha 3 (antigen CD49C, alpha 3 subunit of VLA-3 receptor) |
|  | ATP2A2 | ATPase, Ca++ transporting, cardiac muscle, slow twitch 2 |
|  | ITGB8 | integrin, beta 8 |
|  | DSC2 | desmocollin 2 |
|  | CACNB2 | calcium channel, voltage-dependent, beta 2 subunit |
|  | DSG2 | desmoglein 2 |
|  | ITGA2 | integrin, alpha 2 (CD49B, alpha 2 subunit of VLA-2 receptor) |
|  | ITGB1 | integrin, beta 1 (fibronectin receptor, beta polypeptide, antigen CD29 includes MDF2, MSK12) |
|  | ITGB1 | integrin, beta 1 (fibronectin receptor, beta polypeptide, antigen CD29 includes MDF2, MSK12) |
|  | ITGAV | integrin, alpha V (vitronectin receptor, alpha polypeptide, antigen CD51) |
|  | CACNB3 | calcium channel, voltage-dependent, beta 3 subunit |
|  | CACNB3 | calcium channel, voltage-dependent, beta 3 subunit |
|  | ITGB4 | integrin, beta 4 |
|  | SGCB | sarcoglycan, beta (43kDa dystrophin-associated glycoprotein) |
|  | SGCB | sarcoglycan, beta (43kDa dystrophin-associated glycoprotein) |
|  | SGCB | sarcoglycan, beta (43kDa dystrophin-associated glycoprotein) |
|  | ITGB6 | integrin, beta 6 |
| **KEGG pathway----RNA polymerase----03020** | **Gene Symbol** | **Gene Name** |
|  | POLR2C | polymerase (RNA) II (DNA directed) polypeptide C, 33kDa |
|  | POLR2I | polymerase (RNA) II (DNA directed) polypeptide I, 14.5kDa |
|  | POLR2G | polymerase (RNA) II (DNA directed) polypeptide G |
|  | POLR1D | polymerase (RNA) I polypeptide D, 16kDa |
|  | POLR2F | polymerase (RNA) II (DNA directed) polypeptide F |
|  | POLR2K | polymerase (RNA) II (DNA directed) polypeptide K, 7.0kDa |
|  | POLR2K | polymerase (RNA) II (DNA directed) polypeptide K, 7.0kDa |
|  | POLR2E | polymerase (RNA) II (DNA directed) polypeptide E, 25kDa |
|  | POLR2D | polymerase (RNA) II (DNA directed) polypeptide D |
|  | POLR1B | polymerase (RNA) I polypeptide B, 128kDa |
|  | POLR1C | polymerase (RNA) I polypeptide C, 30kDa |
|  | POLR1C | polymerase (RNA) I polypeptide C, 30kDa |
|  | POLR3C | polymerase (RNA) III (DNA directed) polypeptide C (62kD) |
|  | ZNRD1 | zinc ribbon domain containing 1 |
|  | ZNRD1 | zinc ribbon domain containing 1 |
|  | POLR3K | polymerase (RNA) III (DNA directed) polypeptide K, 12.3 kDa |
|  | POLR3F | polymerase (RNA) III (DNA directed) polypeptide F, 39 kDa |
| **KEGG pathway----Thyroid cancer----05216** | **Gene Symbol** | **Gene Name** |
|  | CCND1 | cyclin D1 |
|  | CCND1 | cyclin D1 |
|  | TPR | translocated promoter region (to activated MET oncogene) |
|  | TPR | translocated promoter region (to activated MET oncogene) |
|  | BRAF | v-raf murine sarcoma viral oncogene homolog B1 |
|  | NRAS | neuroblastoma RAS viral (v-ras) oncogene homolog |
|  | TPM3 | tropomyosin 3 |
|  | CDH1 | cadherin 1, type 1, E-cadherin (epithelial) |
|  | PPARG | peroxisome proliferator-activated receptor gamma |
|  | MYC | v-myc myelocytomatosis viral oncogene homolog (avian) |
|  | RXRA | retinoid X receptor, alpha |
|  | MAP2K1 | mitogen-activated protein kinase kinase 1 |
|  | TFG | TRK-fused gene |
|  | MAPK1 | mitogen-activated protein kinase 1 |
|  | MAPK1 | mitogen-activated protein kinase 1 |
|  | MAP2K2 | mitogen-activated protein kinase kinase 2 |
|  | TCF7L2 | transcription factor 7-like 2 (T-cell specific, HMG-box) |
|  | TCF7L2 | transcription factor 7-like 2 (T-cell specific, HMG-box) |
|  | TCF7L2 | transcription factor 7-like 2 (T-cell specific, HMG-box) |
|  | TCF7L2 | transcription factor 7-like 2 (T-cell specific, HMG-box) |
|  | TCF7L2 | transcription factor 7-like 2 (T-cell specific, HMG-box) |
|  | TCF7L2 | transcription factor 7-like 2 (T-cell specific, HMG-box) |
| **KEGG pathway----Non-small cell lung cancer----05223** | **Gene Symbol** | **Gene Name** |
|  | PDPK1 | 3-phosphoinositide dependent protein kinase-1 |
|  | BRAF | v-raf murine sarcoma viral oncogene homolog B1 |
|  | NRAS | neuroblastoma RAS viral (v-ras) oncogene homolog |
|  | SOS1 | son of sevenless homolog 1 (Drosophila) |
|  | CDK4 | cyclin-dependent kinase 4 |
|  | RXRA | retinoid X receptor, alpha |
|  | MAP2K1 | mitogen-activated protein kinase kinase 1 |
|  | E2F3 | E2F transcription factor 3 |
|  | PRKCA | protein kinase C, alpha |
|  | PIK3CB | phosphoinositide-3-kinase, catalytic, beta polypeptide |
|  | MAP2K2 | mitogen-activated protein kinase kinase 2 |
|  | EGFR | epidermal growth factor receptor (erythroblastic leukemia viral (v-erb-b) oncogene homolog, avian) |
|  | EGFR | epidermal growth factor receptor (erythroblastic leukemia viral (v-erb-b) oncogene homolog, avian) |
|  | EGFR | epidermal growth factor receptor (erythroblastic leukemia viral (v-erb-b) oncogene homolog, avian) |
|  | GRB2 | growth factor receptor-bound protein 2 |
|  | CCND1 | cyclin D1 |
|  | CCND1 | cyclin D1 |
|  | AKT3 | v-akt murine thymoma viral oncogene homolog 3 (protein kinase B, gamma) |
|  | AKT3 | v-akt murine thymoma viral oncogene homolog 3 (protein kinase B, gamma) |
|  | AKT3 | v-akt murine thymoma viral oncogene homolog 3 (protein kinase B, gamma) |
|  | AKT3 | v-akt murine thymoma viral oncogene homolog 3 (protein kinase B, gamma) |
|  | PIK3CA | phosphoinositide-3-kinase, catalytic, alpha polypeptide |
|  | MAPK1 | mitogen-activated protein kinase 1 |
|  | MAPK1 | mitogen-activated protein kinase 1 |
|  | CDK6 | cyclin-dependent kinase 6 |
|  | CDK6 | cyclin-dependent kinase 6 |
|  | CDKN2A | cyclin-dependent kinase inhibitor 2A (melanoma, p16, inhibits CDK4) |
|  | STK4 | serine/threonine kinase 4 |
| **KEGG pathway----Epithelial cell signaling in Helicobacter pylori infection----05120** | **Gene Symbol** | **Gene Name** |
|  | PAK1 | p21 protein (Cdc42/Rac)-activated kinase 1 |
|  | PAK1 | p21 protein (Cdc42/Rac)-activated kinase 1 |
|  | MAPK8 | mitogen-activated protein kinase 8 |
|  | ADAM17 | ADAM metallopeptidase domain 17 |
|  | ADAM17 | ADAM metallopeptidase domain 17 |
|  | ATP6V1A | ATPase, H+ transporting, lysosomal 70kDa, V1 subunit A |
|  | ATP6V1A | ATPase, H+ transporting, lysosomal 70kDa, V1 subunit A |
|  | ADAM10 | ADAM metallopeptidase domain 10 |
|  | ADAM10 | ADAM metallopeptidase domain 10 |
|  | CCL5 | chemokine (C-C motif) ligand 5 |
|  | CCL5 | chemokine (C-C motif) ligand 5 |
|  | ATP6V1B2 | ATPase, H+ transporting, lysosomal 56/58kDa, V1 subunit B2 |
|  | SRC | v-src sarcoma (Schmidt-Ruppin A-2) viral oncogene homolog (avian) |
|  | EGFR | epidermal growth factor receptor (erythroblastic leukemia viral (v-erb-b) oncogene homolog, avian) |
|  | EGFR | epidermal growth factor receptor (erythroblastic leukemia viral (v-erb-b) oncogene homolog, avian) |
|  | EGFR | epidermal growth factor receptor (erythroblastic leukemia viral (v-erb-b) oncogene homolog, avian) |
|  | JUN | jun oncogene |
|  | JUN | jun oncogene |
|  | JUN | jun oncogene |
|  | ATP6V0B | ATPase, H+ transporting, lysosomal 21kDa, V0 subunit b |
|  | TCIRG1 | T-cell, immune regulator 1, ATPase, H+ transporting, lysosomal V0 subunit A3 |
|  | NFKBIA | nuclear factor of kappa light polypeptide gene enhancer in B-cells inhibitor, alpha |
|  | IL8 | interleukin 8 |
|  | MAPK9 | mitogen-activated protein kinase 9 |
|  | IKBKB | inhibitor of kappa light polypeptide gene enhancer in B-cells, kinase beta |
|  | IKBKB | inhibitor of kappa light polypeptide gene enhancer in B-cells, kinase beta |
|  | CXCL1 | chemokine (C-X-C motif) ligand 1 (melanoma growth stimulating activity, alpha) |
|  | ATP6V1C1 | ATPase, H+ transporting, lysosomal 42kDa, V1 subunit C1 |
|  | ATP6V1C1 | ATPase, H+ transporting, lysosomal 42kDa, V1 subunit C1 |
|  | MAPK10 | mitogen-activated protein kinase 10 |
|  | ATP6V1E1 | ATPase, H+ transporting, lysosomal 31kDa, V1 subunit E1 |
|  | ATP6V0E1 | ATPase, H+ transporting, lysosomal 9kDa, V0 subunit e1 |
|  | ATP6V0E1 | ATPase, H+ transporting, lysosomal 9kDa, V0 subunit e1 |
|  | MAP2K4 | mitogen-activated protein kinase kinase 4 |
|  | ATP6V1G1 | ATPase, H+ transporting, lysosomal 13kDa, V1 subunit G1 |
| **KEGG pathway----Chemokine signaling pathway----04062** | **Gene Symbol** | **Gene Name** |
|  | GNG12 | guanine nucleotide binding protein (G protein), gamma 12 |
|  | GNG12 | guanine nucleotide binding protein (G protein), gamma 12 |
|  | GNG12 | guanine nucleotide binding protein (G protein), gamma 12 |
|  | BRAF | v-raf murine sarcoma viral oncogene homolog B1 |
|  | GRK5 | G protein-coupled receptor kinase 5 |
|  | GNG11 | guanine nucleotide binding protein (G protein), gamma 11 |
|  | CCL2 | chemokine (C-C motif) ligand 2 |
|  | MAP2K1 | mitogen-activated protein kinase kinase 1 |
|  | GNB1 | guanine nucleotide binding protein (G protein), beta polypeptide 1 |
|  | CXCL2 | chemokine (C-X-C motif) ligand 2 |
|  | CCL5 | chemokine (C-C motif) ligand 5 |
|  | CCL5 | chemokine (C-C motif) ligand 5 |
|  | SHC2 | SHC (Src homology 2 domain containing) transforming protein 2 |
|  | CRKL | v-crk sarcoma virus CT10 oncogene homolog (avian)-like |
|  | CXCL9 | chemokine (C-X-C motif) ligand 9 |
|  | CXCL16 | chemokine (C-X-C motif) ligand 16 |
|  | RAC2 | ras-related C3 botulinum toxin substrate 2 (rho family, small GTP binding protein Rac2) |
|  | RAP1A | RAP1A, member of RAS oncogene family |
|  | SHC1 | SHC (Src homology 2 domain containing) transforming protein 1 |
|  | IKBKB | inhibitor of kappa light polypeptide gene enhancer in B-cells, kinase beta |
|  | IKBKB | inhibitor of kappa light polypeptide gene enhancer in B-cells, kinase beta |
|  | PIK3CA | phosphoinositide-3-kinase, catalytic, alpha polypeptide |
|  | ADCY3 | adenylate cyclase 3 |
|  | TIAM1 | T-cell lymphoma invasion and metastasis 1 |
|  | ROCK2 | Rho-associated, coiled-coil containing protein kinase 2 |
|  | PRKACB | protein kinase, cAMP-dependent, catalytic, beta |
|  | ADCY7 | adenylate cyclase 7 |
|  | PAK1 | p21 protein (Cdc42/Rac)-activated kinase 1 |
|  | PAK1 | p21 protein (Cdc42/Rac)-activated kinase 1 |
|  | VAV3 | vav 3 guanine nucleotide exchange factor |
|  | NRAS | neuroblastoma RAS viral (v-ras) oncogene homolog |
|  | RHOA | ras homolog gene family, member A |
|  | RHOA | ras homolog gene family, member A |
|  | CXCL13 | chemokine (C-X-C motif) ligand 13 |
|  | PXN | paxillin |
|  | SOS1 | son of sevenless homolog 1 (Drosophila) |
|  | PTK2 | PTK2 protein tyrosine kinase 2 |
|  | PTK2 | PTK2 protein tyrosine kinase 2 |
|  | GNAI3 | guanine nucleotide binding protein (G protein), alpha inhibiting activity polypeptide 3 |
|  | GNAI3 | guanine nucleotide binding protein (G protein), alpha inhibiting activity polypeptide 3 |
|  | GNAI3 | guanine nucleotide binding protein (G protein), alpha inhibiting activity polypeptide 3 |
|  | HCK | hemopoietic cell kinase |
|  | PIK3CB | phosphoinositide-3-kinase, catalytic, beta polypeptide |
|  | GRB2 | growth factor receptor-bound protein 2 |
|  | CXCL10 | chemokine (C-X-C motif) ligand 10 |
|  | CXCR4 | chemokine (C-X-C motif) receptor 4 |
|  | CXCR4 | chemokine (C-X-C motif) receptor 4 |
|  | AKT3 | v-akt murine thymoma viral oncogene homolog 3 (protein kinase B, gamma) |
|  | AKT3 | v-akt murine thymoma viral oncogene homolog 3 (protein kinase B, gamma) |
|  | AKT3 | v-akt murine thymoma viral oncogene homolog 3 (protein kinase B, gamma) |
|  | AKT3 | v-akt murine thymoma viral oncogene homolog 3 (protein kinase B, gamma) |
|  | GNB4 | guanine nucleotide binding protein (G protein), beta polypeptide 4 |
|  | NFKBIA | nuclear factor of kappa light polypeptide gene enhancer in B-cells inhibitor, alpha |
|  | IL8 | interleukin 8 |
|  | PRKCZ | protein kinase C, zeta |
|  | CXCL1 | chemokine (C-X-C motif) ligand 1 (melanoma growth stimulating activity, alpha) |
|  | ROCK1 | Rho-associated, coiled-coil containing protein kinase 1 |
|  | ROCK1 | Rho-associated, coiled-coil containing protein kinase 1 |
|  | MAPK1 | mitogen-activated protein kinase 1 |
|  | MAPK1 | mitogen-activated protein kinase 1 |
|  | CCL18 | chemokine (C-C motif) ligand 18 (pulmonary and activation-regulated) |
|  | CCL18 | chemokine (C-C motif) ligand 18 (pulmonary and activation-regulated) |
| **KEGG pathway----SNARE interactions in vesicular transport----04130** | **Gene Symbol** | **Gene Name** |
|  | VAMP4 | vesicle-associated membrane protein 4 |
|  | VAMP7 | vesicle-associated membrane protein 7 |
|  | GOSR2 | golgi SNAP receptor complex member 2 |
|  | GOSR2 | golgi SNAP receptor complex member 2 |
|  | STX8 | syntaxin 8 |
|  | VAMP3 | vesicle-associated membrane protein 3 (cellubrevin) |
|  | VAMP3 | vesicle-associated membrane protein 3 (cellubrevin) |
|  | VTI1A | vesicle transport through interaction with t-SNAREs homolog 1A (yeast) |
|  | YKT6 | YKT6 v-SNARE homolog (S. cerevisiae) |
|  | YKT6 | YKT6 v-SNARE homolog (S. cerevisiae) |
|  | BET1 | blocked early in transport 1 homolog (S. cerevisiae) |
|  | VAMP2 | vesicle-associated membrane protein 2 (synaptobrevin 2) |
|  | GOSR1 | golgi SNAP receptor complex member 1 |
|  | STX16 | syntaxin 16 |
|  | STX16 | syntaxin 16 |
|  | STX6 | syntaxin 6 |
|  | STX6 | syntaxin 6 |
|  | SNAP23 | synaptosomal-associated protein, 23kDa |
|  | SNAP29 | synaptosomal-associated protein, 29kDa |
|  | SEC22B | SEC22 vesicle trafficking protein homolog B (S. cerevisiae) |
|  | SEC22B | SEC22 vesicle trafficking protein homolog B (S. cerevisiae) |
|  | SEC22B | SEC22 vesicle trafficking protein homolog B (S. cerevisiae) |
|  | STX5 | syntaxin 5 |
| **KEGG pathway----Acute myeloid leukemia----05221** | **Gene Symbol** | **Gene Name** |
|  | BRAF | v-raf murine sarcoma viral oncogene homolog B1 |
|  | NRAS | neuroblastoma RAS viral (v-ras) oncogene homolog |
|  | JUP | junction plakoglobin |
|  | SOS1 | son of sevenless homolog 1 (Drosophila) |
|  | MAP2K1 | mitogen-activated protein kinase kinase 1 |
|  | CEBPA | CCAAT/enhancer binding protein (C/EBP), alpha |
|  | PIK3CB | phosphoinositide-3-kinase, catalytic, beta polypeptide |
|  | MAP2K2 | mitogen-activated protein kinase kinase 2 |
|  | TCF7L2 | transcription factor 7-like 2 (T-cell specific, HMG-box) |
|  | TCF7L2 | transcription factor 7-like 2 (T-cell specific, HMG-box) |
|  | TCF7L2 | transcription factor 7-like 2 (T-cell specific, HMG-box) |
|  | TCF7L2 | transcription factor 7-like 2 (T-cell specific, HMG-box) |
|  | TCF7L2 | transcription factor 7-like 2 (T-cell specific, HMG-box) |
|  | TCF7L2 | transcription factor 7-like 2 (T-cell specific, HMG-box) |
|  | GRB2 | growth factor receptor-bound protein 2 |
|  | CCND1 | cyclin D1 |
|  | CCND1 | cyclin D1 |
|  | MYC | v-myc myelocytomatosis viral oncogene homolog (avian) |
|  | EIF4EBP1 | eukaryotic translation initiation factor 4E binding protein 1 |
|  | AKT3 | v-akt murine thymoma viral oncogene homolog 3 (protein kinase B, gamma) |
|  | AKT3 | v-akt murine thymoma viral oncogene homolog 3 (protein kinase B, gamma) |
|  | AKT3 | v-akt murine thymoma viral oncogene homolog 3 (protein kinase B, gamma) |
|  | AKT3 | v-akt murine thymoma viral oncogene homolog 3 (protein kinase B, gamma) |
|  | IKBKB | inhibitor of kappa light polypeptide gene enhancer in B-cells, kinase beta |
|  | IKBKB | inhibitor of kappa light polypeptide gene enhancer in B-cells, kinase beta |
|  | PML | promyelocytic leukemia |
|  | PML | promyelocytic leukemia |
|  | PML | promyelocytic leukemia |
|  | PIK3CA | phosphoinositide-3-kinase, catalytic, alpha polypeptide |
|  | RPS6KB1 | ribosomal protein S6 kinase, 70kDa, polypeptide 1 |
|  | PIM1 | pim-1 oncogene |
|  | RUNX1 | runt-related transcription factor 1 |
|  | MAPK1 | mitogen-activated protein kinase 1 |
|  | MAPK1 | mitogen-activated protein kinase 1 |
| **KEGG pathway----Long-term potentiation----04720** | **Gene Symbol** | **Gene Name** |
|  | PPP3CA | protein phosphatase 3 (formerly 2B), catalytic subunit, alpha isoform |
|  | PPP3CA | protein phosphatase 3 (formerly 2B), catalytic subunit, alpha isoform |
|  | BRAF | v-raf murine sarcoma viral oncogene homolog B1 |
|  | NRAS | neuroblastoma RAS viral (v-ras) oncogene homolog |
|  | PPP1R12A | protein phosphatase 1, regulatory (inhibitor) subunit 12A |
|  | CALM1 | calmodulin 1 (phosphorylase kinase, delta) |
|  | MAP2K1 | mitogen-activated protein kinase kinase 1 |
|  | PPP3CB | protein phosphatase 3 (formerly 2B), catalytic subunit, beta isoform |
|  | PRKCA | protein kinase C, alpha |
|  | ITPR3 | inositol 1,4,5-triphosphate receptor, type 3 |
|  | PPP1CC | protein phosphatase 1, catalytic subunit, gamma isoform |
|  | MAP2K2 | mitogen-activated protein kinase kinase 2 |
|  | CALM2 | calmodulin 2 (phosphorylase kinase, delta) |
|  | CAMK2D | calcium/calmodulin-dependent protein kinase II delta |
|  | RAP1A | RAP1A, member of RAS oncogene family |
|  | ITPR1 | inositol 1,4,5-triphosphate receptor, type 1 |
|  | PPP3R1 | protein phosphatase 3 (formerly 2B), regulatory subunit B, alpha isoform |
|  | PPP1CB | protein phosphatase 1, catalytic subunit, beta isoform |
|  | PPP1CB | protein phosphatase 1, catalytic subunit, beta isoform |
|  | GNAQ | guanine nucleotide binding protein (G protein), q polypeptide |
|  | RAPGEF3 | Rap guanine nucleotide exchange factor (GEF) 3 |
|  | MAPK1 | mitogen-activated protein kinase 1 |
|  | MAPK1 | mitogen-activated protein kinase 1 |
|  | PRKACB | protein kinase, cAMP-dependent, catalytic, beta |
|  | CAMK2G | calcium/calmodulin-dependent protein kinase II gamma |
|  | RPS6KA3 | ribosomal protein S6 kinase, 90kDa, polypeptide 3 |
|  | RPS6KA3 | ribosomal protein S6 kinase, 90kDa, polypeptide 3 |
| **KEGG pathway----Valine, leucine and isoleucine biosynthesis----00290** | **Gene Symbol** | **Gene Name** |
|  | PDHA1 | pyruvate dehydrogenase (lipoamide) alpha 1 |
|  | LARS | leucyl-tRNA synthetase |
|  | IARS | isoleucyl-tRNA synthetase |
|  | LARS2 | leucyl-tRNA synthetase 2, mitochondrial |
|  | IARS2 | isoleucyl-tRNA synthetase 2, mitochondrial |
|  | BCAT1 | branched chain aminotransferase 1, cytosolic |
|  | BCAT1 | branched chain aminotransferase 1, cytosolic |
|  | PDHB | pyruvate dehydrogenase (lipoamide) beta |
|  | BCAT2 | branched chain aminotransferase 2, mitochondrial |
| **KEGG pathway----Basal transcription factors----03022** | **Gene Symbol** | **Gene Name** |
|  | Gene Symbol | Gene Name |
|  | TAF5 | TAF5 RNA polymerase II, TATA box binding protein (TBP)-associated factor, 100kDa |
|  | GTF2E2 | general transcription factor IIE, polypeptide 2, beta 34kDa |
|  | GTF2A1 | general transcription factor IIA, 1, 19/37kDa |
|  | TAF4 | TAF4 RNA polymerase II, TATA box binding protein (TBP)-associated factor, 135kDa |
|  | TBPL1 | TBP-like 1 |
|  | GTF2F2 | general transcription factor IIF, polypeptide 2, 30kDa |
|  | TAF7 | TAF7 RNA polymerase II, TATA box binding protein (TBP)-associated factor, 55kDa |
|  | TAF11 | TAF11 RNA polymerase II, TATA box binding protein (TBP)-associated factor, 28kDa |
|  | TAF11 | TAF11 RNA polymerase II, TATA box binding protein (TBP)-associated factor, 28kDa |
|  | TAF12 | TAF12 RNA polymerase II, TATA box binding protein (TBP)-associated factor, 20kDa |
|  | GTF2E1 | general transcription factor IIE, polypeptide 1, alpha 56kDa |
|  | TAF9 | TAF9 RNA polymerase II, TATA box binding protein (TBP)-associated factor, 32kDa |
|  | GTF2A2 | general transcription factor IIA, 2, 12kDa |
|  | GTF2B | general transcription factor IIB |
|  | GTF2H2 | general transcription factor IIH, polypeptide 2, 44kDa |
|  | GTF2F1 | general transcription factor IIF, polypeptide 1, 74kDa |
|  | GTF2F1 | general transcription factor IIF, polypeptide 1, 74kDa |
| **KEGG pathway----Aminoacyl-tRNA biosynthesis----00970** | **Gene Symbol** | **Gene Name** |
|  | CARS | cysteinyl-tRNA synthetase |
|  | CARS | cysteinyl-tRNA synthetase |
|  | NARS2 | asparaginyl-tRNA synthetase 2, mitochondrial (putative) |
|  | EPRS | glutamyl-prolyl-tRNA synthetase |
|  | EPRS | glutamyl-prolyl-tRNA synthetase |
|  | SARS | seryl-tRNA synthetase |
|  | LARS | leucyl-tRNA synthetase |
|  | GARS | glycyl-tRNA synthetase |
|  | WARS2 | tryptophanyl tRNA synthetase 2, mitochondrial |
|  | IARS | isoleucyl-tRNA synthetase |
|  | LARS2 | leucyl-tRNA synthetase 2, mitochondrial |
|  | RARS2 | arginyl-tRNA synthetase 2, mitochondrial |
|  | DARS | aspartyl-tRNA synthetase |
|  | IARS2 | isoleucyl-tRNA synthetase 2, mitochondrial |
|  | FARS2 | phenylalanyl-tRNA synthetase 2, mitochondrial |
|  | FARS2 | phenylalanyl-tRNA synthetase 2, mitochondrial |
|  | YARS | tyrosyl-tRNA synthetase |
|  | TARS | threonyl-tRNA synthetase |
|  | WARS | tryptophanyl-tRNA synthetase |
|  | WARS | tryptophanyl-tRNA synthetase |
| **KEGG pathway----Natural killer cell mediated cytotoxicity----04650** | **Gene Symbol** | **Gene Name** |
|  | IFNAR2 | interferon (alpha, beta and omega) receptor 2 |
|  | BRAF | v-raf murine sarcoma viral oncogene homolog B1 |
|  | FCER1G | Fc fragment of IgE, high affinity I, receptor for; gamma polypeptide |
|  | MAP2K1 | mitogen-activated protein kinase kinase 1 |
|  | PPP3CB | protein phosphatase 3 (formerly 2B), catalytic subunit, beta isoform |
|  | LCP2 | lymphocyte cytosolic protein 2 (SH2 domain containing leukocyte protein of 76kDa) |
|  | PRKCA | protein kinase C, alpha |
|  | NFAT5 | nuclear factor of activated T-cells 5, tonicity-responsive |
|  | NFAT5 | nuclear factor of activated T-cells 5, tonicity-responsive |
|  | ICAM1 | intercellular adhesion molecule 1 |
|  | SHC2 | SHC (Src homology 2 domain containing) transforming protein 2 |
|  | RAC2 | ras-related C3 botulinum toxin substrate 2 (rho family, small GTP binding protein Rac2) |
|  | NFATC3 | nuclear factor of activated T-cells, cytoplasmic, calcineurin-dependent 3 |
|  | SHC1 | SHC (Src homology 2 domain containing) transforming protein 1 |
|  | PIK3CA | phosphoinositide-3-kinase, catalytic, alpha polypeptide |
|  | IFNGR1 | interferon gamma receptor 1 |
|  | SYK | spleen tyrosine kinase |
|  | SYK | spleen tyrosine kinase |
|  | PAK1 | p21 protein (Cdc42/Rac)-activated kinase 1 |
|  | PAK1 | p21 protein (Cdc42/Rac)-activated kinase 1 |
|  | VAV3 | vav 3 guanine nucleotide exchange factor |
|  | PPP3CA | protein phosphatase 3 (formerly 2B), catalytic subunit, alpha isoform |
|  | PPP3CA | protein phosphatase 3 (formerly 2B), catalytic subunit, alpha isoform |
|  | NRAS | neuroblastoma RAS viral (v-ras) oncogene homolog |
|  | SOS1 | son of sevenless homolog 1 (Drosophila) |
|  | PTPN6 | protein tyrosine phosphatase, non-receptor type 6 |
|  | MICB | MHC class I polypeptide-related sequence B |
|  | TNFRSF10B | tumor necrosis factor receptor superfamily, member 10b |
|  | TNFSF10 | tumor necrosis factor (ligand) superfamily, member 10 |
|  | ICAM2 | intercellular adhesion molecule 2 |
|  | MAP2K2 | mitogen-activated protein kinase kinase 2 |
|  | PIK3CB | phosphoinositide-3-kinase, catalytic, beta polypeptide |
|  | FAS | Fas (TNF receptor superfamily, member 6) |
|  | FAS | Fas (TNF receptor superfamily, member 6) |
|  | FAS | Fas (TNF receptor superfamily, member 6) |
|  | FAS | Fas (TNF receptor superfamily, member 6) |
|  | GRB2 | growth factor receptor-bound protein 2 |
|  | ITGB2 | integrin, beta 2 (complement component 3 receptor 3 and 4 subunit) |
|  | FYN | FYN oncogene related to SRC, FGR, YES |
|  | PPP3R1 | protein phosphatase 3 (formerly 2B), regulatory subunit B, alpha isoform |
|  | TYROBP | TYRO protein tyrosine kinase binding protein |
|  | MAPK1 | mitogen-activated protein kinase 1 |
|  | MAPK1 | mitogen-activated protein kinase 1 |
| **KEGG pathway----Cardiac muscle contraction----04260** | **Gene Symbol** | **Gene Name** |
|  | COX7C | cytochrome c oxidase subunit VIIc |
|  | ACTC1 | actin, alpha, cardiac muscle 1 |
|  | TPM3 | tropomyosin 3 |
|  | TPM2 | tropomyosin 2 (beta) |
|  | UQCRB | ubiquinol-cytochrome c reductase binding protein |
|  | UCRC | ubiquinol-cytochrome c reductase complex (7.2 kD) |
|  | ATP1A4 | ATPase, Na+/K+ transporting, alpha 4 polypeptide |
|  | TPM4 | tropomyosin 4 |
|  | TPM4 | tropomyosin 4 |
|  | COX4I1 | cytochrome c oxidase subunit IV isoform 1 |
|  | ATP1B1 | ATPase, Na+/K+ transporting, beta 1 polypeptide |
|  | ATP1B1 | ATPase, Na+/K+ transporting, beta 1 polypeptide |
|  | SLC8A1 | solute carrier family 8 (sodium/calcium exchanger), member 1 |
|  | UQCRFS1 | ubiquinol-cytochrome c reductase, Rieske iron-sulfur polypeptide 1 |
|  | ATP2A2 | ATPase, Ca++ transporting, cardiac muscle, slow twitch 2 |
|  | CACNB2 | calcium channel, voltage-dependent, beta 2 subunit |
|  | SLC9A6 | solute carrier family 9 (sodium/hydrogen exchanger), member 6 |
|  | COX7A2L | cytochrome c oxidase subunit VIIa polypeptide 2 like |
|  | UQCRC2 | ubiquinol-cytochrome c reductase core protein II |
|  | SLC9A1 | solute carrier family 9 (sodium/hydrogen exchanger), member 1 |
|  | UQCRH | ubiquinol-cytochrome c reductase hinge protein |
|  | ATP1B3 | ATPase, Na+/K+ transporting, beta 3 polypeptide |
|  | CYC1 | cytochrome c-1 |
|  | COX7B | cytochrome c oxidase subunit VIIb |
|  | CACNB3 | calcium channel, voltage-dependent, beta 3 subunit |
|  | CACNB3 | calcium channel, voltage-dependent, beta 3 subunit |
|  | TPM1 | tropomyosin 1 (alpha) |
|  | TPM1 | tropomyosin 1 (alpha) |
| **KEGG pathway----beta-Alanine metabolism----00410** | **Gene Symbol** | **Gene Name** |
|  | AOC3 | amine oxidase, copper containing 3 (vascular adhesion protein 1) |
|  | ALDH2 | aldehyde dehydrogenase 2 family (mitochondrial) |
|  | EHHADH | enoyl-Coenzyme A, hydratase/3-hydroxyacyl Coenzyme A dehydrogenase |
|  | HADHA | hydroxyacyl-Coenzyme A dehydrogenase/3-ketoacyl-Coenzyme A thiolase/enoyl-Coenzyme A hydratase (trifunctional protein), alpha subunit |
|  | ALDH7A1 | aldehyde dehydrogenase 7 family, member A1 |
|  | ALDH7A1 | aldehyde dehydrogenase 7 family, member A1 |
|  | HIBCH | 3-hydroxyisobutyryl-Coenzyme A hydrolase |
|  | ABAT | 4-aminobutyrate aminotransferase |
|  | ACADM | acyl-Coenzyme A dehydrogenase, C-4 to C-12 straight chain |
|  | DPYD | dihydropyrimidine dehydrogenase |
|  | ALDH3A2 | aldehyde dehydrogenase 3 family, member A2 |
|  | SMS | spermine synthase |
| **KEGG pathway----Drug metabolism - other enzymes----00983** | **Gene Symbol** | **Gene Name** |
|  | UGT1A1 | UDP glucuronosyltransferase 1 family, polypeptide A1 |
|  | TK1 | thymidine kinase 1, soluble |
|  | TK1 | thymidine kinase 1, soluble |
|  | CYP3A7 | cytochrome P450, family 3, subfamily A, polypeptide 7 |
|  | UCK2 | uridine-cytidine kinase 2 |
|  | CYP3A43 | cytochrome P450, family 3, subfamily A, polypeptide 43 |
|  | CYP3A4 | cytochrome P450, family 3, subfamily A, polypeptide 4 |
|  | TK2 | thymidine kinase 2, mitochondrial |
|  | UGT1A3 | UDP glucuronosyltransferase 1 family, polypeptide A3 |
|  | HPRT1 | hypoxanthine phosphoribosyltransferase 1 |
|  | TYMP | thymidine phosphorylase |
|  | CES2 | carboxylesterase 2 (intestine, liver) |
|  | CES2 | carboxylesterase 2 (intestine, liver) |
|  | UGT1A9 | UDP glucuronosyltransferase 1 family, polypeptide A9 |
|  | NAT1 | N-acetyltransferase 1 (arylamine N-acetyltransferase) |
|  | CYP3A5 | cytochrome P450, family 3, subfamily A, polypeptide 5 |
|  | UGT1A6 | UDP glucuronosyltransferase 1 family, polypeptide A6 |
|  | GMPS | guanine monphosphate synthetase |
|  | UMPS | uridine monophosphate synthetase |
|  | UMPS | uridine monophosphate synthetase |
|  | DPYD | dihydropyrimidine dehydrogenase |
| **KEGG pathway----Long-term depression----04730** | **Gene Symbol** | **Gene Name** |
|  | BRAF | v-raf murine sarcoma viral oncogene homolog B1 |
|  | NRAS | neuroblastoma RAS viral (v-ras) oncogene homolog |
|  | JMJD7-PLA2G4B | JMJD7-PLA2G4B readthrough |
|  | MAP2K1 | mitogen-activated protein kinase kinase 1 |
|  | PPP2CB | protein phosphatase 2 (formerly 2A), catalytic subunit, beta isoform |
|  | PLA2G12A | phospholipase A2, group XIIA |
|  | PRKCA | protein kinase C, alpha |
|  | ITPR3 | inositol 1,4,5-triphosphate receptor, type 3 |
|  | GNAI3 | guanine nucleotide binding protein (G protein), alpha inhibiting activity polypeptide 3 |
|  | GNAI3 | guanine nucleotide binding protein (G protein), alpha inhibiting activity polypeptide 3 |
|  | GNAI3 | guanine nucleotide binding protein (G protein), alpha inhibiting activity polypeptide 3 |
|  | MAP2K2 | mitogen-activated protein kinase kinase 2 |
|  | PPP2R1B | protein phosphatase 2 (formerly 2A), regulatory subunit A, beta isoform |
|  | PPP2R1B | protein phosphatase 2 (formerly 2A), regulatory subunit A, beta isoform |
|  | PPP2R1B | protein phosphatase 2 (formerly 2A), regulatory subunit A, beta isoform |
|  | PLA2G2A | phospholipase A2, group IIA (platelets, synovial fluid) |
|  | GNA12 | guanine nucleotide binding protein (G protein) alpha 12 |
|  | GUCY1B3 | guanylate cyclase 1, soluble, beta 3 |
|  | GNAO1 | guanine nucleotide binding protein (G protein), alpha activating activity polypeptide O |
|  | PLA2G1B | phospholipase A2, group IB (pancreas) |
|  | ITPR1 | inositol 1,4,5-triphosphate receptor, type 1 |
|  | GNAQ | guanine nucleotide binding protein (G protein), q polypeptide |
|  | GUCY1A3 | guanylate cyclase 1, soluble, alpha 3 |
|  | MAPK1 | mitogen-activated protein kinase 1 |
|  | MAPK1 | mitogen-activated protein kinase 1 |
|  | GNA13 | guanine nucleotide binding protein (G protein), alpha 13 |
|  | PLA2G6 | phospholipase A2, group VI (cytosolic, calcium-independent) |
| **KEGG pathway----Hypertrophic cardiomyopathy (HCM)----05410** | **Gene Symbol** | **Gene Name** |
|  | TGFB1 | transforming growth factor, beta 1 |
|  | PRKAB1 | protein kinase, AMP-activated, beta 1 non-catalytic subunit |
|  | ACTC1 | actin, alpha, cardiac muscle 1 |
|  | TPM3 | tropomyosin 3 |
|  | TPM2 | tropomyosin 2 (beta) |
|  | DMD | dystrophin |
|  | DES | desmin |
|  | PRKAB2 | protein kinase, AMP-activated, beta 2 non-catalytic subunit |
|  | PRKAB2 | protein kinase, AMP-activated, beta 2 non-catalytic subunit |
|  | PRKAA1 | protein kinase, AMP-activated, alpha 1 catalytic subunit |
|  | TPM4 | tropomyosin 4 |
|  | TPM4 | tropomyosin 4 |
|  | ITGA5 | integrin, alpha 5 (fibronectin receptor, alpha polypeptide) |
|  | ITGB5 | integrin, beta 5 |
|  | ITGB5 | integrin, beta 5 |
|  | SLC8A1 | solute carrier family 8 (sodium/calcium exchanger), member 1 |
|  | ITGA3 | integrin, alpha 3 (antigen CD49C, alpha 3 subunit of VLA-3 receptor) |
|  | ITGB8 | integrin, beta 8 |
|  | ATP2A2 | ATPase, Ca++ transporting, cardiac muscle, slow twitch 2 |
|  | CACNB2 | calcium channel, voltage-dependent, beta 2 subunit |
|  | ITGA2 | integrin, alpha 2 (CD49B, alpha 2 subunit of VLA-2 receptor) |
|  | ITGB1 | integrin, beta 1 (fibronectin receptor, beta polypeptide, antigen CD29 includes MDF2, MSK12) |
|  | ITGB1 | integrin, beta 1 (fibronectin receptor, beta polypeptide, antigen CD29 includes MDF2, MSK12) |
|  | ITGAV | integrin, alpha V (vitronectin receptor, alpha polypeptide, antigen CD51) |
|  | CACNB3 | calcium channel, voltage-dependent, beta 3 subunit |
|  | CACNB3 | calcium channel, voltage-dependent, beta 3 subunit |
|  | ITGB4 | integrin, beta 4 |
|  | TPM1 | tropomyosin 1 (alpha) |
|  | TPM1 | tropomyosin 1 (alpha) |
|  | ITGB6 | integrin, beta 6 |
|  | SGCB | sarcoglycan, beta (43kDa dystrophin-associated glycoprotein) |
|  | SGCB | sarcoglycan, beta (43kDa dystrophin-associated glycoprotein) |
|  | SGCB | sarcoglycan, beta (43kDa dystrophin-associated glycoprotein) |
| **KEGG pathway----Notch signaling pathway----04330** | **Gene Symbol** | **Gene Name** |
|  | NOTCH1 | Notch homolog 1, translocation-associated (Drosophila) |
|  | HES1 | hairy and enhancer of split 1, (Drosophila) |
|  | HES1 | hairy and enhancer of split 1, (Drosophila) |
|  | ADAM17 | ADAM metallopeptidase domain 17 |
|  | ADAM17 | ADAM metallopeptidase domain 17 |
|  | KAT2A | K(lysine) acetyltransferase 2A |
|  | KAT2B | K(lysine) acetyltransferase 2B |
|  | APH1A | anterior pharynx defective 1 homolog A (C. elegans) |
|  | LFNG | LFNG O-fucosylpeptide 3-beta-N-acetylglucosaminyltransferase |
|  | CTBP2 | C-terminal binding protein 2 |
|  | CTBP2 | C-terminal binding protein 2 |
|  | CTBP2 | C-terminal binding protein 2 |
|  | NOTCH2 | Notch homolog 2 (Drosophila) |
|  | NOTCH2 | Notch homolog 2 (Drosophila) |
|  | SNW1 | SNW domain containing 1 |
|  | JAG1 | jagged 1 (Alagille syndrome) |
|  | JAG1 | jagged 1 (Alagille syndrome) |
|  | JAG1 | jagged 1 (Alagille syndrome) |
|  | DTX4 | deltex homolog 4 (Drosophila) |
|  | DTX2 | deltex homolog 2 (Drosophila) |
|  | RBPJ | recombination signal binding protein for immunoglobulin kappa J region |
|  | CTBP1 | C-terminal binding protein 1 |
|  | NUMB | numb homolog (Drosophila) |
|  | NUMB | numb homolog (Drosophila) |
|  | MAML3 | mastermind-like 3 (Drosophila) |
| **KEGG pathway----Endometrial cancer----05213** | **Gene Symbol** | **Gene Name** |
|  | PDPK1 | 3-phosphoinositide dependent protein kinase-1 |
|  | BRAF | v-raf murine sarcoma viral oncogene homolog B1 |
|  | NRAS | neuroblastoma RAS viral (v-ras) oncogene homolog |
|  | SOS1 | son of sevenless homolog 1 (Drosophila) |
|  | CTNNA1 | catenin (cadherin-associated protein), alpha 1, 102kDa |
|  | MAP2K1 | mitogen-activated protein kinase kinase 1 |
|  | APC | adenomatous polyposis coli |
|  | PIK3CB | phosphoinositide-3-kinase, catalytic, beta polypeptide |
|  | MAP2K2 | mitogen-activated protein kinase kinase 2 |
|  | EGFR | epidermal growth factor receptor (erythroblastic leukemia viral (v-erb-b) oncogene homolog, avian) |
|  | EGFR | epidermal growth factor receptor (erythroblastic leukemia viral (v-erb-b) oncogene homolog, avian) |
|  | EGFR | epidermal growth factor receptor (erythroblastic leukemia viral (v-erb-b) oncogene homolog, avian) |
|  | TCF7L2 | transcription factor 7-like 2 (T-cell specific, HMG-box) |
|  | TCF7L2 | transcription factor 7-like 2 (T-cell specific, HMG-box) |
|  | TCF7L2 | transcription factor 7-like 2 (T-cell specific, HMG-box) |
|  | TCF7L2 | transcription factor 7-like 2 (T-cell specific, HMG-box) |
|  | TCF7L2 | transcription factor 7-like 2 (T-cell specific, HMG-box) |
|  | TCF7L2 | transcription factor 7-like 2 (T-cell specific, HMG-box) |
|  | GRB2 | growth factor receptor-bound protein 2 |
|  | CCND1 | cyclin D1 |
|  | CCND1 | cyclin D1 |
|  | CDH1 | cadherin 1, type 1, E-cadherin (epithelial) |
|  | MYC | v-myc myelocytomatosis viral oncogene homolog (avian) |
|  | AKT3 | v-akt murine thymoma viral oncogene homolog 3 (protein kinase B, gamma) |
|  | AKT3 | v-akt murine thymoma viral oncogene homolog 3 (protein kinase B, gamma) |
|  | AKT3 | v-akt murine thymoma viral oncogene homolog 3 (protein kinase B, gamma) |
|  | AKT3 | v-akt murine thymoma viral oncogene homolog 3 (protein kinase B, gamma) |
|  | PIK3CA | phosphoinositide-3-kinase, catalytic, alpha polypeptide |
|  | MAPK1 | mitogen-activated protein kinase 1 |
|  | MAPK1 | mitogen-activated protein kinase 1 |
| **KEGG pathway----Drug metabolism - cytochrome P450----00982** | **Gene Symbol** | **Gene Name** |
|  | UGT1A1 | UDP glucuronosyltransferase 1 family, polypeptide A1 |
|  | ADH7 | alcohol dehydrogenase 7 (class IV), mu or sigma polypeptide |
|  | GSTM2 | glutathione S-transferase mu 2 (muscle) |
|  | CYP2C9 | cytochrome P450, family 2, subfamily C, polypeptide 9 |
|  | MGST3 | microsomal glutathione S-transferase 3 |
|  | CYP3A7 | cytochrome P450, family 3, subfamily A, polypeptide 7 |
|  | CYP3A43 | cytochrome P450, family 3, subfamily A, polypeptide 43 |
|  | MGST2 | microsomal glutathione S-transferase 2 |
|  | MGST1 | microsomal glutathione S-transferase 1 |
|  | MGST1 | microsomal glutathione S-transferase 1 |
|  | MGST1 | microsomal glutathione S-transferase 1 |
|  | CYP3A4 | cytochrome P450, family 3, subfamily A, polypeptide 4 |
|  | UGT1A3 | UDP glucuronosyltransferase 1 family, polypeptide A3 |
|  | ALDH3B2 | aldehyde dehydrogenase 3 family, member B2 |
|  | ADH5 | alcohol dehydrogenase 5 (class III), chi polypeptide |
|  | ADH5 | alcohol dehydrogenase 5 (class III), chi polypeptide |
|  | GSTK1 | glutathione S-transferase kappa 1 |
|  | ALDH3B1 | aldehyde dehydrogenase 3 family, member B1 |
|  | ALDH3B1 | aldehyde dehydrogenase 3 family, member B1 |
|  | UGT1A9 | UDP glucuronosyltransferase 1 family, polypeptide A9 |
|  | ALDH1A3 | aldehyde dehydrogenase 1 family, member A3 |
|  | CYP3A5 | cytochrome P450, family 3, subfamily A, polypeptide 5 |
|  | MAOA | monoamine oxidase A |
|  | MAOA | monoamine oxidase A |
|  | MAOA | monoamine oxidase A |
|  | UGT1A6 | UDP glucuronosyltransferase 1 family, polypeptide A6 |
|  | GSTA4 | glutathione S-transferase alpha 4 |
|  | GSTM1 | glutathione S-transferase mu 1 |
| **KEGG pathway----Pentose phosphate pathway----00030** | **Gene Symbol** | **Gene Name** |
|  | PGM2 | phosphoglucomutase 2 |
|  | PGM2 | phosphoglucomutase 2 |
|  | PGM2 | phosphoglucomutase 2 |
|  | ALDOC | aldolase C, fructose-bisphosphate |
|  | FBP1 | fructose-1,6-bisphosphatase 1 |
|  | PRPS2 | phosphoribosyl pyrophosphate synthetase 2 |
|  | PRPS2 | phosphoribosyl pyrophosphate synthetase 2 |
|  | TKT | transketolase |
|  | DERA | 2-deoxyribose-5-phosphate aldolase homolog (C. elegans) |
|  | ALDOB | aldolase B, fructose-bisphosphate |
|  | PRPS1 | phosphoribosyl pyrophosphate synthetase 1 |
|  | PFKM | phosphofructokinase, muscle |
|  | PFKP | phosphofructokinase, platelet |
|  | RPIA | ribose 5-phosphate isomerase A |
|  | PGD | phosphogluconate dehydrogenase |
| **KEGG pathway----Lysine degradation----00310** | **Gene Symbol** | **Gene Name** |
|  | ALDH2 | aldehyde dehydrogenase 2 family (mitochondrial) |
|  | EHHADH | enoyl-Coenzyme A, hydratase/3-hydroxyacyl Coenzyme A dehydrogenase |
|  | HADHA | hydroxyacyl-Coenzyme A dehydrogenase/3-ketoacyl-Coenzyme A thiolase/enoyl-Coenzyme A hydratase (trifunctional protein), alpha subunit |
|  | SETMAR | SET domain and mariner transposase fusion gene |
|  | SETD2 | SET domain containing 2 |
|  | PLOD1 | procollagen-lysine 1, 2-oxoglutarate 5-dioxygenase 1 |
|  | SETD7 | SET domain containing (lysine methyltransferase) 7 |
|  | NSD1 | nuclear receptor binding SET domain protein 1 |
|  | ACAT2 | acetyl-Coenzyme A acetyltransferase 2 |
|  | ACAT2 | acetyl-Coenzyme A acetyltransferase 2 |
|  | ACAT2 | acetyl-Coenzyme A acetyltransferase 2 |
|  | AASDHPPT | aminoadipate-semialdehyde dehydrogenase-phosphopantetheinyl transferase |
|  | AASDHPPT | aminoadipate-semialdehyde dehydrogenase-phosphopantetheinyl transferase |
|  | SUV39H2 | suppressor of variegation 3-9 homolog 2 (Drosophila) |
|  | HADH | hydroxyacyl-Coenzyme A dehydrogenase |
|  | PLOD2 | procollagen-lysine, 2-oxoglutarate 5-dioxygenase 2 |
|  | ACAT1 | acetyl-Coenzyme A acetyltransferase 1 |
|  | ALDH7A1 | aldehyde dehydrogenase 7 family, member A1 |
|  | ALDH7A1 | aldehyde dehydrogenase 7 family, member A1 |
|  | ALDH3A2 | aldehyde dehydrogenase 3 family, member A2 |
| **KEGG pathway----Tryptophan metabolism----00380** | **Gene Symbol** | **Gene Name** |
|  | CAT | catalase |
|  | ALDH2 | aldehyde dehydrogenase 2 family (mitochondrial) |
|  | WARS2 | tryptophanyl tRNA synthetase 2, mitochondrial |
|  | ACAT2 | acetyl-Coenzyme A acetyltransferase 2 |
|  | ACAT2 | acetyl-Coenzyme A acetyltransferase 2 |
|  | ACAT2 | acetyl-Coenzyme A acetyltransferase 2 |
|  | HADH | hydroxyacyl-Coenzyme A dehydrogenase |
|  | EHHADH | enoyl-Coenzyme A, hydratase/3-hydroxyacyl Coenzyme A dehydrogenase |
|  | KYNU | kynureninase (L-kynurenine hydrolase) |
|  | KYNU | kynureninase (L-kynurenine hydrolase) |
|  | KYNU | kynureninase (L-kynurenine hydrolase) |
|  | ABP1 | amiloride binding protein 1 (amine oxidase (copper-containing)) |
|  | HADHA | hydroxyacyl-Coenzyme A dehydrogenase/3-ketoacyl-Coenzyme A thiolase/enoyl-Coenzyme A hydratase (trifunctional protein), alpha subunit |
|  | ACAT1 | acetyl-Coenzyme A acetyltransferase 1 |
|  | MAOA | monoamine oxidase A |
|  | MAOA | monoamine oxidase A |
|  | MAOA | monoamine oxidase A |
|  | ALDH7A1 | aldehyde dehydrogenase 7 family, member A1 |
|  | ALDH7A1 | aldehyde dehydrogenase 7 family, member A1 |
|  | ALDH3A2 | aldehyde dehydrogenase 3 family, member A2 |
|  | WARS | tryptophanyl-tRNA synthetase |
|  | WARS | tryptophanyl-tRNA synthetase |
|  | CYP1B1 | cytochrome P450, family 1, subfamily B, polypeptide 1 |
|  | CYP1B1 | cytochrome P450, family 1, subfamily B, polypeptide 1 |
| **KEGG pathway----PPAR signaling pathway----03320** | **Gene Symbol** | **Gene Name** |
|  | ACSL5 | acyl-CoA synthetase long-chain family member 5 |
|  | ACSL5 | acyl-CoA synthetase long-chain family member 5 |
|  | PDPK1 | 3-phosphoinositide dependent protein kinase-1 |
|  | SORBS1 | sorbin and SH3 domain containing 1 |
|  | SORBS1 | sorbin and SH3 domain containing 1 |
|  | ACSL1 | acyl-CoA synthetase long-chain family member 1 |
|  | ACSL1 | acyl-CoA synthetase long-chain family member 1 |
|  | EHHADH | enoyl-Coenzyme A, hydratase/3-hydroxyacyl Coenzyme A dehydrogenase |
|  | SLC27A2 | solute carrier family 27 (fatty acid transporter), member 2 |
|  | SLC27A2 | solute carrier family 27 (fatty acid transporter), member 2 |
|  | PPARG | peroxisome proliferator-activated receptor gamma |
|  | CPT2 | carnitine palmitoyltransferase 2 |
|  | SCD5 | stearoyl-CoA desaturase 5 |
|  | SCD5 | stearoyl-CoA desaturase 5 |
|  | RXRA | retinoid X receptor, alpha |
|  | MMP1 | matrix metallopeptidase 1 (interstitial collagenase) |
|  | SCP2 | sterol carrier protein 2 |
|  | SCD | stearoyl-CoA desaturase (delta-9-desaturase) |
|  | SCD | stearoyl-CoA desaturase (delta-9-desaturase) |
|  | SCD | stearoyl-CoA desaturase (delta-9-desaturase) |
|  | CD36 | CD36 molecule (thrombospondin receptor) |
|  | ACOX1 | acyl-Coenzyme A oxidase 1, palmitoyl |
|  | ACOX1 | acyl-Coenzyme A oxidase 1, palmitoyl |
|  | ACOX1 | acyl-Coenzyme A oxidase 1, palmitoyl |
|  | DBI | diazepam binding inhibitor (GABA receptor modulator, acyl-Coenzyme A binding protein) |
|  | DBI | diazepam binding inhibitor (GABA receptor modulator, acyl-Coenzyme A binding protein) |
|  | DBI | diazepam binding inhibitor (GABA receptor modulator, acyl-Coenzyme A binding protein) |
|  | ME1 | malic enzyme 1, NADP(+)-dependent, cytosolic |
|  | ACSL4 | acyl-CoA synthetase long-chain family member 4 |
|  | ACADM | acyl-Coenzyme A dehydrogenase, C-4 to C-12 straight chain |
|  | PLTP | phospholipid transfer protein |
|  | FABP1 | fatty acid binding protein 1, liver |
| **KEGG pathway----Porphyrin and chlorophyll metabolism----00860** | **Gene Symbol** | **Gene Name** |
|  | FECH | ferrochelatase (protoporphyria) |
|  | UGT1A1 | UDP glucuronosyltransferase 1 family, polypeptide A1 |
|  | EPRS | glutamyl-prolyl-tRNA synthetase |
|  | EPRS | glutamyl-prolyl-tRNA synthetase |
|  | COX15 | COX15 homolog, cytochrome c oxidase assembly protein (yeast) |
|  | UROS | uroporphyrinogen III synthase |
|  | UGT1A9 | UDP glucuronosyltransferase 1 family, polypeptide A9 |
|  | BLVRB | biliverdin reductase B (flavin reductase (NADPH)) |
|  | CPOX | coproporphyrinogen oxidase |
|  | UROD | uroporphyrinogen decarboxylase |
|  | ALAS1 | aminolevulinate, delta-, synthase 1 |
|  | UGT1A6 | UDP glucuronosyltransferase 1 family, polypeptide A6 |
|  | BLVRA | biliverdin reductase A |
|  | HCCS | holocytochrome c synthase (cytochrome c heme-lyase) |
|  | HMOX1 | heme oxygenase (decycling) 1 |
|  | UGT1A3 | UDP glucuronosyltransferase 1 family, polypeptide A3 |
| **KEGG pathway----Histidine metabolism----00340** | **Gene Symbol** | **Gene Name** |
|  | ALDH3B1 | aldehyde dehydrogenase 3 family, member B1 |
|  | ALDH3B1 | aldehyde dehydrogenase 3 family, member B1 |
|  | ALDH2 | aldehyde dehydrogenase 2 family (mitochondrial) |
|  | HNMT | histamine N-methyltransferase |
|  | ABP1 | amiloride binding protein 1 (amine oxidase (copper-containing)) |
|  | METTL6 | methyltransferase like 6 |
|  | ALDH1A3 | aldehyde dehydrogenase 1 family, member A3 |
|  | WBSCR22 | Williams Beuren syndrome chromosome region 22 |
|  | MAOA | monoamine oxidase A |
|  | MAOA | monoamine oxidase A |
|  | MAOA | monoamine oxidase A |
|  | ALDH7A1 | aldehyde dehydrogenase 7 family, member A1 |
|  | ALDH7A1 | aldehyde dehydrogenase 7 family, member A1 |
|  | METTL2B | methyltransferase like 2B |
|  | ALDH3A2 | aldehyde dehydrogenase 3 family, member A2 |
|  | ALDH3B2 | aldehyde dehydrogenase 3 family, member B2 |
| **KEGG pathway----Limonene and pinene degradation----00903** | **Gene Symbol** | **Gene Name** |
|  | ALDH2 | aldehyde dehydrogenase 2 family (mitochondrial) |
|  | EHHADH | enoyl-Coenzyme A, hydratase/3-hydroxyacyl Coenzyme A dehydrogenase |
|  | HADHA | hydroxyacyl-Coenzyme A dehydrogenase/3-ketoacyl-Coenzyme A thiolase/enoyl-Coenzyme A hydratase (trifunctional protein), alpha subunit |
|  | ALDH7A1 | aldehyde dehydrogenase 7 family, member A1 |
|  | ALDH7A1 | aldehyde dehydrogenase 7 family, member A1 |
|  | YOD1 | YOD1 OTU deubiquinating enzyme 1 homolog (S. cerevisiae) |
|  | NAT5 | N-acetyltransferase 5 (GCN5-related, putative) |
|  | LCLAT1 | lysocardiolipin acyltransferase 1 |
|  | ALDH3A2 | aldehyde dehydrogenase 3 family, member A2 |
| **KEGG pathway----Melanoma----05218** | **Gene Symbol** | **Gene Name** |
|  | PDGFC | platelet derived growth factor C |
|  | BRAF | v-raf murine sarcoma viral oncogene homolog B1 |
|  | NRAS | neuroblastoma RAS viral (v-ras) oncogene homolog |
|  | CDK4 | cyclin-dependent kinase 4 |
|  | MAP2K1 | mitogen-activated protein kinase kinase 1 |
|  | E2F3 | E2F transcription factor 3 |
|  | PDGFRA | platelet-derived growth factor receptor, alpha polypeptide |
|  | PIK3CB | phosphoinositide-3-kinase, catalytic, beta polypeptide |
|  | MAP2K2 | mitogen-activated protein kinase kinase 2 |
|  | EGFR | epidermal growth factor receptor (erythroblastic leukemia viral (v-erb-b) oncogene homolog, avian) |
|  | EGFR | epidermal growth factor receptor (erythroblastic leukemia viral (v-erb-b) oncogene homolog, avian) |
|  | EGFR | epidermal growth factor receptor (erythroblastic leukemia viral (v-erb-b) oncogene homolog, avian) |
|  | FGFR1 | fibroblast growth factor receptor 1 |
|  | CCND1 | cyclin D1 |
|  | CCND1 | cyclin D1 |
|  | MDM2 | Mdm2 p53 binding protein homolog (mouse) |
|  | CDH1 | cadherin 1, type 1, E-cadherin (epithelial) |
|  | AKT3 | v-akt murine thymoma viral oncogene homolog 3 (protein kinase B, gamma) |
|  | AKT3 | v-akt murine thymoma viral oncogene homolog 3 (protein kinase B, gamma) |
|  | AKT3 | v-akt murine thymoma viral oncogene homolog 3 (protein kinase B, gamma) |
|  | AKT3 | v-akt murine thymoma viral oncogene homolog 3 (protein kinase B, gamma) |
|  | MITF | microphthalmia-associated transcription factor |
|  | MITF | microphthalmia-associated transcription factor |
|  | PIK3CA | phosphoinositide-3-kinase, catalytic, alpha polypeptide |
|  | MAPK1 | mitogen-activated protein kinase 1 |
|  | MAPK1 | mitogen-activated protein kinase 1 |
|  | CDK6 | cyclin-dependent kinase 6 |
|  | CDK6 | cyclin-dependent kinase 6 |
|  | CDKN2A | cyclin-dependent kinase inhibitor 2A (melanoma, p16, inhibits CDK4) |
|  | PDGFRB | platelet-derived growth factor receptor, beta polypeptide |
| **KEGG pathway----Dilated cardiomyopathy----05414** | **Gene Symbol** | **Gene Name** |
|  | TGFB1 | transforming growth factor, beta 1 |
|  | ACTC1 | actin, alpha, cardiac muscle 1 |
|  | TPM3 | tropomyosin 3 |
|  | TPM2 | tropomyosin 2 (beta) |
|  | DMD | dystrophin |
|  | DES | desmin |
|  | TPM4 | tropomyosin 4 |
|  | TPM4 | tropomyosin 4 |
|  | ITGA5 | integrin, alpha 5 (fibronectin receptor, alpha polypeptide) |
|  | ITGB5 | integrin, beta 5 |
|  | ITGB5 | integrin, beta 5 |
|  | SLC8A1 | solute carrier family 8 (sodium/calcium exchanger), member 1 |
|  | ITGA3 | integrin, alpha 3 (antigen CD49C, alpha 3 subunit of VLA-3 receptor) |
|  | ITGB8 | integrin, beta 8 |
|  | ATP2A2 | ATPase, Ca++ transporting, cardiac muscle, slow twitch 2 |
|  | CACNB2 | calcium channel, voltage-dependent, beta 2 subunit |
|  | ITGA2 | integrin, alpha 2 (CD49B, alpha 2 subunit of VLA-2 receptor) |
|  | ITGB1 | integrin, beta 1 (fibronectin receptor, beta polypeptide, antigen CD29 includes MDF2, MSK12) |
|  | ITGB1 | integrin, beta 1 (fibronectin receptor, beta polypeptide, antigen CD29 includes MDF2, MSK12) |
|  | ADCY3 | adenylate cyclase 3 |
|  | ITGAV | integrin, alpha V (vitronectin receptor, alpha polypeptide, antigen CD51) |
|  | CACNB3 | calcium channel, voltage-dependent, beta 3 subunit |
|  | CACNB3 | calcium channel, voltage-dependent, beta 3 subunit |
|  | ITGB4 | integrin, beta 4 |
|  | TPM1 | tropomyosin 1 (alpha) |
|  | TPM1 | tropomyosin 1 (alpha) |
|  | ITGB6 | integrin, beta 6 |
|  | SGCB | sarcoglycan, beta (43kDa dystrophin-associated glycoprotein) |
|  | SGCB | sarcoglycan, beta (43kDa dystrophin-associated glycoprotein) |
|  | SGCB | sarcoglycan, beta (43kDa dystrophin-associated glycoprotein) |
|  | PRKACB | protein kinase, cAMP-dependent, catalytic, beta |
|  | ADCY7 | adenylate cyclase 7 |
| **KEGG pathway----Melanogenesis----04916** | **Gene Symbol** | **Gene Name** |
|  | CREB3L4 | cAMP responsive element binding protein 3-like 4 |
|  | NRAS | neuroblastoma RAS viral (v-ras) oncogene homolog |
|  | FZD3 | frizzled homolog 3 (Drosophila) |
|  | WNT2 | wingless-type MMTV integration site family member 2 |
|  | CALM1 | calmodulin 1 (phosphorylase kinase, delta) |
|  | MAP2K1 | mitogen-activated protein kinase kinase 1 |
|  | FZD2 | frizzled homolog 2 (Drosophila) |
|  | PRKCA | protein kinase C, alpha |
|  | GNAI3 | guanine nucleotide binding protein (G protein), alpha inhibiting activity polypeptide 3 |
|  | GNAI3 | guanine nucleotide binding protein (G protein), alpha inhibiting activity polypeptide 3 |
|  | GNAI3 | guanine nucleotide binding protein (G protein), alpha inhibiting activity polypeptide 3 |
|  | MAP2K2 | mitogen-activated protein kinase kinase 2 |
|  | TCF7L2 | transcription factor 7-like 2 (T-cell specific, HMG-box) |
|  | TCF7L2 | transcription factor 7-like 2 (T-cell specific, HMG-box) |
|  | TCF7L2 | transcription factor 7-like 2 (T-cell specific, HMG-box) |
|  | TCF7L2 | transcription factor 7-like 2 (T-cell specific, HMG-box) |
|  | TCF7L2 | transcription factor 7-like 2 (T-cell specific, HMG-box) |
|  | TCF7L2 | transcription factor 7-like 2 (T-cell specific, HMG-box) |
|  | FZD7 | frizzled homolog 7 (Drosophila) |
|  | FZD7 | frizzled homolog 7 (Drosophila) |
|  | WNT5A | wingless-type MMTV integration site family, member 5A |
|  | WNT5A | wingless-type MMTV integration site family, member 5A |
|  | CALM2 | calmodulin 2 (phosphorylase kinase, delta) |
|  | CAMK2D | calcium/calmodulin-dependent protein kinase II delta |
|  | GNAO1 | guanine nucleotide binding protein (G protein), alpha activating activity polypeptide O |
|  | WNT10A | wingless-type MMTV integration site family, member 10A |
|  | MITF | microphthalmia-associated transcription factor |
|  | MITF | microphthalmia-associated transcription factor |
|  | ADCY3 | adenylate cyclase 3 |
|  | EDNRB | endothelin receptor type B |
|  | EDNRB | endothelin receptor type B |
|  | GNAQ | guanine nucleotide binding protein (G protein), q polypeptide |
|  | CREB1 | cAMP responsive element binding protein 1 |
|  | MAPK1 | mitogen-activated protein kinase 1 |
|  | MAPK1 | mitogen-activated protein kinase 1 |
|  | PRKACB | protein kinase, cAMP-dependent, catalytic, beta |
|  | CAMK2G | calcium/calmodulin-dependent protein kinase II gamma |
|  | FZD1 | frizzled homolog 1 (Drosophila) |
|  | ADCY7 | adenylate cyclase 7 |
| **KEGG pathway----Dorso-ventral axis formation----04320** | **Gene Symbol** | **Gene Name** |
|  | GRB2 | growth factor receptor-bound protein 2 |
|  | NOTCH1 | Notch homolog 1, translocation-associated (Drosophila) |
|  | SPIRE2 | spire homolog 2 (Drosophila) |
|  | ETS2 | v-ets erythroblastosis virus E26 oncogene homolog 2 (avian) |
|  | ETS2 | v-ets erythroblastosis virus E26 oncogene homolog 2 (avian) |
|  | SOS1 | son of sevenless homolog 1 (Drosophila) |
|  | ETV6 | ets variant 6 |
|  | ETV6 | ets variant 6 |
|  | PIWIL4 | piwi-like 4 (Drosophila) |
|  | MAP2K1 | mitogen-activated protein kinase kinase 1 |
|  | NOTCH2 | Notch homolog 2 (Drosophila) |
|  | NOTCH2 | Notch homolog 2 (Drosophila) |
|  | MAPK1 | mitogen-activated protein kinase 1 |
|  | MAPK1 | mitogen-activated protein kinase 1 |
|  | EGFR | epidermal growth factor receptor (erythroblastic leukemia viral (v-erb-b) oncogene homolog, avian) |
|  | EGFR | epidermal growth factor receptor (erythroblastic leukemia viral (v-erb-b) oncogene homolog, avian) |
|  | EGFR | epidermal growth factor receptor (erythroblastic leukemia viral (v-erb-b) oncogene homolog, avian) |
| **KEGG pathway----Toll-like receptor signaling pathway----04620** | **Gene Symbol** | **Gene Name** |
|  | MAPK8 | mitogen-activated protein kinase 8 |
|  | IFNAR2 | interferon (alpha, beta and omega) receptor 2 |
|  | MAP2K1 | mitogen-activated protein kinase kinase 1 |
|  | CD14 | CD14 molecule |
|  | CCL5 | chemokine (C-C motif) ligand 5 |
|  | CCL5 | chemokine (C-C motif) ligand 5 |
|  | FOS | FBJ murine osteosarcoma viral oncogene homolog |
|  | PIK3CB | phosphoinositide-3-kinase, catalytic, beta polypeptide |
|  | MAP2K2 | mitogen-activated protein kinase kinase 2 |
|  | CXCL9 | chemokine (C-X-C motif) ligand 9 |
|  | JUN | jun oncogene |
|  | JUN | jun oncogene |
|  | JUN | jun oncogene |
|  | CXCL10 | chemokine (C-X-C motif) ligand 10 |
|  | SPP1 | secreted phosphoprotein 1 |
|  | TLR7 | toll-like receptor 7 |
|  | IRAK1 | interleukin-1 receptor-associated kinase 1 |
|  | MAP3K7IP2 | mitogen-activated protein kinase kinase kinase 7 interacting protein 2 |
|  | MAP3K7IP2 | mitogen-activated protein kinase kinase kinase 7 interacting protein 2 |
|  | AKT3 | v-akt murine thymoma viral oncogene homolog 3 (protein kinase B, gamma) |
|  | AKT3 | v-akt murine thymoma viral oncogene homolog 3 (protein kinase B, gamma) |
|  | AKT3 | v-akt murine thymoma viral oncogene homolog 3 (protein kinase B, gamma) |
|  | AKT3 | v-akt murine thymoma viral oncogene homolog 3 (protein kinase B, gamma) |
|  | NFKBIA | nuclear factor of kappa light polypeptide gene enhancer in B-cells inhibitor, alpha |
|  | IL8 | interleukin 8 |
|  | IKBKB | inhibitor of kappa light polypeptide gene enhancer in B-cells, kinase beta |
|  | IKBKB | inhibitor of kappa light polypeptide gene enhancer in B-cells, kinase beta |
|  | MAPK9 | mitogen-activated protein kinase 9 |
|  | PIK3CA | phosphoinositide-3-kinase, catalytic, alpha polypeptide |
|  | LY96 | lymphocyte antigen 96 |
|  | MAP3K7 | mitogen-activated protein kinase kinase kinase 7 |
|  | MAP3K7 | mitogen-activated protein kinase kinase kinase 7 |
|  | MAPK10 | mitogen-activated protein kinase 10 |
|  | MAPK1 | mitogen-activated protein kinase 1 |
|  | MAPK1 | mitogen-activated protein kinase 1 |
|  | MAP2K4 | mitogen-activated protein kinase kinase 4 |
| **KEGG pathway----Galactose metabolism----00052** | **Gene Symbol** | **Gene Name** |
|  | GAA | glucosidase, alpha; acid |
|  | UGP2 | UDP-glucose pyrophosphorylase 2 |
|  | UGP2 | UDP-glucose pyrophosphorylase 2 |
|  | PGM2 | phosphoglucomutase 2 |
|  | PGM2 | phosphoglucomutase 2 |
|  | PGM2 | phosphoglucomutase 2 |
|  | AKR1B1 | aldo-keto reductase family 1, member B1 (aldose reductase) |
|  | GLB1 | galactosidase, beta 1 |
|  | GLA | galactosidase, alpha |
|  | GALK2 | galactokinase 2 |
|  | PFKM | phosphofructokinase, muscle |
|  | B4GALT2 | UDP-Gal:betaGlcNAc beta 1,4- galactosyltransferase, polypeptide 2 |
|  | PFKP | phosphofructokinase, platelet |
|  | GALE | UDP-galactose-4-epimerase |
| **KEGG pathway----Butanoate metabolism----00650** | **Gene Symbol** | **Gene Name** |
|  | PDHA1 | pyruvate dehydrogenase (lipoamide) alpha 1 |
|  | ALDH2 | aldehyde dehydrogenase 2 family (mitochondrial) |
|  | ACAT2 | acetyl-Coenzyme A acetyltransferase 2 |
|  | ACAT2 | acetyl-Coenzyme A acetyltransferase 2 |
|  | ACAT2 | acetyl-Coenzyme A acetyltransferase 2 |
|  | HADH | hydroxyacyl-Coenzyme A dehydrogenase |
|  | OXCT1 | 3-oxoacid CoA transferase 1 |
|  | EHHADH | enoyl-Coenzyme A, hydratase/3-hydroxyacyl Coenzyme A dehydrogenase |
|  | HADHA | hydroxyacyl-Coenzyme A dehydrogenase/3-ketoacyl-Coenzyme A thiolase/enoyl-Coenzyme A hydratase (trifunctional protein), alpha subunit |
|  | BDH2 | 3-hydroxybutyrate dehydrogenase, type 2 |
|  | ACAT1 | acetyl-Coenzyme A acetyltransferase 1 |
|  | ALDH7A1 | aldehyde dehydrogenase 7 family, member A1 |
|  | ALDH7A1 | aldehyde dehydrogenase 7 family, member A1 |
|  | ABAT | 4-aminobutyrate aminotransferase |
|  | ALDH3A2 | aldehyde dehydrogenase 3 family, member A2 |
|  | PDHB | pyruvate dehydrogenase (lipoamide) beta |
| **KEGG pathway----Viral myocarditis----05416** | **Gene Symbol** | **Gene Name** |
|  | HLA-DRA | major histocompatibility complex, class II, DR alpha |
|  | HLA-DRA | major histocompatibility complex, class II, DR alpha |
|  | CD55 | CD55 molecule, decay accelerating factor for complement (Cromer blood group) |
|  | CD55 | CD55 molecule, decay accelerating factor for complement (Cromer blood group) |
|  | DMD | dystrophin |
|  | HLA-DQA1 | major histocompatibility complex, class II, DQ alpha 1 |
|  | HLA-DQA1 | major histocompatibility complex, class II, DQ alpha 1 |
|  | MYH9 | myosin, heavy chain 9, non-muscle |
|  | CAV1 | caveolin 1, caveolae protein, 22kDa |
|  | CAV1 | caveolin 1, caveolae protein, 22kDa |
|  | MYH10 | myosin, heavy chain 10, non-muscle |
|  | ICAM1 | intercellular adhesion molecule 1 |
|  | MYH11 | myosin, heavy chain 11, smooth muscle |
|  | MYH11 | myosin, heavy chain 11, smooth muscle |
|  | CXADR | coxsackie virus and adenovirus receptor |
|  | HLA-DPA1 | major histocompatibility complex, class II, DP alpha 1 |
|  | CCND1 | cyclin D1 |
|  | CCND1 | cyclin D1 |
|  | ABL2 | v-abl Abelson murine leukemia viral oncogene homolog 2 (arg, Abelson-related gene) |
|  | ITGB2 | integrin, beta 2 (complement component 3 receptor 3 and 4 subunit) |
|  | RAC2 | ras-related C3 botulinum toxin substrate 2 (rho family, small GTP binding protein Rac2) |
|  | EIF4G1 | eukaryotic translation initiation factor 4 gamma, 1 |
|  | FYN | FYN oncogene related to SRC, FGR, YES |
|  | HLA-DQB1 | major histocompatibility complex, class II, DQ beta 1 |
|  | HLA-DQB1 | major histocompatibility complex, class II, DQ beta 1 |
|  | MYH14 | myosin, heavy chain 14 |
|  | MYH14 | myosin, heavy chain 14 |
|  | SGCB | sarcoglycan, beta (43kDa dystrophin-associated glycoprotein) |
|  | SGCB | sarcoglycan, beta (43kDa dystrophin-associated glycoprotein) |
|  | SGCB | sarcoglycan, beta (43kDa dystrophin-associated glycoprotein) |
|  | HLA-DRB5 | major histocompatibility complex, class II, DR beta 5 |
| **KEGG pathway----Antigen processing and presentation----04612** | **Gene Symbol** | **Gene Name** |
|  | HLA-DRA | major histocompatibility complex, class II, DR alpha |
|  | HLA-DRA | major histocompatibility complex, class II, DR alpha |
|  | HLA-DQA1 | major histocompatibility complex, class II, DQ alpha 1 |
|  | HLA-DQA1 | major histocompatibility complex, class II, DQ alpha 1 |
|  | CTSB | cathepsin B |
|  | CTSB | cathepsin B |
|  | HSP90AB1 | heat shock protein 90kDa alpha (cytosolic), class B member 1 |
|  | HSP90AB1 | heat shock protein 90kDa alpha (cytosolic), class B member 1 |
|  | HSPA8 | heat shock 70kDa protein 8 |
|  | HSPA8 | heat shock 70kDa protein 8 |
|  | HSPA8 | heat shock 70kDa protein 8 |
|  | HSPA5 | heat shock 70kDa protein 5 (glucose-regulated protein, 78kDa) |
|  | CANX | calnexin |
|  | CANX | calnexin |
|  | CANX | calnexin |
|  | HSPA1A | heat shock 70kDa protein 1A |
|  | HSPA1B | heat shock 70kDa protein 1B |
|  | HSPA6 | heat shock 70kDa protein 6 (HSP70B') |
|  | HLA-DPA1 | major histocompatibility complex, class II, DP alpha 1 |
|  | CALR | calreticulin |
|  | CTSL1 | cathepsin L1 |
|  | HSPA4 | heat shock 70kDa protein 4 |
|  | CTSS | cathepsin S |
|  | CTSS | cathepsin S |
|  | HLA-DQB1 | major histocompatibility complex, class II, DQ beta 1 |
|  | HLA-DQB1 | major histocompatibility complex, class II, DQ beta 1 |
|  | NFYC | nuclear transcription factor Y, gamma |
|  | NFYC | nuclear transcription factor Y, gamma |
|  | NFYC | nuclear transcription factor Y, gamma |
|  | NFYC | nuclear transcription factor Y, gamma |
|  | IFI30 | interferon, gamma-inducible protein 30 |
|  | CREB1 | cAMP responsive element binding protein 1 |
|  | LGMN | legumain |
|  | PSME3 | proteasome (prosome, macropain) activator subunit 3 (PA28 gamma; Ki) |
|  | PSME3 | proteasome (prosome, macropain) activator subunit 3 (PA28 gamma; Ki) |
|  | NFYB | nuclear transcription factor Y, beta |
|  | NFYB | nuclear transcription factor Y, beta |
|  | NFYB | nuclear transcription factor Y, beta |
|  | RFXAP | regulatory factor X-associated protein |
|  | HLA-DRB5 | major histocompatibility complex, class II, DR beta 5 |
| **KEGG pathway----Adipocytokine signaling pathway----04920** | **Gene Symbol** | **Gene Name** |
|  | ACSL5 | acyl-CoA synthetase long-chain family member 5 |
|  | ACSL5 | acyl-CoA synthetase long-chain family member 5 |
|  | MAPK8 | mitogen-activated protein kinase 8 |
|  | ACACB | acetyl-Coenzyme A carboxylase beta |
|  | PRKAB1 | protein kinase, AMP-activated, beta 1 non-catalytic subunit |
|  | ACSL1 | acyl-CoA synthetase long-chain family member 1 |
|  | ACSL1 | acyl-CoA synthetase long-chain family member 1 |
|  | PRKAB2 | protein kinase, AMP-activated, beta 2 non-catalytic subunit |
|  | PRKAB2 | protein kinase, AMP-activated, beta 2 non-catalytic subunit |
|  | RXRA | retinoid X receptor, alpha |
|  | PRKAA1 | protein kinase, AMP-activated, alpha 1 catalytic subunit |
|  | ADIPOR2 | adiponectin receptor 2 |
|  | CD36 | CD36 molecule (thrombospondin receptor) |
|  | LEPR | leptin receptor |
|  | SOCS3 | suppressor of cytokine signaling 3 |
|  | IRS1 | insulin receptor substrate 1 |
|  | IRS2 | insulin receptor substrate 2 |
|  | IRS2 | insulin receptor substrate 2 |
|  | AKT3 | v-akt murine thymoma viral oncogene homolog 3 (protein kinase B, gamma) |
|  | AKT3 | v-akt murine thymoma viral oncogene homolog 3 (protein kinase B, gamma) |
|  | AKT3 | v-akt murine thymoma viral oncogene homolog 3 (protein kinase B, gamma) |
|  | AKT3 | v-akt murine thymoma viral oncogene homolog 3 (protein kinase B, gamma) |
|  | NFKBIA | nuclear factor of kappa light polypeptide gene enhancer in B-cells inhibitor, alpha |
|  | MAPK9 | mitogen-activated protein kinase 9 |
|  | IKBKB | inhibitor of kappa light polypeptide gene enhancer in B-cells, kinase beta |
|  | IKBKB | inhibitor of kappa light polypeptide gene enhancer in B-cells, kinase beta |
|  | ACSL4 | acyl-CoA synthetase long-chain family member 4 |
|  | MAPK10 | mitogen-activated protein kinase 10 |
| **KEGG pathway----Circadian rhythm - mammal----04710** | **Gene Symbol** | **Gene Name** |
|  | BHLHE40 | basic helix-loop-helix family, member e40 |
|  | BHLHE40 | basic helix-loop-helix family, member e40 |
|  | CRY1 | cryptochrome 1 (photolyase-like) |
|  | NR1D1 | nuclear receptor subfamily 1, group D, member 1 |
|  | NPAS2 | neuronal PAS domain protein 2 |
|  | NPAS2 | neuronal PAS domain protein 2 |
|  | BHLHE41 | basic helix-loop-helix family, member e41 |
|  | BHLHE41 | basic helix-loop-helix family, member e41 |
|  | CSNK1D | casein kinase 1, delta |
|  | PER2 | period homolog 2 (Drosophila) |
| **KEGG pathway----Arachidonic acid metabolism----00590** | **Gene Symbol** | **Gene Name** |
|  | GPX3 | glutathione peroxidase 3 (plasma) |
|  | CYP2C9 | cytochrome P450, family 2, subfamily C, polypeptide 9 |
|  | JMJD7-PLA2G4B | JMJD7-PLA2G4B readthrough |
|  | PLA2G12A | phospholipase A2, group XIIA |
|  | AKR1C3 | aldo-keto reductase family 1, member C3 (3-alpha hydroxysteroid dehydrogenase, type II) |
|  | PLA2G2A | phospholipase A2, group IIA (platelets, synovial fluid) |
|  | ALOX5 | arachidonate 5-lipoxygenase |
|  | ALOX5 | arachidonate 5-lipoxygenase |
|  | ALOX5 | arachidonate 5-lipoxygenase |
|  | PTGS2 | prostaglandin-endoperoxide synthase 2 (prostaglandin G/H synthase and cyclooxygenase) |
|  | PTGS2 | prostaglandin-endoperoxide synthase 2 (prostaglandin G/H synthase and cyclooxygenase) |
|  | CBR1 | carbonyl reductase 1 |
|  | PTGDS | prostaglandin D2 synthase 21kDa (brain) |
|  | PTGDS | prostaglandin D2 synthase 21kDa (brain) |
|  | PLA2G1B | phospholipase A2, group IB (pancreas) |
|  | GPX2 | glutathione peroxidase 2 (gastrointestinal) |
|  | GPX2 | glutathione peroxidase 2 (gastrointestinal) |
|  | PTGS1 | prostaglandin-endoperoxide synthase 1 (prostaglandin G/H synthase and cyclooxygenase) |
|  | CBR3 | carbonyl reductase 3 |
|  | GPX7 | glutathione peroxidase 7 |
|  | PTGES | prostaglandin E synthase |
|  | PLA2G6 | phospholipase A2, group VI (cytosolic, calcium-independent) |
| **KEGG pathway----Jak-STAT signaling pathway----04630** | **Gene Symbol** | **Gene Name** |
|  | IFNAR2 | interferon (alpha, beta and omega) receptor 2 |
|  | SOCS2 | suppressor of cytokine signaling 2 |
|  | IL20RB | interleukin 20 receptor beta |
|  | IL13RA1 | interleukin 13 receptor, alpha 1 |
|  | IL13RA1 | interleukin 13 receptor, alpha 1 |
|  | BCL2L1 | BCL2-like 1 |
|  | CCND1 | cyclin D1 |
|  | CCND1 | cyclin D1 |
|  | IL6ST | interleukin 6 signal transducer (gp130, oncostatin M receptor) |
|  | MYC | v-myc myelocytomatosis viral oncogene homolog (avian) |
|  | PRLR | prolactin receptor |
|  | PIK3CA | phosphoinositide-3-kinase, catalytic, alpha polypeptide |
|  | PIM1 | pim-1 oncogene |
|  | IL4R | interleukin 4 receptor |
|  | IFNGR1 | interferon gamma receptor 1 |
|  | CCND2 | cyclin D2 |
|  | SPRY2 | sprouty homolog 2 (Drosophila) |
|  | SOCS5 | suppressor of cytokine signaling 5 |
|  | SOCS5 | suppressor of cytokine signaling 5 |
|  | SOCS5 | suppressor of cytokine signaling 5 |
|  | IFNE | interferon, epsilon |
|  | LIF | leukemia inhibitory factor (cholinergic differentiation factor) |
|  | EPOR | erythropoietin receptor |
|  | SOS1 | son of sevenless homolog 1 (Drosophila) |
|  | PTPN6 | protein tyrosine phosphatase, non-receptor type 6 |
|  | IL2RG | interleukin 2 receptor, gamma (severe combined immunodeficiency) |
|  | CBLB | Cas-Br-M (murine) ecotropic retroviral transforming sequence b |
|  | LEPR | leptin receptor |
|  | PIK3CB | phosphoinositide-3-kinase, catalytic, beta polypeptide |
|  | SOCS3 | suppressor of cytokine signaling 3 |
|  | SOCS7 | suppressor of cytokine signaling 7 |
|  | PIAS3 | protein inhibitor of activated STAT, 3 |
|  | GRB2 | growth factor receptor-bound protein 2 |
|  | JAK1 | Janus kinase 1 |
|  | STAM2 | signal transducing adaptor molecule (SH3 domain and ITAM motif) 2 |
|  | STAT6 | signal transducer and activator of transcription 6, interleukin-4 induced |
|  | STAT6 | signal transducer and activator of transcription 6, interleukin-4 induced |
|  | AKT3 | v-akt murine thymoma viral oncogene homolog 3 (protein kinase B, gamma) |
|  | AKT3 | v-akt murine thymoma viral oncogene homolog 3 (protein kinase B, gamma) |
|  | AKT3 | v-akt murine thymoma viral oncogene homolog 3 (protein kinase B, gamma) |
|  | AKT3 | v-akt murine thymoma viral oncogene homolog 3 (protein kinase B, gamma) |
|  | SPRY4 | sprouty homolog 4 (Drosophila) |
| **KEGG pathway----Cytokine-cytokine receptor interaction----04060** | **Gene Symbol** | **Gene Name** |
|  | IFNAR2 | interferon (alpha, beta and omega) receptor 2 |
|  | ACVR1 | activin A receptor, type I |
|  | BMPR2 | bone morphogenetic protein receptor, type II (serine/threonine kinase) |
|  | CCL2 | chemokine (C-C motif) ligand 2 |
|  | TNFRSF21 | tumor necrosis factor receptor superfamily, member 21 |
|  | IL20RB | interleukin 20 receptor beta |
|  | IL13RA1 | interleukin 13 receptor, alpha 1 |
|  | IL13RA1 | interleukin 13 receptor, alpha 1 |
|  | TGFBR1 | transforming growth factor, beta receptor 1 |
|  | TGFBR1 | transforming growth factor, beta receptor 1 |
|  | CXCL2 | chemokine (C-X-C motif) ligand 2 |
|  | TNFRSF25 | tumor necrosis factor receptor superfamily, member 25 |
|  | TNFRSF25 | tumor necrosis factor receptor superfamily, member 25 |
|  | TNFRSF25 | tumor necrosis factor receptor superfamily, member 25 |
|  | CCL5 | chemokine (C-C motif) ligand 5 |
|  | CCL5 | chemokine (C-C motif) ligand 5 |
|  | PDGFRA | platelet-derived growth factor receptor, alpha polypeptide |
|  | BMP2 | bone morphogenetic protein 2 |
|  | TNFRSF14 | tumor necrosis factor receptor superfamily, member 14 (herpesvirus entry mediator) |
|  | CXCL9 | chemokine (C-X-C motif) ligand 9 |
|  | CXCL16 | chemokine (C-X-C motif) ligand 16 |
|  | BMP7 | bone morphogenetic protein 7 |
|  | BMP7 | bone morphogenetic protein 7 |
|  | IL6ST | interleukin 6 signal transducer (gp130, oncostatin M receptor) |
|  | PRLR | prolactin receptor |
|  | IL1R2 | interleukin 1 receptor, type II |
|  | ACVR2A | activin A receptor, type IIA |
|  | TNFRSF12A | tumor necrosis factor receptor superfamily, member 12A |
|  | IL4R | interleukin 4 receptor |
|  | IFNGR1 | interferon gamma receptor 1 |
|  | TNFRSF11A | tumor necrosis factor receptor superfamily, member 11a, NFKB activator |
|  | IL1RAP | interleukin 1 receptor accessory protein |
|  | INHBA | inhibin, beta A |
|  | PDGFRB | platelet-derived growth factor receptor, beta polypeptide |
|  | VEGFC | vascular endothelial growth factor C |
|  | IFNE | interferon, epsilon |
|  | LIF | leukemia inhibitory factor (cholinergic differentiation factor) |
|  | TGFB1 | transforming growth factor, beta 1 |
|  | PDGFC | platelet derived growth factor C |
|  | CXCL13 | chemokine (C-X-C motif) ligand 13 |
|  | EPOR | erythropoietin receptor |
|  | ACVR1B | activin A receptor, type IB |
|  | TNFRSF10B | tumor necrosis factor receptor superfamily, member 10b |
|  | IL2RG | interleukin 2 receptor, gamma (severe combined immunodeficiency) |
|  | TNFSF10 | tumor necrosis factor (ligand) superfamily, member 10 |
|  | ACVR2B | activin A receptor, type IIB |
|  | VEGFA | vascular endothelial growth factor A |
|  | VEGFA | vascular endothelial growth factor A |
|  | VEGFA | vascular endothelial growth factor A |
|  | LEPR | leptin receptor |
|  | EGFR | epidermal growth factor receptor (erythroblastic leukemia viral (v-erb-b) oncogene homolog, avian) |
|  | EGFR | epidermal growth factor receptor (erythroblastic leukemia viral (v-erb-b) oncogene homolog, avian) |
|  | EGFR | epidermal growth factor receptor (erythroblastic leukemia viral (v-erb-b) oncogene homolog, avian) |
|  | FAS | Fas (TNF receptor superfamily, member 6) |
|  | FAS | Fas (TNF receptor superfamily, member 6) |
|  | FAS | Fas (TNF receptor superfamily, member 6) |
|  | FAS | Fas (TNF receptor superfamily, member 6) |
|  | IL1R1 | interleukin 1 receptor, type I |
|  | CXCL10 | chemokine (C-X-C motif) ligand 10 |
|  | CXCR4 | chemokine (C-X-C motif) receptor 4 |
|  | CXCR4 | chemokine (C-X-C motif) receptor 4 |
|  | IL8 | interleukin 8 |
|  | CXCL1 | chemokine (C-X-C motif) ligand 1 (melanoma growth stimulating activity, alpha) |
|  | TNFSF15 | tumor necrosis factor (ligand) superfamily, member 15 |
|  | BMPR1A | bone morphogenetic protein receptor, type IA |
|  | CCL18 | chemokine (C-C motif) ligand 18 (pulmonary and activation-regulated) |
|  | CCL18 | chemokine (C-C motif) ligand 18 (pulmonary and activation-regulated) |
| **KEGG pathway----Systemic lupus erythematosus----05322** | **Gene Symbol** | **Gene Name** |
|  | HLA-DRA | major histocompatibility complex, class II, DR alpha |
|  | HLA-DRA | major histocompatibility complex, class II, DR alpha |
|  | C3 | complement component 3 |
|  | H2AFZ | H2A histone family, member Z |
|  | H2AFZ | H2A histone family, member Z |
|  | HLA-DQA1 | major histocompatibility complex, class II, DQ alpha 1 |
|  | HLA-DQA1 | major histocompatibility complex, class II, DQ alpha 1 |
|  | C4A | complement component 4A (Rodgers blood group) |
|  | SNRPB | small nuclear ribonucleoprotein polypeptides B and B1 |
|  | H2AFJ | H2A histone family, member J |
|  | H2AFJ | H2A histone family, member J |
|  | HIST1H2BK | histone cluster 1, H2bk |
|  | C1R | complement component 1, r subcomponent |
|  | ACTN1 | actinin, alpha 1 |
|  | ACTN1 | actinin, alpha 1 |
|  | ACTN1 | actinin, alpha 1 |
|  | H3F3B | H3 histone, family 3B (H3.3B) |
|  | SNRPD3 | small nuclear ribonucleoprotein D3 polypeptide 18kDa |
|  | H2AFV | H2A histone family, member V |
|  | H2AFV | H2A histone family, member V |
|  | H2AFV | H2A histone family, member V |
|  | H2AFV | H2A histone family, member V |
|  | C1QB | complement component 1, q subcomponent, B chain |
|  | HIST1H2BE | histone cluster 1, H2be |
|  | HLA-DPA1 | major histocompatibility complex, class II, DP alpha 1 |
|  | SNRPD1 | small nuclear ribonucleoprotein D1 polypeptide 16kDa |
|  | SNRPD1 | small nuclear ribonucleoprotein D1 polypeptide 16kDa |
|  | C1QA | complement component 1, q subcomponent, A chain |
|  | HLA-DQB1 | major histocompatibility complex, class II, DQ beta 1 |
|  | HLA-DQB1 | major histocompatibility complex, class II, DQ beta 1 |
|  | HIST1H4C | histone cluster 1, H4c |
|  | H2AFX | H2A histone family, member X |
|  | FCGR2A | Fc fragment of IgG, low affinity IIa, receptor (CD32) |
|  | H2AFY | H2A histone family, member Y |
|  | H2AFY | H2A histone family, member Y |
|  | C1S | complement component 1, s subcomponent |
|  | SSB | Sjogren syndrome antigen B (autoantigen La) |
|  | SSB | Sjogren syndrome antigen B (autoantigen La) |
|  | HIST1H2BH | histone cluster 1, H2bh |
|  | HIST1H2BF | histone cluster 1, H2bf |
|  | HIST1H2BD | histone cluster 1, H2bd |
|  | TROVE2 | TROVE domain family, member 2 |
|  | HLA-DRB5 | major histocompatibility complex, class II, DR beta 5 |
|  | HIST3H2A | histone cluster 3, H2a |
| **KEGG pathway----Biosynthesis of unsaturated fatty acids----01040** | **Gene Symbol** | **Gene Name** |
|  | FADS1 | fatty acid desaturase 1 |
|  | FADS1 | fatty acid desaturase 1 |
|  | FADS1 | fatty acid desaturase 1 |
|  | ELOVL5 | ELOVL family member 5, elongation of long chain fatty acids (FEN1/Elo2, SUR4/Elo3-like, yeast) |
|  | PTPLB | protein tyrosine phosphatase-like (proline instead of catalytic arginine), member b |
|  | PTPLB | protein tyrosine phosphatase-like (proline instead of catalytic arginine), member b |
|  | SCD5 | stearoyl-CoA desaturase 5 |
|  | SCD5 | stearoyl-CoA desaturase 5 |
|  | HADHA | hydroxyacyl-Coenzyme A dehydrogenase/3-ketoacyl-Coenzyme A thiolase/enoyl-Coenzyme A hydratase (trifunctional protein), alpha subunit |
|  | ACOX1 | acyl-Coenzyme A oxidase 1, palmitoyl |
|  | ACOX1 | acyl-Coenzyme A oxidase 1, palmitoyl |
|  | ACOX1 | acyl-Coenzyme A oxidase 1, palmitoyl |
|  | SCD | stearoyl-CoA desaturase (delta-9-desaturase) |
|  | SCD | stearoyl-CoA desaturase (delta-9-desaturase) |
|  | SCD | stearoyl-CoA desaturase (delta-9-desaturase) |
|  | YOD1 | YOD1 OTU deubiquinating enzyme 1 homolog (S. cerevisiae) |
|  | ACOT7 | acyl-CoA thioesterase 7 |
| **KEGG pathway----Glycine, serine and threonine metabolism----00260** | **Gene Symbol** | **Gene Name** |
|  | AOC3 | amine oxidase, copper containing 3 (vascular adhesion protein 1) |
|  | PSAT1 | phosphoserine aminotransferase 1 |
|  | SRR | serine racemase |
|  | ALAS1 | aminolevulinate, delta-, synthase 1 |
|  | MAOA | monoamine oxidase A |
|  | MAOA | monoamine oxidase A |
|  | MAOA | monoamine oxidase A |
|  | SHMT2 | serine hydroxymethyltransferase 2 (mitochondrial) |
|  | SHMT2 | serine hydroxymethyltransferase 2 (mitochondrial) |
|  | DLD | dihydrolipoamide dehydrogenase |
|  | PHGDH | phosphoglycerate dehydrogenase |
|  | GATM | glycine amidinotransferase (L-arginine:glycine amidinotransferase) |
|  | AMT | aminomethyltransferase |
|  | PSPH | phosphoserine phosphatase |
| **KEGG pathway----Terpenoid backbone biosynthesis----00900** | **Gene Symbol** | **Gene Name** |
|  | ACAT1 | acetyl-Coenzyme A acetyltransferase 1 |
|  | IDI1 | isopentenyl-diphosphate delta isomerase 1 |
|  | IDI1 | isopentenyl-diphosphate delta isomerase 1 |
|  | ACAT2 | acetyl-Coenzyme A acetyltransferase 2 |
|  | ACAT2 | acetyl-Coenzyme A acetyltransferase 2 |
|  | ACAT2 | acetyl-Coenzyme A acetyltransferase 2 |
|  | PMVK | phosphomevalonate kinase |
|  | FDPS | farnesyl diphosphate synthase (farnesyl pyrophosphate synthetase, dimethylallyltranstransferase, geranyltranstransferase) |
|  | HMGCR | 3-hydroxy-3-methylglutaryl-Coenzyme A reductase |
|  | HMGCR | 3-hydroxy-3-methylglutaryl-Coenzyme A reductase |
|  | PDSS1 | prenyl (decaprenyl) diphosphate synthase, subunit 1 |
| **KEGG pathway----Type II diabetes mellitus----04930** | **Gene Symbol** | **Gene Name** |
|  | IRS2 | insulin receptor substrate 2 |
|  | IRS2 | insulin receptor substrate 2 |
|  | MAPK8 | mitogen-activated protein kinase 8 |
|  | IRS1 | insulin receptor substrate 1 |
|  | INSR | insulin receptor |
|  | INSR | insulin receptor |
|  | IKBKB | inhibitor of kappa light polypeptide gene enhancer in B-cells, kinase beta |
|  | IKBKB | inhibitor of kappa light polypeptide gene enhancer in B-cells, kinase beta |
|  | SOCS2 | suppressor of cytokine signaling 2 |
|  | MAPK9 | mitogen-activated protein kinase 9 |
|  | PRKCZ | protein kinase C, zeta |
|  | PIK3CA | phosphoinositide-3-kinase, catalytic, alpha polypeptide |
|  | MAPK10 | mitogen-activated protein kinase 10 |
|  | MAPK1 | mitogen-activated protein kinase 1 |
|  | MAPK1 | mitogen-activated protein kinase 1 |
|  | PIK3CB | phosphoinositide-3-kinase, catalytic, beta polypeptide |
|  | INS | insulin |
|  | SOCS3 | suppressor of cytokine signaling 3 |
| **KEGG pathway----Keratan sulfate biosynthesis----00533** | **Gene Symbol** | **Gene Name** |
|  | B3GNT7 | UDP-GlcNAc:betaGal beta-1,3-N-acetylglucosaminyltransferase 7 |
|  | B3GNT7 | UDP-GlcNAc:betaGal beta-1,3-N-acetylglucosaminyltransferase 7 |
|  | CHST6 | carbohydrate (N-acetylglucosamine 6-O) sulfotransferase 6 |
|  | B3GNT2 | UDP-GlcNAc:betaGal beta-1,3-N-acetylglucosaminyltransferase 2 |
|  | B4GALT3 | UDP-Gal:betaGlcNAc beta 1,4- galactosyltransferase, polypeptide 3 |
|  | B4GALT4 | UDP-Gal:betaGlcNAc beta 1,4- galactosyltransferase, polypeptide 4 |
|  | ST3GAL1 | ST3 beta-galactoside alpha-2,3-sialyltransferase 1 |
|  | B4GALT2 | UDP-Gal:betaGlcNAc beta 1,4- galactosyltransferase, polypeptide 2 |
| **KEGG pathway----Starch and sucrose metabolism----00500** | **Gene Symbol** | **Gene Name** |
|  | UGT1A1 | UDP glucuronosyltransferase 1 family, polypeptide A1 |
|  | UXS1 | UDP-glucuronate decarboxylase 1 |
|  | UXS1 | UDP-glucuronate decarboxylase 1 |
|  | GAA | glucosidase, alpha; acid |
|  | AMY1A | amylase, alpha 1A (salivary) |
|  | UGP2 | UDP-glucose pyrophosphorylase 2 |
|  | UGP2 | UDP-glucose pyrophosphorylase 2 |
|  | PGM2 | phosphoglucomutase 2 |
|  | PGM2 | phosphoglucomutase 2 |
|  | PGM2 | phosphoglucomutase 2 |
|  | UGT1A9 | UDP glucuronosyltransferase 1 family, polypeptide A9 |
|  | UGDH | UDP-glucose dehydrogenase |
|  | SI | sucrase-isomaltase (alpha-glucosidase) |
|  | PGM2L1 | phosphoglucomutase 2-like 1 |
|  | PYGL | phosphorylase, glycogen, liver |
|  | UGT1A6 | UDP glucuronosyltransferase 1 family, polypeptide A6 |
|  | AGL | amylo-1, 6-glucosidase, 4-alpha-glucanotransferase |
|  | UGT1A3 | UDP glucuronosyltransferase 1 family, polypeptide A3 |
|  | PYGB | phosphorylase, glycogen; brain |
| **KEGG pathway----Nicotinate and nicotinamide metabolism----00760** | **Gene Symbol** | **Gene Name** |
|  | C9orf95 | chromosome 9 open reading frame 95 |
|  | NT5C2 | 5'-nucleotidase, cytosolic II |
|  | NADSYN1 | NAD synthetase 1 |
|  | NNMT | nicotinamide N-methyltransferase |
|  | NNMT | nicotinamide N-methyltransferase |
|  | NP | nucleoside phosphorylase |
|  | NNT | nicotinamide nucleotide transhydrogenase |
|  | NNT | nicotinamide nucleotide transhydrogenase |
|  | NUDT12 | nudix (nucleoside diphosphate linked moiety X)-type motif 12 |
|  | NAMPT | nicotinamide phosphoribosyltransferase |
|  | NAMPT | nicotinamide phosphoribosyltransferase |
|  | NT5E | 5'-nucleotidase, ecto (CD73) |
| **KEGG pathway----Fructose and mannose metabolism----00051** | **Gene Symbol** | **Gene Name** |
|  | SORD | sorbitol dehydrogenase |
|  | ALDOC | aldolase C, fructose-bisphosphate |
|  | FBP1 | fructose-1,6-bisphosphatase 1 |
|  | AKR1B1 | aldo-keto reductase family 1, member B1 (aldose reductase) |
|  | MTMR6 | myotubularin related protein 6 |
|  | FPGT | fucose-1-phosphate guanylyltransferase |
|  | GMDS | GDP-mannose 4,6-dehydratase |
|  | ALDOB | aldolase B, fructose-bisphosphate |
|  | MTMR2 | myotubularin related protein 2 |
|  | MTMR2 | myotubularin related protein 2 |
|  | MTMR2 | myotubularin related protein 2 |
|  | PFKM | phosphofructokinase, muscle |
|  | PFKP | phosphofructokinase, platelet |
| **KEGG pathway----Other glycan degradation----00511** | **Gene Symbol** | **Gene Name** |
|  | FUCA1 | fucosidase, alpha-L- 1, tissue |
|  | GLB1 | galactosidase, beta 1 |
|  | MAN2C1 | mannosidase, alpha, class 2C, member 1 |
|  | AGA | aspartylglucosaminidase |
|  | AGA | aspartylglucosaminidase |
|  | AGA | aspartylglucosaminidase |
|  | NEU1 | sialidase 1 (lysosomal sialidase) |
|  | HEXB | hexosaminidase B (beta polypeptide) |
|  | GBA | glucosidase, beta; acid (includes glucosylceramidase) |
| **KEGG pathway----Linoleic acid metabolism----00591** | **Gene Symbol** | **Gene Name** |
|  | CYP2C9 | cytochrome P450, family 2, subfamily C, polypeptide 9 |
|  | PLA2G1B | phospholipase A2, group IB (pancreas) |
|  | JMJD7-PLA2G4B | JMJD7-PLA2G4B readthrough |
|  | CYP3A7 | cytochrome P450, family 3, subfamily A, polypeptide 7 |
|  | CYP3A5 | cytochrome P450, family 3, subfamily A, polypeptide 5 |
|  | CYP3A43 | cytochrome P450, family 3, subfamily A, polypeptide 43 |
|  | PLA2G12A | phospholipase A2, group XIIA |
|  | PLA2G6 | phospholipase A2, group VI (cytosolic, calcium-independent) |
|  | CYP3A4 | cytochrome P450, family 3, subfamily A, polypeptide 4 |
|  | PLA2G2A | phospholipase A2, group IIA (platelets, synovial fluid) |
| **KEGG pathway----RIG-I-like receptor signaling pathway----04622** | **Gene Symbol** | **Gene Name** |
|  | MAPK8 | mitogen-activated protein kinase 8 |
|  | IFNE | interferon, epsilon |
|  | CYLD | cylindromatosis (turban tumor syndrome) |
|  | CYLD | cylindromatosis (turban tumor syndrome) |
|  | DDX3Y | DEAD (Asp-Glu-Ala-Asp) box polypeptide 3, Y-linked |
|  | ATG12 | ATG12 autophagy related 12 homolog (S. cerevisiae) |
|  | SIKE1 | suppressor of IKBKE 1 |
|  | SIKE1 | suppressor of IKBKE 1 |
|  | AZI2 | 5-azacytidine induced 2 |
|  | AZI2 | 5-azacytidine induced 2 |
|  | DDX3X | DEAD (Asp-Glu-Ala-Asp) box polypeptide 3, X-linked |
|  | CXCL10 | chemokine (C-X-C motif) ligand 10 |
|  | TANK | TRAF family member-associated NFKB activator |
|  | MAPK9 | mitogen-activated protein kinase 9 |
|  | IKBKB | inhibitor of kappa light polypeptide gene enhancer in B-cells, kinase beta |
|  | IKBKB | inhibitor of kappa light polypeptide gene enhancer in B-cells, kinase beta |
|  | IL8 | interleukin 8 |
|  | NFKBIA | nuclear factor of kappa light polypeptide gene enhancer in B-cells inhibitor, alpha |
|  | ATG5 | ATG5 autophagy related 5 homolog (S. cerevisiae) |
|  | ATG5 | ATG5 autophagy related 5 homolog (S. cerevisiae) |
|  | MAP3K7 | mitogen-activated protein kinase kinase kinase 7 |
|  | MAP3K7 | mitogen-activated protein kinase kinase kinase 7 |
|  | MAPK10 | mitogen-activated protein kinase 10 |
|  | TMEM173 | transmembrane protein 173 |
| **KEGG pathway----Riboflavin metabolism----00740** | **Gene Symbol** | **Gene Name** |
|  | ACP1 | acid phosphatase 1, soluble |
|  | ACP1 | acid phosphatase 1, soluble |
|  | ACP1 | acid phosphatase 1, soluble |
|  | FLAD1 | FAD1 flavin adenine dinucleotide synthetase homolog (S. cerevisiae) |
|  | MTMR6 | myotubularin related protein 6 |
|  | RFK | riboflavin kinase |
|  | MTMR2 | myotubularin related protein 2 |
|  | MTMR2 | myotubularin related protein 2 |
|  | MTMR2 | myotubularin related protein 2 |
|  | ACP6 | acid phosphatase 6, lysophosphatidic |
|  | ACP5 | acid phosphatase 5, tartrate resistant |
| **KEGG pathway----One carbon pool by folate----00670** | **Gene Symbol** | **Gene Name** |
|  | DHFR | dihydrofolate reductase |
|  | DHFR | dihydrofolate reductase |
|  | DHFR | dihydrofolate reductase |
|  | TYMS | thymidylate synthetase |
|  | TYMS | thymidylate synthetase |
|  | SHMT2 | serine hydroxymethyltransferase 2 (mitochondrial) |
|  | SHMT2 | serine hydroxymethyltransferase 2 (mitochondrial) |
|  | GART | phosphoribosylglycinamide formyltransferase, phosphoribosylglycinamide synthetase, phosphoribosylaminoimidazole synthetase |
|  | GART | phosphoribosylglycinamide formyltransferase, phosphoribosylglycinamide synthetase, phosphoribosylaminoimidazole synthetase |
|  | GART | phosphoribosylglycinamide formyltransferase, phosphoribosylglycinamide synthetase, phosphoribosylaminoimidazole synthetase |
|  | MTHFD2 | methylenetetrahydrofolate dehydrogenase (NADP+ dependent) 2, methenyltetrahydrofolate cyclohydrolase |
|  | AMT | aminomethyltransferase |
|  | MTHFD1 | methylenetetrahydrofolate dehydrogenase (NADP+ dependent) 1, methenyltetrahydrofolate cyclohydrolase, formyltetrahydrofolate synthetase |
| **KEGG pathway----Glycosylphosphatidylinositol(GPI)-anchor biosynthesis----00563** | **Gene Symbol** | **Gene Name** |
|  | PIGM | phosphatidylinositol glycan anchor biosynthesis, class M |
|  | PIGW | phosphatidylinositol glycan anchor biosynthesis, class W |
|  | PIGT | phosphatidylinositol glycan anchor biosynthesis, class T |
|  | PIGT | phosphatidylinositol glycan anchor biosynthesis, class T |
|  | PIGK | phosphatidylinositol glycan anchor biosynthesis, class K |
|  | PIGK | phosphatidylinositol glycan anchor biosynthesis, class K |
|  | PIGP | phosphatidylinositol glycan anchor biosynthesis, class P |
|  | PGAP1 | post-GPI attachment to proteins 1 |
|  | PGAP1 | post-GPI attachment to proteins 1 |
|  | PIGF | phosphatidylinositol glycan anchor biosynthesis, class F |
|  | PIGF | phosphatidylinositol glycan anchor biosynthesis, class F |
|  | PIGY | phosphatidylinositol glycan anchor biosynthesis, class Y |
|  | PIGX | phosphatidylinositol glycan anchor biosynthesis, class X |
|  | PIGX | phosphatidylinositol glycan anchor biosynthesis, class X |
| **KEGG pathway----Hedgehog signaling pathway----04340** | **Gene Symbol** | **Gene Name** |
|  | BMP2 | bone morphogenetic protein 2 |
|  | CSNK1G1 | casein kinase 1, gamma 1 |
|  | CSNK1G1 | casein kinase 1, gamma 1 |
|  | WNT10A | wingless-type MMTV integration site family, member 10A |
|  | BMP7 | bone morphogenetic protein 7 |
|  | BMP7 | bone morphogenetic protein 7 |
|  | WNT2 | wingless-type MMTV integration site family member 2 |
|  | SHH | sonic hedgehog homolog (Drosophila) |
|  | IHH | Indian hedgehog homolog (Drosophila) |
|  | RAB23 | RAB23, member RAS oncogene family |
|  | STK36 | serine/threonine kinase 36, fused homolog (Drosophila) |
|  | STK36 | serine/threonine kinase 36, fused homolog (Drosophila) |
|  | CSNK1A1 | casein kinase 1, alpha 1 |
|  | CSNK1A1 | casein kinase 1, alpha 1 |
|  | PRKACB | protein kinase, cAMP-dependent, catalytic, beta |
|  | CSNK1D | casein kinase 1, delta |
|  | GAS1 | growth arrest-specific 1 |
|  | BTRC | beta-transducin repeat containing |
|  | WNT5A | wingless-type MMTV integration site family, member 5A |
|  | WNT5A | wingless-type MMTV integration site family, member 5A |
| **KEGG pathway----Tyrosine metabolism----00350** | **Gene Symbol** | **Gene Name** |
|  | ADH5 | alcohol dehydrogenase 5 (class III), chi polypeptide |
|  | ADH5 | alcohol dehydrogenase 5 (class III), chi polypeptide |
|  | ADH7 | alcohol dehydrogenase 7 (class IV), mu or sigma polypeptide |
|  | AOC3 | amine oxidase, copper containing 3 (vascular adhesion protein 1) |
|  | ALDH3B1 | aldehyde dehydrogenase 3 family, member B1 |
|  | ALDH3B1 | aldehyde dehydrogenase 3 family, member B1 |
|  | METTL6 | methyltransferase like 6 |
|  | ALDH1A3 | aldehyde dehydrogenase 1 family, member A3 |
|  | WBSCR22 | Williams Beuren syndrome chromosome region 22 |
|  | MAOA | monoamine oxidase A |
|  | MAOA | monoamine oxidase A |
|  | MAOA | monoamine oxidase A |
|  | COMT | catechol-O-methyltransferase |
|  | NAT5 | N-acetyltransferase 5 (GCN5-related, putative) |
|  | LCLAT1 | lysocardiolipin acyltransferase 1 |
|  | METTL2B | methyltransferase like 2B |
|  | ALDH3B2 | aldehyde dehydrogenase 3 family, member B2 |
| **KEGG pathway----Methane metabolism----00680** | **Gene Symbol** | **Gene Name** |
|  | CAT | catalase |
|  | ADH5 | alcohol dehydrogenase 5 (class III), chi polypeptide |
|  | ADH5 | alcohol dehydrogenase 5 (class III), chi polypeptide |
|  | SHMT2 | serine hydroxymethyltransferase 2 (mitochondrial) |
|  | SHMT2 | serine hydroxymethyltransferase 2 (mitochondrial) |
|  | PRDX6 | peroxiredoxin 6 |
|  | PRDX6 | peroxiredoxin 6 |
| **KEGG pathway----Fatty acid biosynthesis----00061** | **Gene Symbol** | **Gene Name** |
|  | ACACB | acetyl-Coenzyme A carboxylase beta |
|  | OXSM | 3-oxoacyl-ACP synthase, mitochondrial |
|  | ACACA | acetyl-Coenzyme A carboxylase alpha |
|  | FASN | fatty acid synthase |
| **KEGG pathway----O-Glycan biosynthesis----00512** | **Gene Symbol** | **Gene Name** |
|  | C1GALT1 | core 1 synthase, glycoprotein-N-acetylgalactosamine 3-beta-galactosyltransferase, 1 |
|  | GALNT2 | UDP-N-acetyl-alpha-D-galactosamine:polypeptide N-acetylgalactosaminyltransferase 2 (GalNAc-T2) |
|  | GALNT2 | UDP-N-acetyl-alpha-D-galactosamine:polypeptide N-acetylgalactosaminyltransferase 2 (GalNAc-T2) |
|  | GALNT5 | UDP-N-acetyl-alpha-D-galactosamine:polypeptide N-acetylgalactosaminyltransferase 5 (GalNAc-T5) |
|  | GALNT12 | UDP-N-acetyl-alpha-D-galactosamine:polypeptide N-acetylgalactosaminyltransferase 12 (GalNAc-T12) |
|  | GALNT12 | UDP-N-acetyl-alpha-D-galactosamine:polypeptide N-acetylgalactosaminyltransferase 12 (GalNAc-T12) |
|  | OGT | O-linked N-acetylglucosamine (GlcNAc) transferase (UDP-N-acetylglucosamine:polypeptide-N-acetylglucosaminyl transferase) |
|  | OGT | O-linked N-acetylglucosamine (GlcNAc) transferase (UDP-N-acetylglucosamine:polypeptide-N-acetylglucosaminyl transferase) |
|  | C1GALT1C1 | C1GALT1-specific chaperone 1 |
|  | ST6GALNAC1 | ST6 (alpha-N-acetyl-neuraminyl-2,3-beta-galactosyl-1,3)-N-acetylgalactosaminide alpha-2,6-sialyltransferase 1 |
|  | GALNT10 | UDP-N-acetyl-alpha-D-galactosamine:polypeptide N-acetylgalactosaminyltransferase 10 (GalNAc-T10) |
|  | GALNT10 | UDP-N-acetyl-alpha-D-galactosamine:polypeptide N-acetylgalactosaminyltransferase 10 (GalNAc-T10) |
|  | ST3GAL1 | ST3 beta-galactoside alpha-2,3-sialyltransferase 1 |
|  | GALNT14 | UDP-N-acetyl-alpha-D-galactosamine:polypeptide N-acetylgalactosaminyltransferase 14 (GalNAc-T14) |
| **KEGG pathway----Phenylalanine metabolism----00360** | **Gene Symbol** | **Gene Name** |
|  | AOC3 | amine oxidase, copper containing 3 (vascular adhesion protein 1) |
|  | ALDH3B1 | aldehyde dehydrogenase 3 family, member B1 |
|  | ALDH3B1 | aldehyde dehydrogenase 3 family, member B1 |
|  | PRDX6 | peroxiredoxin 6 |
|  | PRDX6 | peroxiredoxin 6 |
|  | ALDH1A3 | aldehyde dehydrogenase 1 family, member A3 |
|  | MAOA | monoamine oxidase A |
|  | MAOA | monoamine oxidase A |
|  | MAOA | monoamine oxidase A |
|  | NAT5 | N-acetyltransferase 5 (GCN5-related, putative) |
|  | LCLAT1 | lysocardiolipin acyltransferase 1 |
|  | ALDH3B2 | aldehyde dehydrogenase 3 family, member B2 |
| **KEGG pathway----Phosphatidylinositol signaling system----04070** | **Gene Symbol** | **Gene Name** |
|  | CDS2 | CDP-diacylglycerol synthase (phosphatidate cytidylyltransferase) 2 |
|  | INPP5D | inositol polyphosphate-5-phosphatase, 145kDa |
|  | DGKQ | diacylglycerol kinase, theta 110kDa |
|  | CALM1 | calmodulin 1 (phosphorylase kinase, delta) |
|  | DGKA | diacylglycerol kinase, alpha 80kDa |
|  | PRKCA | protein kinase C, alpha |
|  | ITPR3 | inositol 1,4,5-triphosphate receptor, type 3 |
|  | INPP1 | inositol polyphosphate-1-phosphatase |
|  | PIK3CB | phosphoinositide-3-kinase, catalytic, beta polypeptide |
|  | PIP4K2A | phosphatidylinositol-5-phosphate 4-kinase, type II, alpha |
|  | PIK3C2A | phosphoinositide-3-kinase, class 2, alpha polypeptide |
|  | PIK3C2A | phosphoinositide-3-kinase, class 2, alpha polypeptide |
|  | CALM2 | calmodulin 2 (phosphorylase kinase, delta) |
|  | INPP5A | inositol polyphosphate-5-phosphatase, 40kDa |
|  | ITPR1 | inositol 1,4,5-triphosphate receptor, type 1 |
|  | PIK3CA | phosphoinositide-3-kinase, catalytic, alpha polypeptide |
|  | INPP4B | inositol polyphosphate-4-phosphatase, type II, 105kDa |
|  | PIK3C2B | phosphoinositide-3-kinase, class 2, beta polypeptide |
|  | IMPA2 | inositol(myo)-1(or 4)-monophosphatase 2 |
| **KEGG pathway----Glycosphingolipid biosynthesis - globo series----00603** | **Gene Symbol** | **Gene Name** |
|  | B3GALNT1 | beta-1,3-N-acetylgalactosaminyltransferase 1 (globoside blood group) |
|  | B3GALNT1 | beta-1,3-N-acetylgalactosaminyltransferase 1 (globoside blood group) |
|  | GLA | galactosidase, alpha |
|  | FUT9 | fucosyltransferase 9 (alpha (1,3) fucosyltransferase) |
|  | ST3GAL1 | ST3 beta-galactoside alpha-2,3-sialyltransferase 1 |
|  | NAGA | N-acetylgalactosaminidase, alpha- |
|  | NAGA | N-acetylgalactosaminidase, alpha- |
|  | HEXB | hexosaminidase B (beta polypeptide) |
| **KEGG pathway----Calcium signaling pathway----04020** | **Gene Symbol** | **Gene Name** |
|  | ATP2A3 | ATPase, Ca++ transporting, ubiquitous |
|  | PPP3CB | protein phosphatase 3 (formerly 2B), catalytic subunit, beta isoform |
|  | PRKCA | protein kinase C, alpha |
|  | PDGFRA | platelet-derived growth factor receptor, alpha polypeptide |
|  | ERBB4 | v-erb-a erythroblastic leukemia viral oncogene homolog 4 (avian) |
|  | CALM2 | calmodulin 2 (phosphorylase kinase, delta) |
|  | PTGER3 | prostaglandin E receptor 3 (subtype EP3) |
|  | ATP2A2 | ATPase, Ca++ transporting, cardiac muscle, slow twitch 2 |
|  | PPID | peptidylprolyl isomerase D |
|  | PPID | peptidylprolyl isomerase D |
|  | PPID | peptidylprolyl isomerase D |
|  | ADCY3 | adenylate cyclase 3 |
|  | VDAC3 | voltage-dependent anion channel 3 |
|  | TRPC1 | transient receptor potential cation channel, subfamily C, member 1 |
|  | EDNRB | endothelin receptor type B |
|  | EDNRB | endothelin receptor type B |
|  | ERBB3 | v-erb-b2 erythroblastic leukemia viral oncogene homolog 3 (avian) |
|  | ERBB3 | v-erb-b2 erythroblastic leukemia viral oncogene homolog 3 (avian) |
|  | F2R | coagulation factor II (thrombin) receptor |
|  | PRKACB | protein kinase, cAMP-dependent, catalytic, beta |
|  | CAMK2G | calcium/calmodulin-dependent protein kinase II gamma |
|  | ATP2B1 | ATPase, Ca++ transporting, plasma membrane 1 |
|  | ATP2B1 | ATPase, Ca++ transporting, plasma membrane 1 |
|  | ATP2B1 | ATPase, Ca++ transporting, plasma membrane 1 |
|  | PDGFRB | platelet-derived growth factor receptor, beta polypeptide |
|  | ADCY7 | adenylate cyclase 7 |
|  | PPP3CA | protein phosphatase 3 (formerly 2B), catalytic subunit, alpha isoform |
|  | PPP3CA | protein phosphatase 3 (formerly 2B), catalytic subunit, alpha isoform |
|  | CALM1 | calmodulin 1 (phosphorylase kinase, delta) |
|  | ITPR3 | inositol 1,4,5-triphosphate receptor, type 3 |
|  | VDAC1 | voltage-dependent anion channel 1 |
|  | SLC25A5 | solute carrier family 25 (mitochondrial carrier; adenine nucleotide translocator), member 5 |
|  | SLC8A1 | solute carrier family 8 (sodium/calcium exchanger), member 1 |
|  | EGFR | epidermal growth factor receptor (erythroblastic leukemia viral (v-erb-b) oncogene homolog, avian) |
|  | EGFR | epidermal growth factor receptor (erythroblastic leukemia viral (v-erb-b) oncogene homolog, avian) |
|  | EGFR | epidermal growth factor receptor (erythroblastic leukemia viral (v-erb-b) oncogene homolog, avian) |
|  | EDNRA | endothelin receptor type A |
|  | CAMK2D | calcium/calmodulin-dependent protein kinase II delta |
|  | ITPR1 | inositol 1,4,5-triphosphate receptor, type 1 |
|  | MYLK | myosin light chain kinase |
|  | PPP3R1 | protein phosphatase 3 (formerly 2B), regulatory subunit B, alpha isoform |
|  | GNAQ | guanine nucleotide binding protein (G protein), q polypeptide |
|  | HRH1 | histamine receptor H1 |
| **KEGG pathway----Cysteine and methionine metabolism----00270** | **Gene Symbol** | **Gene Name** |
|  | ENOPH1 | enolase-phosphatase 1 |
|  | ADI1 | acireductone dioxygenase 1 |
|  | AMD1 | adenosylmethionine decarboxylase 1 |
|  | AMD1 | adenosylmethionine decarboxylase 1 |
|  | DNMT3B | DNA (cytosine-5-)-methyltransferase 3 beta |
|  | AHCY | adenosylhomocysteinase |
|  | APIP | APAF1 interacting protein |
|  | DNMT1 | DNA (cytosine-5-)-methyltransferase 1 |
|  | MAT2A | methionine adenosyltransferase II, alpha |
|  | LDHB | lactate dehydrogenase B |
|  | SMS | spermine synthase |
| **KEGG pathway----Glycosphingolipid biosynthesis - ganglio series----00604** | **Gene Symbol** | **Gene Name** |
|  | GLB1 | galactosidase, beta 1 |
|  | B3GALT4 | UDP-Gal:betaGlcNAc beta 1,3-galactosyltransferase, polypeptide 4 |
|  | ST3GAL1 | ST3 beta-galactoside alpha-2,3-sialyltransferase 1 |
|  | ST3GAL5 | ST3 beta-galactoside alpha-2,3-sialyltransferase 5 |
|  | HEXB | hexosaminidase B (beta polypeptide) |
|  | SLC33A1 | solute carrier family 33 (acetyl-CoA transporter), member 1 |
|  | SLC33A1 | solute carrier family 33 (acetyl-CoA transporter), member 1 |
| **KEGG pathway----Glyoxylate and dicarboxylate metabolism----00630** | **Gene Symbol** | **Gene Name** |
|  | MDH1 | malate dehydrogenase 1, NAD (soluble) |
|  | GRHPR | glyoxylate reductase/hydroxypyruvate reductase |
|  | GRHPR | glyoxylate reductase/hydroxypyruvate reductase |
|  | GRHPR | glyoxylate reductase/hydroxypyruvate reductase |
|  | MTHFD2 | methylenetetrahydrofolate dehydrogenase (NADP+ dependent) 2, methenyltetrahydrofolate cyclohydrolase |
|  | PGP | phosphoglycolate phosphatase |
|  | MDH2 | malate dehydrogenase 2, NAD (mitochondrial) |
|  | MTHFD1 | methylenetetrahydrofolate dehydrogenase (NADP+ dependent) 1, methenyltetrahydrofolate cyclohydrolase, formyltetrahydrofolate synthetase |
| **KEGG pathway----Pantothenate and CoA biosynthesis----00770** | **Gene Symbol** | **Gene Name** |
|  | PANK2 | pantothenate kinase 2 |
|  | PANK2 | pantothenate kinase 2 |
|  | BCAT1 | branched chain aminotransferase 1, cytosolic |
|  | BCAT1 | branched chain aminotransferase 1, cytosolic |
|  | DPYD | dihydropyrimidine dehydrogenase |
|  | PANK4 | pantothenate kinase 4 |
|  | PANK1 | pantothenate kinase 1 |
|  | BCAT2 | branched chain aminotransferase 2, mitochondrial |
| **KEGG pathway----Cytosolic DNA-sensing pathway----04623** | **Gene Symbol** | **Gene Name** |
|  | CXCL10 | chemokine (C-X-C motif) ligand 10 |
|  | AIM2 | absent in melanoma 2 |
|  | POLR1D | polymerase (RNA) I polypeptide D, 16kDa |
|  | NFKBIA | nuclear factor of kappa light polypeptide gene enhancer in B-cells inhibitor, alpha |
|  | PYCARD | PYD and CARD domain containing |
|  | IKBKB | inhibitor of kappa light polypeptide gene enhancer in B-cells, kinase beta |
|  | IKBKB | inhibitor of kappa light polypeptide gene enhancer in B-cells, kinase beta |
|  | TREX1 | three prime repair exonuclease 1 |
|  | CCL5 | chemokine (C-C motif) ligand 5 |
|  | CCL5 | chemokine (C-C motif) ligand 5 |
|  | POLR3C | polymerase (RNA) III (DNA directed) polypeptide C (62kD) |
|  | POLR1C | polymerase (RNA) I polypeptide C, 30kDa |
|  | POLR1C | polymerase (RNA) I polypeptide C, 30kDa |
|  | CASP1 | caspase 1, apoptosis-related cysteine peptidase (interleukin 1, beta, convertase) |
|  | POLR3K | polymerase (RNA) III (DNA directed) polypeptide K, 12.3 kDa |
|  | TMEM173 | transmembrane protein 173 |
|  | POLR3F | polymerase (RNA) III (DNA directed) polypeptide F, 39 kDa |
| **KEGG pathway----D-Glutamine and D-glutamate metabolism----00471** | **Gene Symbol** | **Gene Name** |
|  | GLS | glutaminase |
|  | GLS | glutaminase |
|  | GLS | glutaminase |
|  | GLUD1 | glutamate dehydrogenase 1 |
|  | GLUD2 | glutamate dehydrogenase 2 |
| **KEGG pathway----Regulation of autophagy----04140** | **Gene Symbol** | **Gene Name** |
|  | ATG4C | ATG4 autophagy related 4 homolog C (S. cerevisiae) |
|  | ATG3 | ATG3 autophagy related 3 homolog (S. cerevisiae) |
|  | PRKAA1 | protein kinase, AMP-activated, alpha 1 catalytic subunit |
|  | PIK3R4 | phosphoinositide-3-kinase, regulatory subunit 4 |
|  | GABARAPL2 | GABA(A) receptor-associated protein-like 2 |
|  | ATG12 | ATG12 autophagy related 12 homolog (S. cerevisiae) |
|  | ATG5 | ATG5 autophagy related 5 homolog (S. cerevisiae) |
|  | ATG5 | ATG5 autophagy related 5 homolog (S. cerevisiae) |
|  | INS | insulin |
|  | ULK2 | unc-51-like kinase 2 (C. elegans) |
|  | GABARAPL1 | GABA(A) receptor-associated protein like 1 |
| **KEGG pathway----Ascorbate and aldarate metabolism----00053** | **Gene Symbol** | **Gene Name** |
|  | UGT1A1 | UDP glucuronosyltransferase 1 family, polypeptide A1 |
|  | ALDH2 | aldehyde dehydrogenase 2 family (mitochondrial) |
|  | UGT1A9 | UDP glucuronosyltransferase 1 family, polypeptide A9 |
|  | UGDH | UDP-glucose dehydrogenase |
|  | UGT1A6 | UDP glucuronosyltransferase 1 family, polypeptide A6 |
|  | ALDH7A1 | aldehyde dehydrogenase 7 family, member A1 |
|  | ALDH7A1 | aldehyde dehydrogenase 7 family, member A1 |
|  | ALDH3A2 | aldehyde dehydrogenase 3 family, member A2 |
|  | UGT1A3 | UDP glucuronosyltransferase 1 family, polypeptide A3 |
