# Supplementary material for: Expression Microarray Meta-Analysis Identifies Genes Associated with Ras/MAPK and Related Pathways in Progression of Muscle-Invasive Bladder Transition Cell Carcinoma
Source: PLoS One. 2013 Feb 1;8(2):e55414. doi: 10.1371/journal.pone.0055414 (PMC3562183; doi:10.1371/journal.pone.0055414)
Supplement: Table S4 — Genes differentially expressed, both increased and decreased, in Ta/T1-staged bladder tumors vs T2 tumors, and associated with KEGG pathways, including Top 10 pathways listen in Table 3 . Pathways selected based on Fisher's Exact Scores ≤0.01. (DOC) [file pone.0055414.s004.doc]

| **Pathway** | **Gene Symbol** | **Gene Name** |
| --- | --- | --- |
| **KEGG pathway----Metabolic pathways----01100** | ARG2 | arginase, type II |
|  | ARG2 | arginase, type II |
|  | FUK | fucokinase |
|  | PAPSS2 | 3'-phosphoadenosine 5'-phosphosulfate synthase 2 |
|  | PAPSS2 | 3'-phosphoadenosine 5'-phosphosulfate synthase 2 |
|  | PRDX6 | peroxiredoxin 6 |
|  | DGKA | diacylglycerol kinase, alpha 80kDa |
|  | MCCC1 | methylcrotonoyl-Coenzyme A carboxylase 1 (alpha) |
|  | ETNK1 | ethanolamine kinase 1 |
|  | LIPG | lipase, endothelial |
|  | BCAT1 | branched chain aminotransferase 1, cytosolic |
|  | BCAT1 | branched chain aminotransferase 1, cytosolic |
|  | GLUD1 | glutamate dehydrogenase 1 |
|  | CHKB | choline kinase beta |
|  | ABAT | 4-aminobutyrate aminotransferase |
|  | ABAT | 4-aminobutyrate aminotransferase |
|  | EXT2 | exostoses (multiple) 2 |
|  | EXT2 | exostoses (multiple) 2 |
|  | SDHA | succinate dehydrogenase complex, subunit A, flavoprotein (Fp) |
|  | ST3GAL4 | ST3 beta-galactoside alpha-2,3-sialyltransferase 4 |
|  | TYMP | thymidine phosphorylase |
|  | GOT2 | glutamic-oxaloacetic transaminase 2, mitochondrial (aspartate aminotransferase 2) |
|  | PANK4 | pantothenate kinase 4 |
|  | ACAA2 | acetyl-Coenzyme A acyltransferase 2 |
|  | MECR | mitochondrial trans-2-enoyl-CoA reductase |
|  | NDUFB8 | NADH dehydrogenase (ubiquinone) 1 beta subcomplex, 8, 19kDa |
|  | NDUFV3 | NADH dehydrogenase (ubiquinone) flavoprotein 3, 10kDa |
|  | COMT | catechol-O-methyltransferase |
|  | COMT | catechol-O-methyltransferase |
|  | AHCYL1 | adenosylhomocysteinase-like 1 |
|  | AHCYL1 | adenosylhomocysteinase-like 1 |
|  | ACSL5 | acyl-CoA synthetase long-chain family member 5 |
|  | ACSL5 | acyl-CoA synthetase long-chain family member 5 |
|  | DTYMK | deoxythymidylate kinase (thymidylate kinase) |
|  | COQ7 | coenzyme Q7 homolog, ubiquinone (yeast) |
|  | B3GALT4 | UDP-Gal:betaGlcNAc beta 1,3-galactosyltransferase, polypeptide 4 |
|  | IDH3A | isocitrate dehydrogenase 3 (NAD+) alpha |
|  | PPAP2C | phosphatidic acid phosphatase type 2C |
|  | PLCE1 | phospholipase C, epsilon 1 |
|  | HSD17B2 | hydroxysteroid (17-beta) dehydrogenase 2 |
|  | ST3GAL5 | ST3 beta-galactoside alpha-2,3-sialyltransferase 5 |
|  | CYP4F3 | cytochrome P450, family 4, subfamily F, polypeptide 3 |
|  | CYP4F3 | cytochrome P450, family 4, subfamily F, polypeptide 3 |
|  | CYP2A7 | cytochrome P450, family 2, subfamily A, polypeptide 7 |
|  | ACO2 | aconitase 2, mitochondrial |
|  | GRHPR | glyoxylate reductase/hydroxypyruvate reductase |
|  | ALDH5A1 | aldehyde dehydrogenase 5 family, member A1 |
|  | UGT1A9 | UDP glucuronosyltransferase 1 family, polypeptide A9 |
|  | FBP1 | fructose-1,6-bisphosphatase 1 |
|  | CYP3A5 | cytochrome P450, family 3, subfamily A, polypeptide 5 |
|  | MAOA | monoamine oxidase A |
|  | MAOA | monoamine oxidase A |
|  | MAOA | monoamine oxidase A |
|  | COX5A | cytochrome c oxidase subunit Va |
|  | MLYCD | malonyl-CoA decarboxylase |
|  | PIK3C2B | phosphoinositide-3-kinase, class 2, beta polypeptide |
|  | GALNT10 | UDP-N-acetyl-alpha-D-galactosamine:polypeptide N-acetylgalactosaminyltransferase 10 (GalNAc-T10) |
|  | CHPT1 | choline phosphotransferase 1 |
|  | CHPT1 | choline phosphotransferase 1 |
|  | CHPT1 | choline phosphotransferase 1 |
|  | PFKP | phosphofructokinase, platelet |
|  | UGT1A1 | UDP glucuronosyltransferase 1 family, polypeptide A1 |
|  | SEPHS2 | selenophosphate synthetase 2 |
|  | NADSYN1 | NAD synthetase 1 |
|  | NADSYN1 | NAD synthetase 1 |
|  | PIGN | phosphatidylinositol glycan anchor biosynthesis, class N |
|  | NDUFS1 | NADH dehydrogenase (ubiquinone) Fe-S protein 1, 75kDa (NADH-coenzyme Q reductase) |
|  | NNMT | nicotinamide N-methyltransferase |
|  | NNMT | nicotinamide N-methyltransferase |
|  | SORD | sorbitol dehydrogenase |
|  | CYP2J2 | cytochrome P450, family 2, subfamily J, polypeptide 2 |
|  | ALG12 | asparagine-linked glycosylation 12, alpha-1,6-mannosyltransferase homolog (S. cerevisiae) |
|  | ME3 | malic enzyme 3, NADP(+)-dependent, mitochondrial |
|  | MAN1A2 | mannosidase, alpha, class 1A, member 2 |
|  | NDUFS6 | NADH dehydrogenase (ubiquinone) Fe-S protein 6, 13kDa (NADH-coenzyme Q reductase) |
|  | ASAH1 | N-acylsphingosine amidohydrolase (acid ceramidase) 1 |
|  | APRT | adenine phosphoribosyltransferase |
|  | PI4KA | phosphatidylinositol 4-kinase, catalytic, alpha |
|  | COX4I1 | cytochrome c oxidase subunit IV isoform 1 |
|  | COX4I1 | cytochrome c oxidase subunit IV isoform 1 |
|  | DHRS3 | dehydrogenase/reductase (SDR family) member 3 |
|  | PGAP1 | post-GPI attachment to proteins 1 |
|  | UGT1A3 | UDP glucuronosyltransferase 1 family, polypeptide A3 |
|  | SGPL1 | sphingosine-1-phosphate lyase 1 |
|  | SGPL1 | sphingosine-1-phosphate lyase 1 |
|  | C1GALT1 | core 1 synthase, glycoprotein-N-acetylgalactosamine 3-beta-galactosyltransferase, 1 |
|  | INPP5K | inositol polyphosphate-5-phosphatase K |
|  | INPP5K | inositol polyphosphate-5-phosphatase K |
|  | NDUFV1 | NADH dehydrogenase (ubiquinone) flavoprotein 1, 51kDa |
|  | PFKL | phosphofructokinase, liver |
|  | INPP5A | inositol polyphosphate-5-phosphatase, 40kDa |
|  | MGAT1 | mannosyl (alpha-1,3-)-glycoprotein beta-1,2-N-acetylglucosaminyltransferase |
|  | CTPS2 | CTP synthase II |
|  | UGT1A6 | UDP glucuronosyltransferase 1 family, polypeptide A6 |
|  | HMGCL | 3-hydroxymethyl-3-methylglutaryl-Coenzyme A lyase |
|  | PTGS1 | prostaglandin-endoperoxide synthase 1 (prostaglandin G/H synthase and cyclooxygenase) |
|  | UGT1A8 | UDP glucuronosyltransferase 1 family, polypeptide A8 |
|  | PCYT2 | phosphate cytidylyltransferase 2, ethanolamine |
|  | NDUFA3 | NADH dehydrogenase (ubiquinone) 1 alpha subcomplex, 3, 9kDa |
|  | ALDH3A2 | aldehyde dehydrogenase 3 family, member A2 |
|  | MCCC2 | methylcrotonoyl-Coenzyme A carboxylase 2 (beta) |
|  | IMPA2 | inositol(myo)-1(or 4)-monophosphatase 2 |
|  | MTHFD1 | methylenetetrahydrofolate dehydrogenase (NADP+ dependent) 1, methenyltetrahydrofolate cyclohydrolase, formyltetrahydrofolate synthetase |
|  | SMS | spermine synthase |
|  | NDUFA4L2 | NADH dehydrogenase (ubiquinone) 1 alpha subcomplex, 4-like 2 |
|  | ADH7 | alcohol dehydrogenase 7 (class IV), mu or sigma polypeptide |
|  | GUSB | glucuronidase, beta |
|  | BCKDHB | branched chain keto acid dehydrogenase E1, beta polypeptide |
|  | HMGCS2 | 3-hydroxy-3-methylglutaryl-Coenzyme A synthase 2 (mitochondrial) |
|  | AK3L1 | adenylate kinase 3-like 1 |
|  | DCTD | dCMP deaminase |
|  | HSD17B4 | hydroxysteroid (17-beta) dehydrogenase 4 |
|  | ALDH1B1 | aldehyde dehydrogenase 1 family, member B1 |
|  | NT5C2 | 5'-nucleotidase, cytosolic II |
|  | GALNT2 | UDP-N-acetyl-alpha-D-galactosamine:polypeptide N-acetylgalactosaminyltransferase 2 (GalNAc-T2) |
|  | POLR2J | polymerase (RNA) II (DNA directed) polypeptide J, 13.3kDa |
|  | ACADSB | acyl-Coenzyme A dehydrogenase, short/branched chain |
|  | ACADSB | acyl-Coenzyme A dehydrogenase, short/branched chain |
|  | PPAP2B | phosphatidic acid phosphatase type 2B |
|  | QARS | glutaminyl-tRNA synthetase |
|  | ACOX1 | acyl-Coenzyme A oxidase 1, palmitoyl |
|  | DEGS1 | degenerative spermatocyte homolog 1, lipid desaturase (Drosophila) |
|  | DEGS1 | degenerative spermatocyte homolog 1, lipid desaturase (Drosophila) |
|  | DGAT2 | diacylglycerol O-acyltransferase homolog 2 (mouse) |
|  | ACLY | ATP citrate lyase |
|  | SGMS2 | sphingomyelin synthase 2 |
|  | SQLE | squalene epoxidase |
|  | HK1 | hexokinase 1 |
|  | ACADVL | acyl-Coenzyme A dehydrogenase, very long chain |
|  | PHPT1 | phosphohistidine phosphatase 1 |
|  | NADK | NAD kinase |
| **KEGG pathway----Adherens junction----04520** | **Gene Symbol** | **Gene Name** |
|  | EP300 | E1A binding protein p300 |
|  | SORBS1 | sorbin and SH3 domain containing 1 |
|  | ACVR1B | activin A receptor, type IB |
|  | PTPRM | protein tyrosine phosphatase, receptor type, M |
|  | ERBB2 | v-erb-b2 erythroblastic leukemia viral oncogene homolog 2, neuro/glioblastoma derived oncogene homolog (avian) |
|  | TGFBR1 | transforming growth factor, beta receptor 1 |
|  | ACTN1 | actinin, alpha 1 |
|  | ACTN1 | actinin, alpha 1 |
|  | ACTN1 | actinin, alpha 1 |
|  | SRC | v-src sarcoma (Schmidt-Ruppin A-2) viral oncogene homolog (avian) |
|  | SSX2IP | synovial sarcoma, X breakpoint 2 interacting protein |
|  | FGFR1 | fibroblast growth factor receptor 1 |
|  | FGFR1 | fibroblast growth factor receptor 1 |
|  | SMAD3 | SMAD family member 3 |
|  | SMAD3 | SMAD family member 3 |
|  | INSR | insulin receptor |
|  | PTPRB | protein tyrosine phosphatase, receptor type, B |
|  | CDC42 | cell division cycle 42 (GTP binding protein, 25kDa) |
|  | PVRL2 | poliovirus receptor-related 2 (herpesvirus entry mediator B) |
|  | PVRL2 | poliovirus receptor-related 2 (herpesvirus entry mediator B) |
|  | PVRL2 | poliovirus receptor-related 2 (herpesvirus entry mediator B) |
|  | WASL | Wiskott-Aldrich syndrome-like |
|  | MAP3K7 | mitogen-activated protein kinase kinase kinase 7 |
| **KEGG pathway----Focal adhesion----04510** | **Gene Symbol** | **Gene Name** |
|  | LAMA4 | laminin, alpha 4 |
|  | MYL9 | myosin, light chain 9, regulatory |
|  | PDPK1 | 3-phosphoinositide dependent protein kinase-1 |
|  | PDGFC | platelet derived growth factor C |
|  | FN1 | fibronectin 1 |
|  | FN1 | fibronectin 1 |
|  | FN1 | fibronectin 1 |
|  | FN1 | fibronectin 1 |
|  | COL1A1 | collagen, type I, alpha 1 |
|  | ERBB2 | v-erb-b2 erythroblastic leukemia viral oncogene homolog 2, neuro/glioblastoma derived oncogene homolog (avian) |
|  | ACTN1 | actinin, alpha 1 |
|  | ACTN1 | actinin, alpha 1 |
|  | ACTN1 | actinin, alpha 1 |
|  | COL11A1 | collagen, type XI, alpha 1 |
|  | COL11A1 | collagen, type XI, alpha 1 |
|  | FLNA | filamin A, alpha |
|  | FLNA | filamin A, alpha |
|  | PTK2 | PTK2 protein tyrosine kinase 2 |
|  | VEGFA | vascular endothelial growth factor A |
|  | VEGFA | vascular endothelial growth factor A |
|  | VEGFA | vascular endothelial growth factor A |
|  | SRC | v-src sarcoma (Schmidt-Ruppin A-2) viral oncogene homolog (avian) |
|  | COL3A1 | collagen, type III, alpha 1 |
|  | PAK6 | p21 protein (Cdc42/Rac)-activated kinase 6 |
|  | PARVB | parvin, beta |
|  | CDC42 | cell division cycle 42 (GTP binding protein, 25kDa) |
|  | AKT3 | v-akt murine thymoma viral oncogene homolog 3 (protein kinase B, gamma) |
|  | AKT3 | v-akt murine thymoma viral oncogene homolog 3 (protein kinase B, gamma) |
|  | AKT3 | v-akt murine thymoma viral oncogene homolog 3 (protein kinase B, gamma) |
|  | AKT3 | v-akt murine thymoma viral oncogene homolog 3 (protein kinase B, gamma) |
|  | MYLK | myosin light chain kinase |
|  | MAPK9 | mitogen-activated protein kinase 9 |
|  | COL5A1 | collagen, type V, alpha 1 |
|  | COL5A1 | collagen, type V, alpha 1 |
|  | PDGFD | platelet derived growth factor D |
|  | ITGA11 | integrin, alpha 11 |
|  | COL1A2 | collagen, type I, alpha 2 |
|  | COL1A2 | collagen, type I, alpha 2 |
|  | COL1A2 | collagen, type I, alpha 2 |
|  | MAPK10 | mitogen-activated protein kinase 10 |
| **KEGG pathway----Endocytosis----04144** | **Gene Symbol** | **Gene Name** |
|  | CHMP2A | chromatin modifying protein 2A |
|  | PDCD6IP | programmed cell death 6 interacting protein |
|  | STAMBP | STAM binding protein |
|  | ITCH | itchy E3 ubiquitin protein ligase homolog (mouse) |
|  | ACVR1B | activin A receptor, type IB |
|  | USP8 | ubiquitin specific peptidase 8 |
|  | TGFBR1 | transforming growth factor, beta receptor 1 |
|  | SRC | v-src sarcoma (Schmidt-Ruppin A-2) viral oncogene homolog (avian) |
|  | SH3KBP1 | SH3-domain kinase binding protein 1 |
|  | SH3KBP1 | SH3-domain kinase binding protein 1 |
|  | SH3GLB1 | SH3-domain GRB2-like endophilin B1 |
|  | EPN3 | epsin 3 |
|  | EPN3 | epsin 3 |
|  | FGFR3 | fibroblast growth factor receptor 3 |
|  | VPS28 | vacuolar protein sorting 28 homolog (S. cerevisiae) |
|  | CDC42 | cell division cycle 42 (GTP binding protein, 25kDa) |
|  | EHD3 | EH-domain containing 3 |
|  | PRKCI | protein kinase C, iota |
|  | SH3GLB2 | SH3-domain GRB2-like endophilin B2 |
|  | DNM2 | dynamin 2 |
|  | PRKCZ | protein kinase C, zeta |
|  | RAB11FIP1 | RAB11 family interacting protein 1 (class I) |
|  | AP2B1 | adaptor-related protein complex 2, beta 1 subunit |
|  | ERBB3 | v-erb-b2 erythroblastic leukemia viral oncogene homolog 3 (avian) |
|  | ERBB3 | v-erb-b2 erythroblastic leukemia viral oncogene homolog 3 (avian) |
|  | SMURF1 | SMAD specific E3 ubiquitin protein ligase 1 |
|  | EHD1 | EH-domain containing 1 |
| **KEGG pathway----Valine, leucine and isoleucine degradation----00280** | **Gene Symbol** | **Gene Name** |
|  | ACADSB | acyl-Coenzyme A dehydrogenase, short/branched chain |
|  | ACADSB | acyl-Coenzyme A dehydrogenase, short/branched chain |
|  | OXCT1 | 3-oxoacid CoA transferase 1 |
|  | BCKDHB | branched chain keto acid dehydrogenase E1, beta polypeptide |
|  | ACAA2 | acetyl-Coenzyme A acyltransferase 2 |
|  | MCCC1 | methylcrotonoyl-Coenzyme A carboxylase 1 (alpha) |
|  | HMGCS2 | 3-hydroxy-3-methylglutaryl-Coenzyme A synthase 2 (mitochondrial) |
|  | HMGCL | 3-hydroxymethyl-3-methylglutaryl-Coenzyme A lyase |
|  | BCAT1 | branched chain aminotransferase 1, cytosolic |
|  | BCAT1 | branched chain aminotransferase 1, cytosolic |
|  | ABAT | 4-aminobutyrate aminotransferase |
|  | ABAT | 4-aminobutyrate aminotransferase |
|  | ALDH1B1 | aldehyde dehydrogenase 1 family, member B1 |
|  | ALDH3A2 | aldehyde dehydrogenase 3 family, member A2 |
|  | MCCC2 | methylcrotonoyl-Coenzyme A carboxylase 2 (beta) |
| **KEGG pathway----Insulin signaling pathway----04910** | **Gene Symbol** | **Gene Name** |
|  | PDPK1 | 3-phosphoinositide dependent protein kinase-1 |
|  | PRKAB1 | protein kinase, AMP-activated, beta 1 non-catalytic subunit |
|  | SORBS1 | sorbin and SH3 domain containing 1 |
|  | CALM1 | calmodulin 1 (phosphorylase kinase, delta) |
|  | CALM1 | calmodulin 1 (phosphorylase kinase, delta) |
|  | PYGL | phosphorylase, glycogen, liver |
|  | EXOC7 | exocyst complex component 7 |
|  | EXOC7 | exocyst complex component 7 |
|  | RHEB | Ras homolog enriched in brain |
|  | INPP5K | inositol polyphosphate-5-phosphatase K |
|  | INPP5K | inositol polyphosphate-5-phosphatase K |
|  | INSR | insulin receptor |
|  | FBP1 | fructose-1,6-bisphosphatase 1 |
|  | AKT3 | v-akt murine thymoma viral oncogene homolog 3 (protein kinase B, gamma) |
|  | AKT3 | v-akt murine thymoma viral oncogene homolog 3 (protein kinase B, gamma) |
|  | AKT3 | v-akt murine thymoma viral oncogene homolog 3 (protein kinase B, gamma) |
|  | AKT3 | v-akt murine thymoma viral oncogene homolog 3 (protein kinase B, gamma) |
|  | IKBKB | inhibitor of kappa light polypeptide gene enhancer in B-cells, kinase beta |
|  | MAPK9 | mitogen-activated protein kinase 9 |
|  | SREBF1 | sterol regulatory element binding transcription factor 1 |
|  | PRKCZ | protein kinase C, zeta |
|  | PRKCI | protein kinase C, iota |
|  | LIPE | lipase, hormone-sensitive |
|  | HK1 | hexokinase 1 |
|  | MAPK10 | mitogen-activated protein kinase 10 |
| **KEGG pathway----Lysosome----04142** | **Gene Symbol** | **Gene Name** |
|  | CTSL1 | cathepsin L1 |
|  | FUCA1 | fucosidase, alpha-L- 1, tissue |
|  | SCARB2 | scavenger receptor class B, member 2 |
|  | ENTPD4 | ectonucleoside triphosphate diphosphohydrolase 4 |
|  | CD164 | CD164 molecule, sialomucin |
|  | AP4S1 | adaptor-related protein complex 4, sigma 1 subunit |
|  | AP4S1 | adaptor-related protein complex 4, sigma 1 subunit |
|  | GUSB | glucuronidase, beta |
|  | CTSO | cathepsin O |
|  | AP3S2 | adaptor-related protein complex 3, sigma 2 subunit |
|  | GNPTAB | N-acetylglucosamine-1-phosphate transferase, alpha and beta subunits |
|  | LAPTM5 | lysosomal protein transmembrane 5 |
|  | ASAH1 | N-acylsphingosine amidohydrolase (acid ceramidase) 1 |
|  | SLC11A2 | solute carrier family 11 (proton-coupled divalent metal ion transporters), member 2 |
|  | SLC11A2 | solute carrier family 11 (proton-coupled divalent metal ion transporters), member 2 |
|  | CLN3 | ceroid-lipofuscinosis, neuronal 3 |
|  | AP1G1 | adaptor-related protein complex 1, gamma 1 subunit |
|  | CTSH | cathepsin H |
| **KEGG pathway----Tight junction----04530** | **Gene Symbol** | **Gene Name** |
|  | MYL9 | myosin, light chain 9, regulatory |
|  | AMOTL1 | angiomotin like 1 |
|  | AMOTL1 | angiomotin like 1 |
|  | CLDN23 | claudin 23 |
|  | CLDN23 | claudin 23 |
|  | PPP2R2A | protein phosphatase 2 (formerly 2A), regulatory subunit B, alpha isoform |
|  | ACTN1 | actinin, alpha 1 |
|  | ACTN1 | actinin, alpha 1 |
|  | ACTN1 | actinin, alpha 1 |
|  | EPB41L2 | erythrocyte membrane protein band 4.1-like 2 |
|  | EPB41L2 | erythrocyte membrane protein band 4.1-like 2 |
|  | SRC | v-src sarcoma (Schmidt-Ruppin A-2) viral oncogene homolog (avian) |
|  | CDC42 | cell division cycle 42 (GTP binding protein, 25kDa) |
|  | LLGL2 | lethal giant larvae homolog 2 (Drosophila) |
|  | LLGL2 | lethal giant larvae homolog 2 (Drosophila) |
|  | AKT3 | v-akt murine thymoma viral oncogene homolog 3 (protein kinase B, gamma) |
|  | AKT3 | v-akt murine thymoma viral oncogene homolog 3 (protein kinase B, gamma) |
|  | AKT3 | v-akt murine thymoma viral oncogene homolog 3 (protein kinase B, gamma) |
|  | AKT3 | v-akt murine thymoma viral oncogene homolog 3 (protein kinase B, gamma) |
|  | PRKCD | protein kinase C, delta |
|  | PRKCZ | protein kinase C, zeta |
|  | PRKCI | protein kinase C, iota |
|  | CLDN4 | claudin 4 |
|  | MYH14 | myosin, heavy chain 14 |
|  | MYH14 | myosin, heavy chain 14 |
|  | MYH14 | myosin, heavy chain 14 |
|  | MYH14 | myosin, heavy chain 14 |
|  | PPP2R2D | protein phosphatase 2, regulatory subunit B, delta isoform |
|  | ZAK | sterile alpha motif and leucine zipper containing kinase AZK |
|  | ZAK | sterile alpha motif and leucine zipper containing kinase AZK |
|  | ZAK | sterile alpha motif and leucine zipper containing kinase AZK |
| **KEGG pathway----Drug metabolism - cytochrome P450----00982** | **Gene Symbol** | **Gene Name** |
|  | UGT1A1 | UDP glucuronosyltransferase 1 family, polypeptide A1 |
|  | GSTK1 | glutathione S-transferase kappa 1 |
|  | ADH7 | alcohol dehydrogenase 7 (class IV), mu or sigma polypeptide |
|  | UGT1A9 | UDP glucuronosyltransferase 1 family, polypeptide A9 |
|  | CYP3A5 | cytochrome P450, family 3, subfamily A, polypeptide 5 |
|  | UGT1A6 | UDP glucuronosyltransferase 1 family, polypeptide A6 |
|  | MAOA | monoamine oxidase A |
|  | MAOA | monoamine oxidase A |
|  | MAOA | monoamine oxidase A |
|  | UGT1A8 | UDP glucuronosyltransferase 1 family, polypeptide A8 |
|  | MGST2 | microsomal glutathione S-transferase 2 |
|  | CYP2A7 | cytochrome P450, family 2, subfamily A, polypeptide 7 |
|  | UGT1A3 | UDP glucuronosyltransferase 1 family, polypeptide A3 |
|  | GSTA2 | glutathione S-transferase alpha 2 |
| **KEGG pathway----Pancreatic cancer----05212** | **Gene Symbol** | **Gene Name** |
|  | SMAD3 | SMAD family member 3 |
|  | SMAD3 | SMAD family member 3 |
|  | CDC42 | cell division cycle 42 (GTP binding protein, 25kDa) |
|  | ARHGEF6 | Rac/Cdc42 guanine nucleotide exchange factor (GEF) 6 |
|  | AKT3 | v-akt murine thymoma viral oncogene homolog 3 (protein kinase B, gamma) |
|  | AKT3 | v-akt murine thymoma viral oncogene homolog 3 (protein kinase B, gamma) |
|  | AKT3 | v-akt murine thymoma viral oncogene homolog 3 (protein kinase B, gamma) |
|  | AKT3 | v-akt murine thymoma viral oncogene homolog 3 (protein kinase B, gamma) |
|  | ACVR1B | activin A receptor, type IB |
|  | IKBKB | inhibitor of kappa light polypeptide gene enhancer in B-cells, kinase beta |
|  | MAPK9 | mitogen-activated protein kinase 9 |
|  | ERBB2 | v-erb-b2 erythroblastic leukemia viral oncogene homolog 2, neuro/glioblastoma derived oncogene homolog (avian) |
|  | TGFBR1 | transforming growth factor, beta receptor 1 |
|  | VEGFA | vascular endothelial growth factor A |
|  | VEGFA | vascular endothelial growth factor A |
|  | VEGFA | vascular endothelial growth factor A |
|  | MAPK10 | mitogen-activated protein kinase 10 |
|  | CDK6 | cyclin-dependent kinase 6 |
|  | CDK6 | cyclin-dependent kinase 6 |
|  | CDK6 | cyclin-dependent kinase 6 |
|  | CDK6 | cyclin-dependent kinase 6 |
| **KEGG pathway----TGF-beta signaling pathway----04350** | **Gene Symbol** | **Gene Name** |
|  | SMAD3 | SMAD family member 3 |
|  | SMAD3 | SMAD family member 3 |
|  | EP300 | E1A binding protein p300 |
|  | ID3 | inhibitor of DNA binding 3, dominant negative helix-loop-helix protein |
|  | ID1 | inhibitor of DNA binding 1, dominant negative helix-loop-helix protein |
|  | BMP7 | bone morphogenetic protein 7 |
|  | BMP7 | bone morphogenetic protein 7 |
|  | ACVR1B | activin A receptor, type IB |
|  | ACVR2A | activin A receptor, type IIA |
|  | TGFBR1 | transforming growth factor, beta receptor 1 |
|  | SMURF1 | SMAD specific E3 ubiquitin protein ligase 1 |
|  | SMAD6 | SMAD family member 6 |
|  | SMAD6 | SMAD family member 6 |
|  | INHBA | inhibin, beta A |
|  | ZFYVE9 | zinc finger, FYVE domain containing 9 |
|  | SP1 | Sp1 transcription factor |
| **KEGG pathway----Regulation of actin cytoskeleton----04810** | **Gene Symbol** | **Gene Name** |
|  | MYL9 | myosin, light chain 9, regulatory |
|  | PDGFC | platelet derived growth factor C |
|  | SSH3 | slingshot homolog 3 (Drosophila) |
|  | FN1 | fibronectin 1 |
|  | FN1 | fibronectin 1 |
|  | FN1 | fibronectin 1 |
|  | FN1 | fibronectin 1 |
|  | ARHGEF7 | Rho guanine nucleotide exchange factor (GEF) 7 |
|  | ENAH | enabled homolog (Drosophila) |
|  | ENAH | enabled homolog (Drosophila) |
|  | ACTN1 | actinin, alpha 1 |
|  | ACTN1 | actinin, alpha 1 |
|  | ACTN1 | actinin, alpha 1 |
|  | PTK2 | PTK2 protein tyrosine kinase 2 |
|  | CFL2 | cofilin 2 (muscle) |
|  | CFL2 | cofilin 2 (muscle) |
|  | FGFR1 | fibroblast growth factor receptor 1 |
|  | FGFR1 | fibroblast growth factor receptor 1 |
|  | PAK6 | p21 protein (Cdc42/Rac)-activated kinase 6 |
|  | FGFR3 | fibroblast growth factor receptor 3 |
|  | CDC42 | cell division cycle 42 (GTP binding protein, 25kDa) |
|  | ARHGEF6 | Rac/Cdc42 guanine nucleotide exchange factor (GEF) 6 |
|  | WASL | Wiskott-Aldrich syndrome-like |
|  | MYLK | myosin light chain kinase |
|  | PDGFD | platelet derived growth factor D |
|  | SLC9A1 | solute carrier family 9 (sodium/hydrogen exchanger), member 1 |
|  | ITGA11 | integrin, alpha 11 |
|  | MYH14 | myosin, heavy chain 14 |
|  | MYH14 | myosin, heavy chain 14 |
|  | MYH14 | myosin, heavy chain 14 |
|  | MYH14 | myosin, heavy chain 14 |
|  | IQGAP3 | IQ motif containing GTPase activating protein 3 |
| **KEGG pathway----Ascorbate and aldarate metabolism----00053** | **Gene Symbol** | **Gene Name** |
|  | UGT1A6 | UDP glucuronosyltransferase 1 family, polypeptide A6 |
|  | UGT1A1 | UDP glucuronosyltransferase 1 family, polypeptide A1 |
|  | UGT1A8 | UDP glucuronosyltransferase 1 family, polypeptide A8 |
|  | ALDH1B1 | aldehyde dehydrogenase 1 family, member B1 |
|  | UGT1A9 | UDP glucuronosyltransferase 1 family, polypeptide A9 |
|  | ALDH3A2 | aldehyde dehydrogenase 3 family, member A2 |
|  | UGT1A3 | UDP glucuronosyltransferase 1 family, polypeptide A3 |
| **KEGG pathway----MAPK signaling pathway----04010** | **Gene Symbol** | **Gene Name** |
|  | DUSP2 | dual specificity phosphatase 2 |
|  | RAPGEF2 | Rap guanine nucleotide exchange factor (GEF) 2 |
|  | ACVR1B | activin A receptor, type IB |
|  | MAP4K4 | mitogen-activated protein kinase kinase kinase kinase 4 |
|  | TGFBR1 | transforming growth factor, beta receptor 1 |
|  | FLNA | filamin A, alpha |
|  | FLNA | filamin A, alpha |
|  | DUSP14 | dual specificity phosphatase 14 |
|  | FGFR1 | fibroblast growth factor receptor 1 |
|  | FGFR1 | fibroblast growth factor receptor 1 |
|  | JUND | jun D proto-oncogene |
|  | FAS | Fas (TNF receptor superfamily, member 6) |
|  | FAS | Fas (TNF receptor superfamily, member 6) |
|  | FGFR3 | fibroblast growth factor receptor 3 |
|  | CDC42 | cell division cycle 42 (GTP binding protein, 25kDa) |
|  | AKT3 | v-akt murine thymoma viral oncogene homolog 3 (protein kinase B, gamma) |
|  | AKT3 | v-akt murine thymoma viral oncogene homolog 3 (protein kinase B, gamma) |
|  | AKT3 | v-akt murine thymoma viral oncogene homolog 3 (protein kinase B, gamma) |
|  | AKT3 | v-akt murine thymoma viral oncogene homolog 3 (protein kinase B, gamma) |
|  | IKBKB | inhibitor of kappa light polypeptide gene enhancer in B-cells, kinase beta |
|  | PTPRR | protein tyrosine phosphatase, receptor type, R |
|  | PTPRR | protein tyrosine phosphatase, receptor type, R |
|  | MAPK9 | mitogen-activated protein kinase 9 |
|  | CACNA1D | calcium channel, voltage-dependent, L type, alpha 1D subunit |
|  | CDC25B | cell division cycle 25 homolog B (S. pombe) |
|  | MAP3K5 | mitogen-activated protein kinase kinase kinase 5 |
|  | MAP3K7 | mitogen-activated protein kinase kinase kinase 7 |
|  | MAP3K12 | mitogen-activated protein kinase kinase kinase 12 |
|  | MAPK10 | mitogen-activated protein kinase 10 |
|  | ZAK | sterile alpha motif and leucine zipper containing kinase AZK |
|  | ZAK | sterile alpha motif and leucine zipper containing kinase AZK |
|  | ZAK | sterile alpha motif and leucine zipper containing kinase AZK |
| **KEGG pathway----Retinol metabolism----00830** | **Gene Symbol** | **Gene Name** |
|  | UGT1A1 | UDP glucuronosyltransferase 1 family, polypeptide A1 |
|  | ADH7 | alcohol dehydrogenase 7 (class IV), mu or sigma polypeptide |
|  | UGT1A9 | UDP glucuronosyltransferase 1 family, polypeptide A9 |
|  | DGAT2 | diacylglycerol O-acyltransferase homolog 2 (mouse) |
|  | CYP3A5 | cytochrome P450, family 3, subfamily A, polypeptide 5 |
|  | UGT1A6 | UDP glucuronosyltransferase 1 family, polypeptide A6 |
|  | UGT1A8 | UDP glucuronosyltransferase 1 family, polypeptide A8 |
|  | DHRS3 | dehydrogenase/reductase (SDR family) member 3 |
|  | CYP2A7 | cytochrome P450, family 2, subfamily A, polypeptide 7 |
|  | UGT1A3 | UDP glucuronosyltransferase 1 family, polypeptide A3 |
| **KEGG pathway----Drug metabolism - other enzymes----00983** | **Gene Symbol** | **Gene Name** |
|  | UGT1A1 | UDP glucuronosyltransferase 1 family, polypeptide A1 |
|  | TYMP | thymidine phosphorylase |
|  | GUSB | glucuronidase, beta |
|  | UGT1A9 | UDP glucuronosyltransferase 1 family, polypeptide A9 |
|  | CYP3A5 | cytochrome P450, family 3, subfamily A, polypeptide 5 |
|  | UGT1A6 | UDP glucuronosyltransferase 1 family, polypeptide A6 |
|  | UGT1A8 | UDP glucuronosyltransferase 1 family, polypeptide A8 |
|  | CYP2A7 | cytochrome P450, family 2, subfamily A, polypeptide 7 |
|  | UGT1A3 | UDP glucuronosyltransferase 1 family, polypeptide A3 |
| **KEGG pathway----Starch and sucrose metabolism----00500** | **Gene Symbol** | **Gene Name** |
|  | UGT1A1 | UDP glucuronosyltransferase 1 family, polypeptide A1 |
|  | GUSB | glucuronidase, beta |
|  | UGT1A9 | UDP glucuronosyltransferase 1 family, polypeptide A9 |
|  | PYGL | phosphorylase, glycogen, liver |
|  | PGM2L1 | phosphoglucomutase 2-like 1 |
|  | PGM2L1 | phosphoglucomutase 2-like 1 |
|  | UGT1A6 | UDP glucuronosyltransferase 1 family, polypeptide A6 |
|  | UGT1A8 | UDP glucuronosyltransferase 1 family, polypeptide A8 |
|  | HK1 | hexokinase 1 |
|  | UGT1A3 | UDP glucuronosyltransferase 1 family, polypeptide A3 |
| **KEGG pathway----Pathways in cancer----05200** | **Gene Symbol** | **Gene Name** |
|  | LAMA4 | laminin, alpha 4 |
|  | EP300 | E1A binding protein p300 |
|  | FN1 | fibronectin 1 |
|  | FN1 | fibronectin 1 |
|  | FN1 | fibronectin 1 |
|  | FN1 | fibronectin 1 |
|  | ACVR1B | activin A receptor, type IB |
|  | RXRA | retinoid X receptor, alpha |
|  | ERBB2 | v-erb-b2 erythroblastic leukemia viral oncogene homolog 2, neuro/glioblastoma derived oncogene homolog (avian) |
|  | RASSF5 | Ras association (RalGDS/AF-6) domain family member 5 |
|  | RASSF5 | Ras association (RalGDS/AF-6) domain family member 5 |
|  | TGFBR1 | transforming growth factor, beta receptor 1 |
|  | DVL3 | dishevelled, dsh homolog 3 (Drosophila) |
|  | WNT7B | wingless-type MMTV integration site family, member 7B |
|  | KLK3 | kallikrein-related peptidase 3 |
|  | PTK2 | PTK2 protein tyrosine kinase 2 |
|  | VEGFA | vascular endothelial growth factor A |
|  | VEGFA | vascular endothelial growth factor A |
|  | VEGFA | vascular endothelial growth factor A |
|  | FGFR1 | fibroblast growth factor receptor 1 |
|  | FGFR1 | fibroblast growth factor receptor 1 |
|  | FAS | Fas (TNF receptor superfamily, member 6) |
|  | FAS | Fas (TNF receptor superfamily, member 6) |
|  | SMAD3 | SMAD family member 3 |
|  | SMAD3 | SMAD family member 3 |
|  | FGFR3 | fibroblast growth factor receptor 3 |
|  | CDC42 | cell division cycle 42 (GTP binding protein, 25kDa) |
|  | HIF1A | hypoxia inducible factor 1, alpha subunit (basic helix-loop-helix transcription factor) |
|  | AKT3 | v-akt murine thymoma viral oncogene homolog 3 (protein kinase B, gamma) |
|  | AKT3 | v-akt murine thymoma viral oncogene homolog 3 (protein kinase B, gamma) |
|  | AKT3 | v-akt murine thymoma viral oncogene homolog 3 (protein kinase B, gamma) |
|  | AKT3 | v-akt murine thymoma viral oncogene homolog 3 (protein kinase B, gamma) |
|  | IKBKB | inhibitor of kappa light polypeptide gene enhancer in B-cells, kinase beta |
|  | MAPK9 | mitogen-activated protein kinase 9 |
|  | MAPK10 | mitogen-activated protein kinase 10 |
|  | CTBP1 | C-terminal binding protein 1 |
|  | CDK6 | cyclin-dependent kinase 6 |
|  | CDK6 | cyclin-dependent kinase 6 |
|  | CDK6 | cyclin-dependent kinase 6 |
|  | CDK6 | cyclin-dependent kinase 6 |
| **KEGG pathway----Fatty acid metabolism----00071** | **Gene Symbol** | **Gene Name** |
|  | ACSL5 | acyl-CoA synthetase long-chain family member 5 |
|  | ACSL5 | acyl-CoA synthetase long-chain family member 5 |
|  | ADH7 | alcohol dehydrogenase 7 (class IV), mu or sigma polypeptide |
|  | ACADSB | acyl-Coenzyme A dehydrogenase, short/branched chain |
|  | ACADSB | acyl-Coenzyme A dehydrogenase, short/branched chain |
|  | ACOX1 | acyl-Coenzyme A oxidase 1, palmitoyl |
|  | ACAA2 | acetyl-Coenzyme A acyltransferase 2 |
|  | ALDH1B1 | aldehyde dehydrogenase 1 family, member B1 |
|  | ACADVL | acyl-Coenzyme A dehydrogenase, very long chain |
|  | ALDH3A2 | aldehyde dehydrogenase 3 family, member A2 |
| **KEGG pathway----Metabolism of xenobiotics by cytochrome P450----00980** | **Gene Symbol** | **Gene Name** |
|  | UGT1A1 | UDP glucuronosyltransferase 1 family, polypeptide A1 |
|  | GSTK1 | glutathione S-transferase kappa 1 |
|  | ADH7 | alcohol dehydrogenase 7 (class IV), mu or sigma polypeptide |
|  | UGT1A9 | UDP glucuronosyltransferase 1 family, polypeptide A9 |
|  | CYP3A5 | cytochrome P450, family 3, subfamily A, polypeptide 5 |
|  | UGT1A6 | UDP glucuronosyltransferase 1 family, polypeptide A6 |
|  | UGT1A8 | UDP glucuronosyltransferase 1 family, polypeptide A8 |
|  | MGST2 | microsomal glutathione S-transferase 2 |
|  | UGT1A3 | UDP glucuronosyltransferase 1 family, polypeptide A3 |
|  | GSTA2 | glutathione S-transferase alpha 2 |
| **KEGG pathway----Androgen and estrogen metabolism----00150** | **Gene Symbol** | **Gene Name** |
|  | UGT1A6 | UDP glucuronosyltransferase 1 family, polypeptide A6 |
|  | UGT1A1 | UDP glucuronosyltransferase 1 family, polypeptide A1 |
|  | UGT1A8 | UDP glucuronosyltransferase 1 family, polypeptide A8 |
|  | HEMK1 | HemK methyltransferase family member 1 |
|  | HEMK1 | HemK methyltransferase family member 1 |
|  | UGT1A9 | UDP glucuronosyltransferase 1 family, polypeptide A9 |
|  | SRD5A1 | steroid-5-alpha-reductase, alpha polypeptide 1 (3-oxo-5 alpha-steroid delta 4-dehydrogenase alpha 1) |
|  | SRD5A1 | steroid-5-alpha-reductase, alpha polypeptide 1 (3-oxo-5 alpha-steroid delta 4-dehydrogenase alpha 1) |
|  | UGT1A3 | UDP glucuronosyltransferase 1 family, polypeptide A3 |
|  | HSD17B2 | hydroxysteroid (17-beta) dehydrogenase 2 |
| **KEGG pathway----Butanoate metabolism----00650** | **Gene Symbol** | **Gene Name** |
|  | HMGCL | 3-hydroxymethyl-3-methylglutaryl-Coenzyme A lyase |
|  | ALDH5A1 | aldehyde dehydrogenase 5 family, member A1 |
|  | ABAT | 4-aminobutyrate aminotransferase |
|  | ABAT | 4-aminobutyrate aminotransferase |
|  | ALDH1B1 | aldehyde dehydrogenase 1 family, member B1 |
|  | OXCT1 | 3-oxoacid CoA transferase 1 |
|  | ALDH3A2 | aldehyde dehydrogenase 3 family, member A2 |
|  | HMGCS2 | 3-hydroxy-3-methylglutaryl-Coenzyme A synthase 2 (mitochondrial) |
| **KEGG pathway----Fructose and mannose metabolism----00051** | **Gene Symbol** | **Gene Name** |
|  | FUK | fucokinase |
|  | HK1 | hexokinase 1 |
|  | PFKL | phosphofructokinase, liver |
|  | SORD | sorbitol dehydrogenase |
|  | PHPT1 | phosphohistidine phosphatase 1 |
|  | FBP1 | fructose-1,6-bisphosphatase 1 |
|  | PFKP | phosphofructokinase, platelet |
| **KEGG pathway----Ubiquitin mediated proteolysis----04120** | **Gene Symbol** | **Gene Name** |
|  | CUL5 | cullin 5 |
|  | CUL7 | cullin 7 |
|  | CUL7 | cullin 7 |
|  | CDC27 | cell division cycle 27 homolog (S. cerevisiae) |
|  | SIAH1 | seven in absentia homolog 1 (Drosophila) |
|  | SIAH1 | seven in absentia homolog 1 (Drosophila) |
|  | ITCH | itchy E3 ubiquitin protein ligase homolog (mouse) |
|  | UBE2H | ubiquitin-conjugating enzyme E2H (UBC8 homolog, yeast) |
|  | UBE2H | ubiquitin-conjugating enzyme E2H (UBC8 homolog, yeast) |
|  | RHOBTB2 | Rho-related BTB domain containing 2 |
|  | UBE2B | ubiquitin-conjugating enzyme E2B (RAD6 homolog) |
|  | ANAPC7 | anaphase promoting complex subunit 7 |
|  | UBE4B | ubiquitination factor E4B (UFD2 homolog, yeast) |
|  | SMURF1 | SMAD specific E3 ubiquitin protein ligase 1 |
|  | UBE2K | ubiquitin-conjugating enzyme E2K (UBC1 homolog, yeast) |
|  | BTRC | beta-transducin repeat containing |
|  | WWP2 | WW domain containing E3 ubiquitin protein ligase 2 |
| **KEGG pathway----Type II diabetes mellitus----04930** | **Gene Symbol** | **Gene Name** |
|  | INSR | insulin receptor |
|  | PRKCD | protein kinase C, delta |
|  | IKBKB | inhibitor of kappa light polypeptide gene enhancer in B-cells, kinase beta |
|  | MAPK9 | mitogen-activated protein kinase 9 |
|  | PRKCZ | protein kinase C, zeta |
|  | CACNA1D | calcium channel, voltage-dependent, L type, alpha 1D subunit |
|  | HK1 | hexokinase 1 |
|  | MAPK10 | mitogen-activated protein kinase 10 |
| **KEGG pathway----Pentose and glucuronate interconversions----00040** | **Gene Symbol** | **Gene Name** |
|  | UGT1A6 | UDP glucuronosyltransferase 1 family, polypeptide A6 |
|  | UGT1A1 | UDP glucuronosyltransferase 1 family, polypeptide A1 |
|  | UGT1A8 | UDP glucuronosyltransferase 1 family, polypeptide A8 |
|  | GUSB | glucuronidase, beta |
|  | UGT1A9 | UDP glucuronosyltransferase 1 family, polypeptide A9 |
|  | UGT1A3 | UDP glucuronosyltransferase 1 family, polypeptide A3 |
| **KEGG pathway----Huntington's disease----05016** | **Gene Symbol** | **Gene Name** |
|  | NDUFA4L2 | NADH dehydrogenase (ubiquinone) 1 alpha subcomplex, 4-like 2 |
|  | EP300 | E1A binding protein p300 |
|  | NDUFV1 | NADH dehydrogenase (ubiquinone) flavoprotein 1, 51kDa |
|  | NDUFS1 | NADH dehydrogenase (ubiquinone) Fe-S protein 1, 75kDa (NADH-coenzyme Q reductase) |
|  | POLR2J | polymerase (RNA) II (DNA directed) polypeptide J, 13.3kDa |
|  | DCTN2 | dynactin 2 (p50) |
|  | NDUFS6 | NADH dehydrogenase (ubiquinone) Fe-S protein 6, 13kDa (NADH-coenzyme Q reductase) |
|  | SOD2 | superoxide dismutase 2, mitochondrial |
|  | SOD2 | superoxide dismutase 2, mitochondrial |
|  | NDUFB8 | NADH dehydrogenase (ubiquinone) 1 beta subcomplex, 8, 19kDa |
|  | AP2B1 | adaptor-related protein complex 2, beta 1 subunit |
|  | COX5A | cytochrome c oxidase subunit Va |
|  | NDUFV3 | NADH dehydrogenase (ubiquinone) flavoprotein 3, 10kDa |
|  | COX4I1 | cytochrome c oxidase subunit IV isoform 1 |
|  | COX4I1 | cytochrome c oxidase subunit IV isoform 1 |
|  | NDUFA3 | NADH dehydrogenase (ubiquinone) 1 alpha subcomplex, 3, 9kDa |
|  | SDHA | succinate dehydrogenase complex, subunit A, flavoprotein (Fp) |
|  | SP1 | Sp1 transcription factor |
| **KEGG pathway----ECM-receptor interaction----04512** | **Gene Symbol** | **Gene Name** |
|  | LAMA4 | laminin, alpha 4 |
|  | FN1 | fibronectin 1 |
|  | FN1 | fibronectin 1 |
|  | FN1 | fibronectin 1 |
|  | FN1 | fibronectin 1 |
|  | COL1A1 | collagen, type I, alpha 1 |
|  | CD47 | CD47 molecule |
|  | CD47 | CD47 molecule |
|  | COL5A1 | collagen, type V, alpha 1 |
|  | COL5A1 | collagen, type V, alpha 1 |
|  | ITGA11 | integrin, alpha 11 |
|  | COL11A1 | collagen, type XI, alpha 1 |
|  | COL11A1 | collagen, type XI, alpha 1 |
|  | COL1A2 | collagen, type I, alpha 2 |
|  | COL1A2 | collagen, type I, alpha 2 |
|  | COL1A2 | collagen, type I, alpha 2 |
|  | DAG1 | dystroglycan 1 (dystrophin-associated glycoprotein 1) |
|  | COL3A1 | collagen, type III, alpha 1 |
| **KEGG pathway----Glycerolipid metabolism----00561** | **Gene Symbol** | **Gene Name** |
|  | LIPG | lipase, endothelial |
|  | ALDH1B1 | aldehyde dehydrogenase 1 family, member B1 |
|  | PPAP2B | phosphatidic acid phosphatase type 2B |
|  | ALDH3A2 | aldehyde dehydrogenase 3 family, member A2 |
|  | DGKA | diacylglycerol kinase, alpha 80kDa |
|  | PPAP2C | phosphatidic acid phosphatase type 2C |
|  | DGAT2 | diacylglycerol O-acyltransferase homolog 2 (mouse) |
| **KEGG pathway----beta-Alanine metabolism----00410** | **Gene Symbol** | **Gene Name** |
|  | MLYCD | malonyl-CoA decarboxylase |
|  | ABAT | 4-aminobutyrate aminotransferase |
|  | ABAT | 4-aminobutyrate aminotransferase |
|  | ALDH1B1 | aldehyde dehydrogenase 1 family, member B1 |
|  | ALDH3A2 | aldehyde dehydrogenase 3 family, member A2 |
|  | SMS | spermine synthase |
| **KEGG pathway----Sulfur metabolism----00920** | **Gene Symbol** | **Gene Name** |
|  | CHST11 | carbohydrate (chondroitin 4) sulfotransferase 11 |
|  | CHST11 | carbohydrate (chondroitin 4) sulfotransferase 11 |
|  | SUOX | sulfite oxidase |
|  | PAPSS2 | 3'-phosphoadenosine 5'-phosphosulfate synthase 2 |
|  | PAPSS2 | 3'-phosphoadenosine 5'-phosphosulfate synthase 2 |
|  | SULT1A1 | sulfotransferase family, cytosolic, 1A, phenol-preferring, member 1 |
| **KEGG pathway----Alzheimer's disease----05010** | **Gene Symbol** | **Gene Name** |
|  | NDUFA4L2 | NADH dehydrogenase (ubiquinone) 1 alpha subcomplex, 4-like 2 |
|  | NDUFV1 | NADH dehydrogenase (ubiquinone) flavoprotein 1, 51kDa |
|  | NDUFS1 | NADH dehydrogenase (ubiquinone) Fe-S protein 1, 75kDa (NADH-coenzyme Q reductase) |
|  | NDUFS6 | NADH dehydrogenase (ubiquinone) Fe-S protein 6, 13kDa (NADH-coenzyme Q reductase) |
|  | CALM1 | calmodulin 1 (phosphorylase kinase, delta) |
|  | CALM1 | calmodulin 1 (phosphorylase kinase, delta) |
|  | NDUFB8 | NADH dehydrogenase (ubiquinone) 1 beta subcomplex, 8, 19kDa |
|  | COX5A | cytochrome c oxidase subunit Va |
|  | COX4I1 | cytochrome c oxidase subunit IV isoform 1 |
|  | COX4I1 | cytochrome c oxidase subunit IV isoform 1 |
|  | NDUFV3 | NADH dehydrogenase (ubiquinone) flavoprotein 3, 10kDa |
|  | CACNA1D | calcium channel, voltage-dependent, L type, alpha 1D subunit |
|  | NDUFA3 | NADH dehydrogenase (ubiquinone) 1 alpha subcomplex, 3, 9kDa |
|  | SDHA | succinate dehydrogenase complex, subunit A, flavoprotein (Fp) |
|  | MME | membrane metallo-endopeptidase |
|  | MME | membrane metallo-endopeptidase |
|  | FAS | Fas (TNF receptor superfamily, member 6) |
|  | FAS | Fas (TNF receptor superfamily, member 6) |
| **KEGG pathway----Wnt signaling pathway----04310** | **Gene Symbol** | **Gene Name** |
|  | SMAD3 | SMAD family member 3 |
|  | SMAD3 | SMAD family member 3 |
|  | EP300 | E1A binding protein p300 |
|  | SIAH1 | seven in absentia homolog 1 (Drosophila) |
|  | SIAH1 | seven in absentia homolog 1 (Drosophila) |
|  | MAPK9 | mitogen-activated protein kinase 9 |
|  | DVL3 | dishevelled, dsh homolog 3 (Drosophila) |
|  | WNT7B | wingless-type MMTV integration site family, member 7B |
|  | MAP3K7 | mitogen-activated protein kinase kinase kinase 7 |
|  | MAPK10 | mitogen-activated protein kinase 10 |
|  | CTBP1 | C-terminal binding protein 1 |
|  | DAAM1 | dishevelled associated activator of morphogenesis 1 |
|  | NFAT5 | nuclear factor of activated T-cells 5, tonicity-responsive |
|  | NFAT5 | nuclear factor of activated T-cells 5, tonicity-responsive |
|  | NFAT5 | nuclear factor of activated T-cells 5, tonicity-responsive |
|  | CAMK2G | calcium/calmodulin-dependent protein kinase II gamma |
|  | CAMK2G | calcium/calmodulin-dependent protein kinase II gamma |
|  | BTRC | beta-transducin repeat containing |
| **KEGG pathway----Adipocytokine signaling pathway----04920** | **Gene Symbol** | **Gene Name** |
|  | ACSL5 | acyl-CoA synthetase long-chain family member 5 |
|  | ACSL5 | acyl-CoA synthetase long-chain family member 5 |
|  | PRKAB1 | protein kinase, AMP-activated, beta 1 non-catalytic subunit |
|  | AKT3 | v-akt murine thymoma viral oncogene homolog 3 (protein kinase B, gamma) |
|  | AKT3 | v-akt murine thymoma viral oncogene homolog 3 (protein kinase B, gamma) |
|  | AKT3 | v-akt murine thymoma viral oncogene homolog 3 (protein kinase B, gamma) |
|  | AKT3 | v-akt murine thymoma viral oncogene homolog 3 (protein kinase B, gamma) |
|  | IKBKB | inhibitor of kappa light polypeptide gene enhancer in B-cells, kinase beta |
|  | RXRA | retinoid X receptor, alpha |
|  | MAPK9 | mitogen-activated protein kinase 9 |
|  | MAPK10 | mitogen-activated protein kinase 10 |
|  | LEPR | leptin receptor |
| **KEGG pathway----PPAR signaling pathway----03320** | **Gene Symbol** | **Gene Name** |
|  | ACSL5 | acyl-CoA synthetase long-chain family member 5 |
|  | ACSL5 | acyl-CoA synthetase long-chain family member 5 |
|  | PDPK1 | 3-phosphoinositide dependent protein kinase-1 |
|  | SORBS1 | sorbin and SH3 domain containing 1 |
|  | SCD5 | stearoyl-CoA desaturase 5 |
|  | ACOX1 | acyl-Coenzyme A oxidase 1, palmitoyl |
|  | RXRA | retinoid X receptor, alpha |
|  | HMGCS2 | 3-hydroxy-3-methylglutaryl-Coenzyme A synthase 2 (mitochondrial) |
|  | UBC | ubiquitin C |
| **KEGG pathway----Arginine and proline metabolism----00330** | **Gene Symbol** | **Gene Name** |
|  | MAOA | monoamine oxidase A |
|  | MAOA | monoamine oxidase A |
|  | MAOA | monoamine oxidase A |
|  | ARG2 | arginase, type II |
|  | ARG2 | arginase, type II |
|  | GLUD1 | glutamate dehydrogenase 1 |
|  | ALDH1B1 | aldehyde dehydrogenase 1 family, member B1 |
|  | GOT2 | glutamic-oxaloacetic transaminase 2, mitochondrial (aspartate aminotransferase 2) |
|  | ALDH3A2 | aldehyde dehydrogenase 3 family, member A2 |
|  | SMS | spermine synthase |
| **KEGG pathway----Sphingolipid metabolism----00600** | **Gene Symbol** | **Gene Name** |
|  | ASAH1 | N-acylsphingosine amidohydrolase (acid ceramidase) 1 |
|  | SGMS2 | sphingomyelin synthase 2 |
|  | PPAP2B | phosphatidic acid phosphatase type 2B |
|  | DEGS1 | degenerative spermatocyte homolog 1, lipid desaturase (Drosophila) |
|  | DEGS1 | degenerative spermatocyte homolog 1, lipid desaturase (Drosophila) |
|  | PPAP2C | phosphatidic acid phosphatase type 2C |
|  | SGPL1 | sphingosine-1-phosphate lyase 1 |
|  | SGPL1 | sphingosine-1-phosphate lyase 1 |
| **KEGG pathway----Glycerophospholipid metabolism----00564** | **Gene Symbol** | **Gene Name** |
|  | GDE1 | glycerophosphodiester phosphodiesterase 1 |
|  | PPAP2B | phosphatidic acid phosphatase type 2B |
|  | DGKA | diacylglycerol kinase, alpha 80kDa |
|  | PPAP2C | phosphatidic acid phosphatase type 2C |
|  | ETNK1 | ethanolamine kinase 1 |
|  | PCYT2 | phosphate cytidylyltransferase 2, ethanolamine |
|  | CHKB | choline kinase beta |
|  | CHPT1 | choline phosphotransferase 1 |
|  | CHPT1 | choline phosphotransferase 1 |
|  | CHPT1 | choline phosphotransferase 1 |
| **KEGG pathway----ErbB signaling pathway----04012** | **Gene Symbol** | **Gene Name** |
|  | PAK6 | p21 protein (Cdc42/Rac)-activated kinase 6 |
|  | AKT3 | v-akt murine thymoma viral oncogene homolog 3 (protein kinase B, gamma) |
|  | AKT3 | v-akt murine thymoma viral oncogene homolog 3 (protein kinase B, gamma) |
|  | AKT3 | v-akt murine thymoma viral oncogene homolog 3 (protein kinase B, gamma) |
|  | AKT3 | v-akt murine thymoma viral oncogene homolog 3 (protein kinase B, gamma) |
|  | MAPK9 | mitogen-activated protein kinase 9 |
|  | ERBB2 | v-erb-b2 erythroblastic leukemia viral oncogene homolog 2, neuro/glioblastoma derived oncogene homolog (avian) |
|  | ERBB3 | v-erb-b2 erythroblastic leukemia viral oncogene homolog 3 (avian) |
|  | ERBB3 | v-erb-b2 erythroblastic leukemia viral oncogene homolog 3 (avian) |
|  | PTK2 | PTK2 protein tyrosine kinase 2 |
|  | MAPK10 | mitogen-activated protein kinase 10 |
|  | SRC | v-src sarcoma (Schmidt-Ruppin A-2) viral oncogene homolog (avian) |
|  | CAMK2G | calcium/calmodulin-dependent protein kinase II gamma |
|  | CAMK2G | calcium/calmodulin-dependent protein kinase II gamma |
| **KEGG pathway----Porphyrin and chlorophyll metabolism----00860** | **Gene Symbol** | **Gene Name** |
|  | UGT1A6 | UDP glucuronosyltransferase 1 family, polypeptide A6 |
|  | UGT1A1 | UDP glucuronosyltransferase 1 family, polypeptide A1 |
|  | UGT1A8 | UDP glucuronosyltransferase 1 family, polypeptide A8 |
|  | GUSB | glucuronidase, beta |
|  | UGT1A9 | UDP glucuronosyltransferase 1 family, polypeptide A9 |
|  | UGT1A3 | UDP glucuronosyltransferase 1 family, polypeptide A3 |
| **KEGG pathway----Cytokine-cytokine receptor interaction----04060** | **Gene Symbol** | **Gene Name** |
|  | PDGFC | platelet derived growth factor C |
|  | ACVR1B | activin A receptor, type IB |
|  | TNFRSF21 | tumor necrosis factor receptor superfamily, member 21 |
|  | CXCL6 | chemokine (C-X-C motif) ligand 6 (granulocyte chemotactic protein 2) |
|  | TGFBR1 | transforming growth factor, beta receptor 1 |
|  | IL13RA1 | interleukin 13 receptor, alpha 1 |
|  | VEGFA | vascular endothelial growth factor A |
|  | VEGFA | vascular endothelial growth factor A |
|  | VEGFA | vascular endothelial growth factor A |
|  | LEPR | leptin receptor |
|  | FAS | Fas (TNF receptor superfamily, member 6) |
|  | FAS | Fas (TNF receptor superfamily, member 6) |
|  | BMP7 | bone morphogenetic protein 7 |
|  | BMP7 | bone morphogenetic protein 7 |
|  | CXCL1 | chemokine (C-X-C motif) ligand 1 (melanoma growth stimulating activity, alpha) |
|  | ACVR2A | activin A receptor, type IIA |
|  | CXCL14 | chemokine (C-X-C motif) ligand 14 |
|  | CXCL14 | chemokine (C-X-C motif) ligand 14 |
|  | TNFSF15 | tumor necrosis factor (ligand) superfamily, member 15 |
|  | CCL3 | chemokine (C-C motif) ligand 3 |
|  | IL20RA | interleukin 20 receptor, alpha |
|  | IL20RA | interleukin 20 receptor, alpha |
|  | CCL11 | chemokine (C-C motif) ligand 11 |
|  | INHBA | inhibin, beta A |
| **KEGG pathway----Prostate cancer----05215** | **Gene Symbol** | **Gene Name** |
|  | PDPK1 | 3-phosphoinositide dependent protein kinase-1 |
|  | EP300 | E1A binding protein p300 |
|  | PDGFC | platelet derived growth factor C |
|  | AKT3 | v-akt murine thymoma viral oncogene homolog 3 (protein kinase B, gamma) |
|  | AKT3 | v-akt murine thymoma viral oncogene homolog 3 (protein kinase B, gamma) |
|  | AKT3 | v-akt murine thymoma viral oncogene homolog 3 (protein kinase B, gamma) |
|  | AKT3 | v-akt murine thymoma viral oncogene homolog 3 (protein kinase B, gamma) |
|  | IKBKB | inhibitor of kappa light polypeptide gene enhancer in B-cells, kinase beta |
|  | ERBB2 | v-erb-b2 erythroblastic leukemia viral oncogene homolog 2, neuro/glioblastoma derived oncogene homolog (avian) |
|  | PDGFD | platelet derived growth factor D |
|  | KLK3 | kallikrein-related peptidase 3 |
|  | FGFR1 | fibroblast growth factor receptor 1 |
|  | FGFR1 | fibroblast growth factor receptor 1 |
| **KEGG pathway----Histidine metabolism----00340** | **Gene Symbol** | **Gene Name** |
|  | MAOA | monoamine oxidase A |
|  | MAOA | monoamine oxidase A |
|  | MAOA | monoamine oxidase A |
|  | HEMK1 | HemK methyltransferase family member 1 |
|  | HEMK1 | HemK methyltransferase family member 1 |
|  | ALDH1B1 | aldehyde dehydrogenase 1 family, member B1 |
|  | HNMT | histamine N-methyltransferase |
|  | HNMT | histamine N-methyltransferase |
|  | ALDH3A2 | aldehyde dehydrogenase 3 family, member A2 |
| **KEGG pathway----Neurotrophin signaling pathway----04722** | **Gene Symbol** | **Gene Name** |
|  | CDC42 | cell division cycle 42 (GTP binding protein, 25kDa) |
|  | AKT3 | v-akt murine thymoma viral oncogene homolog 3 (protein kinase B, gamma) |
|  | AKT3 | v-akt murine thymoma viral oncogene homolog 3 (protein kinase B, gamma) |
|  | AKT3 | v-akt murine thymoma viral oncogene homolog 3 (protein kinase B, gamma) |
|  | AKT3 | v-akt murine thymoma viral oncogene homolog 3 (protein kinase B, gamma) |
|  | PRKCD | protein kinase C, delta |
|  | IKBKB | inhibitor of kappa light polypeptide gene enhancer in B-cells, kinase beta |
|  | IRAK2 | interleukin-1 receptor-associated kinase 2 |
|  | CALM1 | calmodulin 1 (phosphorylase kinase, delta) |
|  | CALM1 | calmodulin 1 (phosphorylase kinase, delta) |
|  | MAPK9 | mitogen-activated protein kinase 9 |
|  | YWHAQ | tyrosine 3-monooxygenase/tryptophan 5-monooxygenase activation protein, theta polypeptide |
|  | MAP3K5 | mitogen-activated protein kinase kinase kinase 5 |
|  | MAPK10 | mitogen-activated protein kinase 10 |
|  | CAMK2G | calcium/calmodulin-dependent protein kinase II gamma |
|  | CAMK2G | calcium/calmodulin-dependent protein kinase II gamma |
| **KEGG pathway----Phosphatidylinositol signaling system----04070** | **Gene Symbol** | **Gene Name** |
|  | INPP5K | inositol polyphosphate-5-phosphatase K |
|  | INPP5K | inositol polyphosphate-5-phosphatase K |
|  | INPP5A | inositol polyphosphate-5-phosphatase, 40kDa |
|  | CALM1 | calmodulin 1 (phosphorylase kinase, delta) |
|  | CALM1 | calmodulin 1 (phosphorylase kinase, delta) |
|  | DGKA | diacylglycerol kinase, alpha 80kDa |
|  | PLCE1 | phospholipase C, epsilon 1 |
|  | PIK3C2B | phosphoinositide-3-kinase, class 2, beta polypeptide |
|  | PI4KA | phosphatidylinositol 4-kinase, catalytic, alpha |
|  | IMPA2 | inositol(myo)-1(or 4)-monophosphatase 2 |
| **KEGG pathway----Synthesis and degradation of ketone bodies----00072** | **Gene Symbol** | **Gene Name** |
|  | HMGCL | 3-hydroxymethyl-3-methylglutaryl-Coenzyme A lyase |
|  | OXCT1 | 3-oxoacid CoA transferase 1 |
|  | HMGCS2 | 3-hydroxy-3-methylglutaryl-Coenzyme A synthase 2 (mitochondrial) |
| **KEGG pathway----Chemokine signaling pathway----04062** | **Gene Symbol** | **Gene Name** |
|  | CDC42 | cell division cycle 42 (GTP binding protein, 25kDa) |
|  | WASL | Wiskott-Aldrich syndrome-like |
|  | GNB4 | guanine nucleotide binding protein (G protein), beta polypeptide 4 |
|  | AKT3 | v-akt murine thymoma viral oncogene homolog 3 (protein kinase B, gamma) |
|  | AKT3 | v-akt murine thymoma viral oncogene homolog 3 (protein kinase B, gamma) |
|  | AKT3 | v-akt murine thymoma viral oncogene homolog 3 (protein kinase B, gamma) |
|  | AKT3 | v-akt murine thymoma viral oncogene homolog 3 (protein kinase B, gamma) |
|  | PRKCD | protein kinase C, delta |
|  | IKBKB | inhibitor of kappa light polypeptide gene enhancer in B-cells, kinase beta |
|  | CXCL6 | chemokine (C-X-C motif) ligand 6 (granulocyte chemotactic protein 2) |
|  | PRKCZ | protein kinase C, zeta |
|  | CXCL14 | chemokine (C-X-C motif) ligand 14 |
|  | CXCL14 | chemokine (C-X-C motif) ligand 14 |
|  | PTK2B | PTK2B protein tyrosine kinase 2 beta |
|  | CXCL1 | chemokine (C-X-C motif) ligand 1 (melanoma growth stimulating activity, alpha) |
|  | CCL3 | chemokine (C-C motif) ligand 3 |
|  | PTK2 | PTK2 protein tyrosine kinase 2 |
|  | CCL11 | chemokine (C-C motif) ligand 11 |
| **KEGG pathway----Parkinson's disease----05012** | **Gene Symbol** | **Gene Name** |
|  | NDUFA4L2 | NADH dehydrogenase (ubiquinone) 1 alpha subcomplex, 4-like 2 |
|  | NDUFV1 | NADH dehydrogenase (ubiquinone) flavoprotein 1, 51kDa |
|  | NDUFS1 | NADH dehydrogenase (ubiquinone) Fe-S protein 1, 75kDa (NADH-coenzyme Q reductase) |
|  | NDUFS6 | NADH dehydrogenase (ubiquinone) Fe-S protein 6, 13kDa (NADH-coenzyme Q reductase) |
|  | NDUFB8 | NADH dehydrogenase (ubiquinone) 1 beta subcomplex, 8, 19kDa |
|  | COX5A | cytochrome c oxidase subunit Va |
|  | NDUFV3 | NADH dehydrogenase (ubiquinone) flavoprotein 3, 10kDa |
|  | COX4I1 | cytochrome c oxidase subunit IV isoform 1 |
|  | COX4I1 | cytochrome c oxidase subunit IV isoform 1 |
|  | NDUFA3 | NADH dehydrogenase (ubiquinone) 1 alpha subcomplex, 3, 9kDa |
|  | SDHA | succinate dehydrogenase complex, subunit A, flavoprotein (Fp) |
|  | HTRA2 | HtrA serine peptidase 2 |
| **KEGG pathway----Glycolysis / Gluconeogenesis----00010** | **Gene Symbol** | **Gene Name** |
|  | ADH7 | alcohol dehydrogenase 7 (class IV), mu or sigma polypeptide |
|  | HK1 | hexokinase 1 |
|  | ALDH1B1 | aldehyde dehydrogenase 1 family, member B1 |
|  | PFKL | phosphofructokinase, liver |
|  | ALDH3A2 | aldehyde dehydrogenase 3 family, member A2 |
|  | FBP1 | fructose-1,6-bisphosphatase 1 |
|  | PFKP | phosphofructokinase, platelet |
| **KEGG pathway----Fc gamma R-mediated phagocytosis----04666** | **Gene Symbol** | **Gene Name** |
|  | CDC42 | cell division cycle 42 (GTP binding protein, 25kDa) |
|  | MYO10 | myosin X |
|  | WASL | Wiskott-Aldrich syndrome-like |
|  | PPAP2B | phosphatidic acid phosphatase type 2B |
|  | AKT3 | v-akt murine thymoma viral oncogene homolog 3 (protein kinase B, gamma) |
|  | AKT3 | v-akt murine thymoma viral oncogene homolog 3 (protein kinase B, gamma) |
|  | AKT3 | v-akt murine thymoma viral oncogene homolog 3 (protein kinase B, gamma) |
|  | AKT3 | v-akt murine thymoma viral oncogene homolog 3 (protein kinase B, gamma) |
|  | PRKCD | protein kinase C, delta |
|  | PPAP2C | phosphatidic acid phosphatase type 2C |
|  | DNM2 | dynamin 2 |
|  | CFL2 | cofilin 2 (muscle) |
|  | CFL2 | cofilin 2 (muscle) |
| **KEGG pathway----Notch signaling pathway----04330** | **Gene Symbol** | **Gene Name** |
|  | EP300 | E1A binding protein p300 |
|  | HES1 | hairy and enhancer of split 1, (Drosophila) |
|  | DVL3 | dishevelled, dsh homolog 3 (Drosophila) |
|  | MAML1 | mastermind-like 1 (Drosophila) |
|  | CTBP1 | C-terminal binding protein 1 |
|  | MAML3 | mastermind-like 3 (Drosophila) |
